# Correction: Cohort profile of the Sloane Project: methodology for a prospective UK cohort study of >15 000 women with screen-detected non-invasive breast neoplasia

**DOI:** 10.1136/bmjopen-2022-061585corr1

**Published:** 2023-01-13

Clements K, Dodwell D, Hilton B, *et al*. Cohort profile of the Sloane Project: methodology for a prospective UK cohort study of >15 000 women with screen-detected non-invasive breast neoplasia. *BMJ Open* 2022;12:e061585. doi:10.1136/ bmjopen-2022-061585

This article has been corrected since it was published online. The sentence has been changed from ‘Coding in other linked datasets may be inaccurate leading to overestimation of relative proportion of DCIS recurrences’ to ‘Coding in other linked datasets may be inaccurate leading to overestimation of relative proportion of DCIS recurring as invasive disease compared with recurring as DCIS’ in the Strengths and Limitations box.

