# Supplementary material for: Cohort profile of the Sloane Project: methodology for a prospective UK cohort study of >15 000 women with screen-detected non-invasive breast neoplasia
Source: BMJ Open. 2022 Dec 19;12(12):e061585. doi: 10.1136/bmjopen-2022-061585 (PMC9764674; doi:10.1136/bmjopen-2022-061585)
Supplement: Supplementary data [file bmjopen-2022-061585supp001.pdf]

Sloane Project: Radiology Data Form

**Surname**	☐	**Forename(s)**	☐
**Date of Birth**	☐	**Screening Unit**	☐
**Screening Number (Sx)**	☐	**Screening Round**	☐
**Date of Screening Mammogram**	☐	**NHS Number**	☐

Side: **Left** ☐ **Right** ☐ ***For bilateral disease, please use separate form for each breast.***

Site (Tick all that are involved)

Upper outer quadrant ☐ Upper inner quadrant ☐

Lower outer quadrant ☐ Lower inner quadrant ☐ Retroareolar ☐

Background Pattern (Tick ONE only)

Fatty ☐ Scattered fibroglandular density ☐

Hetrogeneously dense ☐ Extremely dense ☐

Predominant Radiological Feature (Tick ONE only)

Calcification ☐ Parenchymal Distortion ☐

Mass – Well Defined ☐ Spiculate Mass ☐

Mass – Ill Defined ☐ None of the above ☐

Does the lesion contain microcalcification? **Yes** ☐ **No** ☐

If "Yes", what is the most suspicious pattern? (Tick ONE only)

Cast/linear / Casting ☐ Granular/irregular / Crushed Stone ☐ Punctate / Powderish ☐

Sloane Project Number (For Official Use Only) ☐

QA Ref Number (For Official Use Only) ☐

Version 2: Issued 01/08/2009

Estimated Size of Lesion (in mm)

Mammogram

	**obl**	**cc**
Distance from base of nipple to nearest part of the lesion		
Maximum length of lesion		
Maximum diameter of lesion at 90º to long axis of lesion		

Data Required to Calculate Breast Volume (in mm)

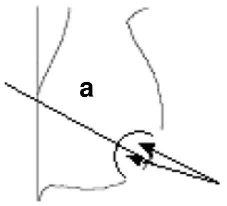

a = ☐

Angles should be equal

Chest wall to nipple (in the nipple plane)

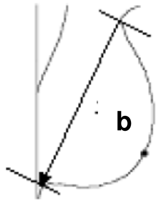

b = ☐

Infra mammary fold to supra mammary (axillary) fold

Magnetic Resonance Imaging (MRI)

Was a pre-operative MRI performed? **Yes** ☐ **No** ☐

Estimated maximum dimension of lesion (mm) on MRI ☐

Specimen X-Ray

Was a specimen X-ray performed? **Yes** ☐ **No** ☐

If "Yes":

Was the specimen X-ray diagnostic or therapeutic? **Diagnostic** ☐ **Therapeutic** ☐

Is the lesion present in the specimen X-ray? **Yes** ☐ **No** ☐

Does the lesion appear radiologically to have been completely excised? **Yes** ☐ **No** ☐

Minimum Distance from Edge of Lesion to Edge of Specimen (in mm) ☐

Date (DD/MM/YY) ☐ **Radiologist (Print name)** ☐

Version 2: Issued 01/08/2009

Sloane Project: Treatment Data Form

Name ☐ **Screening No.** ☐

Date of Birth ☐ **Screening Unit** ☐

Hospital ☐ **Hospital No.** ☐

NHS No. ☐

Side: **Left** ☐ **Right** ☐ ***For bilateral disease, please use separate form for each breast.***

Surgical Procedure(s)
Total number of operations performed
(if more than 2 operations please use separate form for operation 3 onwards) ☐

Was there a pre-operative diagnosis of DCIS? **Yes** ☐ **No** ☐

Operation 1
Date (dd/mm/yy) ☐

Grade of surgeon performing operation ☐

Operation 2
Date (dd/mm/yy) ☐

Grade of surgeon performing operation ☐

Breast Procedures
Please indicate procedures undertaken at Operation 1 & Operation 2.
(Tick one box only for each operation. If more than 2 operations please use separate form)

	**Op 1**	**Op 2**
Diagnostic Biopsy	☐	☐
Wide Local Excision including excision of nipple	☐	☐
Wide Local Excision preserving nipple	☐	☐
Further excision to clear margins	☐	☐

	**Op 1**	**Op 2**
Simple mastectomy	☐	☐
Subcutaneous mastectomy	☐	☐
Skin sparing mastectomy	☐	☐
Therapeutic mammoplasty with nipple preservation	☐	☐
Therapeutic mammoplasty without nipple preservation	☐	☐

Immediate Breast Reconstruction
(If more than 2 operations please use separate form)

	**Op 1**	**Op 2**	***Plus Implants (please tick)***
Latissimus Dorsi flap	☐	☐	☐
D.I.E.P. flap	☐	☐	☐
T.R.A.M. flap	☐	☐	☐

	**Op 1**	**Op 2**
Wide Local Excision + mini latissimus dorsi flap (Rainsbury's procedure)	☐	☐
Implant only	☐	☐
Other autologous tissue technique	☐	☐

Sloane Project Number (For Official Use Only) ☐

QA Ref Number (For Official Use Only) ☐

Version 2: Issued 01/08/2009

Axillary Procedures

	Op 1 Yes	Op 1 No	Op 2 Yes	Op 2 No
Was a decision(s) made pre-operatively re axillary surgery at the MDM?	☐	☐	☐	☐

Were axillary nodes taken? **Yes** ☐ **No** ☐

If "Yes" which of the following procedures were carried out.
(Tick all that apply. If more than 2 operations performed please use separate form.)

	Op 1	Op 2
Sentinel node Procedure	☐	☐
Axillary node sample	☐	☐
Axillary node clearance	☐	☐

Further Surgical Details
For women treated with conservation surgery:

	Op 1 Yes	Op 1 No	Op 2 Yes	Op 2 No
Was the margin nearest to the nipple marked?	☐	☐	☐	☐
Was the specimen orientated?	☐	☐	☐	☐

For ALL operation types:

	Op 1 Yes	Op 1 No	Op 2 Yes	Op 2 No
Was a further excision performed at the same operation?	☐	☐	☐	☐
Was the immediate re-excision orientated?	☐	☐	☐	☐
Were cavity shavings (bed biopsies) taken?	☐	☐	☐	☐
Was a further excision carried out on the basis of the Specimen X-ray at the time?	☐	☐	☐	☐

Treatment Strategy – Following the Final Operation

Was the status of the margins discussed at the MDM? **Yes** ☐ **No** ☐

Does the final margin status fulfil local protocol for adequacy of resection margins? **Yes** ☐ **No** ☐ **No Protocol** ☐

If "No" to above question, please state reasons why no further surgical treatment was undertaken if margins were inadequate. ☐

Did the post-op MDM decision on adjuvant therapy include (Tick all that apply):

Referral for Radiotherapy ☐ No further adjuvant therapy ☐ Other therapy ☐

Hormone therapy ☐ Date started Hormone Therapy ☐

Type of Hormone Therapy Tamoxifen ☐ Aromatase Inhibitor ☐ Other ☐

Clinical Trials
Patient entered Clinical Trial **Yes** ☐ **No** ☐ **Name of trial entered** ☐

Date (DD/MM/YY) ☐ **Surgeon (Print name)** ☐

Version 2: Issued 01/08/2009

Sloane Project: Pathology Data Form

Please complete ONE form for an EPISODE.

For bilateral disease, please use separate form for each breast.

**Surname**		**Forename(s)**	
**Date of Birth**		**Screening Unit**	
**Hospital**		**Hospital No.**	
**Histology Report No.**		**NHS No.**	

Side: **Left** ☐ **Right** ☐

Specimen Type (Tick ALL that apply, and weight, if known)

	Y/N	Disease present in core: DCIS	ADH	LCIS
Core biopsy (standard 14 gauge)				
8 or 11 gauge core (e.g. mammotomy)				

DCIS core grade: High ☐ Intermediate ☐ Low ☐

Calcification Present: None ☐ Benign ☐ Malignant ☐ Benign & Malignant ☐

**Surgical Specimens:**	**Y/N**	**Weight (g)**		**Y/N**	**Weight (g)**
Diagnostic Open Biopsy			Therapeutic Excision (WLE)		
Bed Biopsies/ Cavity Shaves			Delayed re-excision (i.e. at a later operation)		
			Completion Mastectomy		
Immediate re-excision (i.e. at time of WLE)			Mastectomy		

**For therapeutic excision / WLE:**	**Yes**	**No**	**Uninterpretable**
Nipple orientation marker present?			
Other orientation markers (sutures/clips)?			

Excision Histology – Lesion (Tick all present): **DCIS** ☐ **ADH** ☐ **LISN** ☐

For DCIS only
Maximum (total) size of DCIS (mm) (summation from all specimens) ☐

Please tick if the total is size from one specimen only ☐

If summation from several specimens, please give size in each (mm) ☐ **+** ☐ **+** ☐

Sloane Project Number (For Official Use Only) ☐

QA Ref Number (For Official Use Only) ☐

Version 2: Issued 01/08/2009

Highest Nuclear Grade (Please tick one only) High ☐ Intermediate ☐ Low ☐

Growth Pattern (Tick all that apply)

Solid ☐ Cribriform ☐ Micropapillary ☐ Papillary ☐

Apocrine ☐ Flat/clinging ☐ Other type, please state…….…………………

	**Yes**	**No**		**Yes**	**No**	**N/A**
Comedo necrosis present?	☐	☐	Paget's disease?	☐	☐	☐

	**Yes**	**No**		
Microinvasion (<1mm)?	☐	☐	No. foci microinvasion?	☐

Distance of DCIS to margins (mm) **(Please complete all available and indicate if >10mm)**

	Medial	Lateral	Superior	Inferior	Superficial	Deep	Nipple
**Therapeutic Excision**	mm	mm	mm	mm	mm	mm	mm

Please supply margin data above for a therapeutic excision, even if followed by mastectomy.

**If altered by further surgery, final margin distance**	mm	mm	mm	mm	mm	mm	mm

Mastectomy
(please tick if this applies) ☐

Does another process extend to the margin?
(Please tick all that apply) **ADH** ☐ **LISN** ☐

Immediate re-excision / Cavity Shaves / Bed Biopsies

No. samples received ☐

	**DCIS**	**ADH**	**LISN**
Did these samples contain (Tick all that apply)	☐	☐	☐

**Nodes**	**Axillary Nodes**	**Sentinel Node**	**Others**
Number examined overall	☐	☐	☐
Number positive overall	☐	☐	☐

**Receptor Status**	**Positive**	**Negative**	**Not Known**	**Cut off used**
ER Status	☐	☐	☐	
PGR Status	☐	☐	☐	
HER-2 Status	☐	☐	☐	

Date (DD/MM/YY) ☐ **Pathologist (Print name)** ☐

Version 2: Issued 01/08/2009

Sloane Project: Radiotherapy Data Form

Surname ☐ **Forename(s)** ☐

Date of Birth ☐ **Screening Unit** ☐

Hospital ☐ **Hospital No.** ☐

NHS No. ☐

Side: Left ☐ Right ☐ ***For bilateral disease, please use separate form for each breast.***

Method of treatment
Radiotherapy - External Beam **Yes** ☐ **No** ☐ ***If "No" why?*** ☐

If "Yes" please complete the following:-

Dose(Gy) ☐ Energy (MV) ☐ Number of fractions ☐

Treated daily? **Yes** ☐ **No** ☐ *If "**No**" please state frequency* ☐

Date treatment commenced (dd/mm/yy) ☐ Date treatment finished (dd/mm/yy) ☐

Axilla treated? **Yes** ☐ **No** ☐ Boost given? **Yes** ☐ **No** ☐

If Boost given, please complete the following:-

Electrons (Energy – MeV) ☐ Megavoltage (Energy – MV) ☐ Orthovoltage (Energy – kV) ☐ Dose (Gy) ☐ Number of fractions ☐

Treated daily? **Yes** ☐ **No** ☐

Date treatment commenced (dd/mm/yy) ☐ Date treatment finished (dd/mm/yy) ☐

Brachytherapy – Interstitial

Yes ☐ **No** ☐ *If "**Yes**" please complete the following:-* Dose(Gy) ☐

Date (DD/MM/YY)) ☐ **Oncologist (Print name)** ☐

Sloane Project Number (For Official Use Only) ☐ **QA Ref Number (For Official Use Only)** ☐

Version 2: Issued 01/08/2009

SLOANE PROJECT FOLLOW UP FORM
(FOR RECURRENCES)

The purpose of this detailed follow up form is to accumulate information on ipsilateral recurrences, contralateral breast disease and metastases. The form is split up into four sections:

1) **Demographic details (these will already be completed if all information known)**
2) **Type of recurrence detected and route of presentation**
3) **Diagnosis, treatment and pathology of recurrence/contralateral disease**
   (a) **Ipsilateral recurrence**
   (b) **Contralateral breast disease**
4) **Diagnosis and treatment of distant metastases**

Please complete all sections that apply to this patient.

SECTION 1 Demographic Details

Screening Unit ☐ **Date of Birth** ☐

NHS Number ☐ **Screening Number** ☐

Hospital Number ☐

SECTION 2

Diagnosis of recurrence / contralateral disease / metastases

Type of Recurrence Detected and Date of Diagnosis (please tick all that apply)

Local-regional ☐ Distant ☐ Contralateral disease ☐

Date ☐ Date ☐ Date ☐

Route of Presentation (tick all that apply)

Detected on FU mammogram ☐ Clinical Exam at routine FU ☐

Following GP referral to OPD clinic ☐ Other (please give details) ______________________

Version 2: Issued 01/08/2009

SECTION 3 (a) Local-regional recurrence

The purpose of this section is to accumulate information on the IPSILATERAL RECURRENCE – when it occurred, where it occurred in the breast, how it presented and how it was treated.

Diagnosis of local-regional recurrence

**Site of local-regional recurrence(s) (Tick all that apply)**		**Procedures used to confirm recurrence (Tick all that apply)**					
		Mammogram	FNA	Core Biopsy	Excision biopsy	MRI	Other (please specify)
Breast (if conserved) – at or adjacent to site of original primary							
Breast (if conserved) – second neoplasm some distance from site of original							
Nipple							
Mastectomy scar/flaps							
Ipsilateral axilla							
Ipsilateral supraclavicular fossa							
Reconstructed breast mound							
Other (Please specify)							

Treatment of local-regional recurrence

Treatment of local-regional recurrence (Tick all that apply)

Surgical procedures ☐ Radiotherapy ☐ Hormone Therapy ☐ Chemotherapy ☐

Surgical procedures (tick all that apply)

Further Wide Local Excision ☐ Mastectomy ☐ Axillary node surgery ☐ Other__________________ (Please give details)

Radiotherapy to recurrence (Please tick all sites that were treated)

Breast ☐ Axilla ☐ Chest wall ☐ Supraclavicular fossa ☐ Interstitial ☐

Hormone Therapy (please tick if given for recurrence)

Tamoxifen ☐ Aromatase Inhibitor ☐ Other (Please state) __________________

Chemotherapy (please give details of the regime)

CMF alone ☐ Herceptin ☐ Anthracycline containing regime (e.g. FEC, Epi-CMF) ☐

Taxane containing regime (e.g. Taxol, Taxotere) ☐ Other regime (Please give details) __________________

Version 2: Issued 01/08/2009

SECTION 3 (a) – continued
Pathology of local-regional recurrence

Type and Grade of recurrence (Please tick all that apply)

Invasive ☐ Non-Invasive (DCIS) ☐ Non-invasive (LCIS/ALH) ☐

Invasive grade Grade 1 ☐ Grade 2 ☐ Grade 3 ☐

DCIS Grade Low ☐ Intermediate ☐ High ☐

Size of local-regional recurrence

DCIS (mm) ☐ Invasive size (mm) ☐ Whole tumour (DCIS + invasive) size (mm) ☐

DCIS growth pattern(s) (Tick all that apply)

Solid ☐ Cribriform ☐ Micropapillary ☐ Papillary ☐

Apocrine ☐ Flat ☐ Other (please specify) ☐

Microinvasion Present ☐ Not Present ☐

Histological Type of Invasive Tumour

No special type (ductal NST) ☐ Pure special type (90% purity, specify components present below) ☐ Mixed tumour type (50-90% special type component, specify components present below) ☐

Component (s) present for pure special type and mixed tumour types:

Tubular/cribriform ☐ Lobular ☐ Mucinous ☐ Medullary like ☐

Ductal / no special type ☐ Other (please specify) ☐

Nodes

Number examined overall ☐ Number positive ☐

Vascular invasion Present ☐ Possible ☐ Absent ☐ Not known ☐

Receptor status

	**Positive**	**Negative**	**Not known**	**Cut off for positivity used**	**Invasive**	**DCIS (indicate)**
ER status	☐	☐	☐	☐	☐	☐
PgR status	☐	☐	☐	☐	☐	☐
HER-2 status	☐	☐	☐	☐	☐	☐

Version 2: Issued 01/08/2009

SECTION 3 (b) Contralateral disease

We need to quantify the risk to the opposite breast, which is why we need to know whether the cancer is invasive or non-invasive and how it was treated.

Site of contralateral disease (Tick all that apply)		Procedures used to confirm contralateral disease (Tick all that apply)					
		Mammogram	FNA	Core Biopsy	Excision biopsy	MRI	Other (please specify)
Contralateral Breast							
Contralateral Nipple							
Contralateral axilla							
Other (Please specify)							

Treatment of contralateral disease

Treatment of contralateral disease (Tick all that apply)

Surgical procedures ☐ Radiotherapy ☐ Hormone Therapy ☐ Chemotherapy ☐

Surgical procedures (tick all that apply)

Wide Local Excision ☐ Mastectomy ☐ Axillary node surgery ☐ Other ________________ (Please give details)

Radiotherapy for contralateral disease (Please tick all sites that were treated)

Breast ☐ Axilla ☐ Chest wall ☐

Supraclavicular fossa ☐ Interstitial ☐

Hormone Therapy (please tick if given after diagnosis of contralateral disease)

Tamoxifen ☐ Aromatase Inhibitor ☐ Other (Please state) ________________

Chemotherapy (please give details of the regime)

CMF alone ☐ Herceptin ☐ Anthracycline containing regime (e.g. FEC, Epi-CMF) ☐

Taxane containing Regime (e.g. Taxol, Taxotere) ☐ Other regime (Please give details) ________________

Other treatment given (please specify) ________________

Version 2: Issued 01/08/2009

SECTION 3 (b) – continued
Pathology of contralateral disease

Type and Grade of recurrence (Please tick all that apply)

Invasive ☐ Non-Invasive (DCIS) ☐ Non-invasive (LCIS/ALH) ☐

Invasive grade Grade 1 ☐ Grade 2 ☐ Grade 3 ☐

DCIS Grade Low ☐ Intermediate ☐ High ☐

Size of Contralateral recurrence

DCIS (mm) ☐ Invasive size (mm) ☐ Whole tumour (DCIS + invasive) size (mm) ☐

DCIS growth pattern(s) (Tick all that apply)

Solid ☐ Cribriform ☐ Micropapillary ☐ Papillary ☐

Apocrine ☐ Flat ☐ Other (please specify) ☐

Microinvasion Present ☐ Not Present ☐

Histological Type of Invasive Tumour

No special type (ductal NST) ☐ Pure special type (90% purity, specify components present below) ☐ Mixed tumour type (50-90% special type component, specify components present below) ☐

Component (s) present for pure special type and mixed tumour types:

Tubular/cribriform ☐ Lobular ☐ Mucinous ☐ Medullary like ☐

Ductal / no special type ☐ Other (please specify) ☐

Nodes

Number examined overall ☐ Number positive ☐

Vascular invasion Present ☐ Possible ☐ Absent ☐

Receptor status

	Positive	Negative	Not known	Cut off for positivity used	Invasive	DCIS (indicate)
ER status	☐	☐	☐	☐	☐	☐
PgR status	☐	☐	☐	☐	☐	☐
HER-2 status	☐	☐	☐	☐	☐	☐

Version 2: Issued 01/08/2009

SECTION 4 Distant metastases

We would like information on whether or not the patient has confirmed distant metastases.

Site of distant metastases (Tick all that apply)

Bone ☐ Lung ☐ Liver ☐ Brain ☐ Other (please specify) ____________________

Treatment (Tick all that apply)

Surgical Procedures ☐ If ticked, please indicate type of procedure ____________________

Radiotherapy ☐ If ticked, please give details of site ____________________

Hormone Therapy ☐ If ticked, please indicate type of hormone therapy ____________________

Bisphosphonates ☐

Chemotherapy ☐ If ticked, please give details of type of regime ____________________

Other ☐ If ticked, please give details ____________________

Is there any evidence of any invasive focus to account for the metastases?

Yes ☐ No ☐ N/K ☐ Please comment if you wish ____________________

____

Thank you for your co-operation.

Version 2: Issued 01/08/2009
