# Supplementary material for: Cohort profile of the Sloane Project: methodology for a prospective UK cohort study of >15 000 women with screen-detected non-invasive breast neoplasia
Source: BMJ Open. 2022 Dec 19;12(12):e061585. doi: 10.1136/bmjopen-2022-061585 (PMC9764674; doi:10.1136/bmjopen-2022-061585)
Supplement: Supplementary data [file bmjopen-2022-061585supp002.pdf]

The Sloane Project
Understanding non-invasive breast disease

Sloane Project atypia audit form

For Official Use Only

Sloane ID

Patient ID

- Complete form for each Sloane Project patient
- Submit data on patients diagnosed with lobular neoplasia (LN), lobular carcinoma in situ (LCIS), pleomorphic lobular carcinoma in situ (PLCIS), atypical intraductal epithelial proliferation (AIDEP), atypical ductal hyperplasia (ADH), atypical lobular hyperplasia (ALH), flat epithelial atypia (FEA)
- Submit data from patients who screen-detected by the NHS Breast Screening Programme on or after 1 April 2012.
- Concurrent or previous Ductal Carcinoma In Situ (DCIS) or Invasive Breast Cancer are not eligible.
- Please return all forms securely using only nhs.net mail to **PHE.Sloaneproject@nhs.net** or to **Sloane Project team, Screening QA Service, NHS England & Improvement, 2nd Floor, 23 Stephenson Street, Birmingham B2 4BH**
- If you need help completing any part of this form, contact the team on PHE.Sloaneproject@nhs.net

**Patient date of birth**		**Patient NHS number**	
**Screening unit**		**Screening number (SX)**	
**Date of screening mammogram**		**Screening round**	
**Histology report number**		**Hospital and hospital number**	

Side: left ☐ right ☐ for bilateral disease, please use separate form for each breast

Has patient had previous breast atypia? Y/N ☐ **If yes, year of diagnosis** ☐

Background pattern (Tick ONE only)

Fatty ☐ Scattered fibroglandular density ☐ Hetrogeneously dense ☐ Extremely dense ☐

Predominant radiological feature (Tick ONE only)

Calcification ☐ Parenchymal distortion ☐ Mass: well-defined ☐ Spiculate mass ☐ Mass: ill-defined ☐ None of these ☐

Does the lesion contain microcalcification? Y/N ☐

If yes, what is the most suspicious pattern? (Tick ONE only)

Cast/linear/casting ☐ Granular/irregular/crushed stone ☐ Punctate/powderish ☐

Maximum estimated size of lesion (in millimetres) on mammogram ☐

**Core biopsy** (tick all that apply)	Y/N	No. of cores	Gauge	AIDEP	LN	PLCIS	FEA	Benign
Standard core biopsy (14 gauge)	☐	☐		☐	☐	☐	☐	
Diagnostic vacuum assisted biopsy (for example 8 or 11 gauge core)	☐	☐	☐	☐	☐	☐	☐	☐

	Y/N	No. of cores	Gauge	ADH	ALH	LCIS	PLCIS	FEA	Benign
Therapeutic vacuum assisted excision (for example 8 or 11 gauge core)	☐	☐	☐	☐	☐	☐	☐	☐	☐

Is calcification present in core? Y/N ☐ If yes, indicate site (tick ONE only) Atypical lesion ☐ Benign lesion ☐ Atypia and benign ☐ Unknown site ☐

Other benign lesion present? Y/N ☐ If yes, specify which (tick ONE only)

Fibroadenoma ☐ Papilloma ☐ Radial scar ☐ Other ☐ If other, please specify: ☐

**Surgical Specimens**	Y/N	DATE		Y/N	DATE
Diagnostic open biopsy			Delayed re-excision (for example at a later operation)		
Therapeutic excision (WLE)			Completion mastectomy		
Bed biopsies/ cavity shaves			Mastectomy		
Immediate re-excision (for example at time of WLE)			No surgical specimen		

Disease present in surgical specimen (tick all that apply)

ADH ☐ ALH ☐ LCIS ☐ PLCIS ☐ FEA ☐ No additional residual disease ☐ Maximum (total) size of lesion ☐

Does any disease extend to the margin of the surgical specimen or re-excision specimen?(tick all that apply)

ADH ☐ ALH ☐ LCIS ☐ PLCIS ☐ FEA ☐

Is calcification present in surgical specimen? Y/N ☐ If yes, indicate site (tick ONE only) Atypical lesion ☐ Benign lesion ☐ Atypia and benign ☐ Unknown site ☐

Is comedo necrosis present? Y/N ☐

Other benign lesion present? Y/N ☐ If yes, specify which (tick ONE only)

Fibroadenoma ☐ Papilloma ☐ Radial scar ☐ Other ☐ If other, please specify: ☐

**Nodes**	**Axillary nodes**	**Sentinel nodes**	**Others**
Number examined overall			
Number positive overall			

At which operation were nodes taken? (e.g. 1st, 2nd) ☐

Treatment strategy – following the final operation
Did the post-op MDM decision on adjuvant therapy include (tick all that apply):

Referral for radiotherapy ☐ Date started radiotherapy ☐

No adjuvant therapy ☐ Other therapy ☐ Endocrine therapy ☐

Date started endocrine therapy ☐ Endocrine therapy type (tick all that apply) Tamoxifen ☐ Aromatase inhibitor ☐ Other ☐

Date (DD/MM/YY) ☐ **Print name and email address** ☐
