# Supplementary material for: Cohort profile of the Sloane Project: methodology for a prospective UK cohort study of >15 000 women with screen-detected non-invasive breast neoplasia
Source: BMJ Open. 2022 Dec 19;12(12):e061585. doi: 10.1136/bmjopen-2022-061585 (PMC9764674; doi:10.1136/bmjopen-2022-061585)
Supplement: Supplementary data [file bmjopen-2022-061585supp003.pdf]

SLOANE PROJECT ~

GUIDANCE FOR COMPLETION OF RADIOLOGY DATA FORM

Demographic Details

The demographic details include patient identification, date of screening mammogram and whether this is prevalent or incident screening.

Side

A separate form is required for each side in the cases of bilateral disease. For multi-focal disease in a single site, only one form should be completed.

Site

For multi-focal disease, it is important that there is concordance of the site which is reported on the pathology form with that which is measured on the radiology form. As these cases are being identified at the postoperative MDM, correlation should take place at this time.

Background Pattern

During the pilot phase of the radiology data collection a number of classifications were used to describe the background parenchymal pattern. Recognising that this is a highly subjective classification, the pilot centres agreed that the birads classification was the most reproducible.

Predominant Radiological Feature

For ease of analysis, please choose the predominant radiological feature on the mammogram and classify as appropriate.

Calcification

With regard to the classification of calcification, two systems of classification have been included ~ please choose one system only and examples of the three groups are enclosed.

Estimated Size of Lesion on Mammograms

All measurements have to be done both on the oblique view and the CC views. Three reproducible measurements have been chosen:

a. Distance from base of nipple to nearest part of the lesion i.e. border of mass/first dot of calcium, closest area of parenchymal distortion (see accompanying diagram)
b. Maximum length of lesion ~ see diagram
c. Maximum diameter of lesion at 90^0 to long axis of lesion ~ see diagram

Please note that as cases of distortion are few and far between and because of difficulties in relation to measuring these cases, it has been agreed that measurements of distortions are no longer required. This includes measuring distance from nipple.

In cases where there are two distinct areas separated by apparently normal breast tissue, then the measurement should be from one end of one site to the further most end of the other site, along with all the apparently normal breast tissue in between. The reason for this is that it reflects how we clinically manage such cases (i.e. if DCIS is found in two sites with apparently normal breast tissue in between the patient will generally be subjected to mastectomy).

Data required to Calculate Breast Volume

The accompanying formula (1/3 pi r^2h) has been validated and is thought to be the closest approximation to breast volume that can be carried out from 2 view mammography. Please be aware that on the original form the labels for the measurements were “h” and “2r”. The labels have now been amended to “a” and “b” respectively (though the same two measurements are still required) as it was felt that the previous labels were causing some confusion.

Please note that even though the size of the lesion and distance from the nipple are not to be measured in cases of distortion, the data to calculate breast volume is still required.

Specimen X-Ray

Please identify by tick whether the specimen is as a result of a diagnostic or therapeutic biopsy.

With the increase in the use of mammotomy to diagnose these cases pre-operatively, there is the possibility that in some cases all the calcifications will have been removed and that a clip will be left in situ. For the purposes of completion of this section, it is possible to say that the entire lesion has been removed, but it is inappropriate to make measurements as to proximity to the margin etc.

Excision

With regard to completeness of excision, arbitrarily 2mm (unmagnified) of “normal” breast tissue has to be present between the margins which are closest to the mammographic abnormality.

SLOANE PROJECT
Guidelines for Pathological Assessment of DCIS
Pathology Protocol

Introduction

It is presumed that the vast majority of cases of DCIS entered into the DCIS trial will be identified as screen-detected microcalcification and in general the existing NHSBSP Pathology Reporting Guidelines (NHSBSP Publications. Sheffield. 1995), or updated guidelines, should be adhered to. Reporting pathologists should take part in the National Breast Screening Pathology EQA scheme. Unless pre-operative diagnosis of DCIS has been successful, these may therefore be a diagnostic needle localisation biopsy, prior to a therapeutic procedure. Both the surgical and histopathological technique for handling of these specimens differs significantly. Some general guidelines for specimen handling, both in the operating theatre and in the laboratory can be described. For example, frozen section examination is inappropriate in cases of screen-detected microcalcification. Whatever technique is used, the methodology should enable production of the minimum dataset information.

Surgical Handling

- It is anticipated that lesions will be resected according to the defined protocol. If their surgical resection differs from the protocol, for example dissection does not extend to the deep fascia or skin, this should be clearly indicated on the request form.
- The surgeon should orientate all specimens. Each unit should establish a code of orientation using either different lengths of suture or metal staples/clips or ink, which are anatomically relevant and assist in accurate evaluation of the specimen. The nipple extension / direction of the nipple should be separately marked.
- If more than one piece of tissue is removed, it should be made clear how the samples are orientated with respect to each other in order to simplify assessment of size of the lesion and distance to margins.
- After surgical excision the specimen should be X-rayed. The X-ray should be reported by the breast radiologist. However the X-rays must be available to the pathologist so that they can determine a) whether the microcalcification has been excised and b) its site and extent within the sample.
- The specimen should be sent immediately to the pathology laboratory – ideally in the fresh state. If this is not possible it should be immediately placed in an appropriate quantity of fixative.

General Laboratory Handling

- Once received in the laboratory the entire surface of the specimen should be inked so that the margins of excision can be easily determined. This can be performed by prior removal of surface lipid by dipping the specimen in alcohol and drying and then applying an appropriate pigment such as India Ink, Alcian blue, dyed gelatine or a multiple ink technique.
- Good fixation is vital to preserve the morphological detail. This is particularly relevant for the diagnosis of these sometimes difficult intraductal epithelial proliferations. They must be placed in sufficient formalin or other appropriate fixative either before, or preferably after, receipt by the laboratory.

Diagnostic Biopsies

- The specimen should be weighed and measured and then serially sliced at intervals of approximately 3mm.
- Cases where block selection is required (i.e. not embedded in their entirety) will benefit from specimen slice examination, particularly those where microcalcification is the principle feature by which the DCIS was detected. It enables blocks to be taken from the areas corresponding to the mammographic abnormality, as well as any other suspicious areas identified.
- The sites of sampling can be marked on the specimen X-ray or X-ray of slices, by using a white wax (chinograph) pencil or other marker.
- The sampling technique and the number of blocks taken are clearly dependent on the size of the specimen and the size of the abnormality. If the specimen is small, it is often best to block and examine all of the tissue. Samples approximately 30 mm or less in maximum dimension should be completely sliced, embedded and examined histologically.
- For larger specimens sampling should be adequate to determine accurately the size of the DCIS and exclude, within the boundaries of practicality, associated invasion. Sampling should include the extremes of the mammographic abnormality and adjacent tissue, in order to avoid underestimation of size of DCIS. This is particularly important as it is recognised that mammographic size may be an underestimate of true tumour size.

Diagnostic Biopsies (continued)

- The margins of diagnostic specimens should also be identified. The nearest margin to the mammographic abnormality should be included in sections. It should be noted, however, that the aim of these procedures is not complete excision and the histopathological assessment of margin status is not the primary requisite of the examination.
- Accuracy of size measurement of DCIS is vital and measurements using the Vernier scale of the stage micrometer or an eyepiece graticule are the ideal ways to ascertain this measurement. If the DCIS extends over several blocks the measurement may be partly deduced by summating the thickness of the blocks (if they are parallel samples) or from measurements deduced by mapping the involvement on the specimen.
- Large blocks and sections may be extremely helpful, especially for determining the size of DCIS, and are particularly helpful when the DCIS area is large. Adequate fixation may, however, on occasions be more difficult to achieve and large sections are also more difficult to cut and to store and the longer processing necessary may introduce delays. Their use depends on local preference.
- If diagnostic samples are sent as more than one portion, it can be extremely difficult to measure the absolute extent of DCIS present. In these cases it is appropriate to take a pragmatic approach and measure the maximum distance in any piece of tissue and to add the dimensions to give an approximate total size. If, however, the orientation of the specimens can be determined, the size of DCIS can be ascertained more reliably.

Therapeutic Excisions

- The specimen should be weighed and measured in three dimensions.
- The technique for sampling the abnormality will vary somewhat according to type of sample, specimen size and also with pathologist/laboratory preference. Several options are available. Whichever is utilised, as an absolute minimum, the information for the minimum dataset, including accurate measurement of size and detailed examination of the margin status and distance to margins must be provided. Three suggestions for handling these samples are described below. It should be noted that these are schematic diagrams and that frequently the specimen is more irregular in outline than demonstrated.

The pathology data record should indicate which method has been used. If a local examination policy is used which differs from these methods this must be indicated and the protocol provided to the co-ordination centre.

METHOD 1 – Serial slicing perpendicular to the medial – lateral plane. Figure 1.

Figure 1

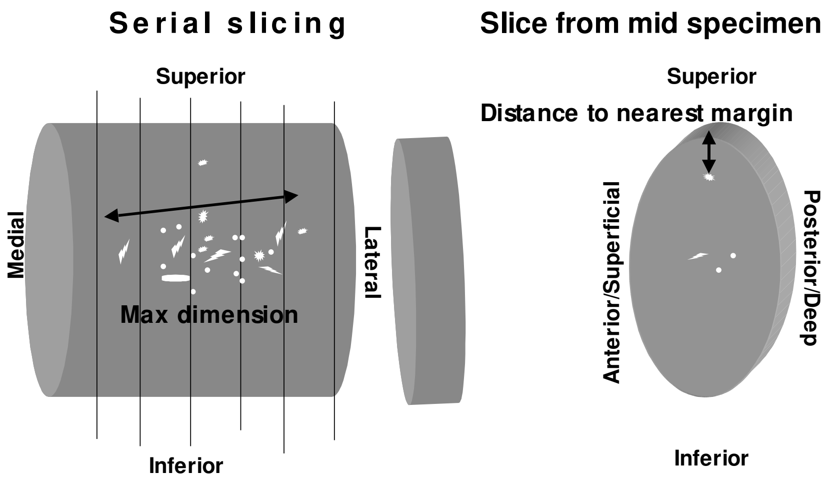

- The majority of these samples taken for mammographic calcification will require serial slicing at intervals of approximately 3mm.
- These specimens may benefit from specimen slice examination, but this may not be absolutely essential for all samples e.g. mass lesions. Where microcalcification is the principle feature by which the DCIS was detected, serially slicing and re-X-raying of the specimen slices will enable blocks to be taken most accurately from the areas corresponding to the mammographic abnormality, as well as any other suspicious areas identified. The sites of sampling can be marked on the specimen for radiological-pathological discussion in difficult cases.
- The number of blocks taken will depend on the size of the specimen and of the size of the abnormality. If the specimen is small, it is often best to block and examine all of the tissue. Samples 30mm or less in maximum dimension should be completely sliced, embedded and examined histologically.
- For larger specimens adequate sampling should be undertaken, to determine accurately the size of the DCIS and to exclude coexisting invasion. Sampling should include the extremes of the mammographic abnormality and adjacent tissue, in order to avoid underestimation of size of DCIS. This is particularly important as it is recognised that mammographic size may be an underestimate of true tumour size.

- If therapeutic samples are sent in more than one portion, it can be extremely difficult to measure the absolute largest extent of DCIS present. In these cases it is appropriate to take a pragmatic approach and measure the maximum distance in any piece of tissue and to add the dimensions to give an approximate total size. If, however, the orientation of the specimens can be determined, the size of DCIS can be ascertained more reliably.
- The margins of therapeutic excision specimens should also be extensively sampled. The nearest margin to the mammographic abnormality must be blocked, as an absolute minimum, in order to facilitate measurement of this distance. Accuracy is vital and measurement using the Vernier scale of the stage micrometer or an eyepiece graticule is the ideal way to ascertain this measurement. In larger lesions dot marking and mapping the lesion extent on the slide coverslip may be helpful.
- If the DCIS extends over several blocks the measurement of distance to margins may be partly deduced by summating the thickness of the parallel slices and from measurements deduced by mapping the involvement on the specimen.
- Large blocks and sections may be extremely helpful, especially for determining the size and the margin status of DCIS, particularly when the DCIS area is large. Adequate fixation may, however, on occasions be more difficult to achieve and large sections are also more difficult to cut and to store and the longer processing necessary may introduce delays. Their use depends on local preference.
- The use of different colour inks/markers on an individual section allows microscopic identification of specific margins.

METHOD 2 - Serial slicing perpendicular to the superficial – deep plane. Figure 2.

Figure 2

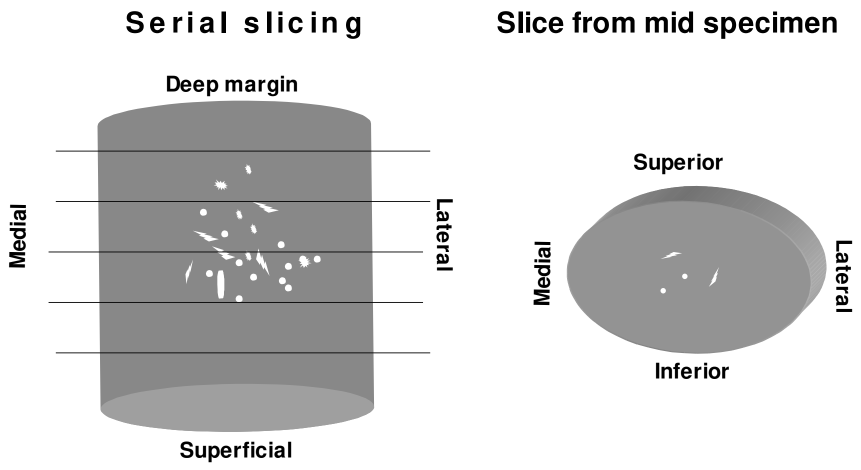

This is a variation of method 1, which is particularly suitable for smaller specimens, making use of large block techniques. The entire specimen can be examined as a small number of serial large sections and the technique is similar to the method used to examine radical prostatectomy specimens in many centres.

METHOD 3 - Radial block and shave margin examination. Figure 3.

Figure 3

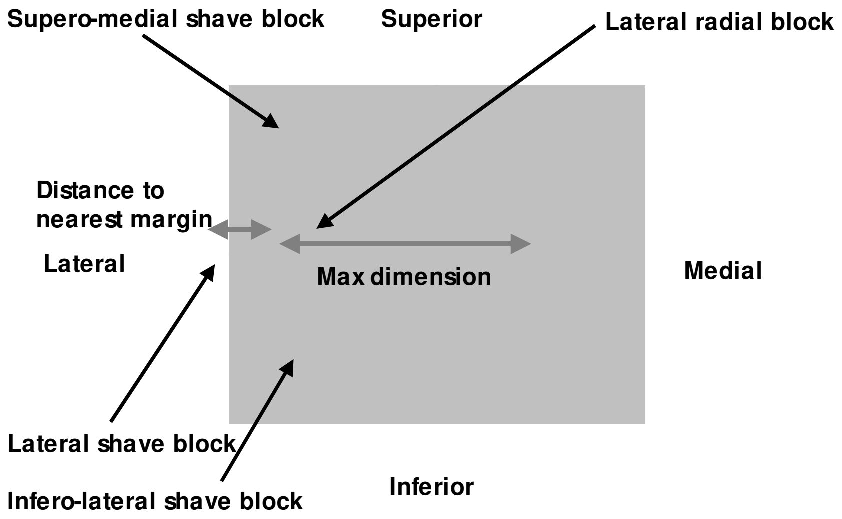

On occasions, particularly if a macroscopic abnormality is identified, it may be appropriate to sample the specimen with multiple blocks of the lesion, taken at right angles, as described below.

Margins

- Sections taken for measurement of distance to margins will include a slice through the lesion to the radial edges of the specimen and allow measurement of the distance using the stage micrometer.
- Blocks of the "*radial*" margins, lateral, medial, superior and inferior to the lesion should be sampled before taking "*shaves*" of the remaining circumferential aspect.
- If appropriate (i.e. nearest on the specimen) one or more of the following: supero-lateral, supero-medial, infero-medial, infero-lateral may be taken.
- It may be possible to take radial margin and the lesion in one section for smaller resections. For larger specimens, blocks may be of sufficient size to require blocking in 2 (or more) cassettes.
- The circumferential edge of the sample should be shaved, unless the surgeon provides cavity shaves. This may produce, for example: superior shave, supero-lateral shave, lateral shave, infero-lateral shave, inferior shave, infero-medial shave, medial shave and supero-medial shaved edges depending on the size of the specimen.

Margins (continued)

- It should be noted that shaved edges of the margins of the specimen of examination of “cavity shaves/bed biopsies” confirm completeness of excision but do not allow measurement of distance.
- **Cavity shave / biopsy specimens.** Centres may wish to use separately submitted surgical cavity shaving / biopsies as an adjunct to pathological examination of the margins of the main specimen. These specimens should be clearly marked and examined separately.

Sampling the lesion

- Blocks of the mammographic abnormality/lesion should be taken at right angles and allow as accurate as possible a determination of maximum extent of the DCIS.
- Additional blocks of the lesion should be sampled. In the majority of cases of DCIS treated by local surgical excision (i.e. not large lesions) it is appropriate to block the majority of the lesion/ area of calcification on specimen.
- In particular, the maximum extent of the calcification and the adjacent tissue should be taken to be certain that non-calcified DCIS does not extend beyond the radiological abnormality.
- If therapeutic samples are sent in more than one fragment, it can be extremely difficult to measure the absolute largest extent of DCIS present. In these cases it is appropriate to take a pragmatic approach and measure the maximum distance in any piece of tissue and to add the dimensions to give an approximate total size. If, however, the orientation of the specimens can be determined, the size of DCIS can be ascertained more reliably.

Re-excision Specimens

- If the radiological abnormality extends close to a margin on the specimen radiograph, the surgeon may undertake an immediate re-excision of that particular margin.
- A separate re-excision specimen may therefore be taken (a) at the time of initial surgery or (b) subsequent to the discovery of incomplete excision in a therapeutic marker or (c) following diagnostic localisation biopsy.
- The aim is to remove all of the previous biopsy site and its margins or one or more specific margins, which are known (or suspected to be) involved in the disease process. Whenever re-excision has been performed, the surgeon should orientate the re-excision specimen. It is therefore possible to measure the distance of any additional DCIS present to the new margin of excision or to calculate the distance of the DCIS to the new margin of excision if no DCIS is present.

Re-excision Specimens (continued)

- If re-excision specimens are taken (or if diagnostic samples are sent in more than one fragment), it can be extremely difficult to measure the absolute largest extent of DCIS present. In these cases it is appropriate to take a pragmatic approach and measure the maximum distance in any piece of tissue and to add the dimensions to give an approximate total size. If, however, the orientation of the specimens can be determined, the size of DCIS can be ascertained more reliably.

Histological Examination

2-4µm thick haematoxylin and eosin stained sections should be examined.

Measurement of lesion size

The size of DCIS and distance to the nearest margin of excision should be assessed accurately using a recognised method. Such methods include use of the microscope stage Vernier scale, a calibrated eyepiece graticule or lesion mapping technique involving dot marking of the lesion boundaries on the slide coverslip. This latter method is particularly suitable for lesions examined as large blocks and sections. If the DCIS lesion extends over several blocks the measurement of distance to margins may be partly deduced by summating the thickness of the parallel slices and from measurements deduced by mapping the involvement on the specimen. Knowledge of the size of the original mammographic lesion should be available and serves as a guide to assist both accurate measurement of lesion size and the assessment of excision status in the resection specimen.

Grade of DCIS

Grade should be ascertained according to the NHSBSP Guidelines.

A high power lens (40x) should be used to compare size of tumour cells with normal epithelial nuclear size and red blood cell size. Other features such as mitotic count, presence of prominent nucleoli and polarisation of nuclei may be helpful in assigning grade. In particular a high mitotic count is very rare in DCIS not of high histological grade.

Grade of DCIS (continued)

High Nuclear Grade DCIS

Cells have pleomorphic, irregularly spaced, and usually large nuclei exhibiting marked variation in size, irregular nuclear contours, coarse chromatin, and prominent nucleoli. Nuclei are often 3 or more times the size of erythrocytes. Mitoses are usually frequent and abnormal forms may be seen. If mitoses are prominent there is a high likelihood that a case is of high grade. High grade DCIS may exhibit several growth patterns. It is often solid with comedo type central necrosis, which frequently contains deposits of amorphous calcification. Sometimes a solid proliferation of malignant cells fills the duct without necrosis but this is relatively uncommon and may be confined to nipple/lactiferous ducts in cases presenting with Paget's disease of the nipple. High nuclear grade DCIS may also exhibit micropapillary and cribriform patterns frequently associated with central comedo type necrosis. Unlike low nuclear grade DCIS there is rarely any polarisation of cells covering the micropapillae or lining the intercellular spaces.

Intermediate Nuclear Grade DCIS

These types cannot be assigned readily to the high or low nuclear grade categories. The nuclei show moderate pleomorphism, less than that seen in the high grade disease, but lack the monotony of the small cell type. The nuclear to cytoplasmic ratio is often high and one or two nucleoli may be identified. The growth pattern may be solid, cribriform or micropapillary and the cells usually exhibit some degree of polarisation covering papillary processes or lining intercellular lumina.

Low Nuclear Grade DCIS

Low grade DCIS is composed of monomorphic evenly spaced cells with rounded, centrally placed nuclei and inconspicuous nucleoli. The nuclei are usually, but not invariably, small. Mitoses are few and there is rarely individual cell necrosis. These cells are generally arranged in micropapillary and cribriform patterns. Both patterns are frequently present within the same lesion although the cribriform pattern is more common and tends to predominate. There is usually polarisation of cells covering the micropapillae or lining the intercellular lumina. Less frequently low grade DCIS has a solid pattern.

When terminal duct lobular units are involved the process can be very difficult to distinguish from lobular carcinoma in situ (LCIS) and if both intraductal and intralobular patterns are seen it is appropriate to classify the process as DCIS **and** LCIS. Features in favour of DCIS are the slightly larger cell size, readily visible cell membranes, cytoplasmic basophilia, and variation in cellular arrangement and size, greater cellular cohesion and lack of intracytoplasmic lumina.

Grade of DCIS (continued)

Mixed Types of DCIS

A small proportion of cases of DCIS exhibit features of differing nuclear grade. Such variation in cell type is unusual but, if present, the case should be classified by the highest nuclear grade present.

Architecture of DCIS

The growth pattern should be recorded. All architectural patterns present should be noted including solid, cribriform, micropapillary, flat (clinging), and papillary. Comedo DCIS is considered to represent solid DCIS (usually of high nuclear grade) with central necrosis and is not therefore included as a specific growth pattern (see below – completing the Sloane Project Pathology Form).

Rarer Subtypes of DCIS

Other rare, but morphologically distinct, subtypes of DCIS are recognised. There is, however, no firm evidence to support the distinction of special DCIS types from commoner DCIS forms with the exception of encysted papillary carcinoma in situ and apocrine DCIS. The practical problem of inter-observer disagreement in distinction of some special DCIS subtypes, particularly apocrine and micropapillary DCIS, has led to some suggesting a working classification of DCIS with five subtypes; high, intermediate and low grade, with additionally apocrine and micropapillary DCIS as separate categories. Simultaneous use of the grading system described above and subtyping according to architecture is suggested.

Apocrine DCIS

The tumour cells show abundant granular cytoplasm, moderate to severe cytological atypia and central necrosis. Apical snouting (cytoplasmic protrusions) are not always seen. The cells may sometimes be highly atypical and no necrosis may be evident. The suggested diagnosis of apocrine DCIS should be made with caution particularly in the absence of comedo type necrosis. It may be extremely difficult to distinguish atypical apocrine hyperplasia from low grade apocrine DCIS. Benign apocrine change is, of course, frequent in breast biopsy material and is recognised to show nuclear atypia, which should not be interpreted as DCIS. Atypical apocrine adenosis may also mimic apocrine DCIS or even invasive apocrine carcinoma. Identification of periductal inflammation and fibrosis may be helpful and is rarely seen in atypical apocrine hyperplasia or apocrine proliferations other than DCIS.

Encysted Papillary Carcinoma in Situ

This is a rare but distinctive form of DCIS, which is more common in older women. It carries an excellent prognosis if confined within the capsule without surrounding DCIS or foci of invasion. The presence of associated DCIS in the surrounding tissue is recognised to be of significance regarding local recurrence and should be recorded. Encysted papillary carcinoma in situ is usually circumscribed and accompanied by a hyalinised fibrous wall giving an encysted appearance. Adjacent to the fibrous capsule haemosiderin (or haematoidin) pigment is often seen. Encysted papillary carcinoma has a papillary structure with fibrovascular cores, which are usually fine but may be absent in at least part of the lesion. Other forms of DCIS, usually of micropapillary or cribriform type, may accompany it.

Clear Cell DCIS

This is an intraductal proliferation of neoplastic cells with optically clear cytoplasm and distinct cell margins forming cribriform and solid structures. Central necrosis may be present. This may be mimicked by poor fixation in other forms of DCIS and care should be taken to achieve optimum fixation, as noted above.

Signet Ring DCIS

This is a very rare variant characterised by the proliferation of signet ring cells in solid or papillary growth patterns. The cytoplasm stains positive with diastase-resistant-PAS or alcian blue.

Neuroendocrine DCIS

The lesion has an organoid appearance with prominent argyrophilia, resembling a carcinoid tumour. The neoplastic cells may be arranged in a solid pattern or may be papillary, forming tubules, pseudorosettes, palisades, or ribbons. Where solid, the proliferation is nearly always punctuated by fine fibrovascular cores. An eosinophilic cytoplasmic granularity or organoid spindle morphology is all supportive of the neuroendocrine phenotype. Because of the lack of microcalcification these tumours tend to present symptomatically, most commonly in elderly patients with blood stained nipple discharge. Immunohistochemical stains for neuroendocrine markers (chromogranin, PGP9.5, synaptophysin, NSE) may be helpful in diagnosis of this sub-type of DCIS.

Cystic Hypersecretory DCIS and Mucocoele Like DCIS

This type of DCIS is a variant of micropapillary DCIS. The cells produce mucinous secretions, which distend involved duct spaces giving a cystic appearance. Microcalcifications are often a very prominent feature.

Rarer Subtypes of DCIS (continued)

Flat (clinging) DCIS

This lesion is becoming increasingly recognised and presents particular problems of recognition. It forms part of the spectrum of columnar cell alterations which range from common benign blunt duct adenosis/columnar cell alteration through atypical forms to flat in situ carcinoma which is believed by many authorities to be a variant of low grade micropapillary DCIS.

Necrosis

Necrosis can be identified by the presence of cell ghosts and is eosinophilic and granular in nature. Karyorrhectic debris is seen. The definition of necrosis does not include single apoptotic individual cells.

Microinvasive Carcinoma

The NHSBSP guidelines definition of microinvasive carcinoma should be used. There is typically a dominant and often extensive DCIS lesion with one or more clearly separate foci of infiltration into non-specialised interlobular stromal tissue, none of which measures more than 1 mm in diameter. Tumours fulfilling these criteria are rare and if there is doubt about the presence of invasion the case should be classified as pure DCIS only. Microinvasion is very rare in DCIS other than high nuclear grade. Cases of pure high or intermediate nuclear grade DCIS and those with comedo type necrosis should be extensively sampled to exclude microinvasion or foci, which are more than 1 mm in size and represent established invasion. In particular, care should be taken to avoid overdiagnosis of cancerisation of lobules as microinvasive carcinoma.

The organoid appearance of cancerisation of lobules should be sought and deeper H&E sections from the paraffin block may be more helpful than immunohistochemical examination. However, stains that label myoepithelial cells (alpha-smooth muscle actin and myosin or cytokeratin 14,) or the basement membrane (laminin and Collagen IV) may assist in the diagnosis, as these will be absent on invasion fronts.

Atypical Ductal Hyperplasia

Atypical Ductal hyperplasia (ADH) is a rare lesion often co-existing with fibrocystic change, a sclerosing lesion, or a papilloma. The current definition rests on identification of the presence of some but not all of the features of DCIS. This is an area of diagnosis where even expert pathologists differ and acceptable diagnostic agreement is difficult to achieve. The difficulty is encountered in distinguishing ADH from low grade variants of DCIS. If pathologists adhere to the view that when cellular changes typical of DCIS occupy two separate duct spaces this is regarded as DCIS but if only one duct space is involved the disease is classified as ADH, then the majority of cases are more readily categorised; ADH is a small microfocal lesion. Two simple rules of thumb are helpful: a) not to entertain the diagnosis of ADH unless low grade DCIS has been seriously considered in the differential diagnosis and b) the larger the lesion the greater the likelihood that it is DCIS.

Tavassoli and Norris have argued that the overall geometric size of the DCIS lesion is more important than the number of duct spaces involved. Two mm in size was used as the cut-off point for the decision between ADH and DCIS in earlier published articles, a lesion less than 2mm in overall size indicating ADH. Page and co-workers have used a 3mm size cut-off in sub-classification of atypia within papillomas.

The AFIP criteria have been updated and the 2mm size criterion is now invoked *only* when assessing non-necrotic atypical intraductal proliferations with both architectural and cytological features similar to those of low grade DCIS. Proliferations with high grade cytology (with or without necrosis) qualify as DCIS, regardless of size or quantity of epithelial proliferation. Lesions that show comedo necrosis or high nuclear grade are not classified as ADH. Other pathologists would also classify a lesion as DCIS if the cellular changes typical of DCIS were present in only one duct space or if comedo type necrosis was present in the duct space in question even if the lesion was less than 2.0mm in size.

In Situ Lobular Neoplasia

The defining cell type in lobular neoplasia is round, cuboidal, or polygonal with clear or light cytoplasm. Nuclei are round to oval, cytologically bland, with an occasional small nucleolus. Mitotic figures and hyperchromatism are not often seen. There is an even distribution of cells and cellular monotony is the rule. Cytoplasmic clear vacuoles are often, although not invariable, present.

The diagnosis of in situ lobular neoplasia rests on finding the characteristic cells within *lobules*. Cytologically identical cells may focally involve the ducts in a 'pagetoid' manner. This proliferation of neoplastic cells above the basement membrane undermines the normal lining epithelial cells.

On occasions a regular, evenly-spaced monotonous population is seen within both ducts and lobules; in these circumstances it may be difficult to classify the lesion. If only scanty terminal ducts are involved and the proliferation is almost entirely lobular, the lesion is classified as in situ lobular neoplasia. However, distinguishing DCIS from lobular neoplasia may indeed be impossible if both an organoid lobular and ductal component are identified. If both ducts and lobules contain epithelial proliferation of this type, categorisation as both lobular neoplasia and DCIS is recommended at the present time to infer the precursor risk of DCIS and the bilateral cancer risk of lobular neoplasia.

Receptor Measurement

The methods used for receptor assay should follow national guidance. Laboratories performing such assays should participate in the relevant UK NEQAS scheme.

COMPLETING THE SLOANE PROJECT PATHOLOGY FORM

One pathology data form should be completed for one episode of DCIS. It is presumed that this will be completed by the pathologist once complete excision of a lesion has been achieved.

Demographic Details: Please indicate surname, forename, date of birth, hospital where pathology was reported and hospital number, also screening number (if known).

Therapeutic Excision Report Number: It is recognised that some patients may have undergone multiple biopsies for diagnosis and therapeutic purposes. It is not necessary to provide all these data but please indicate the therapeutic excision report number for reference.

Side: Indicate left or right breast. For specimens from both sides, a separate form should be completed for each side.

Specimen Type: Choose one of the following terms:

Core biopsy

11 gauge core biopsy (e.g. Mammotome)

Diagnostic open biopsy - Includes lumpectomy, tylectomy, dochectomy etc, taken for diagnostic rather then therapeutic purposes.

Therapeutic excision - Includes lumpectomy/wide local excision, wedge excision, segmentectomy and partial mastectomy specimens with the aim of complete excision of the lesion.

Bed biopsy/cavity shave - A specimen shaved from one or more edges of the biopsy cavity of a therapeutic excision sent separately by the surgeon.

Immediate re-excision specimen - A re-excision specimen, of a specified margin, taken at the time of therapeutic procedure.

Delayed re-excision specimen - A re-excision specimen, usually of biopsy cavity and a specified margin, taken at a subsequent surgical procedure.

Completion mastectomy - Mastectomy performed for the purposes of obtaining complete excision as a subsequent surgical procedure.

Mastectomy

Specimen weight: Record the weight of any specimen if known. Weight is more reproducible than 3 dimensional measurements to determine volume, even taking into account the different densities of fat and fibrous tissue, which vary in proportion in breast specimens. Specimen weight is also used as the means of determining the likely cosmetic disadvantage to women undergoing benign biopsy in the NHS BSP.

Nipple Orientation marker present? Please indicate whether the surgeon has separately identified the nipple margin of the therapeutic excision. Surgical guidelines for the Sloane project recommend that this be undertaken.

Other Orientation markers present? Please indicate whether the surgeon has appropriately identified other margins of the specimen, e.g. with sutures of described length or clips, so that orientation is clear to the pathologist.

Cut up/specimen information (for wide local excision specimens) Please indicate how the therapeutic excision specimen has been sampled and which Sloane cut up protocol was used (see above). If another technique was used it would be helpful if the protocol was described and the protocol used was provided.

Were specimen slices X-rayed? Please indicate whether, after slicing, the specimen was re-X-rayed.

Was a macroscopic abnormality present? Some cases of DCIS may present as a mass and sampling may be targeted at a specific macroscopic abnormality. Please indicate if this was the case.

Number of blocks taken to assess margins and number of blocks taken overall: Please record the number of blocks taken specifically to assess whether the margins of excision were clear of DCIS and the overall number of blocks taken of the wide local excision. This will clearly vary according to the mode of sampling and the size of the specimen. In some cases it may not be possible to specifically ascribe a block as taken to assess the margin or the lesion; if these include the margin of excision they should be recorded in both sections.

Histology: Please indicate whether DCIS, ADH and lobular neoplasia are present. Tick all that are identified.

The subsequent sections refer to DCIS ONLY

Maximum (total) size of lesion mm: Please indicate the total size of the DCIS. This may be a summation from the total in several specimens e.g. diagnostic, therapeutic and re-excision samples, and may therefore necessarily be an approximation.

Highest nuclear grade: Rarely DCIS may demonstrate more than one nuclear morphology, please indicate the highest nuclear grade present.

Growth pattern: Please indicate **all** architectural patterns of DCIS present from solid, micropapillary, cribriform, papillary, apocrine, flat/clinging or other. In addition please record any special features such as neruoendocrine phenotype or clear cell morphology.

Comedo DCIS is considered to represent solid DCIS (usually of high nuclear grade) with central necrosis and is not therefore included as a specific growth pattern.

Comedo Necrosis present: Indicate whether there is central necrotic debris present. Individual cell death is not considered to represent necrosis and should not be recorded.

Paget's disease present: Indicate whether there is associated Paget's disease of the nipple in specimens which include nipple and skin. If nipple is not included in the sample please tick not applicable/not known.

Microinvasion (<1mm) and number of foci of microinvasion? Indicate whether there is no, definite or possible microinvasion, seen as one or more clearly separate foci of infiltration into non-specialised interlobular stromal tissue, none of which measures more than 1mm in diameter. Please also indicate the number of foci of definite microinvasion.

Margins

Distance to margins in therapeutic excision: Please record the distance of DCIS to margins in the therapeutic surgical procedure. Please complete all available; it is recognised that not all of the measurements will be available in all cases. If any/all of the margins are more than 10mm distant this is sufficient.

If altered by further surgery: The margins may have been altered by subsequent surgery/re-excision. Please also provide estimated final distance to specific margins if further surgery has altered the measurement from the therapeutic procedure.

Does another process extend to the margin? Distance of ADH of lobular neoplasia to margin: The clinical significance of ADH and lobular neoplasia at the margins of excision is unclear. Please indicate whether ADH and /or lobular neoplasia are present at the margin and the distance of this process to the nearest margin of excision.

Immediate re-excision/cavity shaves/bed biopsies: Please record the number of samples received from the surgeon and the total number of blocks taken from these specimens.
Please indicate whether DCIS, ADH, and/or lobular neoplasia were present in these additional specimens.

Nodes: Please record the number of nodes examined and the number positive from the axilla, as sentinel nodes or others, such as internal mammary nodes.

Receptor Status: Please record the oestrogen and/or progesterone receptor status and HER2 status, using the cut offs described on the data form. Alternatively indicate if not known/not performed.

Date and pathologist: Please record the data of completion of the form and print your name.
