# Supplementary material for: Cohort profile of the Sloane Project: methodology for a prospective UK cohort study of >15 000 women with screen-detected non-invasive breast neoplasia
Source: BMJ Open. 2022 Dec 19;12(12):e061585. doi: 10.1136/bmjopen-2022-061585 (PMC9764674; doi:10.1136/bmjopen-2022-061585)
Supplement: Supplementary data [file bmjopen-2022-061585supp004.pdf]

The Sloane Project
Understanding non-invasive breast disease

NHS

Sloane Project Atypia Audit

Radiology Protocol

Published July 2014

Version 1.0

www.sloaneproject.org.uk

Operated by Public Health England

Guidelines for completion of Sloane Project Radiology data form

Demographic Details

Please indicate date of birth, screening unit, screening number (SX number), screening round number (this is the number of times the patient has been screened as part of the NHS Breast Screening Programme), date of screening mammogram and NHS Number. The surname and forename boxes are for use within the Trust that is entering the patient, and should be crossed out before returning to the Sloane Project Manager.

Side

Indicate left or right breast. A separate form is required for each side in the cases of bilateral disease. For multi-focal disease in a single side, only one form should be completed.

Site

Please indicate all sites that are involved from the following: Upper Outer Quadrant, Upper Inner Quadrant, Lower Outer Quadrant, Lower Inner Quadrant and Retroareolar.

For multi-focal disease, it is important that there is concordance of the site which is reported on the pathology form with that which is measured on the radiology form. As these cases are being identified at the postoperative MDM, correlation should take place at this time.

Background Pattern

During the pilot phase of the radiology data collection a number of classifications were used to describe the background parenchymal pattern. Recognising that this is a highly subjective classification, the pilot centres agreed that the birads classification was the most reproducible.

Please therefore indicate which one of the following patterns best describes the Background Pattern: Fatty, Scattered Fibroglandular Density, Heterogenously Dense and Extremely Dense.

Predominant Radiological Feature

Please choose the predominant radiological feature on the mammogram and classify as appropriate. Please tick one box only of the following features: Calcification, Parenchymal Distortion, Mass-Well Defined, Spiculate Mass, Mass-Ill Defined or None of the above.

Operated by Public Health England

Calcification

With regard to the classification of calcification, two systems of classification have been included ~ please choose one system only and examples of the three groups are enclosed in picture format at the end of this document.

To clarify, however, the classification is based only on the shape of the individual calcifications. i.e. the presence or absence of a prominent ductal distribution should not influence the classification.

1. **Casting/linear:** this should include any case where there is even a single linear or branching calcification even if the rest are granular or punctate in shape.
2. **Granular/crushed stone:** this should include cases which have irregularly shaped calcifications but no linear forms. Some or even most of the calcifications may be punctate.
3. **Powdery/punctate:** All calcifications are either round or oval in shape.

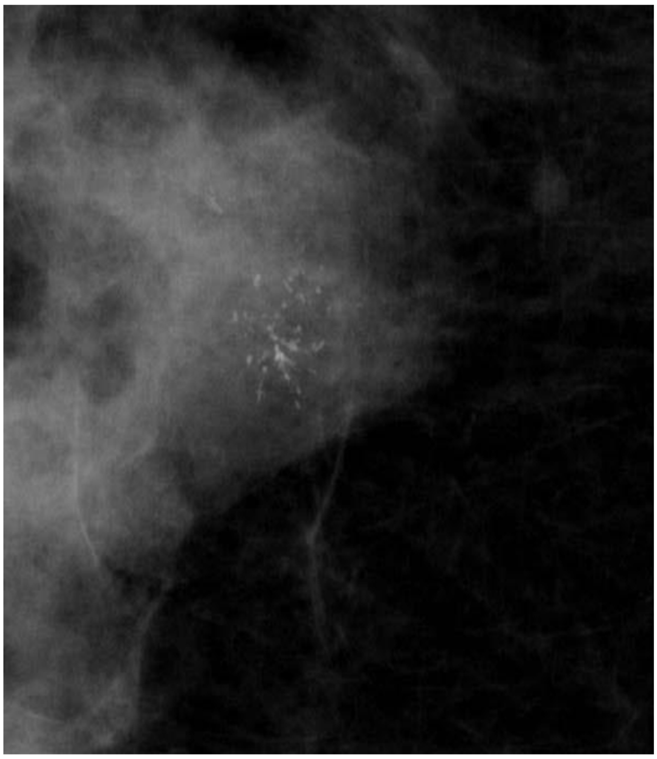

Figure 1. Localised casting type calcification

Operated by Public Health England

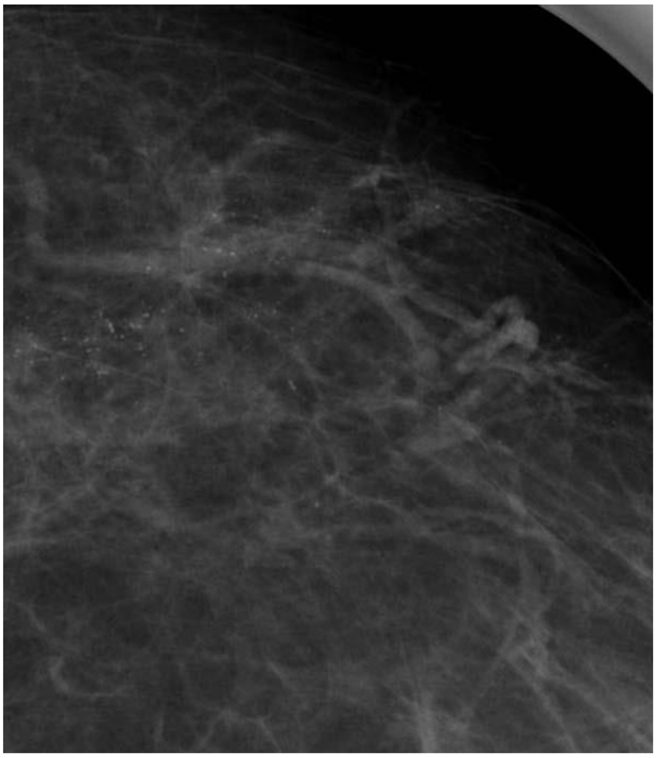

Figure 2. Extensive granular (Nottingham classification) or Crushed Stone (Tabar classification) calcification

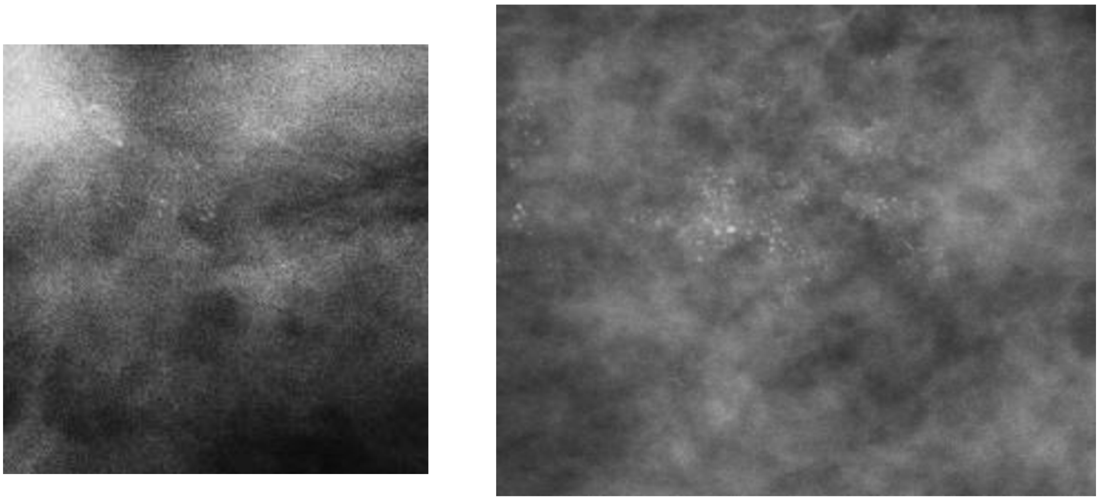

Figure 3. Punctate (Nottingham classification) or Powderish (Tabar classification) calcification

Operated by Public Health England

Estimated Size of Lesion on Mammograms

All measurements have to be done both on the oblique view and the CC views. Three reproducible measurements have been chosen:

1. **Distance from base of nipple to nearest part of the lesion (mm)** i.e. border of mass/first dot of calcium, closest area of parenchymal distortion (see Figure 4 for example).
2. **Maximum length of lesion (mm)** (see Figure 5 for example).
3. **Maximum diameter of lesion at 90^0 to long axis of lesion (mm)** (see Figure 6 for example)

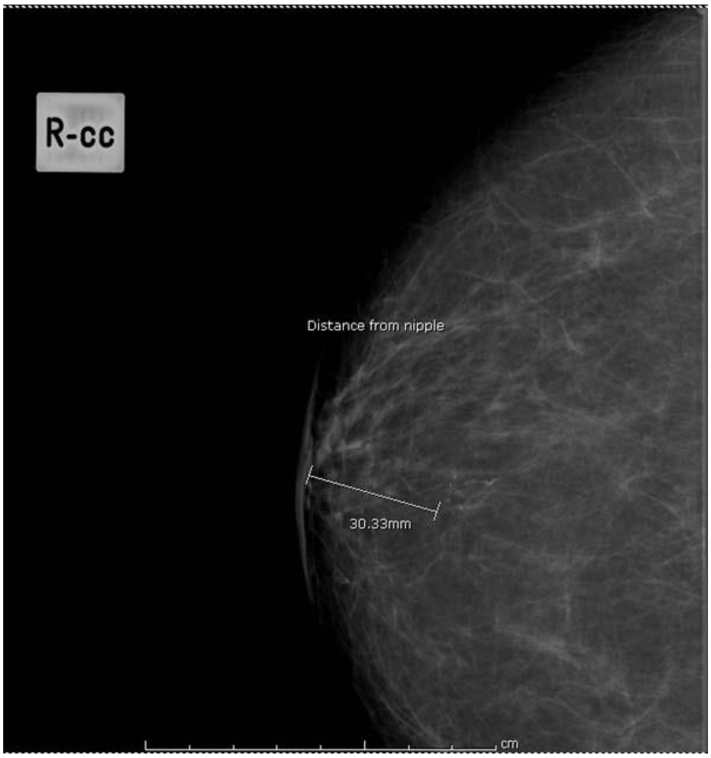

Figure 4. Measuring distance from nipple to lesion

Operated by Public Health England

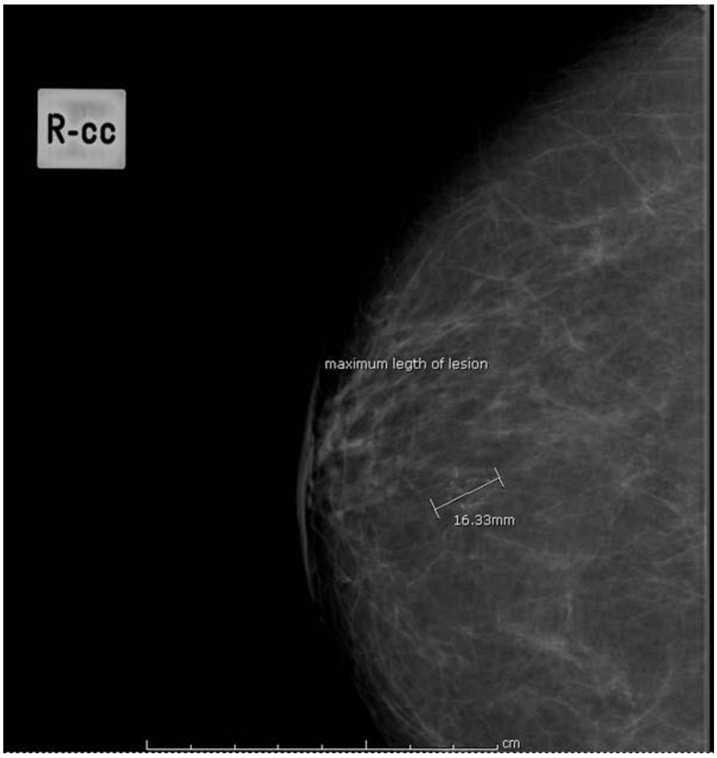

Figure 5. Measuring maximum length of lesion (mm)

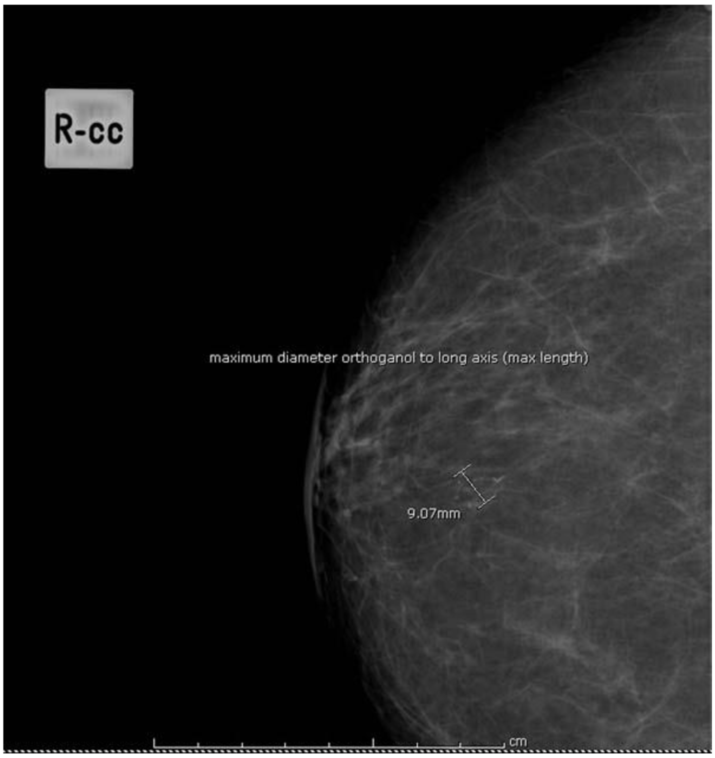

Figure 6. Measuring maximum diameter of lesion (mm)

Operated by Public Health England

Please note that as cases of distortion are few and far between and because of difficulties in relation to measuring these cases, it has been agreed that measurements of distortions are no longer required. This includes measuring distance from nipple.

In cases where there are two distinct areas separated by apparently normal breast tissue, then the measurement should be from one end of one site to the further most end of the other site, along with all the apparently normal breast tissue in between. The reason for this is that it reflects how we clinically manage such cases (i.e. if DCIS is found in two sites with apparently normal breast tissue in between the patient will generally be subjected to mastectomy) (See Figure 7).

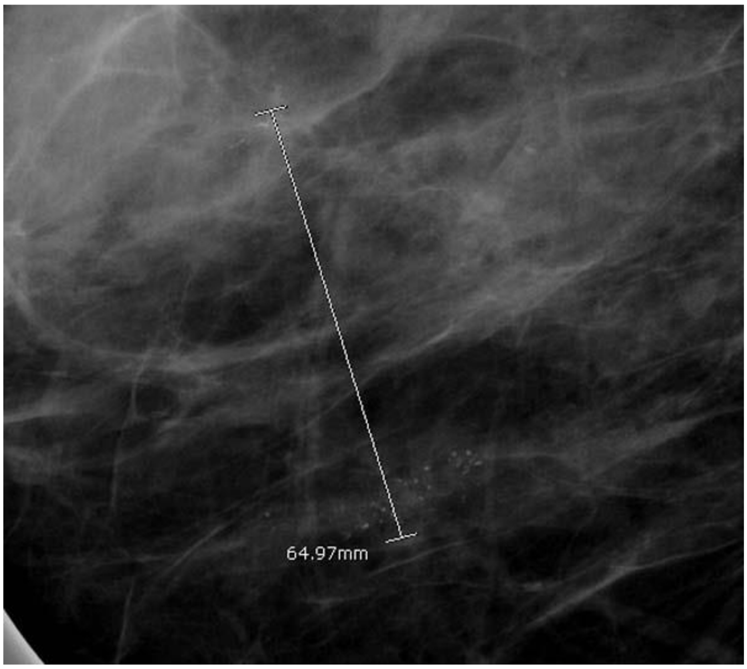

Figure 7. Measuring multi-focal disease

Data required to Calculate Breast Volume

The accompanying formula (1/3 pi r^2h) has been validated and is thought to be the closest approximation to breast volume that can be carried out from 2 view mammography. The labels “a” and “b” correspond to the measurements “h” and “2r” that are required for the breast volume formula.

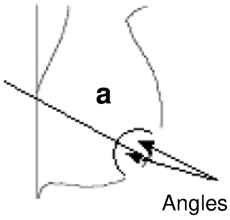

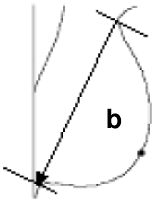

Chest wall to nipple (in the nipple plane)

Infra mammary fold to supra mammary (axillary) fold

Operated by Public Health England

For clarification, the diagram of the MLO shows the perpendicular from the nipple running through the intersection of the anterior border of the pec major and the back of the image. When the mammographic image doesn't completely coincide with this diagram, please measure the perpendicular to the back of the image rather than the front of the pec.

Please note that even though the size of the lesion and distance from the nipple are not to be measured in cases of distortion, the data to calculate breast volume is still required.

Magnetic Resonance Imaging (MRI)

Please indicate whether a pre-operative MRI was performed, and the estimated maximum dimension of the lesion on MRI (mm).

Specimen X-Ray

Please indicate whether a Specimen X-Ray was carried out and identify by tick whether the specimen is as a result of a diagnostic or therapeutic biopsy. Please record whether the lesion is present in the Specimen X-Ray and if it appears to have been radiologically completely excised. With regard to completeness of excision, arbitrarily 2mm (unmagnified) of "normal" breast tissue has to be present between the margins which are closest to the mammographic abnormality.

With the increase in the use of mammotomy to diagnose these cases pre-operatively, there is the possibility that in some cases all the calcifications will have been removed and that a clip will be left in situ. For the purposes of completion of this section, it is possible to say that the entire lesion has been removed, but it is inappropriate to make measurements as to proximity to the margin etc.

Further radiological guidelines can be found on the Sloane Project website (www.sloaneproject.org.uk) Radiology Training Resource page or via the Sloane Project Manager.

Operated by Public Health England

Sloane Project Atypia Audit Pathology Protocol

Published April 2019

Version 2.0

phe.sloaneproject@nhs.net

Operated by Public Health England

Guidelines for Pathological Assessment of Atypical Intra Ductal Epithelial Proliferation (AIDEP), Atypical Ductal Hyperplasia (ADH), Lobular In Situ Neoplasia (LISN), Pleomorphic LCIS (PLCIS) and Flat Epithelial Atypia (FEA)

Introduction

The Sloane Project Phase One Pathology Protocol (available on request), contains detailed information about surgical and laboratory handling of therapeutic surgical specimens, including sampling techniques and cut-up protocols. It also describes the assessment and measurement of margins and what is required from the histological examination. The Sloane Phase One protocol largely refers to ductal carcinoma *in situ* (DCIS), which was the predominant type of screen-detected lesion recorded in the first phase of the Sloane Project. This protocol refers to the ongoing collection of screen-detected atypia of the breast by the Sloane Project. The Sloane Project no longer collects data on patients with DCIS primaries.

Specimen Handling

This Sloane Project Atypia Audit Pathology protocol gives some more detailed and updated guidelines for the pathological assessment of atypical ductal hyperplasia (ADH), lobular in situ neoplasia (LISN), pleomorphic LCIS (PLCIS) and flat epithelial atypia (FEA) which are the screen-detected lesions being recorded in the second phase of the Sloane Project (atypia audit). It is anticipated that these ‘atypical’ lesions are more likely to arise in *diagnostic* (rather than therapeutic) surgical specimens, as well as core biopsies and vacuum-assisted wider gauge samples; thus specimen handling for these may be different from DCIS lesions and pleomorphic LCIS. Such specimens may not be orientated by the surgeons who aim to remove the screen-detected abnormality, without surrounding disease-free margin (less than 20g tissue). For this reason such specimens may not requiring inking in different colours and distance to margins of these atypical lesions may not be applicable. Further guidance can be found in the updated NHS BSP publication 58.

The Sloane Project
Understanding non-invasive breast disease

NHS
Cancer Screening Programmes

Core Biopsy and Diagnostic Vacuum Assisted Biopsy

Such specimens, typically for the diagnosis of screen-detected microcalcification, require adequate fixation in sufficient volume of formalin, routine processing and examination at three levels with haemotyxlin and eosin-stained sections, as per NHSBSP guidelines. Correlation of the histological and radiological extent, degree and size of the microcalcifications at multidisciplinary team meeting is essential. Additional levels may be advised in the assessment of some of these borderline atypical lesions.

Therapeutic Vacuum-Assisted Biopsy

In some centres, after initial narrow (e.g. 14g) or wide gauge (e.g. 8 or 11g) core biopsy, the individual Unit protocol for ‘B3’ lesions may be that wider, vacuum-assisted *excision* biopsy be performed. As for *diagnostic* vacuum-assisted samples, these should be adequately fixed and all the cores examined with haematoxylin and eosin-stained sections. When the diagnosis is of flat epithelial atypia or atypical ductal hyperplasia, it is anticipated that additional levels will often be performed to exclude the diagnosis of low grade DCIS and/or associated invasive carcinoma.

Diagnostic Localisation Excision Biopsies

These specimens are produced when a definitive pre-operative diagnosis of malignancy has not been made but where there are suspicious radiological or clinical findings, or when the pre-operative diagnosis is of an indeterminate (e.g. C3/B3 or C4/B4) lesion.

- The specimen should be inked, weighed, measured in three dimensions and then, usually, serially sliced at intervals of approximately 3-5mm.
- Specimens containing impalpable mammographic lesions, such as microcalcification or sometimes a tissue marker, will require specimen slice x-ray examination if a block selection process is undertaken (i.e. those that are not embedded in their entirety). This enables blocks to be taken from the areas corresponding to the mammographic abnormality as well as any other suspicious areas identified.
- The sites of sampling can be marked on the specimen x-ray, or the x-ray of specimen slices, by using a white wax (*Chinagraph*) pencil or other marker if hard copy film is used. Digital images can also be annotated to indicate sites of block selection.
- The sampling technique and the number of blocks taken are clearly dependent on the size of the specimen and the size of the abnormality. If the specimen is small (e.g. less than 30mm), it is best to block and examine all of the tissue.

Operated by Public Health England

- Blocks should be taken ideally to enable assessment of the histological size of the lesion. Where the maximum macroscopic dimension of a tumour can be blocked directly, it is recommended that a single block across this aspect be taken.
- Where a lesion is larger than can be assessed in a single block, a large (mega) block to encompass the maximum dimension may be taken. When taking large blocks at least one other normal sized lesional block should be processed as well, to allow optimal processing and to avoid the excessive use of antibodies in any immunohistochemistry.
- If large blocks are not available, two or more blocks are recommended from the maximum macroscopic dimension, so that the total lesion size can be estimated by adding the portions together or measuring the maximum dimension on the two slides fitted together. A diagram may be made of how the adjacent blocks relate to each other to avoid misunderstanding of this relationship at the time of microscopy, with the risk of overestimating the lesion size. Occasionally, when the plane of maximum dimension of a non-spherical lesion is not known or is anticipated incorrectly, it may be necessary to stack together the estimated block thicknesses along that axis, to obtain a measurement. This will always be fairly inaccurate and the need to try to find the plane of maximum dimension of the tumour prior to slicing is important.
- For larger specimens, sampling should be adequate to determine accurately the size of the lesion. Sampling should include the extremes of the mammographic abnormality and adjacent tissue in order to avoid underestimation of size. This is particularly important with cases that prove to be DCIS (e.g. including cases with a pre-operative core biopsy containing an atypical ductal epithelial proliferation) as it is recognised that mammographic size may be an underestimate of true size.
- If specimens are sent as more than one piece of tissue, it can be impossible to measure the absolute extent of the lesion. In these cases, it is appropriate to take a pragmatic approach and to measure the maximum size in each piece of tissue and add the dimensions to give an estimated total size. If, however, the orientation of the specimens can be determined, the true size can be ascertained more reliably.
- If calcifications cannot be seen histologically, specimen block x-ray may be helpful to determine their location in the sampled tissue.

Therapeutic Excisions

It is considered unlikely that (other than for pleomorphic LCIS) the atypical lesions to be recorded in the Sloane Atypia Audit will be seen alone in a therapeutic excision specimen. However, lesions that have a pre-operative diagnosis of malignancy (DCIS or pleomorphic LCIS) and are deemed to be suitable for breast conserving surgery with regard to clinical/radiological size may be excised as a therapeutic wide local excision.

- It is usual for the surgeon when performing a therapeutic operation to take all of the tissue from the subcutaneous aspect to the pectoral fascia as per Surgical Guidelines for the management of breast cancer. It is essential that the pathologist be informed if the usual surgical protocol has not been undertaken as this will affect the optimum specimen handling methodology e.g. central excisions. In such circumstances, when tissue remains at the deep (posterior) or superficial (anterior) aspects of the specimen, the distance to these margins is clinically relevant.
- The surgeon should orientate cancer resection specimens. Each unit should establish a code of orientation using either different lengths or numbers of sutures or metal staples/clips or ink. The code should be anatomically relevant and assist in accurate evaluation of the specimen and its margins. The nipple extension/direction of the nipple should ideally be separately marked by the surgeon and it is helpful if the request form indicates the site of the tumour within the breast so that the radial margin of the specimen nearest the nipple can be identified more easily.
- The specimen should be weighed and measured in three dimensions.
- The specimen excision margins should be inked and the specimen can be sliced either before fixation or (less preferably) after fixation.
- The technique for examining the specimen and sampling the abnormality will vary according to type of sample and the lesion nature (e.g. mass, calcification etc) and lesion size and also according to pathologist/laboratory preference; therefore a degree of flexibility is required. Several options are available. Whichever is utilised, as an absolute minimum, the information for the breast cancer minimum dataset, including accurate measurement of size and detailed examination of the margin status and distance to margins, must be provided.

Wide Local Excisions for lesions presenting as mammographic calcification

Pleomorphic LCIS often also presents as radiologically-detected calcifications and it is presently recommended that such specimens are handled as per those with a pre-surgical diagnosis of DCIS

- DCIS and pleomorphic LCIS typically present as a mammographically detected abnormality, usually calcification, which may not be visible on macroscopic examination of the sliced tissue. Specimens from patients presenting with such lesions will therefore benefit from a combined radiological-pathological approach to diagnosis.
- It is usual for the surgeon when performing a therapeutic operation to take all of the tissue from the subcutaneous aspect to the pectoral fascia. It is essential that the pathologist is informed if the usual surgical protocol has not been undertaken as this will affect the optimum specimen handling methodology e.g. central excisions, or specimens where breast tissue remains at the

deep (posterior) and superficial (anterior) aspects of the excision, and the distance to these margins is thus clinically relevant.

- The surgeon should mark the nipple duct margin; DCIS tracks towards the nipple and, in this plane in particular, can be some distance from the obvious area of microcalcification. It is not known whether pleomorphic LCIS demonstrates a similar growth pattern. It is helpful if the request form indicates the site of the lesion within the breast so that the margin of the specimen nearest the nipple can be identified.
- The specimen should have been X-rayed intra-operatively prior to receipt in order to confirm the presence of the lesion within the specimen. The intraoperative radiographs – whether digital or hard copy film - should be available to the pathologist who should be aware of the size and location within the specimen of the radiological abnormality
- The specimen should be weighed and measured in three dimensions.
- The specimen excision margins should be inked and the specimen can be sliced either before or after fixation. The use of different colour inks/markers on an individual section can assist microscopic identification of specific margins. Inks which are radio-opaque should ideally be avoided if applied prior to slice X-ray.
- If the specimen is large, then incision before fixation is recommended. The specimen should be sliced at intervals of approximately 3-5mm
- Serial slicing enables specimen slice radiographic mapping of the specimen which provides a high level of confidence that the lesion has been accurately and adequately sampled; slicing and X-raying the specimen slices enables blocks to be taken most accurately from the areas corresponding to the mammographic abnormality as well as from any other suspicious areas identified. This is essential to avoid underestimation of lesion size and overestimation of the distance to specimen margins. Sites of sampling can be annotated on the specimen radiograph for radiological-pathological correlation.
- Macrophotography or schematic diagrams may also assist in recording macroscopic findings and the block map as well as identifying individual sampled margins.
- Sampling may be facilitated by the identification of any radiological marker (e.g. clip, collagen marker or coil). Tissue changes relating to previous core biopsy are an important landmark to indicate sampling of the site of the index lesion and should be recorded in the report, particularly if the whole abnormality was removed by the cores.
- The macroscopic and or radiographic lesion should be described and its size in 3 dimensions and distance to margins recorded.
- The number of blocks taken will depend on the size of the specimen and the size of the abnormality. If the specimen is small, or if slice radiology unavailable, it is best to block and

Clements K, *et al. BMJ Open* 2023; 12:e061585. doi: 10.1136/bmjopen-2022-061585

examine all of the tissue. Samples 30mm or less in maximum dimension should be completely sliced, embedded and examined histologically. The site of all blocks taken should be recorded.

- For larger specimens sampling should include the extremes of the radiographic calcification and adjacent tissue in order to avoid underestimation of the size of the lesion. This is particularly important for microcalcifications associated with DCIS, as it is recognised that mammographic size may be an underestimate of the true DCIS size. Comparable data is not available for pleomorphic LCIS but it is recommended that a similar approach for specimen handling to that for cases of DCIS is applied at present.
- Representative samples from the entire involved area should be taken. Sufficient blocks should be taken to identify associated invasive carcinoma if present. Samples should include areas of breast tissue from proximal (towards the nipple) and distal to the calcification, as DCIS extends more frequently in this plane; again, there is no data to indicate whether the same is true for pleomorphic LCIS. Defining the minimum number of blocks that should be taken remains unclear, however it is recommended that one to two block per 10mm of the maximum dimension of the area of calcification be taken. Measurement can be made in this way from the most distal involved duct across the main area of calcification to the most proximal involved duct.
- Many units use large blocks to embed the entirety of segmental excisions although the proper processing of these can delay the reporting of the case and storage may also be problematic; units should therefore take a pragmatic approach.
- All surgically relevant margins of therapeutic excision specimens should be sampled. This will include all radial/circumferential margins (superior, inferior, medial, lateral and nipple margins), and the deep (posterior) and superficial (anterior) if dictated by local protocol or by the surgical procedure from an individual patient. Particular attention should be paid to the margin nearest the mammographic abnormality and the margin nearest the nipple.
- If therapeutic samples are sent in more than one portion, it can be extremely difficult to measure the absolute largest extent of the whole lesion present. In these cases, it is appropriate to measure the maximum distance in any piece of tissue and to add the dimensions to give an estimated total size. If, however, the orientation of the specimens can be determined, the size can be ascertained more reliably.

Cavity Shave/Biopsy Specimens

- The circumferential edge of a wide local excision specimen can be shaved and examined by the pathologist to allow more extensive examination of the relevant surgical resection margins. This is typically done after taking the radial tumour blocks, and particularly if a distance of more than 2mm to the surgical margins is required for completeness of excision in local protocol. This can

produce a series of additional blocks including: superior shave, supero-lateral shave, lateral shave, infero-lateral shave, inferior shave, infero-medial shave, medial shave and supero-medial shaved edge, depending on the size of the specimen. This peripheral shaving technique is not applicable to the anterior or posterior aspects; narrower margins are often acceptable at these aspects and widths of normal tissue of less than 2mm are regarded as complete excision.

- Alternatively, the surgeon may provide separate cavity shaves, which may be submitted to the laboratory as 'bed biopsies'. The site of each specimen should be clearly labelled and each specimen examined separately.
- It should be noted that shaved edged of the margins of the specimen or examination of 'cavity shaves/bed biopsies' assess adequacy of excision but do not allow measurement of distance between tumour and margins.
- Cavity shave specimens should be distinguished from more substantive margin re-excision specimens.
- Cavity shave specimens submitted by the surgeons are generally a sheet of fibroadipose tissue with marker suture or other marking on one surface. After weighing and measuring, paying particular regard to their thickness, their new external surface should be inked, taking great care not to ink the internal margin.
- Small cavity shaves should be blocked out in total. If of a larger size, such that this is impractical, they should be sliced and the densest or most suspicious areas blocked out.

Re-excision Specimens

If a radiological abnormality extends close to a margin on an intraoperative specimen radiograph, the surgeon may undertake an immediate re-excision of that particular margin. Similarly, if there is a surgically palpable abnormality extending close to a margin intraoperatively during the operation, the surgeon may undertake an immediate re-excision of that particular aspect. A separate re-excision specimen may therefore be taken either: (1) at the time of initial surgery; (2) subsequent to the discovery of incomplete excision in a therapeutic excision for malignancy; (3) following diagnostic localisation biopsy when malignancy has been diagnosed. The aim of such a procedure is to remove either all of the previous biopsy site and its margins, or one or more specific margins known, or suspected, to be involved by the disease process. Whenever re-excision has been performed, the surgeon should orientate the re-excision specimen.

- Re-excision specimens should be weighed, and serially sliced at 3-5mm.
- Blocks taken should be recorded in such a way as to permit accurate assessment of the adequacy of excision and size of any malignant lesions identified.

- It is difficult to be prescriptive regarding the extent of block sampling as the nature and size of these specimens varies; the focus should be on the new excision margin rather than exhaustive detection of residual disease.
- If re-excision specimens have been taken which contain further tumour, it can be extremely difficult to determine the absolute size of lesion. A pragmatic approach is required, and the maximum distance in each piece of tissue can be measured and added to give an approximate total size of tumour. If, however, the orientation of the specimens can be determined, the size of tumour can be ascertained more reliably.
- The pathologist should measure the distance of any additional tumour present to the new margin of excision, or to approximate the distance of the original tumour to the new margin of excision if no tumour is present.

Histological Examination

2-4µm thick haematoxylin and eosin stained sections should be examined for all specimen types.

Columnar Cell Lesions

Columnar cell lesions (synonyms: blunt duct adenosis, columnar cell change, columnar cell hyperplasia, unfolded lobule, columnar alterations with prominent snouts and secretions (CAPSS)), in broad terms include the spectrum of changes ranging from bland columnar cell change through columnar cell hyperplasia to flat epithelial atypia. They have become increasingly identified clinically as a consequence of more rigorous investigation of radiological calcification and are most commonly seen in association with fibrocystic change. They represent a spectrum of lesions:

- Columnar cell change
- Columnar cell hyperplasia
- Flat epithelial atypia
- Flat high grade in situ carcinoma

Columnar Cell Change and Columnar Cell Hyperplasia

These entities are benign and are NOT included in the Sloane Audit data collection. However, the histological features are included here for completeness and for diagnosis consideration of the differences between these and flat epithelial atypia.

Classical columnar cell change is comprised of lobular acini lined by epithelial cells that are tall and snouted in a manner similar to that observed in tubular carcinoma. Commonly this is associated

with luminal secretions and/or microcalcifications. If there is a piling up of several layers the term '*columnar cell hyperplasia'* is used, assuming that the stratification is real, as opposed to artefactual as a consequence of cross-cutting. There is morphological diversity within these groups, for example the hyperchromasia of the nuclei can vary, as well as nuclear shape and the 'tallness' of the cells; thus in some cases some lesions are more cuboidal than columnar. In columnar cell change, lobules are often dilated and are lined by epithelial cells with a columnar morphology. Other features include increased cytoplasm and apical snouts. The associated luminal secretions often undergo calcification. A single layer of columnar epithelial cells is the norm, although minor multilayering and tufting may be present. If a greater degree of multilayering of the epithelial cells is seen, the process is classified as columnar cell hyperplasia. At present, this is considered to be equivalent to usual epithelial hyperplasia and data is not collected in the Sloane Audit.

Flat Epithelial Atypia

If superimposed mild cytological atypia is seen within the columnar cell change in the terminal duct-lobular unit, the lesion is classified as flat epithelial atypia. In this lesion the cells are morphologically similar to those of atypical ductal hyperplasia/low grade DCIS but are present, typically in a single layer lining round, mildly dilated acini. The nuclei are typically round or oval and evenly spaced. The cells may have clumped chromatin or vesicular nuclei or prominent multiple nucleoli. Mitoses are infrequent. If there is marked cytological atypia is seen the lesion is regarded as flat high grade in situ carcinoma and NOT as FEA.

Differential diagnosis of FEA

True micropapillary structures and rigid epithelial bridges are not seen in columnar cell change or columnar cell hyperplasia. If such architectural atypia, usually in the form of bulbous micropapillary structures, is identified, the lesion should be assessed for degree within the membrane-bound spaces and overall extent, and classified as atypical ductal hyperplasia or low grade DCIS accordingly.

Apocrine metaplasia may occasionally mimic FEA, and vice versa; both may have apical snouts and both line the acini. In such circumstances, immunohistochemistry for oestrogen receptors may be helpful; apocrine lesions are typically ER negative whilst columnar cell lesions are uniformly strongly ER positive.

As well as ADH/low grade DCIS, other epithelial proliferations may merge, or be associated, with columnar cell hyperplasia, including atypical lobular hyperplasia, lobular carcinoma in situ (LCIS) and invasive carcinoma, often of low grade tubular or tubulo-lobular type.

Atypical intraductal epithelial proliferation (AIDEP)

There is a range of intraductal epithelial atypia short of that required for a definite diagnosis of ductal carcinoma in situ. The definition of atypical ductal hyperplasia (ADH) is derived from surgical resection specimens and relies on a combination of architectural, cytological and size extent criteria; ADH is defined as an intraductal epithelial proliferation showing the features of low grade DCIS, but in less than two duct spaces or less than 2 mm in diameter. For this reason, accurate diagnosis of ADH is not possible on core biopsy.

In order to preserve the specific (bilateral) increased risk of subsequent breast cancer conferred by the diagnosis of ADH, the term AIDEP is therefore recommended, both for lesions akin to ADH in core biopsy as well as some lesser degrees of atypical epithelial proliferation that are of sufficient to be of concern histologically.

Atypical Ductal Hyperplasia

Atypical ductal hyperplasia (ADH) is a rare lesion. Its current definition rests on identification of some but not all features of DCIS. Difficulties are encountered mainly in distinguishing ADH from the low grade variants of DCIS. The diagnosis of ADH is based on both a *qualitative* and *quantitative* assessment of the lesion.

The *qualitative* assessment is based on cytological features and architectural growth pattern. These include:

- A uniform monomorphic luminal epithelial cell population (ER, CK8, 18, 19 positive, CK5, 5/6, 14 negative
- Even spacing of the nuclei
- Secondary lumina, some of which are rigid whereas others are tapering
- Hyperchromatic nuclei
- Cribriform, micropapillary or solid growth pattern.

The *quantitative* assessment is based on assessment of lesion size:

- Areas of ADH are usually microfocal/small, not exceeding 2–3 mm in size/involve less than two complete membrane-bound spaces.

Proliferations with high grade cytology qualify as DCIS, regardless of the size or quantity of epithelial proliferation.

The diagnosis of ADH is made in those cases in which a diagnosis of **low grade** DCIS is seriously

considered but where the architectural, cytological and quantitative features do not amount to a confident diagnosis of DCIS. If a diagnosis of ADH is contemplated, extensive sampling and/or levels should be undertaken to search for more evidence to establish an unequivocal diagnosis of DCIS.

USEFUL RULES OF THUMB TO DISTINGUISH ADH FROM DCIS

- Restrict diagnosis of ADH to those cases in which DCIS is seriously considered but where the features are not sufficiently developed to make a confident diagnosis.
- DCIS usually extends to involve multiple duct spaces. If a lesion with features of ADH extends widely, the diagnosis of ADH should be questioned.

Atypical Lobular Hyperplasia and Classical Lobular Carcinoma *In Situ* (*In Situ* Lobular Neoplasia)

Atypical lobular hyperplasia (ALH) and lobular carcinoma in situ (LCIS) have traditionally been separated as distinct entities based on cytological and quantitative features relating to the extent of lobular involvement. The justification for separating the entities is the differing risks of subsequent invasive cancer shown in long-term follow-up series, but molecular analysis suggests that biologically the two appear to be essentially similar and that these are neoplastic processes. In view of the subjective nature of separating ALH from LCIS and the similar molecular profiles, some experts have suggested that the two forms should be grouped together as ‘lobular neoplasia’ (in situ lobular neoplasia). However, for the purposes of recording risk of subsequent invasive carcinoma and for Cancer Registry recording, when the degree and extent can be assessed, for example in excision specimens, it is recommended that these are reported as separate entities. However, very mild forms of ALH can be found in association with fibrocystic change, involution and otherwise normal breast tissue.

As for ADH, assessment of extent and degree of acinar expansion by a lobular proliferation is required to distinguish atypical lobular hyperplasia (ALH) from lobular carcinoma in situ (LCIS). Even when LCIS has been diagnosed on core biopsy, the literature to date does not appear to indicate a different upgrade rate (risk of adjacent/associated invasive carcinoma or DCIS) and thus it is recommended that the term lobular neoplasia (encompassing both ALH and LCIS) is used in core biopsy specimens and a B3 category provided. The exceptions to this include pleomorphic

lobular carcinoma in situ (B5a) and mass-forming/florid LCICS (typically categorised as B4, suspicious).

In an excision specimen the pathologist should distinguish ALH from LCIS because of the reportedly different risk associated with subsequent carcinoma development.

In cytological terms, the two forms of the lesion are identical; in situ lobular neoplasia is characterised by proliferation within terminal duct lobular units of characteristic cells. The defining cell type is discohesive, round, cuboidal or polygonal with clear or light cytoplasm. Nuclei are small to moderate in size, round to oval and cytologically bland, with an occasional small inconspicuous nucleolus. The nucleus may be indented by an intracytoplasmic vacuole containing mucin. The cells have a high nuclear to cytoplasmic ratio. Mitotic figures and hyperchromatism are not often seen. There is an even distribution of cells and cellular monotony is the rule. Cytoplasmic clear vacuoles are often, although not invariably, present, sometimes having a central mucin blob. Pagetoid spread of cells may be present when the proliferation of neoplastic cells above the basement membrane undermines the normal lining epithelial cells.

The distension of lobular units may be variable from mild to gross, resulting in either patent lumina or complete obliteration. In atypical lobular hyperplasia there is minimal extension of less than half of the acini, whilst in lobular carcinoma in situ more than half of the acini within the TDLU are distended by an expansion of the typical cells (8 or more cells across each acinus).

Particularly in some more extensive lesions, distinction between in situ lobular neoplasia and DCIS may be difficult. For example, on occasions, a regular, evenly spaced monotonous population is seen within both ducts and lobules. E-cadherin membrane reactivity may be useful in such cases in distinguishing in situ lobular neoplasia from DCIS as the latter typically shows strong membrane positivity, whilst in the former expression is absent or weak. However, this marker can also be non-contributory or show varied levels within a case. Thus if both ducts and lobules contain epithelial proliferation of this type, particularly if E-cadherin is heterogeneous, categorisation as both LCIS and DCIS is recommended to imply the precursor risk of DCIS and the bilateral cancer risk of in situ lobular neoplasia. Such cases should also be recorded as DCIS **and** LCIS on the reporting form.

Pleomorphic Lobular Carcinoma *In Situ*

Variants, particularly the pleomorphic subtype of lobular carcinoma in situ, are recognised. The discohesion and architecture of the classical form is present but the cells are larger and

Clements K, *et al. BMJ Open* 2023; 12:e061585. doi: 10.1136/bmjopen-2022-061585

pleomorphic (cytonuclear grade 3) with more abundant cytoplasm. Central necrosis and calcification are often seen. Mitoses may be seen, although uncommon in the classical form, and atypical forms may be noted. Pleomorphic LCIS is less frequently ER positive than classical forms and more often expresses HER2 oncoprotein. There is a paucity of information available on the clinical behaviour of pleomorphic LCIS, but it is widely regarded as a more aggressive form of the disease, which should be managed akin to DCIS rather than classical LCIS, based on its biological and molecular profile. As noted above, as for the 'classical' form, E-cadherin membrane negativity may be useful in confirming that the lesion represents a lobular lesion, rather than high grade DCIS.

Extensive and Mass forming Classical LCIS with Necrosis

This rare variant of LCIS has classical cytology with central necrosis in distended acini. The degree of atypia is not sufficient for a diagnosis of pleomorphic LCIS. This variant is uncommon and the clinical behaviour is not well established.

In many centres both of the above entities are regarded as established forms of in situ carcinoma requiring therapeutic excision, equivalent to ductal carcinoma in situ (DCIS).

Size of Lesion

If the size of the lesion has been measured by the histopathologists (for example using the Vernier scale) this should be completed on the Sloane Audit form. As ADH is, by definition, a small microfocal lesion, recording the size will allow some analysis of diagnostic criteria adherence. It is not anticipated that pathologists will routinely record the size of classical (as opposed to pleomorphic) LISN or of FEA. The size of pleomorphic LCIS should be completed as, at present, this is generally clinically managed akin to DCIS and the same dataset is collected.

Margins

As, at present, pleomorphic LCIS is widely managed as per DCIS, the size of the lesion and the distance to margins should be recorded as for DCIS lesions.

If the pathologist has recorded that other atypical processes (FEA, ADH and classical LISN) extend to (or indeed has recorded the distance to) margins, this would be valuable information to collect and the Sloane Project Steering Committee would be

grateful to receive this data for subsequent follow-up analysis of clinical behaviour. It is not part of the routine NHSBSP minimum dataset, however, and we recognised that this is unlikely to be widely available/recorded.

Receptor Measurement

The methods used for receptor assay should follow UK national guidance. Laboratories performing such assays should participate in the relevant UK NEQAS scheme.

Lesions not included in the Sloane Atypia Audit

The above types of lesion are the only types of lesion that are being recorded in the Sloane Project Atypia Audit. We are no longer collecting data on DCIS, and have never recorded data on invasive disease. We are currently not collecting data on columnar cell change or columnar cell hyperplasia or usual epithelial hyperplasia.

Clements K, *et al. BMJ Open* 2023; 12:e061585. doi: 10.1136/bmjopen-2022-061585
