# Supplementary material for: Cohort profile of the Sloane Project: methodology for a prospective UK cohort study of >15 000 women with screen-detected non-invasive breast neoplasia
Source: BMJ Open. 2022 Dec 19;12(12):e061585. doi: 10.1136/bmjopen-2022-061585 (PMC9764674; doi:10.1136/bmjopen-2022-061585)
Supplement: Supplementary data [file bmjopen-2022-061585supp005.pdf]

Validation checks

strErrorText
- Check # 01 entries have been found where the SLOANE ID is '0' in the operation details table.
- Check # 02 patient(s) do not have matching radiology details.
- Check # 03 radiology record(s) do not have matching patient records.
- Check # 04 radiology record(s) do not have a pathology record.
- Check # 05 pathology record(s) do not have a radiology record.
- Check # 06 radiology record(s) do not have a treatment strategy record.
- Check # 07 treatment strategy record(s) do not have a radiology record.
- Check # 08 appointment record(s) do not have a radiology record.
- Check # 09 appointment record(s) do not have an operation record.
- Check # 10 operation record(s) do not have an appointment record.
- Check # 11 treatment strategy record(s) do not have a radiotherapy record.
- Check # 12 radiotherapy record(s) do not have a treatment strategy record.
- Check # 13 appointment(s) are potentially duplicate records (Visit ID).
- Check # 14 operation(s) are potentially duplicate records (Visit ID).
- Check # 15 pathology record(s) are potentially duplicates.
- Check # 16 QARC ID(s) are potentially duplicates (Patient table).
- Check # 17 SLOANE ID(s) are potentially duplicates (Radiology table).
- Check # 18 radiotherapy ID(s) are potentially duplicates.
- Check # 19 treatment strategy ID(s) are potentially duplicates.
- Check # 20 patients have NHS numbers that do not conform to the NHS standard.
- Check # 21 patient(s) with more than one appointment (operation) recorded for the same day (this is unusual and should be checked for accuracy).
- Check # 22 patient(s) with more than two mammograms recorded (this is unusual and should be checked for accuracy).
- Check # 23 patient(s) with more than two mammograms recorded for the same side (this is unusual and should be checked for accuracy).
- Check # 24 patient(s) with the date of mammogram not recorded.
- Check # 25 patient(s) whose adjuvant therapy details are not recorded.
- Check # 26 patient(s) have clear margins but details are not recorded.
- Check # 27 case(s) of DCIS with no Highest Nuclear Grade recorded.

Check # 28 case(s) with multiple cut up protocols recorded.
Check # 29 case(s) with multiple specimen X-Ray types recorded.
Check # 30 case(s) where a lesion was excised without one being present.
Check # 31 case(s) without microcalcification that have a suspicious pattern recorded.
Check # 32 case(s) where a lesion was excised without a specimen X-Ray having been performed.
Check # 33 case(s) with no specimen X-Ray performed, but Diagnostic or Theraputic has been recorded.
Check # 34 case(s) where a lesion was present in the specimen X-Ray without one having been performed.
Check # 35 case(s) where a boost was given, but treated daily is unrecorded.
Check # 36 case(s) where an external beam was given, but axilla treated is unrecorded.
Check # 37 case(s) where an external beam was given, but boost given is unrecorded.
Check # 38 case(s) where both an external beam and brachytherapy were given.
Check # 39 case(s) where an external beam was given, but treated daily is unrecorded.
Check # 40 case(s) where treated daily, but boost given is unrecorded.

Check # 41 case(s) where treated daily, but external beam is unrecorded.
Check # 42 case(s) where axillary nodes not taken, but procedures have been recorded.
Check # 43 case(s) where axillary nodes are taken, but procedure is unrecorded.
Check # 44 case(s) where no further surgical treatment undertaken and no reason has been recorded.
Check # 45 case(s) where further surgical treatment undertaken and reason(s) for not have been recorded.
Check # 46 case(s) where no further excision was carried out, but was orientated.
Check # 47 case(s) where a further excision was carried out without a specimen X-Ray.
Check # 48 case(s) where the lesion was confirmed to the surgeon without a specimen X-Ray having been done.
Check # 49 case(s) where a lesion was present in the specimen X-Ray without one having been performed.
Check # 50 case(s) where the patient entered clinical trials, but a trial name has not been recorded.
Check # 51 case(s) where the patient refused clinical trials, but a trial name has been recorded.
Check # 52 case(s) where a grade hasn't been recorded against a surgeon.
Check # 53 case(s) where a surgeon's name hasn't been recorded against a grade.
Check # 54 case(s) where the recorded specimen sizes do not match the total size of the lesion.
- Check # 57 (AgeAtMammogram)
- Check # 58 (BiLateral_CheckDates)
- Check # 59 (BreastProcedure_10_or_14)
- Check # 61 (DCIS_TotalSpecimenSizes)
- Check # 62 (DOB_MammoDate_OpDates)
- Check # 63 (DuplicateQARCIDs)
- Check # 64 (DuplicateQARCIDs_NotBiLateral)
- Check # 65 (ExcisionalOperatorsIncorrect)
- Check # 66 (ExcisionalValuesIncorrect)
- Check # 67 (ImmedReconMoreThan2)
- Check # 68 (ImmedReconNoMx)
- Check # 69 (MoreThanOnePrimary)
- Check # 70 (MultipleImmediateReconstruction)
- Check # 71 (MultipleMx)
- Check # 72 (OperationsFinalOpNo)
- Check # 73 (OperationsMoreThan4)
- Check # 74 (Path_BiopsyNoDetail)
- Check # 75 (Path_CheckMicroinvasionPresent)
- Check # 76 (Path_CheckNoOfFociMicroinvasion)
- Check # 77 (Path_CompletionMxOnlyOneOp)
- Check # 78 (Path_CompletionMxWithMx)
- Check # 79 (Path_DCISMicroinvasionNull)
- Check # 80 (Path_DCISMicroinvasionUnknown)
- Check # 81 (Path_DCISNoComedoNecrosis)
- Check # 82 (Path_DCISSizeIncorrect)
- Check # 83 (Path_MarginClear)
- Check # 84 (Path_MxMarginCheck)
- Check # 85 (Path_MxNoOperation)
- Check # 86 (Path_NodesCheckBlanks)

- Check # 87 (Path_NodesCheckValues)
- Check # 88 (Path_NodesOverall)
- Check # 89 (Path_NodesPositive)
- Check # 90 (Path_UnknownHistology)
- Check # 91 (Pathology_DCISNoNuclearGrade)
- Check # 92 (Pathology_MultipleCutUpProtocols)
- Check # 93 (PreOpDiagnosis)
- Check # 94 (Radiology_MicrocalcificationNotPresentWithNottmDef)
- Check # 95 (Radiology_MicrocalcificationPresentNoNottmDef)
- Check # 96 (Radiology_MultipleSpecimenXRays)
- Check # 97 (Radiology_NoLesionPresentLesionExcised)
- Check # 98 (Radiology_NoMicrocalcificationWithNottmDefinition)
- Check # 99 (Radiology_NoSpecimenXRayLesionExcised)
- Check # 100 (Radiology_NoSpecimenXRayWithDiagOrTheraputic)
- Check # 101 (Radiology_NoSpecimenXRayWithLesionPresent)
- Check # 102 (RadiologyBackgroundPattern)
- Check # 103 (RadiologyCheckLesionValues)
- Check # 104 (RadiologyCheckLesionValuesErrorsDiameter)
- Check # 105 (RadiologyCheckLesionValuesErrorsDistance)
- Check # 106 (RadiologyCheckLesionValuesErrorsLength)
- Check # 107 (RadiologyCheckLesionValuesErrorsVolume_2r)
- Check # 108 (RadiologyCheckLesionValuesErrorsVolume_h)
- Check # 109 (RadiologyPredominantRadiologicalFeature)
- Check # 110 (Radiotherapy_BoostGivenYesTreatedDailyNull)
- Check # 111 (Radiotherapy_ExternalBeamYesAxillaTreatedNull)
- Check # 112 (Radiotherapy_ExternalBeamYesBoostGivenNull)
- Check # 113 (Radiotherapy_ExternalBeamYesBrachytherapyYes)
- Check # 114 (Radiotherapy_ExternalBeamYesTreatedDailyNull)
- Check # 115 (Radiotherapy_NoBoostTreatedDaily)
- Check # 116 (Radiotherapy_NoExternalBeamTreatedDaily)
- Check # 117 (RadiotherapyUnknown)
- Check # 118 (RadiotherapyYesFormNotReceived)
- Check # 119 (RadiotherapyYesWithMx)
- Check # 120 (ScreeningRound0NullOrMoreThan8)
- Check # 121 (ScreeningYearOfDiagnosis)
- Check # 122 (SLOANEIDCheckFirstAndLast)
- Check # 123 (Treat_AdjuvantTherapyYes)
- Check # 124 (Treat_AxProcedures)
- Check # 125 (Treatment_AxillaryNodesNotTakenWithProcedures)
- Check # 126 (Treatment_AxillaryNodesTakenWithNoProcedures)
- Check # 127 (Treatment_FurtherSurgeryNoWithNoReason)
- Check # 128 (Treatment_FurtherSurgeryYesWithReason)
- Check # 129 (Treatment_NoFurtherExcisionButOrientated)
- Check # 130 (Treatment_NoOperationDate)
- Check # 131 (Treatment_NoSpecimenXRayFutherExcisionCarriedOut)
- Check # 132 (Treatment_NoSpecimenXRayLesionConfirmedToSurgeon)

- Check # 133 (Treatment_NoSpecimenXRayLesionPresent)
- Check # 134 (Treatment_PatientNotEnteredTrialWithTrialName)
- Check # 135 (Treatment_PatientRefusedTrialAndEntered)
- Check # 136 (Treatment_SurgeonNoGrade)
- Check # 137 (Treatment_SurgicalGradeNoSurgeon)
- Check # 138 (TreatStrat_AdjuvantTherapyUnknown)
- Check # 139 (TreatStrat_RadiotherapyWithAdjuvantTherapy)
- Check # 140 (TreatStrat_RefereForRadioYesNoAdjuventYes)
- Check # 141 (TreatStrat_ReferForRadioYesMxYes)
- Check # 142 (TreatStrat_ReferForRadioYesNoRadioForm)
- Check # 143 (AgeCheck)
- Check # 144 (OpAttendanceDatesNoOperation)
