# Supplementary material for: Cohort profile of the Sloane Project: methodology for a prospective UK cohort study of >15 000 women with screen-detected non-invasive breast neoplasia
Source: BMJ Open. 2022 Dec 19;12(12):e061585. doi: 10.1136/bmjopen-2022-061585 (PMC9764674; doi:10.1136/bmjopen-2022-061585)
Supplement: Supplementary data [file bmjopen-2022-061585supp006.pdf]

| Type of further event | Definition |
|---|---|
| Further event | (any of): ipsilateral within breast recurrence (or new primary) after BCS; ipsilateral recurrence (includes post-mastectomy/chest wall recurrence); regional or distant recurrence; or contralateral re/occurrence. |
| Ipsilateral recurrence | First recurrence (DCIS or invasive) or new primary (DCIS or invasive) in the same breast, post mastectomy chest wall, or ipsilateral axillary nodes, occurring 6 months or later after surgery for the index lesion. |
| Ipsilateral breast recurrence | first within breast recurrence (DCIS or invasive) or new primary (DCIS or invasive) in the same breast or post mastectomy chest wall occurring 6 months or later after surgery for the index lesion. |
| Distant recurrence | recurrence outwith the breast or post-mastectomy chest wall, (i.e. distant metastatic disease or regional nodal recurrence (ipsilateral or contralateral) in the axilla or supraclavicular fossa) occurring 6 months or later after surgery for the index breast lesion. |
| Contralateral event | new primary (DCIS or invasive) in the contralateral breast or contralateral axillary nodes, occurring 6 months or later after surgery for the index lesion in the other breast. |

**Supplemental Table 1: Definitions of further events in the Sloane Project cohort**
