# Supplementary material for: Cohort profile of the Sloane Project: methodology for a prospective UK cohort study of >15 000 women with screen-detected non-invasive breast neoplasia
Source: BMJ Open. 2022 Dec 19;12(12):e061585. doi: 10.1136/bmjopen-2022-061585 (PMC9764674; doi:10.1136/bmjopen-2022-061585)
Supplement: Supplementary data [file bmjopen-2022-061585supp007.pdf]

Cause of death	Definition
Breast cancer	Women were considered to have died from breast cancer if breast cancer appeared in the underlying cause of death column in the files available to, or in any section of part 1 (1a, 1b, or 1c) of the death certificate, or if metastases or carcinomatosis from breast cancer were recorded in part 1 of the death certificate, or if metastases or carcinomatosis were recorded in part 1 with breast cancer mentioned in part 2 and no other cancer identified. Women who died of breast cancer but who had no further events recorded were deemed to have had distant metastases on the date they died.
Other cancer	Women were considered to have an "other cancer" death if the underlying cause of death was cancer other than breast, or if one or more than one cancer at any site other than breast appeared in part 1 of the death certificate. Breast cancer may have appeared in part 2 or may not have appeared on the death certificate at all but it was not considered to be the underlying cause of death.
Cancer of unknown type	Women were considered to have died from an unknown type of cancer when they had metastatic cancer or carcinomatosis as the underlying cause of death, or if metastatic cancer or carcinomatosis was mentioned in part 1 of the death certificate but breast cancer or another type of cancer was not mentioned in any other section. Thus, cancer was known to be the cause of death, but the type of cancer was not known.
Non cancer (and non-circulatory)	Women were considered to have a non cancer (and non-circulatory) death if the underlying cause of death was not cancer and was not circulatory (circulatory was defined as any "I" ICD-10 code) or if the death was recorded as non cancer (and non-circulatory) in part 1 of the death certificate in the absence of cancer in part 1. If non cancer (and non-circulatory) cause of

	death was recorded in part 1 but cancer/or circulatory disease was noted in part 2, a check was done to see if it was likely the woman had died with, rather than because of, cancer/circulatory disease.
Non cancer (circulatory) deaths*	Women were considered to have a circulatory death if the underlying cause of death was a circulatory cause (defined as any "I" ICD-10 code) or a circulatory cause was recorded in part 1 of the death certificate in the absence of cancer in part 1. If cancer was identified in part 2, a check was done to ensure that it was clear that this could not considered to be the underlying cause.
Unknown cause of death	Unknown cause of death was recorded when the vital status and date of death had been reported but the cause of death had not been identified.

Supplemental Table 2: Definitions of cause of death in the Sloane Project

*Non-circulatory deaths category included because of the potential for radiotherapy-induced circulatory events
