# Supplementary material for: Cohort profile of the Sloane Project: methodology for a prospective UK cohort study of >15 000 women with screen-detected non-invasive breast neoplasia
Source: BMJ Open. 2022 Dec 19;12(12):e061585. doi: 10.1136/bmjopen-2022-061585 (PMC9764674; doi:10.1136/bmjopen-2022-061585)
Supplement: Author's manuscript [file bmjopen-2022-061585.draft_revisions.pdf]

BMJ Open is committed to open peer review. As part of this commitment we make the peer review history of every article we publish publicly available.

When an article is published we post the peer reviewers' comments and the authors' responses online. We also post the versions of the paper that were used during peer review. These are the versions that the peer review comments apply to.

The versions of the paper that follow are the versions that were submitted during the peer review process. They are not the versions of record or the final published versions. They should not be cited or distributed as the published version of this manuscript.

BMJ Open is an open access journal and the full, final, typeset and author-corrected version of record of the manuscript is available on our site with no access controls, subscription charges or pay-per-view fees (http://bmjopen.bmj.com).

If you have any questions on BMJ Open's open peer review process please email info.bmjopen@bmj.com

BMJ Open

# Cohort profile of the Sloane Project – methodology for a prospective UK cohort study of >15,000 women with screen-detected non-invasive breast neoplasia

| | |
|---|---|
| Journal: | *BMJ Open* |
| Manuscript ID | bmjopen-2022-061585 |
| Article Type: | Cohort profile |
| Date Submitted by the Author: | 02-Feb-2022 |
| Complete List of Authors: | Clements, Karen; NHS England and NHS Improvement Midlands, Screening Quality Assurance Service<br>Dodwell, David; University of Oxford, Nuffield Department of Population Health<br>Hilton, Bridget; NHS England and NHS Improvement Midlands, Screening QA Service<br>Stevens-Harris, Isabella; University Hospitals of Derby and Burton NHS Foundation Trust<br>Pinder, Sarah; Guy's & St Thomas' NHS Foundation Trust, Histopathology; King's College London Faculty of Life Sciences and Medicine, School of Cancer and Pharmaceutical Sciences<br>Wallis, M. G.; Cambridge Univ Hosp NHS Trust, Cambridge Breast Unit<br>Maxwell, AJ; Manchester University NHS Foundation Trust, Nightingale Centre<br>Kearins, Olive; NHS England and NHS Improvement Midlands, National Lead Breast Screening QA<br>Sibbering, Mark; University Hospitals of Derby and Burton NHS Foundation Trust<br>Shaaban, Abeer; Queen Elizabeth Hospital Birmingham and Univeristy of Birmingham, Histopathology<br>Kirwan, Cliona; The University of Manchester Faculty of Biology Medicine and Health, Division of Cancer Sciences<br>Sharma, Nisha; Leeds Teaching Hospitals NHS Trust, Breast Unit, St James's Hospital<br>Stobart, Hilary; Independent Cancer Patients' Voice<br>Dulson-Cox, Joanne; NHS England and NHS Improvement Midlands, Screening Quality Assurance Service<br>Litherland, Janet; West Scotland Breast Screening Centre<br>Mylvaganam, Senthurun; Royal Wolverhampton Hospitals NHS Trust<br>Provenzano, Elena; Cambridge University Hospitals NHS Foundation Trust<br>Sawyer, Elinor; King's College London Faculty of Life Sciences and Medicine, School of Cancer and Pharmaceutical Sciences<br>Thompson, Alastair M ; Baylor College of Medicine, Department of Surgical Oncology, Dan L Duncan Comprehensive Cancer Center, |
| Keywords: | AUDIT, Protocols & guidelines < HEALTH SERVICES ADMINISTRATION & MANAGEMENT, Breast tumours < ONCOLOGY, PUBLIC HEALTH, STATISTICS & RESEARCH METHODS |

SCHOLARONE™
Manuscripts

*I, the Submitting Author has the right to grant and does grant on behalf of all authors of the Work (as defined in the below author licence), an exclusive licence and/or a non-exclusive licence for contributions from authors who are: i) UK Crown employees; ii) where BMJ has agreed a CC-BY licence shall apply, and/or iii) in accordance with the terms applicable for US Federal Government officers or employees acting as part of their official duties; on a worldwide, perpetual, irrevocable, royalty-free basis to BMJ Publishing Group Ltd ("BMJ") its licensees and where the relevant Journal is co-owned by BMJ to the co-owners of the Journal, to publish the Work in this journal and any other BMJ products and to exploit all rights, as set out in our licence.*

*The Submitting Author accepts and understands that any supply made under these terms is made by BMJ to the Submitting Author unless you are acting as an employee on behalf of your employer or a postgraduate student of an affiliated institution which is paying any applicable article publishing charge ("APC") for Open Access articles. Where the Submitting Author wishes to make the Work available on an Open Access basis (and intends to pay the relevant APC), the terms of reuse of such Open Access shall be governed by a Creative Commons licence – details of these licences and which Creative Commons licence will apply to this Work are set out in our licence referred to above.*

*Other than as permitted in any relevant BMJ Author's Self Archiving Policies, I confirm this Work has not been accepted for publication elsewhere, is not being considered for publication elsewhere and does not duplicate material already published. I confirm all authors consent to publication of this Work and authorise the granting of this licence.*

For peer review only

# Cohort profile of the Sloane Project –methodology for a prospective UK cohort study of >15,000 women with screen-detected non-invasive breast neoplasia

Karen Clements, NHS England and NHS Improvement, 23 Stephenson Street, Birmingham, B2 4BH, UK

David Dodwell, Nuffield Department of Population Health, University of Oxford, Oxford, OX3 7LF, UK

Bridget Hilton, NHS England and NHS Improvement, 23 Stephenson Street, Birmingham, B2 4BH, UK

Isabella Stevens-Harris, University Hospitals of Derby & Burton NHS Foundation Trust, Derby DE22 3NE, UK

Sarah E Pinder, School of Cancer & Pharmaceutical Sciences, King's College London and Guy's and St Thomas' Hospitals NHS Foundation Trust, London, SE1 9RT, UK

Matthew G Wallis, Cambridge Breast Unit, Cambridge University Hospitals NHS Foundation Trust, Cambridge & NIHR Cambridge Biomedical Research Centre, Cambridge, CB2 0QQ, UK

Anthony Maxwell Nightingale Centre, Wythenshawe Hospital, Manchester University NHS Foundation Trust, Southmoor Road, Manchester, M23 9LT, UK

Olive Kearins, NHS England and NHS Improvement, 23 Stephenson Street, Birmingham, B2 4BH, UK

Mark Sibbering, University Hospitals of Derby & Burton NHS Foundation Trust, Derby, DE22 3NE, UK

Abeer M Shaaban, Queen Elizabeth Hospital Birmingham and University of Birmingham, Birmingham, B15 2GW, UK

Cliona Kirwan, Division of Cancer Sciences, Faculty of Biology, Medicine and Health, University of Manchester, Manchester, M13 9PT, UK



For peer review only - http://bmjopen.bmj.com/site/about/guidelines.xhtml

Nisha Sharma, Leeds Wakefield Breast Screening Service, Seacroft Hospital, York Road, Leeds, LS14 6UH, UK

Hilary Stobart, Independent Cancer Patients' Voice, 17 Woodbridge Street, London, EC1R 0LL, UK

Joanne Dulson-Cox, NHS England and NHS Improvement, 23 Stephenson Street, Birmingham, B2 4BH, UK

Janet Litherland, West of Scotland Breast Screening Centre, Nelson Mandela Place, Glasgow, G2 1QY, UK

Senthurun Mylvaganam, The Royal Wolverhampton Hospitals NHS Trust, New Cross Hospital, Wolverhampton Road, Wolverhampton, WV10 0QP, UK

Elena Provenzano, Cambridge University Hospitals NHS Foundation Trust, Cambridge & NIHR Cambridge Biomedical Research Centre, Cambridge, CB2 0QQ, UK

Elinor Sawyer, School of Cancer & Pharmaceutical Sciences, King's College London and Guy's and St Thomas' Hospitals NHS Foundation Trust, London, SE1 9RT, UK

Alastair M Thompson, Dan L Duncan Comprehensive Cancer Center, Baylor College of Medicine, Houston, Texas 77030, US

**Corresponding author**

Ms Karen Clements, Screening QA Service, NHS England and NHS Improvement, 2nd Floor, 23 Stephenson Street, Birmingham, B2 4BH, UK

Email: karen.clements@nhs.net



For peer review only - http://bmjopen.bmj.com/site/about/guidelines.xhtml

## ABSTRACT

### Purpose

The introduction of breast screening in the UK led to an increase in the detection of non-invasive breast neoplasia, predominantly ductal carcinoma in situ (DCIS), a non-obligatory precursor of invasive breast cancer. The Sloane Project, a UK prospective cohort study of screen-detected non-invasive breast neoplasia, commenced in 2003 to evaluate the radiological assessment, surgical management, pathology, adjuvant therapy and outcomes for non-invasive breast neoplasia. Long-term follow-up and accurate data collection are essential to examine the clinical impact. Here, we describe the establishment, development and analytic processes for this large UK cohort study.

### Participants

Women diagnosed with non-invasive breast neoplasia via the UK National Health Service Breast Screening Programme (NHSBSP) from 01 April 2003 are eligible, with a minimum age of 46 years. Demographic, diagnostic, therapeutic and follow-up data collected via proformas, complement date and cause of death from national data sources. Accrual for patients with DCIS ceased in 2012 but is ongoing for patients with epithelial atypia/in situ neoplasia, while follow-up for all patients continues long-term.

### Findings to date

To date, patients comprise a third of those diagnosed with DCIS within the NHSBSP during the relevant time period with the Sloane cohort representative of UK practice at the time of data collection. We have shown that DCIS is not the indolent disease often described and confirmed the need for longer term follow-up for screen detected DCIS. However, the radiology and pathology features of DCIS can be used to inform patient management. We demonstrate validation of follow-up information collected from national datasets against traditional manual methods.




For peer review only - http://bmjopen.bmj.com/site/about/guidelines.xhtml

**Future plans**

Conclusions derived from analyses of care and clinical outcomes from the Sloane Project are robust and generalisable to women in the UK with screen-detected DCIS. The follow-up methodology may be generalisable to other cohort studies and for clinical follow-up.

For peer review only



For peer review only - http://bmjopen.bmj.com/site/about/guidelines.xhtml

## ARTICLE SUMMARY

### Strengths and limitations of this study

- Large national prospective cohort study with high quality clinical data and standardised reporting
- Long-term follow-up data collection allows for the detection of subsequent cancers and analysis of outcome data
- Development of follow-up methodology using routinely collected data to identify further events could be generalised to other cohort studies, clinical trials, and cancer sites
- Coding in other linked datasets may be inaccurate leading to over-estimation of relative proportion of DCIS recurrences
- Participation was voluntary and 100% case capture was not achieved

For peer review only



For peer review only - http://bmjopen.bmj.com/site/about/guidelines.xhtml

## INTRODUCTION

The United Kingdom (UK) National Health Service Breast Screening Programme (NHSBSP) was established in 1988, originally to screen women aged 50-64 every three years using single view mammography. The NHSBSP now comprises individual programmes in each of the four devolved nations which have developed since the inception of UK-wide breast screening. Following the extension of the eligible age range to 70 years in England in 2002, Scotland in 2003-2004, Wales in 2006 and Northern Ireland in 2009, the NHSBSP now routinely invites all women in the UK from the age of 50 to their 71st birthday to attend breast screening every three years.[1] In some areas of the UK, women aged 47 to 49 and/or 71 to 73 received invitations for screening as part of an age extension trial[2] and some women received an invitation at the age of 46 as they were in their 47th year. Women from their 71st birthday onwards can self-refer every three years. Two-view mammography was introduced in Northern Ireland in 1989, Wales in 2001[3], England in 2003, and in Scotland in 2008/09 and is now used in all centres.[4] Analogue mammograms were replaced by digital mammography from 2008, with 99% of UK NHSBSP units able to perform digital mammography as of October 2013.[5] Digitisation of NHSBSP mammography was completed in 2015.

The introduction of breast screening in the UK led to an increase in the detection of non-invasive breast neoplasia, predominantly ductal carcinoma in situ (DCIS).[6] A final diagnosis of DCIS now accounts for 20% of screen-detected breast cancer [7] and 11% of all breast cancers diagnosed in women aged 50 years and older.[8] A diagnosis of DCIS poses a difficult problem for patients and clinicians since there remains uncertainty about the natural history, rate of progression, invasive potential, optimal treatment and follow-up protocols, which has led to concerns about overdiagnosis and overtreatment.[6]



For peer review only - http://bmjopen.bmj.com/site/about/guidelines.xhtml

Some DCIS progresses to invasive breast cancer, but the risk varies with features of DCIS.[6,9] An increased risk of both contemporaneous and subsequent invasive breast cancer is also seen in some other screen-detected lesions of uncertain malignant potential such as atypical intraductal epithelial proliferation (AIDEP)/atypical ductal hyperplasia (ADH), flat epithelial atypia (FEA) and lobular in situ neoplasia (LISN). However, prospective data on their management and outcomes are lacking, supporting the need for large, prospective studies, as data currently available are based on historical trial data[10,11,12] or retrospective studies.[13,14,15,16]

The Sloane Project, established in memory of Professor John Sloane, a breast pathologist,[17] commenced in 2003 as a UK-wide prospective cohort study of non-invasive breast neoplasia detected within the NHS Breast Screening Programme (NHSBSP). The original aim was to accrue a cohort of 10,000 women with screen-detected DCIS and subsequently to inform optimal radiological assessment, surgical management, pathology handling and reporting (including the features of greatest clinical and prognostic importance) and adjuvant therapy.

The potential value of this cohort study has been recognised as providing evidence for clinical practice by the National Institute of Clinical Excellence (NICE), who recommended that screen-detected DCIS patients be entered into the Sloane Project in the guideline "Diagnosis and treatment of early breast cancer, including locally advanced disease" published in February 2009.[18] More recently, the Independent Breast Screening Review[6] recommended "continued support for the Sloane Project which has an extensive database of screen-detected cases of DCIS and the long term follow-up of these cases may well improve our understanding of this condition".

This manuscript describes the Sloane Project process of case ascertainment, data cleaning and, specifically, methodology for verification and analysis of primary information and follow-up



For peer review only - http://bmjopen.bmj.com/site/about/guidelines.xhtml

data. The latter elements, in particular, may be widely applicable for analyses of other large national datasets.

## COHORT DESCRIPTION

The first phase of the Sloane Project collected data on women diagnosed with screen-detected non-invasive breast neoplasia from 01 April 2003 to 31 March 2012. The ongoing second phase is continuing to collect information on patients with epithelial atypia/in situ neoplasia, but excluding DCIS, and commenced on 01 April 2012. The collection of follow-up and mortality data is an ongoing process and continues for all Sloane Project patients. The minimum age for study entry was 46, with no maximum age.

### Eligibility

Eligibility for entry into the Sloane Project is indicated in Figure 1.

**Figure 1: Sloane eligibility flowchart**

### Case Ascertainment

The Sloane prospective cohort study is based on patient-level data collected by the NHS as part of the care and support of patients and is voluntarily submitted by UK NHSBSP units. Sloane Project cases were therefore matched to the data collected through the annual NHSBSP/ Association of Breast Surgery (ABS) breast screening audit[19] a national audit of all patients diagnosed through the NHSBSP, primarily designed to assess surgical performance with surgical Quality Assurance Standards, for the period 01 April 2003 to 31 March 2012, to understand the level of case ascertainment.

### Governance



For peer review only - http://bmjopen.bmj.com/site/about/guidelines.xhtml

The project is managed, and the data are collated, maintained and quality assured by the Screening Quality Assurance Service (SQAS), based in Birmingham, UK. It is overseen by a Project Steering Group (see Acknowledgements) comprising radiologists, pathologists, surgeons, managers, statisticians, oncologists, and patient advocates.

Ethics Committee approval was assessed as not required for this study which was originally conducted under the NHS Cancer Screening Programme's application to the Patient Information Advisory Group (PIAG). More recently, the study has been permitted to process personally identifiable data without consent under Regulation 5 of Statutory Instrument 2002 No. 1438: The Health Service (Control of Patient Information) Regulations 2002[20] (15/CAG/0207) in line with the following clause: “quality assuring screening services to ensure they are effective and safe, and that any incidents are investigated and managed appropriately”. This statutory exemption to common law permits the processing of personally identifiable data, as part of the core remit of population screening.

Data are securely held within the organisation with patient identifiers recorded. There is restricted access to a certified group of individuals and a full audit trail for access. A System Level Security Policy is in place to cover the Sloane Project as a System Asset as well as a Data Protection Impact Assessment. Figure 2 describes example data flows in and out of the Sloane Project, along with the controls in place to mitigate for risks.

**Figure 2: Example data flow diagram and key for Sloane Project data**

**Patient and Public involvement**

Patients from Independent Cancer Patients’ Voice (ICPV)[21] joined the Sloane Project steering group in 2012. Since this time, they have actively contributed to publications guidance,



For peer review only - http://bmjopen.bmj.com/site/about/guidelines.xhtml

communications, and promotional material, including the website, as well as supporting funding applications. They are also involved, as part of a wider international collaboration, with the research that has followed from the cohort study.

Clinical follow-up data from the English Programme are accessed via a Data Sharing Agreement (DSA) between the National Disease Registration Service (NDRS) and SQAS. Follow-up and mortality data from Scotland, Wales and Northern Ireland need specific permissions. The agreements (from these 'devolved nations') do not allow for further onward sharing of data at present. Apart from the limitation described above, access to the Sloane Project data from external parties is governed by application to the breast screening Research Advisory Committee (RAC) and Office for Data Release (ODR). Data will only be released by the Sloane Project to researchers under approval and in an anonymised or depersonalised format and under a data sharing agreement.

**Primary data capture**

Demographic data and data deriving from radiology, pathology, surgery and adjuvant treatments at the time of diagnosis and initial treatment were originally captured via individual, pathology, surgery and radiotherapy (RT) paper proformas (see Supplementary material), collected at NHSBSP unit and hospital level. More recently, the proformas have been shortened and combined into one form, which can be completed electronically.[22] The forms are submitted to the Sloane Project team and data are then entered on a secure database held on an SQL server that generates an individual patient and tumour identifier.

As all Sloane patients are diagnosed within the NHSBSP, adherence to NHSBSP guidelines[23] and participation in the relevant quality assurance programmes[24] are mandatory. Participating units are required to follow a pathology protocol[25] that includes guidance on the handling and



For peer review only - http://bmjopen.bmj.com/site/about/guidelines.xhtml

reporting of specimens, definitions for DCIS and atypia, microinvasion, cytonuclear grade, comedo necrosis and assessment of excision margins to NHSBSP pathology standards.[26] Radiology guidelines[27] encourage participating radiologists to complete the radiology proforma. Radiologists participate in the NHSBSP Personal Performance in Mammographic Screening (PERFORMS) external quality assurance scheme.[28]

**Data Handling**

Data are manually transcribed into the database, which has built in integrity checks run on a regular basis, along with extra validation checks for each subsequent data request. Missing data items are subsequently added as far as possible. If the data cannot be sourced from information already supplied or from the NHSBSP unit or other data sources, then unknown/unrecorded status is assigned. Best efforts are made to minimise the number of missing data items (see Supplementary material).

Validation of data is undertaken by cross-checking with original NHSBSP unit source documents for those patients with recurrence and more generally, for the overall dataset, against the Association of Breast Surgery (ABS) national audits.[19] The Sloane Project data, as completed by clinicians, are taken as the primary source when there is conflicting information. However, the ABS national audit is used to supplement missing data items.

**Classification of subsequent events**

Given the potential difficulties in distinguishing local recurrence from a new primary lesion in the same breast, terminology has been developed to classify subsequent events (Supplementary Table 1).



For peer review only - http://bmjopen.bmj.com/site/about/guidelines.xhtml

**Death classification**

Date and cause of death have historically been obtained directly from the NHS Trusts or Health Boards. Since 2013, the majority are now sourced from the National Disease Registration Service (NDRS)/NHS Wales Cancer Network Information System Cymru (CaNISC) data provided by the Office for National Statistics (ONS),[29] National Records of Scotland [30] and Northern Ireland patient records. Rules for coding the underlying cause of death are based on the rules used by ONS[29] that apply the condition or conditions entered in the lowest completed line of Part I of the Medical Certificate Cause of Death (MCCD). Women who die of breast cancer but who have no intervening breast events recorded are deemed to have had distant metastases on the date they died. The definitions for cause of death in Supplementary Table 2 are used for the Sloane dataset.

**Follow-up and recurrence data capture**

A methodology has been developed to identify further events, including ipsilateral breast/nodal, contralateral breast/nodal, and distant events. This is run annually for the English patients, who comprise 87.1% of the Sloane cohort, and on an *ad hoc* basis for those from Scotland, Wales and Northern Ireland, who make up 10.1%, 2.5% and 0.3% of patients respectively.

Further events are identified by matching women by their unique NHS number (in England and Wales) and Community Health Index (CHI) number (in Scotland and Northern Ireland) and information provided by NHSBSP units to routinely collected UK datasets, as detailed in Table 1.



For peer review only - http://bmjopen.bmj.com/site/about/guidelines.xhtml

| Country | Dataset utilised for Sloane follow-up data | Data collected | Data items used in Sloane English patient follow-up methodology |
|---|---|---|---|
| England (National Disease Registration Service incorporating the National Cancer Registration and Analysis Service - NCRAS) [31] | English Cancer Analysis System (CAS) | Cancer Registration data including diagnosis, treatment and tumour characteristics | date of diagnosis/treatment, trust/provider of diagnosis/treatment, ICD-10, morphology and behaviour, laterality, nodal positivity, adjuvant treatment, OPCS-4 |
| | Hospital Episode Statistics (HES) admitted patient care[32] | Diagnosis and treatment | date of admission/operation/episode start, ICD-10, OPCS-4, laterality, provider code |
| | Cancer Waiting Times (CWT)[33] | Diagnosis and treatment | date first seen/treatment start date, ICD-10, laterality, cancer treatment modality, cancer or symptomatic breast referral patient status, CTE type, and metastatic site |
| | English National Radiotherapy Dataset (RTDS)[34] (2009 onwards) | Adjuvant radiotherapy | treatment/appointment start date, treatment region, ICD-10 |
| | Systemic Anti-Cancer Therapy dataset (SACT) [35] | Adjuvant and neo-adjuvant chemotherapy | organisation code, ICD-10, morphology, drug treatment intent, start date, drug group |



For peer review only - http://bmjopen.bmj.com/site/about/guidelines.xhtml

| | | | |
|---|---|---|---|
| | Office for National Statistics (ONS) mortality data[29] | Date and Cause of death (diagnosed cancer patients) | |
| | Mortality and Birth Information System (MBIS) | Date and cause of death (diagnosed atypia patients) | |
| Scotland – Information Services Division of the Scottish government (ISD) and National Services Scotland (NSS) [36] | Cancer registration data | Diagnosis, treatment, tumour characteristics, date of death and cause of death | |
| Wales – Cancer Network Information System (CaNISC) [37] | Cancer registration data | Diagnosis, treatment, tumour characteristics, date of death and cause of death | |
| Northern Ireland | Sloane contact provides data from hospital records and systems on relatively small cohort of patients | Diagnosis, treatment, tumour characteristics, date of death and cause of death | |

**Table 1: National datasets provided to record follow-up and death information**



For peer review only - http://bmjopen.bmj.com/site/about/guidelines.xhtml

The current version of the International Classification of Diseases and Related Health Problems ICD-10[38] was used when ascertaining diagnostic codes alongside OPCS-4[39] when ascertaining procedure codes.

The censor date used in time-related analyses is the earliest of date of death, date of first further event (DCIS, invasive breast cancer, metastatic breast cancer) or end of time period of matched datasets if no further event has occurred and the woman is still alive.

**Follow-up methodology**

The follow-up methodology was developed to test the proof of concept of utilising the English NCRAS data to identify further events and decrease the burden of local data collection within NHSBSP units and NHS Trusts

The methodology is based on the following variables from the Sloane database:

- date of mammogram
- date of surgery
- laterality of primary lesion

These are then linked (via NHS number) to patients registered within the datasets and data items as described in Table 1.

**Validation of the follow-up methodology**

The methodology was validated on a subset of 3,711 (39%) of the cohort of Sloane patients for whom recurrence information was independently collected directly from the NHSBSP units prior to the generation of the methodology utilising NCRAS data from 2013. These data were compared with NCRAS data focussing on invasive status (invasive/non-invasive/distant), laterality and date of diagnosis for the primary and any further event.



For peer review only - http://bmjopen.bmj.com/site/about/guidelines.xhtml

To confirm a match of either primary or recurrence, data were considered to be:

- *acceptable* – same laterality, same invasive status, date of diagnosis within 6 months (either prior or after)
- *not acceptable* – different laterality, different invasive status (with the exception of microinvasion which is coded as invasive on CAS), dates >12 months apart
- *query acceptable* – e.g. dates different between 6 and 12 months; for example, in one case the diagnosis date and NCRAS chemotherapy date (the only event) differed by 8 months, which was considered to be acceptable.

In order to support this proof of concept project and provide further validation, we sent a list of 100 Sloane Project patients to University Hospitals of Derby and Burton NHS Foundation Trust (UHDB). Some of these patients we had identified as having a further event using the methodology. The Trust was blinded to our records for each patient and was requested to identify those who they believed to be patients with a further event, and provide date and type, but also to indicate those who had not had a further event according to their records.

## FINDINGS TO DATE

To date, several papers[9, 40,41,42,43,44,45,46,47,48,49,50,51] have been published on the Sloane cohort, which have shown that the radiology and pathology features of DCIS can be used to inform patient management. Outcome data also show that DCIS is not the indolent disease often described, and the need for longer term follow-up for screen detected DCIS has been confirmed.

### Case Ascertainment

Overall case ascertainment ranged from 0% to 93% by NHSBSP unit (median 32%, mean 38%). Individual attributes (including patient, tumour and treatment characteristics), such as age



For peer review only - http://bmjopen.bmj.com/site/about/guidelines.xhtml

at screening, cytonuclear grade of DCIS, DCIS size, Oestrogen Receptor (ER) status, surgical and adjuvant treatment have been shown to be representative when comparing the voluntary Sloane prospective cohort study with the compulsory ABS/NHSBSP annual screening audit, although the more detailed Sloane data represents 38% of patients with DCIS diagnosed through the NHSBSP over the same time period.

**Primary data completeness**

Data completeness, assessed against a pragmatic desired completeness level of ≥95%, demonstrated the majority of key data items for the DCIS patients within the Sloane Project database have excellent completeness levels. Data completeness was high for most data fields (Table 2).

| **Data item** | Data completeness of related field/s (%) of forms returned |
|---|---|
| **Patient details** | |
| Date of birth | 100.0 |
| NHS / CHI Number | 100.0 |
| Hospital Number | 96.0 |
| Screening Number | 99.6 |
| Hospital of diagnosis or treatment | 98.2 |
| Laterality of primary | 99.7 |
| **Radiology** | **97.9** |
| Site | 97.5 |
| Background Pattern | 97.5 |
| Predominant Radiological Feature | 98.1 |
| Presence/absence of microcalcification | 97.8 |
| Type of calcification | 99.0 |



For peer review only - http://bmjopen.bmj.com/site/about/guidelines.xhtml

| | |
|---|---|
| Radiological size | 98.1 |
| Breast volume | 96.4 |
| Specimen X-Ray | 83.8 |
| **Treatment** | **99.0** |
| Recorded presence/absence of pre-operative diagnosis of DCIS | 93.0 |
| Surgical procedure (or no surgery confirmed) | 99.0 |
| Date of surgical procedure (where relevant) | 99.8 |
| Axillary procedure done/not done | 97.1 |
| Axillary procedure type (where relevant) | 99.8 |
| Adjuvant therapy (whether adjuvant therapy given or not) | 99.2 |
| **Pathology** | **97.6** |
| Core/vacuum-assisted biopsy | 81.6 |
| Surgical specimen type | 96.2 |
| Final Histological diagnosis overall (from excision if excision done, from core if only core done) | 97.3 |
| Cytonuclear grade on surgical specimen (DCIS) | 99.2 |
| Architectural growth Pattern (DCIS) | 91.7 |
| Size (DCIS) | 98.4 |
| Presence/absence of Comedo Necrosis (DCIS) | 89.7 |
| Presence/absence of Microinvasion (DCIS) | 97.4 |
| Radial margin status (breast conserving surgery (BCS) + DCIS) | 94.1 |
| ER status (positive, negative or unknown [ER not done]) | 85.4 |
| PR status (included positive, negative or unknown [not done]) | 78.7 |
| HER2 status (included positive, negative or unknown [not done]) | 73.6 |
| **Radiotherapy (yes/no)** | **85.3** |
| Dose (where relevant) | 95.2 |
| Energy (where relevant) | 91.8 |
| Number of fractions (where relevant) | 94.5 |



For peer review only - http://bmjopen.bmj.com/site/about/guidelines.xhtml

**Table 2: Data completeness for key primary data items (DCIS patients)**

**Follow-up methodology validation results**

The results of the initial validation of the follow-up methodology, whereby recurrence (presence or absence of) information independently collected by Sloane was compared with routinely collected datasets from NCRAS are shown in Table 3 and 4.

| **NCRAS follow-up (using follow-up methodology)** | **Direct Sloane follow-up (n=3711)** | |
|---|---|---|
| | **No. of patients identified with further event in Sloane (n=211) (% of events identified from Sloane)** | **No. of patients without further event in Sloane (n=3500) (% of patients without further event from Sloane)** |
| **No. of patients identified with further event in NCRAS (n=301)** | 193 (91.5%) [AM] | 108 (3.1%) [NAM] |
| **No. of patients identified without further event in NCRAS (n=3377)** | 4 (1.9%) [NAM] | 3373 (96.4%) [AM] |
| **No. of patients identified with further event different between the datasets (n=18)** | 14 (6.6%) [NAM] | 4 (0.1%) [NAM] |
| **No. of patients not recorded on the NCRAS database (either primary or further event) (n=15)** | 0 (0%) [NAM] | 15 (0.4%) [NAM] |

[AM] = Acceptable match [NAM] = Not acceptable match

**Table 3: Validation of the follow-up methodology for identifying events / disease-free status (total number patients = 3711), comparing direct Sloane follow-up (211 patients with events identified) versus NCRAS follow-up (319 patients with events identified).**



For peer review only - http://bmjopen.bmj.com/site/about/guidelines.xhtml

Table 3 shows that of 3,711 Sloane DCIS patients for whom follow-up data was received direct from the NHSBSP units, 91.5% were also in the NCRAS datasets and were an 'acceptable match'. There was a 96.4% match between the Sloane and NCRAS datasets on fact of 'no further event'. 108 further events were identified through NCRAS datasets that had not been identified by the NHSBSP units. There are likely to be a number of reasons for this such as the patient's further event being diagnosed at a different NHS Trust or Health Board, or the type of hospital systems that were checked for further event and the likelihood of finding the information on those systems.

| **First Sloane further event** | **Number of Sloane patients identified with further event** | **Number matched to NCRAS datasets ('acceptable match')** |
|---|---|---|
| Ipsilateral non-invasive | 84 | 70 (83%) |
| Ipsilateral invasive | 56 | 54 (96%) |
| Ipsilateral distant (with invasive/non-invasive diagnosed at same time) | 2 | 2 (100%) |
| Distant | 6 | 5 (83%) |
| Contralateral non-invasive | 18 | 17 (94%) |
| Contralateral invasive | 42 | 42 (100%) |
| Contralateral invasive and distant (with invasive at same time) | 1 | 1 (100%) |
| Bilateral non-invasive | 2 | 2 (100%) |
| **Total** | **211** | **193 (91%)** |

**Table 4: Number of Sloane patients identified with a recurrence reported directly from NHSBSP units that matched/did not match to the same recurrence in the NCRAS datasets (when applying the Sloane follow-up methodology).**



For peer review only - http://bmjopen.bmj.com/site/about/guidelines.xhtml

Table 4 shows more detail about those 18 recurrences not matched according to our rules by NCRAS datasets. Of these, 11 were identified in HES but the invasive status was recorded incorrectly (9/11) or laterality was incorrect (2/11). Five were not identified in HES because two had surgery performed outside the NHS, one had no surgery due to co-morbidity, two remained unknown, although may have had their surgery in the private sector. Two were not found on CAS or HES but were identified on the CWT dataset, but invasive status was incorrect.

We concluded that although 92% events were successfully matched for further event correctly on NCRAS datasets, a further 13 recurrences (6%) would now be picked up by recent improvements in NCRAS and linkage to other datasets. It is likely that using currently available matching to national data sets, 206/211 patients (97%) would have accurate identification of DCIS or invasive breast cancer recurrence on the same side, contralateral breast or distant metastatic disease.

The further validation on 100 UHDB patients showed that, using the Sloane follow-up methodology, 20 matched exactly for further events, 75 matched as having no further events and five did not match. Thus 95% of patients were correctly matched. Out of the 5 that did not match 3 were because the Sloane methodology had a resulted in a “query further event” and UHDC confirmed “no further event”, 1 was “unknown invasive status” and UHDB confirmed “invasive” and 1 was recorded as “ipsilateral DCIS” whereas UHDB had it recorded as “ipsilateral invasive”. These results can be used to improve the methodology.

## STRENGTHS AND LIMITATIONS

### Strengths



For peer review only - http://bmjopen.bmj.com/site/about/guidelines.xhtml

Long term follow-up of patients is required to ensure the impact of diagnosis, treatment and subsequent care is optimised and changes in management evaluated. Large, prospective cohort studies of conditions such as DCIS where subsequent events are relatively rare and occur over decades present particular challenges. Even for smaller cohorts and especially for clinical trials, the benefits of accurately matching prospectively collected data to national data sets may be invaluable. The ambitious, prospective, long term Sloane Project has identified and followed some 15,000 patients with NHSBSP detected pre-invasive breast neoplasia over almost two decades. We demonstrate here a high level of concordance between subsequent disease events confirmed in the Sloane cohort data (provided directly by NHSBSP units) and those identified using a follow-up methodology applied to electronically stored national datasets. While events were rare, representing only 2% of the total cohort, concordance between data sources was strong. There was also a 96% level of concordance for freedom from further events. This is encouraging but requires future validation, before the use of NCRAS data to determine freedom from further events, can be regarded as definitive.

This methodology, if validated in other settings, and potentially further refined, may be generalisable to other cohort studies and for clinical trial follow-up. The long-term follow-up of patients beyond an initial 5 years is increasingly important in early invasive breast cancer, as well as DCIS, given the low frequency of events over an extended time period in these groups.

Although Sloane patients comprise a third of those diagnosed with DCIS within the NHSBSP during the relevant time period, the approach laid out here confirms that the data quality and completeness are high and appear to be representative of the overall population of patients with screen-detected DCIS in the UK. Thus, conclusions derived from analyses of care and clinical outcomes from the Sloane Project[9, 52,53,54,55,56,57,58,59,60,61,62,63] are robust and generalisable to all women in the UK with screen-detected DCIS. This is supported by a recently published study of over 35,000 women with screen-detected DCIS treated within the UK NHSBSP from its start



For peer review only - http://bmjopen.bmj.com/site/about/guidelines.xhtml

until 2014.[64] Patient, histopathology, treatment and outcomes reported reflect those curated within the more detailed Sloane cohort.

**Limitations**

Participation in data provision by NHSBSP units in the Sloane Project is voluntary, not specifically funded and 100% case capture was not achieved. However, comparison with the mandatory ABS/NHSBSP annual audits, which collect less detailed data, demonstrates that the Sloane dataset is representative of a cross section of patients, geographic spread and of NHSBSP units' practice at the time of accrual.

Engagement has been challenged by lack of resources, particularly NHS staff time, available for long-term for data collection. However, staff participants in the project receive CPD credits and certificates for their participation in primary and follow-up data capture which is useful for annual appraisal and revalidation. The Sloane Project DCIS database remains open for completion of data for those cases with missing information (and is currently over 94% complete).

Additional challenges include different health service structures and processes across England, Scotland, Wales and Northern Ireland. Data regarding the small proportion of patients treated outside the NHS are limited. Furthermore, coding of DCIS in some datasets may be inaccurate, resulting in a potential over-estimation of the relative proportion of DCIS that is recurring as invasive disease compared to that recurring as DCIS.

## COLLABORATION

The Sloane Project group welcomes applications from UK, EEA and international organisations to collaborate and release data. However, any data releases are subject to a common



For peer review only - http://bmjopen.bmj.com/site/about/guidelines.xhtml

governance framework (as summarised in Figure 2), which will ensure the correct confidentiality provisions, legal permissions and ethical approvals are adhered to.

## CONCLUSIONS

Overall, the Sloane prospective cohort study continues to evaluate changes in practice and outcomes for patients with DCIS as a result of publications of its own findings [50, 51] as well as others' research[65] and national guidelines and recommendations, in order to ascertain the impact of such variations. Extending the value of this prospective cohort study further, the data collected have been used to develop and support innovative research proposals examining the underlying biological and imaging features of DCIS.[66, 67] These peer-reviewed, grant-funded studies generated, for example, molecular data for comparison with the high quality clinical data from the Sloane Project obtained through the methodology described here to investigate mechanisms behind the progression of DCIS to invasive breast cancer. [68] Thus, the NHSBSP prospective cohort study of non-invasive breast neoplasia, particularly DCIS, (the Sloane Project)[22] continues to deliver insights into the clinical management of these conditions and resultant patient outcomes over time. Development of cross-validation of primary data, particularly patient outcomes, with the increasingly informative national datasets points to the generalisability of this approach.

**Word count (3505)**



For peer review only - http://bmjopen.bmj.com/site/about/guidelines.xhtml

**ACKNOWLEDGEMENTS**

This work uses patient data collected by the NHS as part of their care and support. The English data is collated, maintained and quality-assured by the Screening Quality Assurance Service (SQAS) and follow-up data by the National Cancer Registration and Analysis Service

**Sloane Project Steering Group**

Professor Alastair Thompson (Chair of Sloane Steering Group) – Professor of Surgery, Baylor College of Medicine, Houston, Texas

Mrs Samantha Brace-McDonnell - Patient Advocate, Independent Cancer Patients' Voice

Mrs Karen Clements - Breast Cancer Research Manager, NHS England and NHS Improvement

Professor Sarah Darby - Professor of Medical Statistics, University of Oxford

Dr David Dodwell – Consultant Clinical Oncologist, University of Oxford, Oxford

Ms Joanne Dulson-Cox - National Audit Project Senior QA Officer, NHS England and NHS Improvement

Mrs Bridget Hilton - National Audit Project Senior QA Officer, NHS England and NHS Improvement

Mrs Olive Kearins – Quality Assurance Director, NHS England and NHS Improvement

Miss Cliona Kirwan - NIHR Clinician Scientist in Surgical Oncology and Consultant Oncoplastic Breast Surgeon, University Hospital of South Manchester

Dr Janet Litherland - Consultant Radiologist, West of Scotland Breast Screening Programme, Glasgow

Dr Anthony Maxwell - Consultant Radiologist, University Hospital of South Manchester, Manchester

Mr Seni Mylvaganam - Consultant Oncoplastic Breast Surgeon, Royal Wolverhampton Hospital

Professor Sarah Pinder - Professor of Breast Pathology, Guys and St Thomas' Hospitals, London

Dr Elena Provenzano - Lead Breast Histopathologist, Addenbrooke's Hospital, Cambridge

Professor Elinor Sawyer – Consultant Clinical Oncologist, Guys Hospital, London

Dr Abeer Shaaban - Consultant Pathologist, University Hospitals Birmingham

Dr Nisha Sharma - Lead Clinician Radiology, Leeds Teaching Hospital NHS Trust

Ms Hilary Stobart - Patient Advocate, Independent Cancer Patients' Voice

Dr Matthew Wallis - Consultant Radiologist, Addenbrooke's Hospital, Cambridge



For peer review only - http://bmjopen.bmj.com/site/about/guidelines.xhtml

We are also grateful for the help, support and professional advice provided by previous members of the Sloane Project Steering Group - Mr Hugh Bishop (previous Chair), Dr Julian Adlard, Miss Jessamy Bagenal, Mr Robert Carpenter, Professor John Dewar, Professor Ian Ellis, Professor Adele Francis, Professor W D George, Professor Andrew Hanby, Professor Sunil Lakhani, Dr Gill Lawrence, Mr Martin Lee, Dr James Macartney, Mr Stewart Nicholson, Professor Julietta Patnick CBE, Dr Gillian Ross, Mr Mark Sibbering, Professor Valerie Speirs, Ms Sheila Stallard, Dr Jeremy Thomas, Professor Ian Tomlinson, Mrs Margot Wheaton, Ms Maggie Wilcox.

**All participating UK NHSBSP units**

Avon
Aylesbury and Wycombe
Barnsley
Bedfordshire and Hertfordshire
Bolton
Breast Test Wales – North
Breast Test Wales – South East
Breast Test Wales – South West
Brighton
Cambridge and Huntingdon
Central & East London
Chelmsford and Colchester
Chester
City, Sandwell and Walsall
Cornwall
Crewe
Derby City and South Derbyshire
Doncaster
Dorset
Dudley and Wolverhampton
East Berkshire
East Cheshire & Stockport
East Lancashire

For peer review only

East Scotland

Gateshead

Gloucestershire

Great Yarmouth and Waveney

Greater Manchester

Guildford

Hereford and Worcester

Humberside

Isle of Wight

King's Lynn

Leeds and Wakefield

Leicestershire

Liverpool

Medway

Milton Keynes

Newcastle upon Tyne

Norfolk and Norwich

North and Eastern Devon

North and Mid Hampshire

North Derbyshire & Chesterfield

North East Scotland

North Lancs & South Cumbria

North London

North Midlands

North Nottingham

North Yorkshire

Northampton

Nottingham

Outer North East London

Oxford

Pennine

Peterborough

Portsmouth

Rotherham

For peer review only



For peer review only - http://bmjopen.bmj.com/site/about/guidelines.xhtml

Sheffield

Shropshire

Somerset

South Birmingham

South East London

South East Scotland

South Essex

South West London

South West Scotland

Southampton and Salisbury

Surrey (Jarvis)

Warrington

Warwick, Solihull and Coventry

West Berkshire

West Devon

West Essex

West of London

West of Scotland

West Suffolk

Western BSU, Altnagelvin Hospital, Northern Ireland

Wiltshire

Wirral

Worthing (West Sussex)

**Public Health Scotland (previously the Information Services Division Scotland)**

**NHS Wales Cancer Network Information System Cymru (Canisc)**

**Independent Cancer Patients' Voice**

**Funders (as described below)**

**COMPETING INTERESTS**



For peer review only - http://bmjopen.bmj.com/site/about/guidelines.xhtml

KC is currently funded (since 2019) as part of the Cancer Research Grand Challenge PRECISION team (C7827/A27366), which is funded by Cancer Research UK and the Dutch Cancer Society.
DD is funded by Cancer Research UK (grant C8225/A21133)
EP received speaker's honoraria (Roche), Travel costs to speak at meeting (Roche)
Participation in Advisory group meetings for IPB Advisors and Roche
SEP participated in Advisory Boards for AstraZeneca, Roche and Exact Sciences, received money for speaking at meetings for Roche and Exact Sciences and is a member of the PRECISION consortium (C7827/A27366).
AS has participated in Advisory Boards for Exact Sciences and Veracyte
HS received travel and support to attend meetings of the CRUK Grand Challenge PRECISION Study (C7827/A27366).
ES, AMT, MGW are members of the PRECISION consortium (C7827/A27366).
BH, ISH, AM, OK, MS, CK, NS, JDC, JL, SM, have nothing to declare.

## CONSENT FOR PUBLICATION

Not applicable

## AVAILABILITY OF DATA AND MATERIALS

No additional data available

## TRANSPARENCY DECLARATION

The lead author* affirms that this manuscript is an honest, accurate, and transparent account of the study being reported; that no important aspects of the study have been omitted; and that any discrepancies from the study as planned (and, if relevant, registered) have been explained.

## AUTHOR CONTRIBUTIONS:

All authors contributed either to the conception or design of the work; or the acquisition, analysis, or interpretation of data for the work. KC, DD and AT drafted the manuscript. All authors assisted with the draft and reviewed/revised it critically and agreed the final version of the manuscript. The corresponding author confirms that all listed authors meet authorship criteria and that others meeting the criteria are not included. All authors have approved the final version of this article and agree to be accountable for all aspects of the work.



For peer review only - http://bmjopen.bmj.com/site/about/guidelines.xhtml

**FUNDING**

At the time of writing, the Sloane Project work is undertaken within the Screening QA Service at NHS England and NHS Improvement and the main author is funded as part of the Cancer Research Grand Challenge PRECISION team (C7827/A27366) .

The Sloane Project has received previous funding from the following:

NHS Breast Screening Programme (via Public Health England April 2013 to September 2021, previously NHS)

Breast Cancer Research Trust (2-year project grant 2008-2009)

Pfizer (3-year unrestricted educational grant 2005-2007)

Ad-hoc fundraising events and private donations including Golf Charity Days (organised by Ken Ward), pub quizzes and fundraising balls (2005-2012)

The funders of the Sloane Project have not had any involvement in the study design; in the collection, analysis, and interpretation of data; in the writing of the report; or in the decision to submit the article for publication.



For peer review only - http://bmjopen.bmj.com/site/about/guidelines.xhtml

## REFERENCES


[1]NHS https://www.nhs.uk/conditions/breast-cancer-screening/when-its-offered/ March 2018. (accessed 20 September 2021)

[2]Age X Trial http://www.agex.uk/about Oxford, 2020 (accessed 20 September 2021)

[3] Osborn GD, Beer H, Wade R, et al. Two-view mammography at the incident round has improved the rate of screen-detected breast cancer in Wales. Clinical Radiology. 2006;61: 478e482

[4]. Dibden A, Offman J, Parmar D, et al. Reduction in interval cancer rates following the introduction of two-view mammography in the UK breast screening programme. British Journal of Cancer. 2013;110, 560-564

[5] The National Archives. http://webarchive.nationalarchives.gov.uk/20150506150512/http://cancerscreening.nhs.uk/breastscreen/digital-mammography.html May 2015 (accessed 20 September 2021)

[6] Independent UK Panel on Breast Cancer Screening. The benefits and harms of breast cancer screening: an independent review. Lancet. 2012;380(9855):1778-86.

[7] NHS Breast Screening Programme & Association of Breast Surgery. An audit of screen detected breast cancers for the year of screening April 2018 to March 2019, May 2021.

[8] Healthcare Quality Improvement Partnership (HQIP) National Audit of Breast Cancer in Older Patients (NABCOP): 2020 annual report. https://www.nabcop.org.uk/reports/nabcop-2020-annual-report/; 2020.

[9] Maxwell AJ, Clements K, Hilton B, et al; Sloane Project Steering Group. Risk factors for the development of invasive cancer in unresected ductal carcinoma in situ. Eur J Surg Oncol. 2018 Apr;44(4):429-435. doi: 10.1016/j.ejso.2017.12.007.

[10] Donker M, Litie`re S, Werutsky G, et al. Breast-conserving treatment with or without radiotherapy in ductal carcinoma in situ: 15-year recurrence rates and outcome after a




For peer review only - http://bmjopen.bmj.com/site/about/guidelines.xhtml

recurrence, from the EORTC 10853 randomized phase III trial. J Clin Oncol 2013;31(32):4054e9.

[11] Narod SA, Iqbal J, Giannakeas V, et al. Breast cancer mortality after a diagnosis of ductal carcinoma in situ. JAMA Oncol 2015;1(7):888e96.

[12] Early Breast Cancer Trialists' Collaborative Group EBCTCG. Overview of the randomized trials of radiotherapy in ductal carcinoma in situ of the breast. J Natl Cancer Inst Monogr 2010;2010(41):162e77.

[13] Sagara Y, Mallory MA, Wong S, et al. Survival benefit of breast surgery for low-grade ductal carcinoma in situ: a population based cohort study. JAMA Surg 2015;150(8):739e45.

[14] Elshof LE, Schaapveld M, Schmidt MK, et al. Subsequent risk of ipsilateral and contralateral invasive breast cancer after treatment for ductal carcinoma in situ: incidence and the effect of radiotherapy in a population-based cohort of 10,090 women. Breast Cancer Res and Treat 2016;159(3):553e63.

[15] Zhou WB, Xue DQ, Liu XA, et al. The influence of family history and histological stratification on breast cancer risk in women with benign breast disease: a meta-analysis. Journal of cancer research and clinical oncology. 2011; 137(7):1053–60. [PubMed: 21499874]

[16] Dyrstad SW, Yan Y, Fowler AM, et al. Breast cancer risk associated with benign breast disease: systematic review and meta-analysis Breast Cancer Res Treat (2015) 149:569–575

[17] Rudland P, Gusterson B. John Sloane – Eminent pathologist who pioneered breast cancer treatment. https://www.theguardian.com/news/2000/may/26/guardianobituaries2 The Guardian, May 2000 (accessed 28 February 2020)

[18] Harnett A, Smallwood J, Titshall V, et al. on behalf of the Guideline Development Group. Diagnosis and treatment of early breast cancer, including locally advanced disease—summary of NICE guidance. BMJ 2009; 338:b438 doi: https://doi.org/10.1136/bmj.b438



For peer review only - http://bmjopen.bmj.com/site/about/guidelines.xhtml

[19] Association of Breast Surgery: NHSBSP ABS Audit of Screen Detected Breast Cancer https://associationofbreastsurgery.org.uk/professionals/audit/nhs-breast-screening-programme-audit/ (accessed 17 September 2021)

[20] NHS, England and Wales. The Health Service (Control of Patient Information) Regulations 2002. http://www.legislation.gov.uk/uksi/2002/1438/contents/made (accessed 30 September 2021)

[21] Independent Cancer Patients' Voice http://www.independentcancerpatientsvoice.org.uk/ (accessed 17 September 2021)

[22] Government Digital Service. www.gov.uk/phe/sloane-project (accessed 30 September 2021)

[23] Government Digital Service. https://www.gov.uk/government/collections/breast-screening-professional-guidance September 2020 (accessed 30 September 2021)

[24] Government Digital Service. https://www.gov.uk/government/publications/breast-screening-programme-specific-operating-model August 2021 (accessed 20 September 2021)

[25] The National Archives https://webarchive.nationalarchives.gov.uk/20180903111938/http://www.sloaneproject.co.uk/Pathology.htm September 2018 (accessed 20 September 2021)

[26] The Royal College of Pathologists. Pathology reporting of breast disease in surgical excision specimens incorporating the dataset for histological reporting of breast cancer. www.rcpath.org June 2016

[27] The National Archives https://webarchive.nationalarchives.gov.uk/ukgwa/20180903111940/http://www.sloaneproject.co.uk/Radiology.htm September 2018 (accessed 30 September 2021)

[28] Gale AG. PERFORMS: a self-assessment scheme for radiologists in breast screening. Semin Breast Dis 2003 Sep 30;6(3):148e52.WB Saunders.



For peer review only - http://bmjopen.bmj.com/site/about/guidelines.xhtml

[29] Office for National Statistics: User guide to mortality statistics https://www.ons.gov.uk/peoplepopulationandcommunity/birthsdeathsandmarriages/deaths/methodologies/userguidetomortalitystatisticsjuly2017 Latest revision July 2021 (accessed 08 October 2021)

[30] National Records of Scotland: Death Certificates and Coding the Cause of Death https://www.nrscotland.gov.uk/statistics-and-data/statistics/statistics-by-theme/vital-events/deaths/deaths-background-information/death-certificates-and-coding-the-causes-of-death August 2017 (accessed 08 October 2021)

[31] National Disease Registration Service https://www.ndrs.nhs.uk/resources/a-guide-to-cancer-data-and-working-with-us/ (accessed 08 October 2021)

[32] NHS Digital: About the HES database https://digital.nhs.uk/data-and-information/data-tools-and-services/data-services/hospital-episode-statistics (accessed 22 January 2021)

[33] NHS Digital Cancer Waiting Times data collection https://digital.nhs.uk/data-and-information/data-collections-and-data-sets/data-collections/cancerwaitingtimescwt (accessed 08 October 2021)

[34] NHS Digital DAPB0111: Radiotherapy Data Set https://digital.nhs.uk/data-and-information/information-standards/information-standards-and-data-collections-including-extractions/publications-and-notifications/standards-and-collections/dapb0111-radiotherapy-data-set (accessed 08 October 2021)

[35] SACT Systemic Anti-Cancer Therapy Dataset http://www.chemodataset.nhs.uk/home (accessed 22 January 2021)

[36] Public Health Scotland: Scottish Cancer Registry https://www.isdscotland.org/Health-Topics/Cancer/Scottish-Cancer-Registry/ (accessed 22 January 2021)

[37] NHS Wales Informatics Service: Information Services https://nwis.nhs.wales/information-services/ (accessed 22 January 2021)


For peer review only



For peer review only - http://bmjopen.bmj.com/site/about/guidelines.xhtml

[38] World Health Organisation: International Statistical Classification of Disease and Related Health Problems (ICD) https://www.who.int/classifications/classification-of-diseases (accessed 22 January 2021)

[39] NHS Digital: NHS Data Model and Dictionary https://datadictionary.nhs.uk/supporting_information/opcs_classification_of_interventions_and_procedures.html?hl=opcs (accessed 22 January 2021)

[40] Evans A, Clements K, Maxwell A, et al. Mammographic bi-dimensional product: a powerful predictor of successful excision of ductal carcinoma in situ. Clinical Radiology 2007; 62: 787-791

[41] Dodwell D, Clements K, Lawrence G, et al. Radiotherapy following breast-conserving surgery for screen-detected ductal carcinoma in situ (DCIS): Indications and utilisation in the UK: Findings from the Sloane Project. British Journal of Cancer 2007; 97: 725-729

[42] Thomas J, Hanby A, Pinder S, et al. Implications of Inconsistent Measurement of ER Status in Non-Invasive Breast Cancer: A Study of 1,684 Cases from the Sloane Project The Breast Journal Volume 14, Number 1, January/February 2008, pp. 33-38(6)

[43] Maxwell A J, Evans A J, Carpenter R, et al. Follow-up for screen-detected ductal carcinoma in situ: Results of a survey of UK centres participating in the Sloane Project. European Journal of Surgical Oncology (EJSO) 2009 35 (10) 1055-1059

[44] Thomas J, Evans A, Macartney J, et al on behalf of the Sloane Project Steering Group. Radiological and pathological size estimations of pure ductal carcinoma in situ of the breast, specimen handling and the influence on the success of breast conservation surgery: a review of 2564 cases from the Sloane Project. British Journal of Cancer (2010) 102, 285 – 293

[45] Evans A, Clements K, Maxwell A, et al on behalf of the Sloane Project Steering Group. Lesion size is a major determinant of the mammographic features of ductal carcinoma in situ: findings from the Sloane project. Clinical Radiology 2010 Volume 65, Issue 3, Pages 181-184



For peer review only - http://bmjopen.bmj.com/site/about/guidelines.xhtml

46 Thomas J, Hanby A M, Pinder S E, et al. Adverse surgical outcomes in screen-detected ductal carcinoma in situ of the breast. European Journal of Cancer (2014) 50, 1880– 1890

47 Nicholson S, Hanby A, Clements K, et al, on behalf of the Sloane Project Steering Group. Variations in the management of the axilla in screen-detected Ductal Carcinoma In Situ: Evidence from the UK NHS Breast Screening Programme audit of screen detected DCIS. European Journal of Surgical Oncology 2015; Jan;41(1):86-93. doi: 10.1016/j.ejso.2014.09.003.

48 Clements K, Dodwell D, Lawrence G, et al, on behalf of the Sloane Project Steering Group. Radiotherapy after mastectomy for screen-detected ductal carcinoma in situ. European Journal of Surgical Oncology (EJSO) Volume 41, Issue 10, October 2015, Pages 1406-1410 https://doi.org/10.1016/j.ejso.2015.07.021

49 Maxwell AJ, Clements K, Dodwell DJ, et al, on behalf of the Sloane Project Steering Group. The radiological features, diagnosis and management of screen-detected lobular neoplasia of the breast: Findings from the Sloane Project. The Breast 27 (2016) 109-115

50 Thompson AM, Clements K, Cheung S, et al. Management and 5-year outcomes in 9938 women with screen-detected ductal carcinoma in situ: the UK Sloane Project. European Journal of Cancer. 2018;101:210-9.

51 Shaaban A, Hilton B, Clements K, et al, on behalf of the Sloane Project Steering Committee. Pathological features of 11337 patients with primary ductal carcinoma in situ (DCIS) and subsequent events – results from the UK Sloane Project. British Journal of Cancer. 2020 https://doi.org/10.1038/s41416-020-01152-5

52 Evans A, Clements K, Maxwell A, et al. Mammographic bi-dimensional product: a powerful predictor of successful excision of ductal carcinoma in situ. Clinical Radiology 2007; 62: 787-791

53 Dodwell D, Clements K, Lawrence G, et al. Radiotherapy following breast-conserving surgery for screen-detected ductal carcinoma in situ (DCIS): Indications and utilisation in the UK: Findings from the Sloane Project. British Journal of Cancer 2007; 97: 725-729



For peer review only - http://bmjopen.bmj.com/site/about/guidelines.xhtml

[54] Thomas J, Hanby A, Pinder S, et al. Implications of Inconsistent Measurement of ER Status in Non-Invasive Breast Cancer: A Study of 1,684 Cases from the Sloane Project The Breast Journal Volume 14, Number 1, January/February 2008, pp. 33-38(6)

[55] Maxwell A J, Evans A J, Carpenter R, et al. Follow-up for screen-detected ductal carcinoma in situ: Results of a survey of UK centres participating in the Sloane Project. European Journal of Surgical Oncology (EJSO) 2009 35 (10) 1055-1059

[56] Thomas J, Evans A, Macartney J, et al on behalf of the Sloane Project Steering Group. Radiological and pathological size estimations of pure ductal carcinoma in situ of the breast, specimen handling and the influence on the success of breast conservation surgery: a review of 2564 cases from the Sloane Project. British Journal of Cancer (2010) 102, 285 – 293

[57] Evans A, Clements K, Maxwell A, et al on behalf of the Sloane Project Steering Group. Lesion size is a major determinant of the mammographic features of ductal carcinoma in situ: findings from the Sloane project. Clinical Radiology 2010 Volume 65, Issue 3, Pages 181-184

[58] Thomas J, Hanby A M, Pinder S E, et al. Adverse surgical outcomes in screen-detected ductal carcinoma in situ of the breast. European Journal of Cancer (2014) 50, 1880– 1890

[59] Nicholson S, Hanby A, Clements K, et al, on behalf of the Sloane Project Steering Group. Variations in the management of the axilla in screen-detected Ductal Carcinoma In Situ: Evidence from the UK NHS Breast Screening Programme audit of screen detected DCIS. European Journal of Surgical Oncology 2015; Jan;41(1):86-93. doi: 10.1016/j.ejso.2014.09.003.

[60] Clements K, Dodwell D, Lawrence G, et al, on behalf of the Sloane Project Steering Group. Radiotherapy after mastectomy for screen-detected ductal carcinoma in situ. European Journal of Surgical Oncology (EJSO) Volume 41, Issue 10, October 2015, Pages 1406-1410 https://doi.org/10.1016/j.ejso.2015.07.021



For peer review only - http://bmjopen.bmj.com/site/about/guidelines.xhtml

[61] Maxwell AJ, Clements K, Dodwell DJ, et al, on behalf of the Sloane Project Steering Group. The radiological features, diagnosis and management of screen-detected lobular neoplasia of the breast: Findings from the Sloane Project. The Breast 27 (2016) 109-115

[62] Thompson AM, Clements K, Cheung S, et al. Management and 5-year outcomes in 9938 women with screen-detected ductal carcinoma in situ: the UK Sloane Project. European Journal of Cancer. 2018;101:210-9.

[63] Shaaban A, Hilton B, Clements K, et al, on behalf of the Sloane Project Steering Committee. Pathological features of 11337 patients with primary ductal carcinoma in situ (DCIS) and subsequent events – results from the UK Sloane Project. British Journal of Cancer. 2020 https://doi.org/10.1038/s41416-020-01152-5

[64] Mannu GS, Wang Z, Broggio J, et al. Invasive breast cancer and breast cancer mortality after ductal carcinoma in situ in women attending for breast screening in England, 1988-2014: population based observational cohort study. BMJ. 2020 May 27;369.

[65] Dodwell D, Jauhari Y, Gathani T, et al. Treatment variation in early breast cancer in the UK. BMJ 2020; 371:m4237 doi: https://doi.org/10.1136/bmj.b438

[66] Megalios A, Shah V, Shami R, et al. Abstract P4-06-05: Heterogeneity of DCIS does not appear to be a biomarker of subsequent invasive cancer. American Association for Cancer Research 2020. Volume 80, Issue 4 Supplement, pp. P4-06-05

[67] https://www.dcisprecision.org/ (Accessed 20 September 2021)

[68] Lips EH, Kumar T, Megalios A, et al. Genomic profiling defines variable clonal relatedness between invasive breast cancer and primary ductal carcinoma in situ medRxiv 2021.03.22.21253209; doi: https://doi.org/10.1101/2021.03.22.21253209



For peer review only - http://bmjopen.bmj.com/site/about/guidelines.xhtml

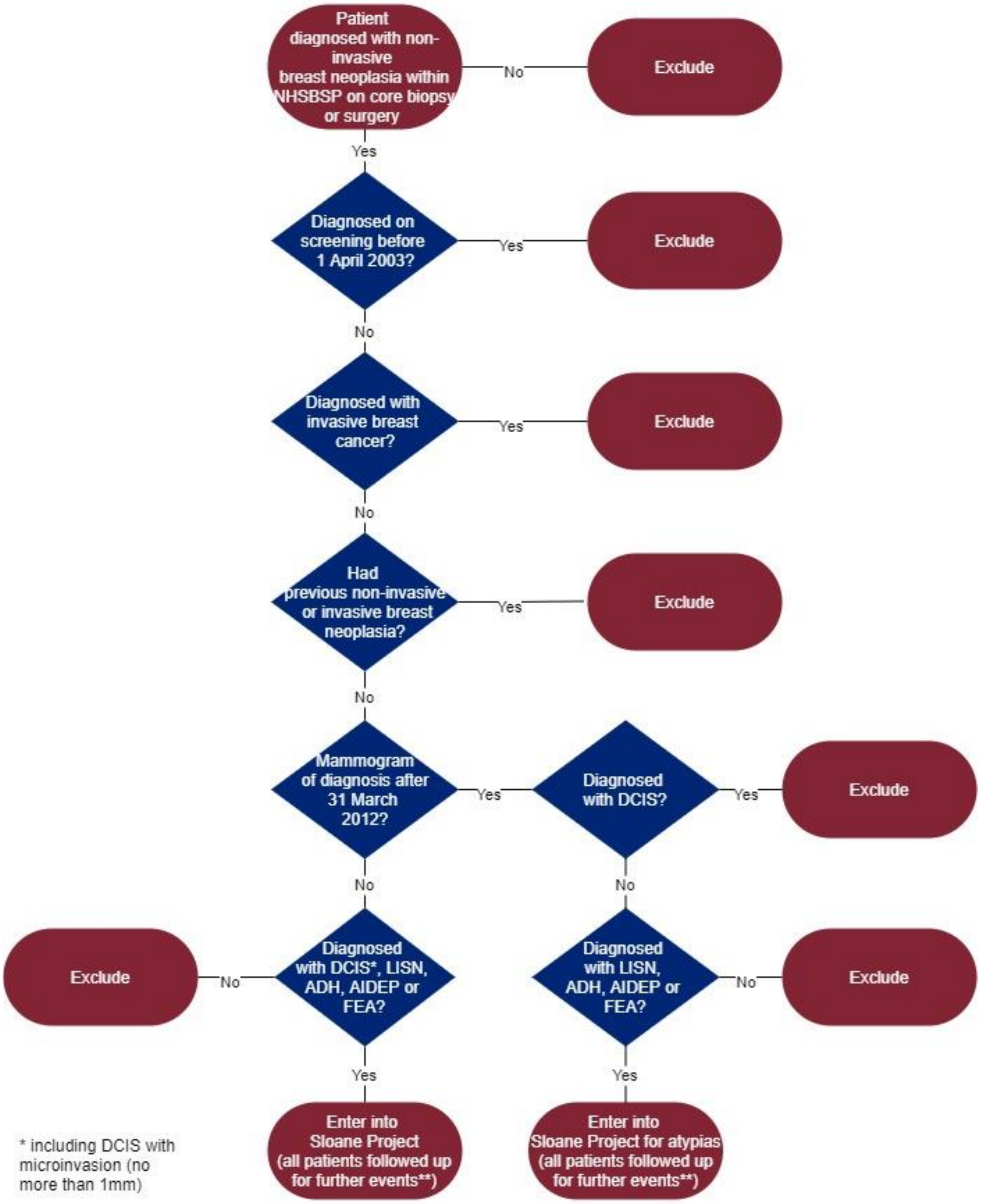


** further events include ipsilateral breast and nodal events, contralateral breast and nodal events and distant events, as well as deaths

| | |
|---|---|
| **NHSBSP** | National Health Service Breast Screening Programme |
| **DCIS** | ductal carcinoma in situ |
| **LISN** | lobular in situ neoplasia (including lobular carcinoma in situ and atypical lobular hyperplasia) |
| **ADH** | atypical ductal hyperplasia |
| **AIDEP** | atypical intraductal epithelial proliferation |
| **FEA** | flat epithelial atypia |

**Figure 1: Sloane eligibility flowchart**

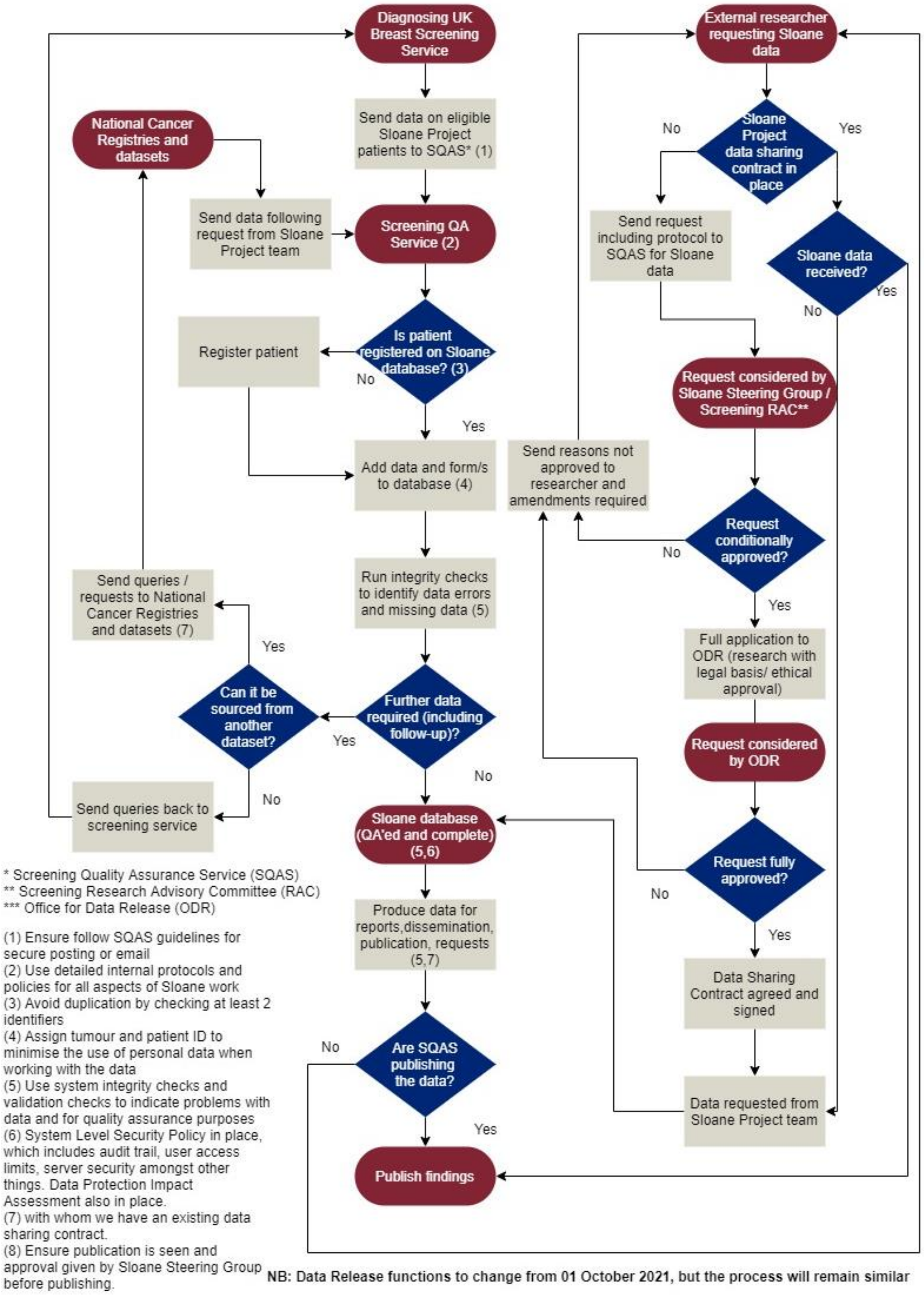


**Figure 2: Example data flow diagram and key for Sloane Project data**

| Type of further event | Definition |
|---|---|
| Further event | (any of): ipsilateral within breast recurrence (or new primary) after BCS; ipsilateral recurrence (includes post-mastectomy/chest wall recurrence); regional or distant recurrence; or contralateral re/occurrence. |
| Ipsilateral recurrence | First recurrence (DCIS or invasive) or new primary (DCIS or invasive) in the same breast, post mastectomy chest wall, or ipsilateral axillary nodes, occurring 6 months or later after surgery for the index lesion. |
| Ipsilateral breast recurrence | first within breast recurrence (DCIS or invasive) or new primary (DCIS or invasive) in the same breast or post mastectomy chest wall occurring 6 months or later after surgery for the index lesion. |
| Distant recurrence | recurrence outwith the breast or post-mastectomy chest wall, (i.e. distant metastatic disease or regional nodal recurrence (ipsilateral or contralateral) in the axilla or supraclavicular fossa) occurring 6 months or later after surgery for the index breast lesion. |
| Contralateral re/occurrence | a within breast recurrence (DCIS or invasive) or new primary (DCIS or invasive) in the contralateral breast or contralateral axillary |

For peer review only

| | |
|---|---|
| | nodes, occurring 6 months or later after surgery for the index lesion in the other breast. |
| Contralateral breast re/occurrence | a within breast recurrence (DCIS or invasive) or new primary (DCIS or invasive) in the contralateral breast occurring 6 months or later after surgery for the index lesion in the other breast. |

**Supplemental Table 1: Definitions of further events in the Sloane Project cohort**

For peer review only

| Cause of death | Definition |
|---|---|
| Breast cancer | Women were considered to have died from breast cancer if breast cancer appeared in the underlying cause of death column in the files available to, or in any section of part 1 (1a, 1b, or 1c) of the death certificate, or if metastases or carcinomatosis from breast cancer were recorded in part 1 of the death certificate, or if metastases or carcinomatosis were recorded in part 1 with breast cancer mentioned in part 2 and no other cancer identified. Women who died of breast cancer but who had no further events recorded were deemed to have had distant metastases on the date they died. |
| Other cancer | Women were considered to have an "other cancer" death if the underlying cause of death was cancer other than breast, or if one or more than one cancer at any site other than breast appeared in part 1 of the death certificate. Breast cancer may have appeared in part 2 or may not have appeared on the death certificate at all but it was not considered to be the underlying cause of death. |
| Cancer of unknown type | Women were considered to have died from an unknown type of cancer when they had metastatic cancer or carcinomatosis as the underlying cause of death, or if metastatic cancer or carcinomatosis was mentioned in part 1 of the death certificate but breast cancer or another type of cancer was not mentioned in any other section. Thus, cancer was known to be the cause of death, but the type of cancer was not known. |
| Non cancer (and non-circulatory) | Women were considered to have a non cancer (and non-circulatory) death if the underlying cause of death was not cancer and was not circulatory (circulatory was defined as any "I" ICD-10 code) or if the death was recorded as non cancer (and non-circulatory) in part 1 of the death certificate in the absence of cancer in part 1. If non cancer (and non-circulatory) cause of |

For peer review only

| | |
|---|---|
| | death was recorded in part 1 but cancer/or circulatory disease was noted in part 2, a check was done to see if it was likely the woman had died with, rather than because of, cancer/circulatory disease. |
| Non cancer (circulatory) deaths | Women were considered to have a circulatory death if the underlying cause of death was a circulatory cause (defined as any "I" ICD-10 code) or a circulatory cause was recorded in part 1 of the death certificate in the absence of cancer in part 1. If cancer was identified in part 2, a check was done to ensure that it was clear that this could not considered to be the underlying cause. |
| Unknown cause of death | Unknown cause of death was recorded when the vital status and date of death had been reported but the cause of death had not been identified. |

**Supplemental Table 2: Definitions of cause of death in the Sloane Project**

For peer review only

# Sloane Project: Radiology Data Form

| | | | |
|---|---|---|---|
| **Surname** | | **Forename(s)** | |
| **Date of Birth** | | **Screening Unit** | |
| **Screening Number (Sx)** | | **Screening Round** | |
| **Date of Screening Mammogram** | | **NHS Number** | |

**Side:** **Left** ☐ **Right** ☐ ***For bilateral disease, please use separate form for each breast.***

**Site (Tick all that are involved)**

Upper outer quadrant ☐ Upper inner quadrant ☐

Lower outer quadrant ☐ Lower inner quadrant ☐ Retroareolar ☐

**Background Pattern (Tick ONE only)**

Fatty ☐ Scattered fibroglandular density ☐

Hetrogeneously dense ☐ Extremely dense ☐

**Predominant Radiological Feature (Tick ONE only)**

Calcification ☐ Parenchymal Distortion ☐

Mass – Well Defined ☐ Spiculate Mass ☐

Mass – Ill Defined ☐ None of the above ☐

**Does the lesion contain microcalcification?** **Yes** ☐ **No** ☐

**If "Yes", what is the most suspicious pattern? (Tick ONE only)**

Cast/linear / Casting ☐ Granular/irregular / Crushed Stone ☐ Punctate / Powderish ☐

**Sloane Project Number (For Official Use Only)** ☐ **QA Ref Number (For Official Use Only)** ☐

For peer review only


Version 2: Issued 01/08/2009

## Estimated Size of Lesion (in mm)

***Mammogram***

| | obl | cc |
|---|---|---|
| Distance from base of nipple to nearest part of the lesion | | |
| Maximum length of lesion | | |
| Maximum diameter of lesion at 90º to long axis of lesion | | |

## Data Required to Calculate Breast Volume (in mm)

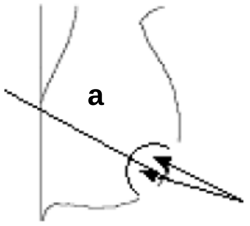


**a =** ☐

**Chest wall to nipple (in the nipple plane)**

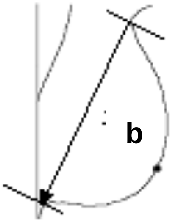


**b =** ☐

**Infra mammary fold to supra mammary (axillary) fold**

***Magnetic Resonance Imaging (MRI)***

Was a pre-operative MRI performed? **Yes** ☐ **No** ☐

Estimated maximum dimension of lesion (mm) on MRI ☐

---

**Specimen X-Ray**

Was a specimen X-ray performed? **Yes** ☐ **No** ☐

**If "Yes":**

Was the specimen X-ray diagnostic or therapeutic? **Diagnostic** ☐ **Therapeutic** ☐

Is the lesion present in the specimen X-ray? **Yes** ☐ **No** ☐

Does the lesion appear radiologically to have been completely excised? **Yes** ☐ **No** ☐

Minimum Distance from Edge of Lesion to Edge of Specimen (in mm) ☐

---

**Date (DD/MM/YY)** ☐ **Radiologist (Print name)** ☐

For peer review only - http://bmjopen.bmj.com/site/about/guidelines.xhtml

For peer review only

# Sloane Project: Treatment Data Form

| | | | |
|---|---|---|---|
| **Name** | | **Screening No.** | |
| **Date of Birth** | | **Screening Unit** | |
| **Hospital** | | **Hospital No.** | |
| | | **NHS No.** | |

**Side:** Left ☐ Right ☐ ***For bilateral disease, please use separate form for each breast.***

**Surgical Procedure(s)**
Total number of operations performed
(if more than 2 operations please use separate form for operation 3 onwards) ☐

Was there a pre-operative diagnosis of DCIS? **Yes** ☐ **No** ☐

| | | | |
|---|---|---|---|
| **Operation 1** Date (dd/mm/yy) | | **Operation 2** Date (dd/mm/yy) | |
| Grade of surgeon performing operation | | Grade of surgeon performing operation | |

**Breast Procedures**
Please indicate procedures undertaken at Operation 1 & Operation 2.
(Tick one box only for each operation. If more than 2 operations please use separate form)

| | Op 1 | Op 2 | | Op 1 | Op 2 |
|---|---|---|---|---|---|
| Diagnostic Biopsy | ☐ | ☐ | Simple mastectomy | ☐ | ☐ |
| Wide Local Excision including excision of nipple | ☐ | ☐ | Subcutaneous mastectomy | ☐ | ☐ |
| | | | Skin sparing mastectomy | ☐ | ☐ |
| Wide Local Excision preserving nipple | ☐ | ☐ | Therapeutic mammoplasty with nipple preservation | ☐ | ☐ |
| Further excision to clear margins | ☐ | ☐ | Therapeutic mammoplasty without nipple preservation | ☐ | ☐ |

**Immediate Breast Reconstruction**
(If more than 2 operations please use separate form)

| | Op 1 | Op 2 | ***Plus Implants (please tick)*** | | Op 1 | Op 2 |
|---|---|---|---|---|---|---|
| Latissimus Dorsi flap | ☐ | ☐ | ☐ | Wide Local Excision + mini latissimus dorsi flap (Rainsbury's procedure) | ☐ | ☐ |
| D.I.E.P. flap | ☐ | ☐ | ☐ | Implant only | ☐ | ☐ |
| T.R.A.M. flap | ☐ | ☐ | ☐ | Other autologous tissue technique | ☐ | ☐ |

**Sloane Project Number (For Official Use Only)**

**QA Ref Number (For Official Use Only)**

Version 2: Issued 01/08/2009

For peer review only

**Axillary Procedures**

| | Op 1 Yes | Op 1 No | Op 2 Yes | Op 2 No |
|---|---|---|---|---|
| Was a decision(s) made pre-operatively re axillary surgery at the MDM? | ☐ | ☐ | ☐ | ☐ |

Were axillary nodes taken? **Yes** ☐ **No** ☐

**If "Yes" which of the following procedures were carried out.**
(Tick all that apply. If more than 2 operations performed please use separate form.)

| | Op 1 | Op 2 |
|---|---|---|
| Sentinel node Procedure | ☐ | ☐ |
| Axillary node sample | ☐ | ☐ |
| Axillary node clearance | ☐ | ☐ |

**Further Surgical Details**
**For women treated with conservation surgery:**

| | Op 1 Yes | Op 1 No | Op 2 Yes | Op 2 No |
|---|---|---|---|---|
| Was the margin nearest to the nipple marked? | ☐ | ☐ | ☐ | ☐ |
| Was the specimen orientated? | ☐ | ☐ | ☐ | ☐ |

**For ALL operation types:**

| | Op 1 Yes | Op 1 No | Op 2 Yes | Op 2 No |
|---|---|---|---|---|
| Was a further excision performed at the same operation? | ☐ | ☐ | ☐ | ☐ |
| Was the immediate re-excision orientated? | ☐ | ☐ | ☐ | ☐ |
| Were cavity shavings (bed biopsies) taken? | ☐ | ☐ | ☐ | ☐ |
| Was a further excision carried out on the basis of the Specimen X-ray at the time? | ☐ | ☐ | ☐ | ☐ |

**Treatment Strategy – Following the Final Operation**

Was the status of the margins discussed at the MDM? **Yes** ☐ **No** ☐

Does the final margin status fulfil local protocol for adequacy of resection margins? **Yes** ☐ **No** ☐ **No Protocol** ☐

If "No" to above question, please state reasons why no further surgical treatment was undertaken if margins were inadequate. ☐

Did the post-op MDM decision on adjuvant therapy include (Tick all that apply):

Referral for Radiotherapy ☐ No further adjuvant therapy ☐ Other therapy ☐

Hormone therapy ☐ Date started Hormone Therapy ☐

Type of Hormone Therapy Tamoxifen ☐ Aromatase Inhibitor ☐ Other ☐

**Clinical Trials**
Patient entered Clinical Trial **Yes** ☐ **No** ☐ **Name of trial entered** ☐

**Date (DD/MM/YY)** ☐ **Surgeon (Print name)** ☐

Version 2: Issued 01/08/2009

# Sloane Project: Pathology Data Form

**Please complete ONE form for an EPISODE.**

***For bilateral disease, please use separate form for each breast.***

| | | | |
|---|---|---|---|
| **Surname** | | **Forename(s)** | |
| **Date of Birth** | | **Screening Unit** | |
| **Hospital** | | **Hospital No.** | |
| **Histology Report No.** | | **NHS No.** | |

**Side:** Left ☐ Right ☐

**Specimen Type (Tick ALL that apply, and weight, if known)**

| | Y/N | Disease present in core: DCIS | ADH | LCIS |
|---|---|---|---|---|
| Core biopsy (standard 14 gauge) | | | | |
| 8 or 11 gauge core (e.g. mammotomy) | | | | |

DCIS core grade: High ☐ Intermediate ☐ Low ☐

Calcification Present: None ☐ Benign ☐ Malignant ☐ Benign & Malignant ☐

| **Surgical Specimens:** | **Y/N** | **Weight (g)** | | **Y/N** | **Weight (g)** |
|---|---|---|---|---|---|
| Diagnostic Open Biopsy | | | Therapeutic Excision (WLE) | | |
| Bed Biopsies/ Cavity Shaves | | | Delayed re-excision (i.e. at a later operation) | | |
| Immediate re-excision (i.e. at time of WLE) | | | Completion Mastectomy | | |
| | | | Mastectomy | | |

| **For therapeutic excision / WLE:** | **Yes** | **No** | **Uninterpretable** |
|---|---|---|---|
| Nipple orientation marker present? | | | |
| Other orientation markers (sutures/clips)? | | | |

**Excision Histology – Lesion** (Tick all present): **DCIS** ☐ **ADH** ☐ **LISN** ☐

**For DCIS only**

Maximum (total) size of DCIS (mm) (summation from all specimens) ☐

Please tick if the total is size from one specimen only ☐

If summation from several specimens, please give size in each (mm) ☐ + ☐ + ☐

**Sloane Project Number (For Official Use Only)** ☐

**QA Ref Number (For Official Use Only)** ☐



Version 2: Issued 01/08/2009

For peer review only

**Highest Nuclear Grade (Please tick one only)** High ☐ Intermediate ☐ Low ☐

**Growth Pattern (Tick all that apply)**

Solid ☐ Cribriform ☐ Micropapillary ☐ Papillary ☐

Apocrine ☐ Flat/clinging ☐ Other type, please state……..…………………

| | Yes | No | | Yes | No | N/A |
|---|---|---|---|---|---|---|
| Comedo necrosis present? | ☐ | ☐ | Paget's disease? | ☐ | ☐ | ☐ |

| | Yes | No | | |
|---|---|---|---|---|
| Microinvasion (<1mm)? | ☐ | ☐ | No. foci microinvasion? | ☐ |

**Distance of DCIS to margins (mm)** **(Please complete all available and indicate if >10mm)**

| | Medial | Lateral | Superior | Inferior | Superficial | Deep | Nipple |
|---|---|---|---|---|---|---|---|
| **Therapeutic Excision** | ☐ mm | ☐ mm | ☐ mm | ☐ mm | ☐ mm | ☐ mm | ☐ mm |

***Please supply margin data above for a therapeutic excision, even if followed by mastectomy.***

| | | | | | | | |
|---|---|---|---|---|---|---|---|
| **If altered by further surgery, final margin distance** | ☐ mm | ☐ mm | ☐ mm | ☐ mm | ☐ mm | ☐ mm | ☐ mm |

**Mastectomy**
*(please tick if this applies)* ☐

Does another process extend to the margin?
(Please tick all that apply) **ADH** ☐ **LISN** ☐

**Immediate re-excision / Cavity Shaves / Bed Biopsies**

No. samples received ☐

| | DCIS | ADH | LISN |
|---|---|---|---|
| Did these samples contain (Tick all that apply) | ☐ | ☐ | ☐ |

| **Nodes** | **Axillary Nodes** | **Sentinel Node** | **Others** |
|---|---|---|---|
| Number examined overall | ☐ | ☐ | ☐ |
| Number positive overall | ☐ | ☐ | ☐ |

| **Receptor Status** | **Positive** | **Negative** | **Not Known** | **Cut off used** |
|---|---|---|---|---|
| ER Status | ☐ | ☐ | ☐ | |
| PGR Status | ☐ | ☐ | ☐ | |
| HER-2 Status | ☐ | ☐ | ☐ | |

**Date (DD/MM/YY)** ☐ **Pathologist (Print name)** ☐

For peer review only



Version 2: Issued 01/08/2009

# Sloane Project: Radiotherapy Data Form

**Surname** [ ] **Forename(s)** [ ]

**Date of Birth** [ ] **Screening Unit** [ ]

**Hospital** [ ] **Hospital No.** [ ]

**NHS No.** [ ]

**Side:** Left [ ] Right [ ] ***For bilateral disease, please use separate form for each breast.***

**Method of treatment**
**Radiotherapy - External Beam** **Yes** [ ] **No** [ ] ***If "No" why?*** [ ]

***If "Yes" please complete the following:-***

Dose(Gy) [ ] Energy (MV) [ ] Number of fractions [ ]

Treated daily? **Yes** [ ] **No** [ ] *If "**No**" please state frequency* [ ]

Date treatment commenced (dd/mm/yy) [ ] Date treatment finished (dd/mm/yy) [ ]

Axilla treated? **Yes** [ ] **No** [ ] Boost given? **Yes** [ ] **No** [ ]

*If Boost given, please complete the following:-*

Electrons (Energy – MeV) [ ] Megavoltage (Energy – MV) [ ] Orthovoltage (Energy – kV) [ ] Dose (Gy) [ ] Number of fractions [ ]

Treated daily? **Yes** [ ] **No** [ ]

Date treatment commenced (dd/mm/yy) [ ] Date treatment finished (dd/mm/yy) [ ]

**Brachytherapy – Interstitial**

**Yes** [ ] **No** [ ] *If "**Yes**" please complete the following:-* Dose(Gy) [ ]

**Date (DD/MM/YY))** [ ] **Oncologist (Print name)** [ ]

**Sloane Project Number (For Official Use Only)** [ ] **QA Ref Number (For Official Use Only)** [ ]

For peer review only


Version 2: Issued 01/08/2009

# SLOANE PROJECT FOLLOW UP FORM
# (FOR RECURRENCES)

**The purpose of this detailed follow up form is to accumulate information on ipsilateral recurrences, contralateral breast disease and metastases. The form is split up into four sections:**

1) **Demographic details (these will already be completed if all information known)**
2) **Type of recurrence detected and route of presentation**
3) **Diagnosis, treatment and pathology of recurrence/contralateral disease**
   (a) **Ipsilateral recurrence**
   (b) **Contralateral breast disease**
4) **Diagnosis and treatment of distant metastases**

**Please complete all sections that apply to this patient.**

---

## SECTION 1 Demographic Details

**Screening Unit** ☐ **Date of Birth** ☐

**NHS Number** ☐ **Screening Number** ☐

**Hospital Number** ☐

---

## SECTION 2

### Diagnosis of recurrence / contralateral disease / metastases

**Type of Recurrence Detected and Date of Diagnosis** (please tick all that apply)

Local-regional ☐ Distant ☐ Contralateral disease ☐

Date ☐ Date ☐ Date ☐

**Route of Presentation** (tick all that apply)

Detected on FU mammogram ☐ Clinical Exam at routine FU ☐

Following GP referral to OPD clinic ☐ Other (please give details) ____________________

---


For peer review only - http://bmjopen.bmj.com/site/about/guidelines.xhtml

**SECTION 3 (a) Local-regional recurrence**

**The purpose of this section is to accumulate information on the IPSILATERAL RECURRENCE – when it occurred, where it occurred in the breast, how it presented and how it was treated.**

**Diagnosis of local-regional recurrence**

| Site of local-regional recurrence(s) (Tick all that apply) | | Procedures used to confirm recurrence (Tick all that apply) | | | | | |
|---|---|---|---|---|---|---|---|
| | | Mammogram | FNA | Core Biopsy | Excision biopsy | MRI | Other (please specify) |
| Breast (if conserved) – at or adjacent to site of original primary | | | | | | | |
| Breast (if conserved) – second neoplasm some distance from site of original | | | | | | | |
| Nipple | | | | | | | |
| Mastectomy scar/flaps | | | | | | | |
| Ipsilateral axilla | | | | | | | |
| Ipsilateral supraclavicular fossa | | | | | | | |
| Reconstructed breast mound | | | | | | | |
| Other (Please specify) | | | | | | | |

**Treatment of local-regional recurrence**

**Treatment of local-regional recurrence (Tick all that apply)**

Surgical procedures ☐ Radiotherapy ☐ Hormone Therapy ☐ Chemotherapy ☐

**Surgical procedures (tick all that apply)**

Further Wide Local Excision ☐ Mastectomy ☐ Axillary node surgery ☐ Other ______________ (Please give details)

**Radiotherapy to recurrence (Please tick all sites that were treated)**

Breast ☐ Axilla ☐ Chest wall ☐ Supraclavicular fossa ☐ Interstitial ☐

**Hormone Therapy (please tick if given for recurrence)**

Tamoxifen ☐ Aromatase Inhibitor ☐ Other (Please state) ______________

**Chemotherapy (please give details of the regime)**

CMF alone ☐ Herceptin ☐ Anthracycline containing regime (e.g. FEC, Epi-CMF) ☐

Taxane containing regime (e.g. Taxol, Taxotere) ☐ Other regime (Please give details) ______________

For peer review only



Version 2: Issued 01/08/2009

## SECTION 3 (a) – continued
## Pathology of local-regional recurrence

**Type and Grade of recurrence (Please tick all that apply)**

Invasive ☐ Non-Invasive (DCIS) ☐ Non-invasive (LCIS/ALH) ☐

| | | | | | | |
|---|---|---|---|---|---|---|
| **Invasive grade** | Grade 1 | ☐ | Grade 2 | ☐ | Grade 3 | ☐ |
| **DCIS Grade** | Low | ☐ | Intermediate | ☐ | High | ☐ |

**Size of local-regional recurrence**

DCIS (mm) ☐ Invasive size (mm) ☐ Whole tumour (DCIS + invasive) size (mm) ☐

**DCIS growth pattern(s) (Tick all that apply)**

Solid ☐ Cribriform ☐ Micropapillary ☐ Papillary ☐

Apocrine ☐ Flat ☐ Other (please specify) ☐

**Microinvasion** Present ☐ Not Present ☐

**Histological Type of Invasive Tumour**

No special type (ductal NST) ☐ Pure special type (90% purity, specify components present below) ☐ Mixed tumour type (50-90% special type component, specify components present below) ☐

**Component (s) present for pure special type and mixed tumour types:**

Tubular/cribriform ☐ Lobular ☐ Mucinous ☐ Medullary like ☐

Ductal / no special type ☐ Other (please specify) ☐

**Nodes**

Number examined overall ☐ Number positive ☐

**Vascular invasion** Present ☐ Possible ☐ Absent ☐ Not known ☐

**Receptor status**

| | Positive | Negative | Not known | Cut off for positivity used | Invasive | DCIS (indicate) |
|---|---|---|---|---|---|---|
| ER status | ☐ | ☐ | ☐ | ☐ | ☐ | ☐ |
| PgR status | ☐ | ☐ | ☐ | ☐ | ☐ | ☐ |
| HER-2 status | ☐ | ☐ | ☐ | ☐ | ☐ | ☐ |

Version 2: Issued 01/08/2009

For peer review only - http://bmjopen.bmj.com/site/about/guidelines.xhtml

## SECTION 3 (b) Contralateral disease

**We need to quantify the risk to the opposite breast, which is why we need to know whether the cancer is invasive or non-invasive and how it was treated.**

| Site of contralateral disease (Tick all that apply) | | Procedures used to confirm contralateral disease (Tick all that apply) | | | | | |
|---|---|---|---|---|---|---|---|
| | | Mammogram | FNA | Core Biopsy | Excision biopsy | MRI | Other (please specify) |
| Contralateral Breast | | | | | | | |
| Contralateral Nipple | | | | | | | |
| Contralateral axilla | | | | | | | |
| Other (Please specify) | | | | | | | |

### Treatment of contralateral disease

**Treatment of contralateral disease (Tick all that apply)**

Surgical procedures ☐ Radiotherapy ☐ Hormone Therapy ☐ Chemotherapy ☐

**Surgical procedures (tick all that apply)**

Wide Local Excision ☐ Mastectomy ☐ Axillary node surgery ☐ Other ____________________ (Please give details)

**Radiotherapy for contralateral disease (Please tick all sites that were treated)**

Breast ☐ Axilla ☐ Chest wall ☐

Supraclavicular fossa ☐ Interstitial ☐

**Hormone Therapy (please tick if given after diagnosis of contralateral disease)**

Tamoxifen ☐ Aromatase Inhibitor ☐ Other (Please state) ____________________

**Chemotherapy (please give details of the regime)**

CMF alone ☐ Herceptin ☐ Anthracycline containing regime ☐ (e.g. FEC, Epi-CMF)

Taxane containing Regime (e.g. Taxol, Taxotere) ☐ Other regime (Please give details) ____________________

**Other treatment given (please specify)** ____________________

Version 2: Issued 01/08/2009

## SECTION 3 (b) – continued
## Pathology of contralateral disease

**Type and Grade of recurrence (Please tick all that apply)**

| | | | | | |
|---|---|---|---|---|---|
| Invasive | ☐ | Non-Invasive (DCIS) | ☐ | Non-invasive (LCIS/ALH) | ☐ |

| | | | | | | |
|---|---|---|---|---|---|---|
| **Invasive grade** | Grade 1 | ☐ | Grade 2 | ☐ | Grade 3 | ☐ |
| **DCIS Grade** | Low | ☐ | Intermediate | ☐ | High | ☐ |

**Size of Contralateral recurrence**

| | | | | | |
|---|---|---|---|---|---|
| DCIS (mm) | ☐ | Invasive size (mm) | ☐ | Whole tumour (DCIS + invasive) size (mm) | ☐ |

**DCIS growth pattern(s) (Tick all that apply)**

| | | | | | | | |
|---|---|---|---|---|---|---|---|
| Solid | ☐ | Cribriform | ☐ | Micropapillary | ☐ | Papillary | ☐ |
| Apocrine | ☐ | Flat | ☐ | Other (please specify) | ☐ | | |

| | | | | |
|---|---|---|---|---|
| **Microinvasion** | Present | ☐ | Not Present | ☐ |

**Histological Type of Invasive Tumour**

| | | | | | |
|---|---|---|---|---|---|
| No special type (ductal NST) | ☐ | Pure special type (90% purity, specify components present below) | ☐ | Mixed tumour type (50-90% special type component, specify components present below) | ☐ |

**Component (s) present for pure special type and mixed tumour types:**

| | | | | | | | |
|---|---|---|---|---|---|---|---|
| Tubular/cribriform | ☐ | Lobular | ☐ | Mucinous | ☐ | Medullary like | ☐ |
| Ductal / no special type | ☐ | Other (please specify) | ☐ | | | | |

**Nodes**

| | | | |
|---|---|---|---|
| Number examined overall | ☐ | Number positive | ☐ |

| | | | | | | |
|---|---|---|---|---|---|---|
| **Vascular invasion** | Present | ☐ | Possible | ☐ | Absent | ☐ |

**Receptor status**

| | **Positive** | **Negative** | **Not known** | **Cut off for positivity used** | **Invasive** | **DCIS (indicate)** |
|---|---|---|---|---|---|---|
| ER status | ☐ | ☐ | ☐ | ☐ | ☐ | ☐ |
| PgR status | ☐ | ☐ | ☐ | ☐ | ☐ | ☐ |
| HER-2 status | ☐ | ☐ | ☐ | ☐ | ☐ | ☐ |

Version 2: Issued 01/08/2009

For peer review only - http://bmjopen.bmj.com/site/about/guidelines.xhtml

## SECTION 4 Distant metastases

**We would like information on whether or not the patient has confirmed distant metastases.**

**Site of distant metastases (Tick all that apply)**

Bone ☐ Lung ☐ Liver ☐ Brain ☐ Other (please specify) ____________________

**Treatment (Tick all that apply)**

**Surgical Procedures** ☐ If ticked, please indicate type of procedure ____________________

**Radiotherapy** ☐ If ticked, please give details of site ____________________

**Hormone Therapy** ☐ If ticked, please indicate type of hormone therapy ____________________

**Bisphosphonates** ☐

**Chemotherapy** ☐ If ticked, please give details of type of regime ____________________

**Other** ☐ If ticked, please give details ____________________

**Is there any evidence of any invasive focus to account for the metastases?**

Yes ☐ No ☐ N/K ☐ Please comment if you wish ____________________

**Thank you for your co-operation.**

Version 2: Issued 01/08/2009

For peer review only

### Validation checks

strErrorText

- Check # 01 entries have been found where the SLOANE ID is '0' in the operation details table.
- Check # 02 patient(s) do not have matching radiology details.
- Check # 03 radiology record(s) do not have matching patient records.
- Check # 04 radiology record(s) do not have a pathology record.
- Check # 05 pathology record(s) do not have a radiology record.
- Check # 06 radiology record(s) do not have a treatment strategy record.
- Check # 07 treatment strategy record(s) do not have a radiology record.
- Check # 08 appointment record(s) do not have a radiology record.
- Check # 09 appointment record(s) do not have an operation record.
- Check # 10 operation record(s) do not have an appointment record.
- Check # 11 treatment strategy record(s) do not have a radiotherapy record.
- Check # 12 radiotherapy record(s) do not have a treatment strategy record.
- Check # 13 appointment(s) are potentially duplicate records (Visit ID).
- Check # 14 operation(s) are potentially duplicate records (Visit ID).
- Check # 15 pathology record(s) are potentially duplicates.
- Check # 16 QARC ID(s) are potentially duplicates (Patient table).
- Check # 17 SLOANE ID(s) are potentially duplicates (Radiology table).
- Check # 18 radiotherapy ID(s) are potentially duplicates.
- Check # 19 treatment strategy ID(s) are potentially duplicates.
- Check # 20 patients have NHS numbers that do not conform to the NHS standard.
- Check # 21 patient(s) with more than one appointment (operation) recorded for the same day (this is unusual and should be checked for accuracy).
- Check # 22 patient(s) with more than two mammograms recorded (this is unusual and should be checked for accuracy).
- Check # 23 patient(s) with more than two mammograms recorded for the same side (this is unusual and should be checked for accuracy).
- Check # 24 patient(s) with the date of mammogram not recorded.
- Check # 25 patient(s) whose adjuvant therapy details are not recorded.
- Check # 26 patient(s) have clear margins but details are not recorded.
- Check # 27 case(s) of DCIS with no Highest Nuclear Grade recorded.

Check # 28 case(s) with multiple cut up protocols recorded.

Check # 29 case(s) with multiple specimen X-Ray types recorded.

Check # 30 case(s) where a lesion was excised without one being present.

Check # 31 case(s) without microcalcification that have a suspicious pattern recorded.

Check # 32 case(s) where a lesion was excised without a specimen X-Ray having been performed.

Check # 33 case(s) with no specimen X-Ray performed, but Diagnostic or Theraputic has been recorded.

Check # 34 case(s) where a lesion was present in the specimen X-Ray without one having been performed.

Check # 35 case(s) where a boost was given, but treated daily is unrecorded.

Check # 36 case(s) where an external beam was given, but axilla treated is unrecorded.

Check # 37 case(s) where an external beam was given, but boost given is unrecorded.

Check # 38 case(s) where both an external beam and brachytherapy were given.

Check # 39 case(s) where an external beam was given, but treated daily is unrecorded.

Check # 40 case(s) where treated daily, but boost given is unrecorded.

For peer review only

Check # 41 case(s) where treated daily, but external beam is unrecorded.
Check # 42 case(s) where axillary nodes not taken, but procedures have been recorded.
Check # 43 case(s) where axillary nodes are taken, but procedure is unrecorded.
Check # 44 case(s) where no further surgical treatment undertaken and no reason has been recorded.
Check # 45 case(s) where further surgical treatment undertaken and reason(s) for not have been recorded.
Check # 46 case(s) where no further excision was carried out, but was orientated.
Check # 47 case(s) where a further excision was carried out without a specimen X-Ray.
Check # 48 case(s) where the lesion was confirmed to the surgeon without a specimen X-Ray having been done.
Check # 49 case(s) where a lesion was present in the specimen X-Ray without one having been performed.
Check # 50 case(s) where the patient entered clinical trials, but a trial name has not been recorded.
Check # 51 case(s) where the patient refused clinical trials, but a trial name has been recorded.
Check # 52 case(s) where a grade hasn't been recorded against a surgeon.
Check # 53 case(s) where a surgeon's name hasn't been recorded against a grade.
Check # 54 case(s) where the recorded specimen sizes do not match the total size of the lesion.

- Check # 57 (AgeAtMammogram)
- Check # 58 (BiLateral_CheckDates)
- Check # 59 (BreastProcedure_10_or_14)
- Check # 61 (DCIS_TotalSpecimenSizes)
- Check # 62 (DOB_MammoDate_OpDates)
- Check # 63 (DuplicateQARCIDs)
- Check # 64 (DuplicateQARCIDs_NotBiLateral)
- Check # 65 (ExcisionalOperatorsIncorrect)
- Check # 66 (ExcisionalValuesIncorrect)
- Check # 67 (ImmedReconMoreThan2)
- Check # 68 (ImmedReconNoMx)
- Check # 69 (MoreThanOnePrimary)
- Check # 70 (MultipleImmediateReconstruction)
- Check # 71 (MultipleMx)
- Check # 72 (OperationsFinalOpNo)
- Check # 73 (OperationsMoreThan4)
- Check # 74 (Path_BiopsyNoDetail)
- Check # 75 (Path_CheckMicroinvasionPresent)
- Check # 76 (Path_CheckNoOfFociMicroinvasion)
- Check # 77 (Path_CompletionMxOnlyOneOp)
- Check # 78 (Path_CompletionMxWithMx)
- Check # 79 (Path_DCISMicroinvasionNull)
- Check # 80 (Path_DCISMicroinvasionUnknown)
- Check # 81 (Path_DCISNoComedoNecrosis)
- Check # 82 (Path_DCISSizeIncorrect)
- Check # 83 (Path_MarginClear)
- Check # 84 (Path_MxMarginCheck)
- Check # 85 (Path_MxNoOperation)
- Check # 86 (Path_NodesCheckBlanks)

For peer review only

- Check # 87 (Path_NodesCheckValues)
- Check # 88 (Path_NodesOverall)
- Check # 89 (Path_NodesPositive)
- Check # 90 (Path_UnknownHistology)
- Check # 91 (Pathology_DCISNoNuclearGrade)
- Check # 92 (Pathology_MultipleCutUpProtocols)
- Check # 93 (PreOpDiagnosis)
- Check # 94 (Radiology_MicrocalcificationNotPresentWithNottmDef)
- Check # 95 (Radiology_MicrocalcificationPresentNoNottmDef)
- Check # 96 (Radiology_MultipleSpecimenXRays)
- Check # 97 (Radiology_NoLesionPresentLesionExcised)
- Check # 98 (Radiology_NoMicrocalcificationWithNottmDefinition)
- Check # 99 (Radiology_NoSpecimenXRayLesionExcised)
- Check # 100 (Radiology_NoSpecimenXRayWithDiagOrTheraputic)
- Check # 101 (Radiology_NoSpecimenXRayWithLesionPresent)
- Check # 102 (RadiologyBackgroundPattern)
- Check # 103 (RadiologyCheckLesionValues)
- Check # 104 (RadiologyCheckLesionValuesErrorsDiameter)
- Check # 105 (RadiologyCheckLesionValuesErrorsDistance)
- Check # 106 (RadiologyCheckLesionValuesErrorsLength)
- Check # 107 (RadiologyCheckLesionValuesErrorsVolume_2r)
- Check # 108 (RadiologyCheckLesionValuesErrorsVolume_h)
- Check # 109 (RadiologyPredominantRadiologicalFeature)
- Check # 110 (Radiotherapy_BoostGivenYesTreatedDailyNull)
- Check # 111 (Radiotherapy_ExternalBeamYesAxillaTreatedNull)
- Check # 112 (Radiotherapy_ExternalBeamYesBoostGivenNull)
- Check # 113 (Radiotherapy_ExternalBeamYesBrachytherapyYes)
- Check # 114 (Radiotherapy_ExternalBeamYesTreatedDailyNull)
- Check # 115 (Radiotherapy_NoBoostTreatedDaily)
- Check # 116 (Radiotherapy_NoExternalBeamTreatedDaily)
- Check # 117 (RadiotherapyUnknown)
- Check # 118 (RadiotherapyYesFormNotReceived)
- Check # 119 (RadiotherapyYesWithMx)
- Check # 120 (ScreeningRound0NullOrMoreThan8)
- Check # 121 (ScreeningYearOfDiagnosis)
- Check # 122 (SLOANEIDCheckFirstAndLast)
- Check # 123 (Treat_AdjuvantTherapyYes)
- Check # 124 (Treat_AxProcedures)
- Check # 125 (Treatment_AxillaryNodesNotTakenWithProcedures)
- Check # 126 (Treatment_AxillaryNodesTakenWithNoProcedures)
- Check # 127 (Treatment_FurtherSurgeryNoWithNoReason)
- Check # 128 (Treatment_FurtherSurgeryYesWithReason)
- Check # 129 (Treatment_NoFurtherExcisionButOrientated)
- Check # 130 (Treatment_NoOperationDate)
- Check # 131 (Treatment_NoSpecimenXRayFutherExcisionCarriedOut)
- Check # 132 (Treatment_NoSpecimenXRayLesionConfirmedToSurgeon)

For peer review only

- Check # 133 (Treatment_NoSpecimenXRayLesionPresent)
- Check # 134 (Treatment_PatientNotEnteredTrialWithTrialName)
- Check # 135 (Treatment_PatientRefusedTrialAndEntered)
- Check # 136 (Treatment_SurgeonNoGrade)
- Check # 137 (Treatment_SurgicalGradeNoSurgeon)
- Check # 138 (TreatStrat_AdjuvantTherapyUnknown)
- Check # 139 (TreatStrat_RadiotherapyWithAdjuvantTherapy)
- Check # 140 (TreatStrat_RefereForRadioYesNoAdjuventYes)
- Check # 141 (TreatStrat_ReferForRadioYesMxYes)
- Check # 142 (TreatStrat_ReferForRadioYesNoRadioForm)
- Check # 143 (AgeCheck)
- Check # 144 (OpAttendanceDatesNoOperation)

For peer review only

BMJ Open

# Cohort profile of the Sloane Project – methodology for a prospective UK cohort study of >15,000 women with screen-detected non-invasive breast neoplasia

| | |
|---|---|
| Journal: | *BMJ Open* |
| Manuscript ID | bmjopen-2022-061585.R1 |
| Article Type: | Cohort profile |
| Date Submitted by the Author: | 10-Oct-2022 |
| Complete List of Authors: | Clements, Karen; NHS England and NHS Improvement Midlands, Screening Quality Assurance Service<br>Dodwell, David; University of Oxford, Nuffield Department of Population Health<br>Hilton, Bridget; NHS England and NHS Improvement Midlands, Screening QA Service<br>Stevens-Harris, Isabella; University Hospitals of Derby and Burton NHS Foundation Trust<br>Pinder, Sarah; Guy's & St Thomas' NHS Foundation Trust, Histopathology; King's College London Faculty of Life Sciences and Medicine, School of Cancer and Pharmaceutical Sciences<br>Wallis, M. G.; Cambridge Univ Hosp NHS Trust, Cambridge Breast Unit<br>Maxwell, AJ; Manchester University NHS Foundation Trust, Nightingale Centre<br>Kearins, Olive; NHS England and NHS Improvement Midlands, National Lead Breast Screening QA<br>Sibbering, Mark; University Hospitals of Derby and Burton NHS Foundation Trust<br>Shaaban, Abeer; Queen Elizabeth Hospital Birmingham and Univeristy of Birmingham, Histopathology<br>Kirwan, Cliona; The University of Manchester Faculty of Biology Medicine and Health, Division of Cancer Sciences<br>Sharma, Nisha; Leeds Teaching Hospitals NHS Trust, Breast Unit, St James's Hospital<br>Stobart, Hilary; Independent Cancer Patients' Voice<br>Dulson-Cox, Joanne; NHS England and NHS Improvement Midlands, Screening Quality Assurance Service<br>Litherland, Janet; West Scotland Breast Screening Centre<br>Mylvaganam, Senthurun; Royal Wolverhampton Hospitals NHS Trust<br>Provenzano, Elena; Cambridge University Hospitals NHS Foundation Trust<br>Sawyer, Elinor; King's College London Faculty of Life Sciences and Medicine, School of Cancer and Pharmaceutical Sciences<br>Thompson, Alastair M ; Baylor College of Medicine, Department of Surgical Oncology, Dan L Duncan Comprehensive Cancer Center, |
| <b>Primary Subject Heading</b>: | Oncology |

| | |
|---|---|
| Secondary Subject Heading: | Research methods, Public health, Health services research, Epidemiology |
| Keywords: | AUDIT, Protocols & guidelines < HEALTH SERVICES ADMINISTRATION & MANAGEMENT, Breast tumours < ONCOLOGY, PUBLIC HEALTH, STATISTICS & RESEARCH METHODS |
| | |


SCHOLARONE™
Manuscripts

*I, the Submitting Author has the right to grant and does grant on behalf of all authors of the Work (as defined in the below author licence), an exclusive licence and/or a non-exclusive licence for contributions from authors who are: i) UK Crown employees; ii) where BMJ has agreed a CC-BY licence shall apply, and/or iii) in accordance with the terms applicable for US Federal Government officers or employees acting as part of their official duties; on a worldwide, perpetual, irrevocable, royalty-free basis to BMJ Publishing Group Ltd ("BMJ") its licensees and where the relevant Journal is co-owned by BMJ to the co-owners of the Journal, to publish the Work in this journal and any other BMJ products and to exploit all rights, as set out in our licence.*

*The Submitting Author accepts and understands that any supply made under these terms is made by BMJ to the Submitting Author unless you are acting as an employee on behalf of your employer or a postgraduate student of an affiliated institution which is paying any applicable article publishing charge ("APC") for Open Access articles. Where the Submitting Author wishes to make the Work available on an Open Access basis (and intends to pay the relevant APC), the terms of reuse of such Open Access shall be governed by a Creative Commons licence – details of these licences and which Creative Commons licence will apply to this Work are set out in our licence referred to above.*

*Other than as permitted in any relevant BMJ Author's Self Archiving Policies, I confirm this Work has not been accepted for publication elsewhere, is not being considered for publication elsewhere and does not duplicate material already published. I confirm all authors consent to publication of this Work and authorise the granting of this licence.*

For peer review only

# Cohort profile of the Sloane Project –methodology for a prospective UK cohort study of >15,000 women with screen-detected non-invasive breast neoplasia

Karen Clements, NHS England and NHS Improvement, 23 Stephenson Street, Birmingham, B2 4BH, UK

David Dodwell, Nuffield Department of Population Health, University of Oxford, Oxford, OX3 7LF, UK

Bridget Hilton, NHS England and NHS Improvement, 23 Stephenson Street, Birmingham, B2 4BH, UK

Isabella Stevens-Harris, University Hospitals of Derby & Burton NHS Foundation Trust, Derby DE22 3NE, UK

Sarah E Pinder, School of Cancer & Pharmaceutical Sciences, King's College London and Guy's and St Thomas' Hospitals NHS Foundation Trust, London, SE1 9RT, UK

Matthew G Wallis, Cambridge Breast Unit, Cambridge University Hospitals NHS Foundation Trust, Cambridge & NIHR Cambridge Biomedical Research Centre, Cambridge, CB2 0QQ, UK

Anthony Maxwell Nightingale Centre, Wythenshawe Hospital, Manchester University NHS Foundation Trust, Southmoor Road, Manchester, M23 9LT, UK

Olive Kearins, NHS England and NHS Improvement, 23 Stephenson Street, Birmingham, B2 4BH, UK

Mark Sibbering, University Hospitals of Derby & Burton NHS Foundation Trust, Derby, DE22 3NE, UK

Abeer M Shaaban, Queen Elizabeth Hospital Birmingham and University of Birmingham, Birmingham, B15 2GW, UK

Cliona Kirwan, Division of Cancer Sciences, Faculty of Biology, Medicine and Health, University of Manchester, Manchester, M13 9PT, UK



For peer review only - http://bmjopen.bmj.com/site/about/guidelines.xhtml

Nisha Sharma, Leeds Wakefield Breast Screening Service, Seacroft Hospital, York Road, Leeds, LS14 6UH, UK

Hilary Stobart, Independent Cancer Patients' Voice, 17 Woodbridge Street, London, EC1R 0LL, UK

Joanne Dulson-Cox, NHS England and NHS Improvement, 23 Stephenson Street, Birmingham, B2 4BH, UK

Janet Litherland, West of Scotland Breast Screening Centre, Nelson Mandela Place, Glasgow, G2 1QY, UK

Senthurun Mylvaganam, The Royal Wolverhampton Hospitals NHS Trust, New Cross Hospital, Wolverhampton Road, Wolverhampton, WV10 0QP, UK

Elena Provenzano, Cambridge University Hospitals NHS Foundation Trust, Cambridge & NIHR Cambridge Biomedical Research Centre, Cambridge, CB2 0QQ, UK

Elinor Sawyer, School of Cancer & Pharmaceutical Sciences, King's College London and Guy's and St Thomas' Hospitals NHS Foundation Trust, London, SE1 9RT, UK

Alastair M Thompson, Dan L Duncan Comprehensive Cancer Center, Baylor College of Medicine, Houston, Texas 77030, US

**Corresponding author**

Ms Karen Clements, Screening QA Service, NHS England and NHS Improvement, 2nd Floor, 23 Stephenson Street, Birmingham, B2 4BH, UK

Email: karen.clements@nhs.net



For peer review only - http://bmjopen.bmj.com/site/about/guidelines.xhtml

## ABSTRACT

### Purpose

The introduction of breast screening in the UK led to an increase in the detection of non-invasive breast neoplasia, predominantly ductal carcinoma in situ (DCIS), a non-obligatory precursor of invasive breast cancer. The Sloane Project, a UK prospective cohort study of screen-detected non-invasive breast neoplasia, commenced in 2003 to evaluate the radiological assessment, surgical management, pathology, adjuvant therapy and outcomes for non-invasive breast neoplasia. Long-term follow-up and accurate data collection are essential to examine the clinical impact. Here, we describe the establishment, development and analytic processes for this large UK cohort study.

### Participants

Women diagnosed with non-invasive breast neoplasia via the UK National Health Service Breast Screening Programme (NHSBSP) from 01 April 2003 are eligible, with a minimum age of 46 years. Diagnostic, therapeutic and follow-up data collected via proformas, complement date and cause of death from national data sources. Accrual for patients with DCIS ceased in 2012 but is ongoing for patients with epithelial atypia/in situ neoplasia, while follow-up for all continues long-term.

### Findings to date

To date, patients within the Sloane cohort comprise a third of those diagnosed with DCIS within the NHSBSP and are representative of UK practice. DCIS has a variable outcome and confirms the need for longer-term follow-up for screen-detected DCIS. However, the radiology and pathology features of DCIS can be used to inform patient management. We demonstrate validation of follow-up information collected from national datasets against traditional, manual, methods.

### Future plans




For peer review only - http://bmjopen.bmj.com/site/about/guidelines.xhtml

Conclusions derived from the Sloane Project are generalisable to women in the UK with screen-detected DCIS. The follow-up methodology may be extended to other UK cohort studies and routine clinical follow-up. Data from English patients entered into the Sloane Project are available on request to researchers under data sharing agreement. Annual follow-up data collection will continue for a minimum of 20 years.

For peer review only



For peer review only - http://bmjopen.bmj.com/site/about/guidelines.xhtml

## ARTICLE SUMMARY

### Strengths and limitations of this study

- Large national prospective cohort study with high quality clinical data and standardised reporting
- Long-term follow-up data collection allows for the detection of subsequent cancers and analysis of outcome data
- Data from the Sloane Project can be utilised by researchers to improve knowledge about DCIS and atypia and help to address concerns about overdiagnosis and overtreatment.
- Development of follow-up methodology using routinely collected data to identify further events could be generalised to other cohort studies, clinical trials, and cancer sites
- Coding in other linked datasets may be inaccurate leading to over-estimation of relative proportion of DCIS recurrences
- Participation was voluntary and 100% case capture was not achieved


For peer review only - http://bmjopen.bmj.com/site/about/guidelines.xhtml

## INTRODUCTION

The United Kingdom (UK) National Health Service Breast Screening Programme (NHSBSP) was established in 1988, originally to screen women aged 50-64 every three years using single view mammography. The NHSBSP now comprises individual programmes in each of the four devolved nations which have developed since the inception of UK-wide breast screening. Following the extension of the eligible age range to 70 years in England in 2002, Scotland in 2003-2004, Wales in 2006 and Northern Ireland in 2009, the NHSBSP now routinely invites all women in the UK from the age of 50 to their 71st birthday to attend breast screening every three years.[1] In some areas of the UK, women aged 47 to 49 and/or 71 to 73 received invitations for screening as part of an age extension trial[2] and some women received an invitation at the age of 46 as they were in their 47th year. Women from their 71st birthday onwards can self-refer every three years. Two-view mammography was introduced in Northern Ireland in 1989, Wales in 2001[3], England in 2003, and in Scotland in 2008/09 and is now used in all centres.[4] Analogue mammograms were replaced by digital mammography from 2008, with 99% of UK NHSBSP units able to perform digital mammography as of October 2013.[5] Digitisation of NHSBSP mammography was completed in 2015.

The introduction of breast screening in the UK led to an increase in the detection of non-invasive breast neoplasia, predominantly ductal carcinoma in situ (DCIS).[6] A final diagnosis of DCIS now accounts for 20% of screen-detected breast cancer [7] and 11% of all breast cancers diagnosed in women aged 50 years and older.[8] A diagnosis of DCIS poses a difficult problem for patients and clinicians since there remains uncertainty about the natural history, rate of progression, invasive potential, optimal treatment and follow-up protocols, which has led to concerns about overdiagnosis and overtreatment.[6]

Some DCIS progresses to invasive breast cancer, but the risk varies with features of DCIS.[6,9]



For peer review only - http://bmjopen.bmj.com/site/about/guidelines.xhtml

Three randomised trials and one single arm trial of active surveillance versus guideline-concordant surgery for 'low-risk' DCIS have been implemented, to investigate concerns about overdiagnosis and overtreatment.[10,11,12,13] In addition, an international study is using a wide range of approaches to address the issues of overdiagnosis and overtreatment as part of the Cancer Grand Challenge.[14]

An increased risk of both contemporaneous and subsequent invasive breast cancer is also seen in some other screen-detected lesions of uncertain malignant potential such as atypical intraductal epithelial proliferation (AIDEP)/atypical ductal hyperplasia (ADH), flat epithelial atypia (FEA) and lobular in situ neoplasia (LISN). However, prospective data on their management and outcomes are lacking, supporting the need for large, prospective studies, as data currently available are based on historical trial data[15,16,17] or retrospective studies.[18,19,20,21]

The Sloane Project, established in memory of Professor John Sloane, a breast pathologist,[22] commenced in 2003 as a UK-wide prospective cohort study of non-invasive breast neoplasia detected within the NHS Breast Screening Programme (NHSBSP). The original aim was to accrue a cohort of 10,000 women with screen-detected DCIS and subsequently to inform optimal radiological assessment, surgical management, pathology handling and reporting (including the features of greatest clinical and prognostic importance) and adjuvant therapy.

The potential value of this cohort study has been recognised as providing evidence for clinical practice by the National Institute of Clinical Excellence (NICE), who recommended that screen-detected DCIS patients be entered into the Sloane Project in the guideline "Diagnosis and treatment of early breast cancer, including locally advanced disease" published in February 2009.[23] More recently, the Independent Breast Screening Review[6] recommended "continued support for the Sloane Project which has an extensive database of screen-detected cases of



For peer review only - http://bmjopen.bmj.com/site/about/guidelines.xhtml

DCIS and the long term follow-up of these cases may well improve our understanding of this condition".

This manuscript describes the Sloane Project process of case ascertainment, data cleaning and, specifically, methodology for verification and analysis of primary information and follow-up data. The latter elements, in particular, may be widely applicable for analyses of other large national datasets.

## COHORT DESCRIPTION

The first phase of the Sloane Project collected data on women diagnosed with screen-detected non-invasive breast neoplasia from 01 April 2003 to 31 March 2012. The ongoing second phase is continuing to collect information on patients with epithelial atypia/in situ neoplasia, but excluding DCIS, and commenced on 01 April 2012. The collection of follow-up and mortality data is an ongoing process and continues for all Sloane Project patients. The minimum age for study entry was 46, with no maximum age.

### Eligibility

Eligibility for entry into the Sloane Project is indicated in Figure 1.

**Figure 1: Sloane eligibility flowchart**

### Case Ascertainment

The Sloane prospective cohort study is based on patient-level data collected by the NHS as part of the care and support of patients and is voluntarily submitted by UK NHSBSP units. Sloane Project cases were therefore matched to the data collected through the annual NHSBSP/ Association of Breast Surgery (ABS) breast screening audit[24] a national audit of all patients diagnosed through the NHSBSP, primarily designed to assess surgical performance with



For peer review only - http://bmjopen.bmj.com/site/about/guidelines.xhtml

surgical Quality Assurance Standards, for the period 01 April 2003 to 31 March 2012, to understand the level of case ascertainment.

**Governance**

The project is managed, and the data are collated, maintained and quality assured by the Screening Quality Assurance Service (SQAS), based in Birmingham, UK. It is overseen by a Project Steering Group (see Acknowledgements) comprising radiologists, pathologists, surgeons, managers, statisticians, oncologists, and patient advocates.

Ethics Committee approval was assessed as not required for this study which was originally conducted under the NHS Cancer Screening Programme's application to the Patient Information Advisory Group (PIAG). More recently, the study has been permitted to process personally identifiable data without consent under Regulation 5 of Statutory Instrument 2002 No. 1438: The Health Service (Control of Patient Information) Regulations 2002[25] (15/CAG/0207) in line with the following clause: “quality assuring screening services to ensure they are effective and safe, and that any incidents are investigated and managed appropriately”. This statutory exemption to common law permits the processing of personally identifiable data, as part of the core remit of population screening.

Data are securely held within the organisation with patient identifiers recorded. There is restricted access to a certified group of individuals and a full audit trail for access. A System Level Security Policy is in place to cover the Sloane Project as a System Asset as well as a Data Protection Impact Assessment. Figure 2 describes example data flows in and out of the Sloane Project, along with the controls in place to mitigate for risks.

**Figure 2: Example data flow diagram and key for Sloane Project data**



For peer review only - http://bmjopen.bmj.com/site/about/guidelines.xhtml

**Patient and Public involvement**

Patients from Independent Cancer Patients' Voice (ICPV)[26] joined the Sloane Project steering group in 2012. Since this time, they have actively contributed to publications guidance, communications, and promotional material, including the website, as well as supporting funding applications. They are also involved, as part of a wider international collaboration, with the research that has followed from the cohort study.

Clinical follow-up data from the English Programme are accessed via a Data Sharing Agreement (DSA) between the National Disease Registration Service (NDRS) and SQAS. Follow-up and mortality data from Scotland, Wales and Northern Ireland need specific permissions. The agreements (from these 'devolved nations') do not allow for further onward sharing of data at present. Apart from the limitation described above, access to the Sloane Project data from external parties is governed by application to the breast screening Research Advisory Committee (RAC) and Office for Data Release (ODR). Data will only be released by the Sloane Project to researchers under approval and in an anonymised or depersonalised format and under a data sharing agreement.

**Primary data capture**

Demographic data and data deriving from radiology, pathology, surgery and adjuvant treatments at the time of diagnosis and initial treatment were originally captured via individual, pathology, surgery and radiotherapy (RT) paper proformas (see Supplementary File 1), collected at NHSBSP unit and hospital level. More recently, the proformas have been shortened and combined into one form, which can be completed electronically (see Supplementary File 2).[27] The forms are submitted to the Sloane Project team and data are then entered on a secure database held on an SQL server that generates an individual patient and tumour identifier.



For peer review only - http://bmjopen.bmj.com/site/about/guidelines.xhtml

As all Sloane patients are diagnosed within the NHSBSP, adherence to NHSBSP guidelines[28] and participation in the relevant quality assurance programmes[29] are mandatory. Participating units are required to follow a pathology protocol (see Supplementary Files 3 and 4)[30] that includes guidance on the handling and reporting of specimens, definitions for DCIS and atypia, microinvasion, cytonuclear grade, comedo necrosis and assessment of excision margins to NHSBSP pathology standards.[31] Radiology guidelines (see Supplementary Files 3 and 4)[32] encourage participating radiologists to complete the radiology proforma. Radiologists participate in the NHSBSP Personal Performance in Mammographic Screening (PERFORMS) external quality assurance scheme.[33]

**Data Handling**

Data are manually transcribed into the database, which has built in integrity checks run on a regular basis, along with extra validation checks for each subsequent data request. Missing data items are subsequently added as far as possible. If the data cannot be sourced from information already supplied or from the NHSBSP unit or other data sources, then unknown/unrecorded status is assigned. Best efforts are made to minimise the number of missing data items (see Supplementary File 5).

Validation of data is undertaken by cross-checking with original NHSBSP unit source documents for those patients with recurrence and more generally, for the overall dataset, against the Association of Breast Surgery (ABS) national audits.[24] The Sloane Project data, as completed by clinicians, are taken as the primary source when there is conflicting information. However, the ABS national audit is used to supplement missing data items.



For peer review only - http://bmjopen.bmj.com/site/about/guidelines.xhtml

## Classification of subsequent events

Given the potential difficulties in distinguishing local recurrence from a new primary lesion in the same breast, terminology has been developed to classify subsequent events (Supplementary File 6 Supplemental Table 1).

## Death classification

Date and cause of death have historically been obtained directly from the NHS Trusts or Health Boards. Since 2013, the majority are now sourced from the National Disease Registration Service (NDRS)/NHS Wales Cancer Network Information System Cymru (CaNISC) data provided by the Office for National Statistics (ONS),[34] National Records of Scotland [35] and Northern Ireland patient records. Rules for coding the underlying cause of death are based on the rules used by ONS[34] that apply the condition or conditions entered in the lowest completed line of Part I of the Medical Certificate Cause of Death (MCCD). Women who die of breast cancer but who have no intervening breast events recorded are deemed to have had distant metastases on the date they died. The definitions for cause of death in Supplementary File 7 Supplemental Table 2 are used for the Sloane dataset.

## Follow-up and recurrence data capture

A methodology has been developed to identify further events, including ipsilateral breast/nodal, contralateral breast/nodal, and distant events. This is run annually for the English patients, who comprise 87.1% of the Sloane cohort, and on an *ad hoc* basis for those from Scotland, Wales and Northern Ireland, who make up 10.1%, 2.5% and 0.3% of patients respectively.

Further events are identified by matching women by their unique NHS number (in England and Wales) and Community Health Index (CHI) number (in Scotland and Northern Ireland) and



For peer review only - http://bmjopen.bmj.com/site/about/guidelines.xhtml

information provided by NHSBSP units to routinely collected UK datasets, as detailed in Table 1.

| **Country** | **Dataset utilised for Sloane follow-up data** | **Data collected** | **Data items used in Sloane English patient follow-up methodology** |
|---|---|---|---|
| England (National Disease Registration Service incorporating the National Cancer Registration and Analysis Service - NCRAS) [36] | English Cancer Analysis System (CAS) | Cancer Registration data including diagnosis, treatment and tumour characteristics | date of diagnosis/treatment, trust/provider of diagnosis/treatment, ICD-10, morphology and behaviour, laterality, nodal positivity, adjuvant treatment, OPCS-4 |
| | Hospital Episode Statistics (HES) admitted patient care[37] | Diagnosis and treatment | date of admission/operation/episode start, ICD-10, OPCS-4, laterality, provider code |
| | Cancer Waiting Times (CWT)[38] | Diagnosis and treatment | date first seen/treatment start date, ICD-10, laterality, cancer treatment modality, cancer or symptomatic breast referral patient status, CTE type, and metastatic site |
| | English National Radiotherapy Dataset (RTDS)[39] (2009 onwards) | Adjuvant radiotherapy | treatment/appointment start date, treatment region, ICD-10 |



For peer review only - http://bmjopen.bmj.com/site/about/guidelines.xhtml

| | | | |
|---|---|---|---|
| | Systemic Anti-Cancer Therapy dataset (SACT) [40] | Adjuvant and neo-adjuvant chemotherapy | organisation code, ICD-10, morphology, drug treatment intent, start date, drug group |
| | Office for National Statistics (ONS) mortality data[34] | Date and Cause of death (diagnosed cancer patients) | |
| | Mortality and Birth Information System (MBIS) | Date and cause of death (diagnosed atypia patients) | |
| Scotland – Information Services Division of the Scottish government (ISD) and National Services Scotland (NSS) [41] | Cancer registration data | Diagnosis, treatment, tumour characteristics, date of death and cause of death | |
| Wales – Cancer Network Information System (CaNISC) [42] | Cancer registration data | Diagnosis, treatment, tumour characteristics, date of death and cause of death | |
| Northern Ireland | Sloane contact provides data from hospital records and systems on relatively small cohort of patients | Diagnosis, treatment, tumour characteristics, date of death and cause of death | |



For peer review only - http://bmjopen.bmj.com/site/about/guidelines.xhtml

**Table 1: National datasets provided to record follow-up and death information**

The current version of the International Classification of Diseases and Related Health Problems ICD-10[43] was used when ascertaining diagnostic codes alongside OPCS-4[44] when ascertaining procedure codes.

The censor date used in time-related analyses is the earliest of date of death, date of first further event (DCIS, invasive breast cancer, metastatic breast cancer) or end of time period of matched datasets if no further event has occurred and the woman is still alive.

## Follow-up methodology

The follow-up methodology was developed to test the proof of concept of utilising the English NCRAS data to identify further events and decrease the burden of local data collection within NHSBSP units and NHS Trusts

The methodology is based on the following variables from the Sloane database:

- date of mammogram
- date of surgery
- laterality of primary lesion

These are then linked (via NHS number) to patients registered within the datasets and data items as described in Table 1.

## Validation of the follow-up methodology

The methodology was validated on a subset of 3,711 (39%) of the cohort of Sloane patients for whom recurrence information was independently collected directly from the NHSBSP units prior to the generation of the methodology utilising NCRAS data from 2013. These data were



For peer review only - http://bmjopen.bmj.com/site/about/guidelines.xhtml

compared with NCRAS data focussing on invasive status (invasive/non-invasive/distant), laterality and date of diagnosis for the primary and any further event.

To confirm a match of either primary or recurrence, data were considered to be:

- *acceptable* – same laterality, same invasive status, date of diagnosis within 6 months (either prior or after)
- *not acceptable* – different laterality, different invasive status (with the exception of microinvasion which is coded as invasive on CAS), dates >12 months apart
- *query acceptable* – e.g. dates different between 6 and 12 months; for example, in one case the diagnosis date and NCRAS chemotherapy date (the only event) differed by 8 months, which was considered to be acceptable.

In order to support this proof of concept project and provide further validation, we sent a list of 100 Sloane Project patients to University Hospitals of Derby and Burton NHS Foundation Trust (UHDB). Some of these patients we had identified as having a further event using the methodology. The Trust was blinded to our records for each patient and was requested to identify those who they believed to be patients with a further event, and provide date and type, but also to indicate those who had not had a further event according to their records.

## FINDINGS TO DATE

To date, several papers[9, 45,46,47,48,49,50,51,52,53,54,55,56] have been published on the Sloane cohort, which have shown that the radiology and pathology features of DCIS can be used to inform patient management. Outcome data also show that DCIS is not the indolent disease often described, and the need for longer term follow-up for screen detected DCIS has been confirmed.

### Case Ascertainment



For peer review only - http://bmjopen.bmj.com/site/about/guidelines.xhtml

Overall case ascertainment ranged from 0% to 93% by NHSBSP unit (median 32%, mean 38%). Individual attributes (including patient, tumour and treatment characteristics), such as age at screening (Table 2), cytonuclear grade of DCIS, DCIS size, Oestrogen Receptor (ER) status, surgical and adjuvant treatment have been shown to be representative when comparing the voluntary Sloane prospective cohort study with the compulsory ABS/NHSBSP annual screening audit, although the more detailed Sloane data represents 38% of patients with DCIS diagnosed through the NHSBSP over the same time period.

| **Age at screening mammogram** | **Sloane cohort (%)** | **ABS/NHSBSP cohort (%)** |
|---|---|---|
| <50 | 2.8 | 3.1 |
| 50 – 54 | 25.1 | 25.1 |
| 55 – 59 | 21.4 | 21.1 |
| 60 – 64 | 23.5 | 23.3 |
| 65 – 69 | 19.9 | 19.9 |
| 70+ | 7.3 | 7.5 |
| Total (known age at mammogram) | 100 | 100 |

**Table 2: Age at screening mammogram for patients in the Sloane cohort compared with patients in the ABS cohort (p=0.81)**

**Primary data completeness**

Data completeness, assessed against a pragmatic desired completeness level of ≥95%, demonstrated the majority of key data items for the DCIS patients within the Sloane Project database have excellent completeness levels. Data completeness was high for most data fields (Table 3).

| **Data item** | Data completeness of related field/s (%) of forms returned |
|---|---|
| **Patient details** | |
| Date of birth | 100.0 |



For peer review only - http://bmjopen.bmj.com/site/about/guidelines.xhtml

| | |
|---|---|
| NHS / CHI Number | 100.0 |
| Hospital Number | 96.0 |
| Screening Number | 99.6 |
| Hospital of diagnosis or treatment | 98.2 |
| Laterality of primary | 99.7 |
| **Radiology** | **97.9** |
| Site | 97.5 |
| Background Pattern | 97.5 |
| Predominant Radiological Feature | 98.1 |
| Presence/absence of microcalcification | 97.8 |
| Type of calcification | 99.0 |
| Radiological size | 98.1 |
| Breast volume | 96.4 |
| Specimen X-Ray (cores of diagnostic surgical specimen) | 83.8 |
| **Treatment** | **99.0** |
| Recorded presence/absence of pre-operative diagnosis of DCIS | 93.0 |
| Surgical procedure (or no surgery confirmed) | 99.0 |
| Date of surgical procedure (where relevant) | 99.8 |
| Axillary procedure done/not done | 97.1 |
| Axillary procedure type (where relevant) | 99.8 |
| Adjuvant therapy (whether adjuvant therapy given or not) | 99.2 |
| **Pathology** | **97.6** |
| Core/vacuum-assisted biopsy | 81.6 |
| Surgical specimen type | 96.2 |
| Final Histological diagnosis overall (from excision if excision done, from core if only core done) | 97.3 |
| Cytonuclear grade on surgical specimen (DCIS) | 99.2 |
| Architectural growth Pattern (DCIS) | 91.7 |



For peer review only - http://bmjopen.bmj.com/site/about/guidelines.xhtml

| | |
|---|---|
| Size (DCIS) | 98.4 |
| Presence/absence of Comedo Necrosis (DCIS) | 89.7 |
| Presence/absence of Microinvasion (DCIS) | 97.4 |
| Radial margin status (breast conserving surgery (BCS) + DCIS) | 94.1 |
| ER status (positive, negative or unknown [ER not done]) | 85.4 |
| PR status (included positive, negative or unknown [not done]) | 78.7 |
| HER2 status (included positive, negative or unknown [not done]) | 73.6 |
| **Radiotherapy (yes/no)** | **85.3** |
| Dose (where relevant) | 95.2 |
| Energy (where relevant) | 91.8 |
| Number of fractions (where relevant) | 94.5 |

**Table 3: Data completeness for key primary data items (DCIS patients)**

**Follow-up methodology validation results**

The results of the initial validation of the follow-up methodology, whereby recurrence (presence or absence of) information independently collected by Sloane was compared with routinely collected datasets from NCRAS are shown in Table 4 and 5.

| **NCRAS follow-up (using follow-up methodology)** | **Direct Sloane follow-up (n=3711)** | |
|---|---|---|
| | **No. of patients identified with further event in Sloane (n=211) (% of events identified from Sloane)** | **No. of patients without further event in Sloane (n=3500) (% of patients without further event from Sloane)** |
| **No. of patients identified with further event in NCRAS (n=301)** | 193 (91.5%) [AM] | 108 (3.1%) [NAM] |
| **No. of patients identified without further event in NCRAS (n=3377)** | 4 (1.9%) [NAM] | 3373 (96.4%) [AM] |



For peer review only - http://bmjopen.bmj.com/site/about/guidelines.xhtml

| | | |
|---|---|---|
| **No. of patients identified with further event different between the datasets (n=18)** | 14 (6.6%) NAM | 4 (0.1%) NAM |
| **No. of patients not recorded on the NCRAS database (either primary or further event) (n=15)** | 0 (0%) NAM | 15 (0.4%) NAM |

AM = Acceptable match NAM = Not acceptable match

For peer review only

**Table 4: Validation of the follow-up methodology for identifying events / disease-free status (total number patients = 3711), comparing direct Sloane follow-up (211 patients with events identified) versus NCRAS follow-up (319 patients with events identified).**

Table 4 shows that of 3,711 Sloane DCIS patients for whom follow-up data was received direct from the NHSBSP units, 91.5% were also in the NCRAS datasets and were an 'acceptable match'. There was a 96.4% match between the Sloane and NCRAS datasets on fact of 'no further event'. 108 further events were identified through NCRAS datasets that had not been identified by the NHSBSP units. Further checking with the hospitals at which the event occurred confirm that these are correct. There are likely to be a number of reasons for this such as the patient's further event being diagnosed at a different NHS Trust or Health Board, or the type of hospital systems that were checked for further event and the likelihood of finding the information on those systems.

| First Sloane further event | Number of Sloane patients identified with further event | Number matched to NCRAS datasets ('acceptable match') |
|---|---|---|
| Ipsilateral non-invasive | 84 | 70 (83%) |



For peer review only - http://bmjopen.bmj.com/site/about/guidelines.xhtml

| | | |
|---|---|---|
| Ipsilateral invasive | 56 | 54 (96%) |
| Ipsilateral distant (with invasive/non-invasive diagnosed at same time) | 2 | 2 (100%) |
| Distant | 6 | 5 (83%) |
| Contralateral non-invasive | 18 | 17 (94%) |
| Contralateral invasive | 42 | 42 (100%) |
| Contralateral invasive and distant (with invasive at same time) | 1 | 1 (100%) |
| Bilateral non-invasive | 2 | 2 (100%) |
| **Total** | **211** | **193 (91%)** |

**Table 5: Number of Sloane patients identified with a recurrence reported directly from NHSBSP units that matched/did not match to the same recurrence in the NCRAS datasets (when applying the Sloane follow-up methodology).**

Table 5 shows more detail about those 18 recurrences not matched according to our rules by NCRAS datasets. Of these, 11 were identified in HES but the invasive status was recorded incorrectly (9/11) or laterality was incorrect (2/11). Five were not identified in HES because two had surgery performed outside the NHS, one had no surgery due to co-morbidity, two remained unknown, although may have had their surgery in the private sector. Two were not found on CAS or HES but were identified on the CWT dataset, but invasive status was incorrect.

We concluded that although 92% events were successfully matched for further event correctly on NCRAS datasets, a further 13 recurrences (6%) would now be picked up by recent improvements in NCRAS and linkage to other datasets. It is likely that using currently available matching to national data sets, 206/211 patients (97%) would have accurate identification of



For peer review only - http://bmjopen.bmj.com/site/about/guidelines.xhtml

DCIS or invasive breast cancer recurrence on the same side, contralateral breast or distant metastatic disease.

The further validation on 100 UHDB patients showed that, using the Sloane follow-up methodology, 20 matched exactly for further events, 75 matched as having no further events and five did not match. Thus 95% of patients were correctly matched. Out of the 5 that did not match 3 were because the Sloane methodology had a resulted in a “query further event” and UHDC confirmed “no further event”, 1 was “unknown invasive status” and UHDB confirmed “invasive” and 1 was recorded as “ipsilateral DCIS” whereas UHDB had it recorded as “ipsilateral invasive”. These results can be used to improve the methodology.

## STRENGTHS AND LIMITATIONS

### Strengths

Long term follow-up of patients is required to ensure the impact of diagnosis, treatment and subsequent care is optimised and changes in management evaluated. Large, prospective cohort studies of conditions such as DCIS where subsequent events are relatively rare and occur over decades present particular challenges. Even for smaller cohorts and especially for clinical trials, the benefits of accurately matching prospectively collected data to national data sets may be invaluable. The ambitious, prospective, long term Sloane Project has identified and followed some 15,000 patients with NHSBSP detected pre-invasive breast neoplasia over almost two decades. We demonstrate here a high level of concordance between subsequent disease events confirmed in the Sloane cohort data (provided directly by NHSBSP units) and those identified using a follow-up methodology applied to electronically stored national datasets. While events were rare, representing only 2% of the total cohort, concordance between data sources was strong. There was also a 96% level of concordance for freedom from further

For peer review only

events. This is encouraging but requires future validation, before the use of NCRAS data to determine freedom from further events, can be regarded as definitive.

This methodology, if validated in other settings, and potentially further refined, may be generalisable to other cohort studies and for clinical trial follow-up. The long-term follow-up of patients beyond an initial 5 years is increasingly important in early invasive breast cancer, as well as DCIS, given the low frequency of events over an extended time period in these groups.

Although Sloane patients comprise a third of those diagnosed with DCIS within the NHSBSP during the relevant time period, the approach laid out here confirms that the data quality and completeness are high and appear to be representative of the overall population of patients with screen-detected DCIS in the UK. Thus, conclusions derived from analyses of care and clinical outcomes from the Sloane Project[9, 45-56] are robust and generalisable to all women in the UK with screen-detected DCIS. This is supported by a recently published study of over 35,000 women with screen-detected DCIS treated within the UK NHSBSP from its start until 2014.[57] Patient, histopathology, treatment and outcomes reported reflect those curated within the more detailed Sloane cohort.

**Limitations**

Participation in data provision by NHSBSP units in the Sloane Project is voluntary, not specifically funded and 100% case capture was not achieved. However, comparison with the mandatory ABS/NHSBSP annual audits, which collect less detailed data, demonstrates that the Sloane dataset is representative of a cross section of patients, geographic spread and of NHSBSP units' practice at the time of accrual.

Engagement has been challenged by lack of resources, particularly NHS staff time, available for long-term for data collection. However, staff participants in the project receive CPD credits and



For peer review only - http://bmjopen.bmj.com/site/about/guidelines.xhtml

certificates for their participation in primary and follow-up data capture which is useful for annual appraisal and revalidation. The Sloane Project DCIS database remains open for completion of data for those cases with missing information (and is currently over 94% complete).

Additional challenges include different health service structures and processes across England, Scotland, Wales and Northern Ireland. Data regarding the small proportion of patients treated outside the NHS are limited. Furthermore, coding of DCIS in some datasets may be inaccurate, resulting in a potential over-estimation of the relative proportion of DCIS that is recurring as invasive disease compared to that recurring as DCIS.

## COLLABORATION

The Sloane Project group welcomes applications from UK, EEA and international organisations to collaborate and release data. However, any data releases are subject to a common governance framework (as summarised in Figure 2), which will ensure the correct confidentiality provisions, legal permissions and ethical approvals are adhered to. At the time of writing, only Sloane data from the English NHS Breast Screening Programme are available to researchers.

## CONCLUSIONS

Overall, the Sloane prospective cohort study continues to evaluate changes in practice and outcomes for patients with DCIS as a result of publications of its own findings [55, 56] as well as others' research[58,59] and national guidelines and recommendations, in order to ascertain the impact of such variations. Extending the value of this prospective cohort study further, the data collected have been used to develop and support innovative research proposals examining the underlying biological and imaging features of DCIS.[60, 61] These peer-reviewed, grant-funded studies generated, for example, molecular data for comparison with the high quality clinical data from the Sloane Project obtained through the methodology described here to investigate



For peer review only - http://bmjopen.bmj.com/site/about/guidelines.xhtml

mechanisms behind the progression of DCIS to invasive breast cancer. [62] Thus, the NHSBSP prospective cohort study of non-invasive breast neoplasia, particularly DCIS, (the Sloane Project)[27] continues to deliver insights into the clinical management of these conditions and resultant patient outcomes over time and can help address concerns about overdiagnosis and overtreatment.[10,63] Development of cross-validation of primary data, particularly patient outcomes, with the increasingly informative national datasets points to the generalisability of this approach.

**Word count (3698)**

For peer review only



For peer review only - http://bmjopen.bmj.com/site/about/guidelines.xhtml

**ACKNOWLEDGEMENTS**

This work uses patient data collected by the NHS as part of their care and support. The English data is collated, maintained and quality-assured by the Screening Quality Assurance Service (SQAS) and follow-up data by the National Cancer Registration and Analysis Service

**Sloane Project Steering Group**

Professor Alastair Thompson (Chair of Sloane Steering Group) – Professor of Surgery, Baylor College of Medicine, Houston, Texas

Mrs Samantha Brace-McDonnell - Patient Advocate, Independent Cancer Patients' Voice

Mrs Karen Clements - Breast Cancer Research Manager, NHS England and NHS Improvement

Professor Sarah Darby - Professor of Medical Statistics, University of Oxford

Dr David Dodwell – Consultant Clinical Oncologist, University of Oxford, Oxford

Ms Joanne Dulson-Cox - National Audit Project Senior QA Officer, NHS England and NHS Improvement

Mrs Bridget Hilton - National Audit Project Senior QA Officer, NHS England and NHS Improvement

Mrs Olive Kearins – Quality Assurance Director, NHS England and NHS Improvement

Miss Cliona Kirwan - NIHR Clinician Scientist in Surgical Oncology and Consultant Oncoplastic Breast Surgeon, University Hospital of South Manchester

Dr Janet Litherland - Consultant Radiologist, West of Scotland Breast Screening Programme, Glasgow

Dr Anthony Maxwell - Consultant Radiologist, University Hospital of South Manchester, Manchester

Mr Seni Mylvaganam - Consultant Oncoplastic Breast Surgeon, Royal Wolverhampton Hospital

Professor Sarah Pinder - Professor of Breast Pathology, Guys and St Thomas' Hospitals, London

Dr Elena Provenzano - Lead Breast Histopathologist, Addenbrooke's Hospital, Cambridge

Professor Elinor Sawyer – Consultant Clinical Oncologist, Guys Hospital, London

Dr Abeer Shaaban - Consultant Pathologist, University Hospitals Birmingham

Dr Nisha Sharma - Lead Clinician Radiology, Leeds Teaching Hospital NHS Trust

Ms Hilary Stobart - Patient Advocate, Independent Cancer Patients' Voice

Dr Matthew Wallis - Consultant Radiologist, Addenbrooke's Hospital, Cambridge



For peer review only - http://bmjopen.bmj.com/site/about/guidelines.xhtml

We are also grateful for the help, support and professional advice provided by previous members of the Sloane Project Steering Group - Mr Hugh Bishop (previous Chair), Dr Julian Adlard, Miss Jessamy Bagenal, Mr Robert Carpenter, Professor John Dewar, Professor Ian Ellis, Professor Adele Francis, Professor W D George, Professor Andrew Hanby, Professor Sunil Lakhani, Dr Gill Lawrence, Mr Martin Lee, Dr James Macartney, Mr Stewart Nicholson, Professor Julietta Patnick CBE, Dr Gillian Ross, Mr Mark Sibbering, Professor Valerie Speirs, Ms Sheila Stallard, Dr Jeremy Thomas, Professor Ian Tomlinson, Mrs Margot Wheaton, Ms Maggie Wilcox.

**All participating UK NHSBSP units**

Avon
Aylesbury and Wycombe
Barnsley
Bedfordshire and Hertfordshire
Bolton
Breast Test Wales – North
Breast Test Wales – South East
Breast Test Wales – South West
Brighton
Cambridge and Huntingdon
Central & East London
Chelmsford and Colchester
Chester
City, Sandwell and Walsall
Cornwall
Crewe
Derby City and South Derbyshire
Doncaster
Dorset
Dudley and Wolverhampton
East Berkshire
East Cheshire & Stockport
East Lancashire

For peer review only



For peer review only - http://bmjopen.bmj.com/site/about/guidelines.xhtml

East Scotland

Gateshead

Gloucestershire

Great Yarmouth and Waveney

Greater Manchester

Guildford

Hereford and Worcester

Humberside

Isle of Wight

King's Lynn

Leeds and Wakefield

Leicestershire

Liverpool

Medway

Milton Keynes

Newcastle upon Tyne

Norfolk and Norwich

North and Eastern Devon

North and Mid Hampshire

North Derbyshire & Chesterfield

North East Scotland

North Lancs & South Cumbria

North London

North Midlands

North Nottingham

North Yorkshire

Northampton

Nottingham

Outer North East London

Oxford

Pennine

Peterborough

Portsmouth

Rotherham



For peer review only - http://bmjopen.bmj.com/site/about/guidelines.xhtml

Sheffield

Shropshire

Somerset

South Birmingham

South East London

South East Scotland

South Essex

South West London

South West Scotland

Southampton and Salisbury

Surrey (Jarvis)

Warrington

Warwick, Solihull and Coventry

West Berkshire

West Devon

West Essex

West of London

West of Scotland

West Suffolk

Western BSU, Altnagelvin Hospital, Northern Ireland

Wiltshire

Wirral

Worthing (West Sussex)

**Public Health Scotland (previously the Information Services Division Scotland)**

**NHS Wales Cancer Network Information System Cymru (Canisc)**

**Independent Cancer Patients' Voice**

**Funders (as described below)**

**COMPETING INTERESTS**



For peer review only - http://bmjopen.bmj.com/site/about/guidelines.xhtml

KC is currently funded (since 2019) as part of the Cancer Research Grand Challenge PRECISION team (C7827/A27366), which is funded by Cancer Research UK and the Dutch Cancer Society.

DD is funded by Cancer Research UK (grant C8225/A21133)

EP received speaker's honoraria (Roche), Travel costs to speak at meeting (Roche) Participation in Advisory group meetings for IPB Advisors and Roche

SEP participated in Advisory Boards for AstraZeneca, Roche and Exact Sciences, received money for speaking at meetings for Roche and Exact Sciences and is a member of the PRECISION consortium (C7827/A27366).

AS has participated in Advisory Boards for Exact Sciences and Veracyte

HS received travel and support to attend meetings of the CRUK Grand Challenge PRECISION Study (C7827/A27366).

ES, AMT, MGW are members of the PRECISION consortium (C7827/A27366).

BH, ISH, AM, OK, MS, CK, NS, JDC, JL, SM, have nothing to declare.

**CONSENT FOR PUBLICATION**

Not applicable

**AVAILABILITY OF DATA AND MATERIALS**

All data relevant to the study are included in the article or uploaded as supplementary information. No data are available.

**TRANSPARENCY DECLARATION**

The lead author* affirms that this manuscript is an honest, accurate, and transparent account of the study being reported; that no important aspects of the study have been omitted; and that any discrepancies from the study as planned (and, if relevant, registered) have been explained.

**AUTHOR CONTRIBUTIONS:**

Contributorship is included below:

KC (corresponding author) - 1) substantial contribution to the conception and design of the work, the acquisition, analysis, and interpretation of data for the work 2) Drafted and revised the work critically

For peer review only



For peer review only - http://bmjopen.bmj.com/site/about/guidelines.xhtml

DD - 1) substantial contribution to the conception and design of the work, the acquisition, analysis, and interpretation of data for the work 2) Drafted and revised the work critically

BH - 1) substantial contribution to the conception and design of the work, the acquisition, analysis, and interpretation of data for the work 2) Drafted and revised the work critically

ISH - 1) substantial contribution to the acquisition, analysis, and interpretation of data for the work 2) Revised the work critically

SEP - 1) substantial contribution to the conception and design of the work, the acquisition, analysis, and interpretation of data for the work 2) Revised the work critically

MGW - 1) substantial contribution to the conception and design of the work, the acquisition, analysis, and interpretation of data for the work 2) Revised the work critically

AM - 1) substantial contribution to the conception and design of the work, the acquisition, analysis, and interpretation of data for the work 2) Revised the work critically

OK - 1) substantial contribution to the conception and design of the work, the acquisition, analysis, and interpretation of data for the work 2) Revised the work critically

MS - 1) substantial contribution to the acquisition, analysis, and interpretation of data for the work 2) Revised the work critically

AMS - 1) substantial contribution to the acquisition, analysis, and interpretation of data for the work 2) Revised the work critically

CK - 1) substantial contribution to the acquisition, analysis, and interpretation of data for the work 2) Revised the work critically

NS - 1) substantial contribution to the acquisition, analysis, and interpretation of data for the work 2) Revised the work critically

HS - 1) substantial contribution to the acquisition, analysis, and interpretation of data for the work 2) Revised the work critically

JDC -1) substantial contribution to the acquisition, analysis, and interpretation of data for the work 2) Revised the work critically

For peer review only



For peer review only - http://bmjopen.bmj.com/site/about/guidelines.xhtml

JL - 1) substantial contribution to the acquisition, analysis, and interpretation of data for the work 2) Revised the work critically

SM - 1) substantial contribution to the acquisition, analysis, and interpretation of data for the work 2) Revised the work critically

EP - 1) substantial contribution to the acquisition, analysis, and interpretation of data for the work 2) Revised the work critically

ES - 1) substantial contribution to the acquisition, analysis, and interpretation of data for the work 2) Revised the work critically

AMT - 1) substantial contribution to the conception and design of the work, the acquisition, analysis, and interpretation of data for the work 2) Drafted and revised the work critically

The corresponding author confirms that all listed authors meet authorship criteria and that others meeting the criteria are not included.

All authors listed in the manuscript have given final approval of the version to be published and agree to be accountable for all aspects of the work in ensuring that questions related to the accuracy or integrity of any part of the work are appropriately investigated and resolved.

**FUNDING**

At the time of writing, the Sloane Project work is undertaken within the Screening QA Service at NHS England and NHS Improvement (no award/grant number) and the main author is currently funded as part of the Cancer Research Grand Challenge PRECISION team (C7827/A27366).

The Sloane Project has received previous funding from the following:

NHS Breast Screening Programme via Public Health England April 2013 to September 2021, previously NHS – no award/grant number

Breast Cancer Research Trust - 2-year project grant 2008-2009 – no award/grant number

Pfizer 3-year unrestricted educational grant 2005-2007 – no award/grant number

Ad-hoc fundraising events and private donations including Golf Charity Days (organised by Ken Ward), pub quizzes and fundraising balls (2005-2012) – no award/grant number



For peer review only - http://bmjopen.bmj.com/site/about/guidelines.xhtml

The funders of the Sloane Project have not had any involvement in the study design; in the collection, analysis, and interpretation of data; in the writing of the report; or in the decision to submit the article for publication.

For peer review only

## REFERENCES

[1]NHS https://www.nhs.uk/conditions/breast-cancer-screening/when-its-offered/ March 2018. (accessed 20 September 2021)

[2]Age X Trial http://www.agex.uk/about Oxford, 2020 (accessed 20 September 2021)

[3] Osborn GD, Beer H, Wade R, et al. Two-view mammography at the incident round has improved the rate of screen-detected breast cancer in Wales. Clinical Radiology. 2006;61: 478e482

[4] Dibden A, Offman J, Parmar D, et al. Reduction in interval cancer rates following the introduction of two-view mammography in the UK breast screening programme. British Journal of Cancer. 2013;110, 560-564

[5] The National Archives. http://webarchive.nationalarchives.gov.uk/20150506150512/http://cancerscreening.nhs.uk/breastscreen/digital-mammography.html May 2015 (accessed 20 September 2021)

[6] Independent UK Panel on Breast Cancer Screening. The benefits and harms of breast cancer screening: an independent review. Lancet. 2012;380(9855):1778-86.

[7] NHS Breast Screening Programme & Association of Breast Surgery. An audit of screen detected breast cancers for the year of screening April 2018 to March 2019, May 2021.

[8] Healthcare Quality Improvement Partnership (HQIP) National Audit of Breast Cancer in Older Patients (NABCOP): 2020 annual report. https://www.nabcop.org.uk/reports/nabcop-2020-annual-report/; 2020.

[9] Maxwell AJ, Clements K, Hilton B, et al; Sloane Project Steering Group. Risk factors for the development of invasive cancer in unresected ductal carcinoma in situ. Eur J Surg Oncol. 2018 Apr;44(4):429-435. doi: 10.1016/j.ejso.2017.12.007.

[10] Francis A, Thomas J, Fallowfield L, et al. Addressing overtreatment of screen detected DCIS; the LORIS trial. Eur J Cancer 2015;51(16):2296e303.https://doi.org/10.1016/j.ejca.2015.07.017.



For peer review only - http://bmjopen.bmj.com/site/about/guidelines.xhtml

[11] Hwang ES, Hyslop T, Lynch T, et al. The COMET (Comparison of Operative versus Monitoring and Endocrine Therapy) trial: a phase III randomised controlled clinical trial for low-risk ductal carcinoma in situ (DCIS). BMJ Open 2019;9:e026797. https://doi.org/10.1136/bmjopen-2018-026797.

[12] Elshof LE, Tryfonidis K, Slaets L, et al. Feasibility of a prospective, randomised, open-label, international multicentre, phase III, non-inferiority trial to assess the safety of active surveillance for low risk ductal carcinoma in situ - the LORD study. Eur J Cancer 2015;51(12):1497e510. https://doi.org/10.1016/j.ejca.2015.05.008.

[13] Kanbayashi C, Iwata H. Current approach and future perspective for ductal carcinoma in situ of the breast. Jpn J Clin Oncol 2017;47(8):671e7. https://doi.org/10.1093/jjco/hyx059.

[14] van Seijen M, Lips EH, Thompson AM, et al. Ductal carcinoma in situ: to treat or not to treat, that is the question. Br J Cancer 2019;121:285e92. https://doi.org/10.1038/s41416-019-0478-6.)

[15] Donker M, Litie`re S, Werutsky G, et al. Breast-conserving treatment with or without radiotherapy in ductal carcinoma in situ: 15-year recurrence rates and outcome after a recurrence, from the EORTC 10853 randomized phase III trial. J Clin Oncol 2013;31(32):4054e9.

[16] Narod SA, Iqbal J, Giannakeas V, et al. Breast cancer mortality after a diagnosis of ductal carcinoma in situ. JAMA Oncol 2015;1(7):888e96.



For peer review only - http://bmjopen.bmj.com/site/about/guidelines.xhtml

[17] Early Breast Cancer Trialists' Collaborative Group EBCTCG. Overview of the randomized trials of radiotherapy in ductal carcinoma in situ of the breast. J Natl Cancer Inst Monogr 2010;2010(41):162e77.

[18] Sagara Y, Mallory MA, Wong S, et al. Survival benefit of breast surgery for low-grade ductal carcinoma in situ: a population based cohort study. JAMA Surg 2015;150(8):739e45.

[19] Elshof LE, Schaapveld M, Schmidt MK, et al. Subsequent risk of ipsilateral and contralateral invasive breast cancer after treatment for ductal carcinoma in situ: incidence and the effect of radiotherapy in a population-based cohort of 10,090 women. Breast Cancer Res and Treat 2016;159(3):553e63.

[20] Zhou WB, Xue DQ, Liu XA, et al. The influence of family history and histological stratification on breast cancer risk in women with benign breast disease: a meta-analysis. Journal of cancer research and clinical oncology. 2011; 137(7):1053–60. [PubMed: 21499874]

[21] Dyrstad SW, Yan Y, Fowler AM, et al. Breast cancer risk associated with benign breast disease: systematic review and meta-analysis Breast Cancer Res Treat (2015) 149:569–575

[22] Rudland P, Gusterson B. John Sloane – Eminent pathologist who pioneered breast cancer treatment. https://www.theguardian.com/news/2000/may/26/guardianobituaries2 The Guardian, May 2000 (accessed 28 February 2020)

[23] Harnett A, Smallwood J, Titshall V, et al. on behalf of the Guideline Development Group. Diagnosis and treatment of early breast cancer, including locally advanced disease—summary of NICE guidance. BMJ 2009; 338:b438 doi: https://doi.org/10.1136/bmj.b438

[24] Association of Breast Surgery: NHSBSP ABS Audit of Screen Detected Breast Cancer https://associationofbreastsurgery.org.uk/professionals/audit/nhs-breast-screening-programme-audit/ (accessed 17 September 2021)



For peer review only - http://bmjopen.bmj.com/site/about/guidelines.xhtml

[25] NHS, England and Wales. The Health Service (Control of Patient Information) Regulations 2002. http://www.legislation.gov.uk/uksi/2002/1438/contents/made (accessed 30 September 2021)

[26] Independent Cancer Patients' Voice http://www.independentcancerpatientsvoice.org.uk/ (accessed 17 September 2021)

[27] Government Digital Service. www.gov.uk/phe/sloane-project (accessed 30 September 2021)

[28] Government Digital Service. https://www.gov.uk/government/collections/breast-screening-professional-guidance September 2020 (accessed 30 September 2021)

[29] Government Digital Service. https://www.gov.uk/government/publications/breast-screening-programme-specific-operating-model August 2021 (accessed 20 September 2021)

[30] The National Archives https://webarchive.nationalarchives.gov.uk/20180903111938/http://www.sloaneproject.co.uk/Pathology.htm September 2018 (accessed 20 September 2021)

[31] The Royal College of Pathologists. Pathology reporting of breast disease in surgical excision specimens incorporating the dataset for histological reporting of breast cancer. www.rcpath.org June 2016

[32] The National Archives https://webarchive.nationalarchives.gov.uk/ukgwa/20180903111940/http://www.sloaneproject.co.uk/Radiology.htm September 2018 (accessed 30 September 2021)

[33] Gale AG. PERFORMS: a self-assessment scheme for radiologists in breast screening. Semin Breast Dis 2003 Sep 30;6(3):148e52.WB Saunders.

[34] Office for National Statistics: User guide to mortality statistics https://www.ons.gov.uk/peoplepopulationandcommunity/birthsdeathsandmarriages/deaths/methodologies/userguidetomortalitystatisticsjuly2017 Latest revision July 2021 (accessed 08 October 2021)



For peer review only - http://bmjopen.bmj.com/site/about/guidelines.xhtml

[35] National Records of Scotland: Death Certificates and Coding the Cause of Death https://www.nrscotland.gov.uk/statistics-and-data/statistics/statistics-by-theme/vital-events/deaths/deaths-background-information/death-certificates-and-coding-the-causes-of-death August 2017 (accessed 08 October 2021)

[36] National Disease Registration Service https://www.ndrs.nhs.uk/resources/a-guide-to-cancer-data-and-working-with-us/ (accessed 08 October 2021)

[37] NHS Digital: About the HES database https://digital.nhs.uk/data-and-information/data-tools-and-services/data-services/hospital-episode-statistics (accessed 22 January 2021)

[38] NHS Digital Cancer Waiting Times data collection https://digital.nhs.uk/data-and-information/data-collections-and-data-sets/data-collections/cancerwaitingtimescwt (accessed 08 October 2021)

[39] NHS Digital DAPB0111: Radiotherapy Data Set https://digital.nhs.uk/data-and-information/information-standards/information-standards-and-data-collections-including-extractions/publications-and-notifications/standards-and-collections/dapb0111-radiotherapy-data-set (accessed 08 October 2021)

[40] SACT Systemic Anti-Cancer Therapy Dataset http://www.chemodataset.nhs.uk/home (accessed 22 January 2021)

[41] Public Health Scotland: Scottish Cancer Registry https://www.isdscotland.org/Health-Topics/Cancer/Scottish-Cancer-Registry/ (accessed 22 January 2021)

[42] NHS Wales Informatics Service: Information Services https://nwis.nhs.wales/information-services/ (accessed 22 January 2021)

[43] World Health Organisation: International Statistical Classification of Disease and Related Health Problems (ICD) https://www.who.int/classifications/classification-of-diseases (accessed 22 January 2021)



For peer review only - http://bmjopen.bmj.com/site/about/guidelines.xhtml

[44] NHS Digital: NHS Data Model and Dictionary https://datadictionary.nhs.uk/supporting_information/opcs_classification_of_interventions_and_procedures.html?hl=opcs (accessed 22 January 2021)

[45] Evans A, Clements K, Maxwell A, et al. Mammographic bi-dimensional product: a powerful predictor of successful excision of ductal carcinoma in situ. Clinical Radiology 2007; 62: 787-791

[46] Dodwell D, Clements K, Lawrence G, et al. Radiotherapy following breast-conserving surgery for screen-detected ductal carcinoma in situ (DCIS): Indications and utilisation in the UK: Findings from the Sloane Project. British Journal of Cancer 2007; 97: 725-729

[47] Thomas J, Hanby A, Pinder S, et al. Implications of Inconsistent Measurement of ER Status in Non-Invasive Breast Cancer: A Study of 1,684 Cases from the Sloane Project The Breast Journal Volume 14, Number 1, January/February 2008, pp. 33-38(6)

[48] Maxwell A J, Evans A J, Carpenter R, et al. Follow-up for screen-detected ductal carcinoma in situ: Results of a survey of UK centres participating in the Sloane Project. European Journal of Surgical Oncology (EJSO) 2009 35 (10) 1055-1059

[49] Thomas J, Evans A, Macartney J, et al on behalf of the Sloane Project Steering Group. Radiological and pathological size estimations of pure ductal carcinoma in situ of the breast, specimen handling and the influence on the success of breast conservation surgery: a review of 2564 cases from the Sloane Project. British Journal of Cancer (2010) 102, 285 – 293

[50] Evans A, Clements K, Maxwell A, et al on behalf of the Sloane Project Steering Group. Lesion size is a major determinant of the mammographic features of ductal carcinoma in situ: findings from the Sloane project. Clinical Radiology 2010 Volume 65, Issue 3, Pages 181-184

[51] Thomas J, Hanby A M, Pinder S E, et al. Adverse surgical outcomes in screen-detected ductal carcinoma in situ of the breast. European Journal of Cancer (2014) 50, 1880– 1890




For peer review only - http://bmjopen.bmj.com/site/about/guidelines.xhtml

[52] Nicholson S, Hanby A, Clements K, et al, on behalf of the Sloane Project Steering Group. Variations in the management of the axilla in screen-detected Ductal Carcinoma In Situ: Evidence from the UK NHS Breast Screening Programme audit of screen detected DCIS. European Journal of Surgical Oncology 2015; Jan;41(1):86-93. doi: 10.1016/j.ejso.2014.09.003.

[53] Clements K, Dodwell D, Lawrence G, et al, on behalf of the Sloane Project Steering Group. Radiotherapy after mastectomy for screen-detected ductal carcinoma in situ. European Journal of Surgical Oncology (EJSO) Volume 41, Issue 10, October 2015, Pages 1406-1410 https://doi.org/10.1016/j.ejso.2015.07.021

[54] Maxwell AJ, Clements K, Dodwell DJ, et al, on behalf of the Sloane Project Steering Group. The radiological features, diagnosis and management of screen-detected lobular neoplasia of the breast: Findings from the Sloane Project. The Breast 27 (2016) 109-115

[55] Thompson AM, Clements K, Cheung S, et al. Management and 5-year outcomes in 9938 women with screen-detected ductal carcinoma in situ: the UK Sloane Project. European Journal of Cancer. 2018;101:210-9.

[56] Shaaban A, Hilton B, Clements K, et al, on behalf of the Sloane Project Steering Committee. Pathological features of 11337 patients with primary ductal carcinoma in situ (DCIS) and subsequent events – results from the UK Sloane Project. British Journal of Cancer. 2020 https://doi.org/10.1038/s41416-020-01152-5

[57] Mannu GS, Wang Z, Broggio J, et al. Invasive breast cancer and breast cancer mortality after ductal carcinoma in situ in women attending for breast screening in England, 1988-2014: population based observational cohort study. BMJ. 2020 May 27;369.

[58] Dodwell D, Jauhari Y, Gathani T, et al. Treatment variation in early breast cancer in the UK. BMJ 2020; 371:m4237 doi: https://doi.org/10.1136/bmj.b438

[59] Jenkinson D, Freeman K, Clements K, et al. Breast screening atypia and subsequent development of cancer: protocol for an observational analysis of the Sloane database in



For peer review only - http://bmjopen.bmj.com/site/about/guidelines.xhtml

England (Sloane atypia cohort study). BMJ Open 2022;12:e058050. doi:10.1136/bmjopen-2021-058050

[60] Megalios A, Shah V, Shami R, et al. Abstract P4-06-05: Heterogeneity of DCIS does not appear to be a biomarker of subsequent invasive cancer. American Association for Cancer Research 2020. Volume 80, Issue 4 Supplement, pp. P4-06-05

[61] https://www.dcisprecision.org/ (Accessed 20 September 2021)

[62] Lips EH, Kumar T, Megalios A, et al. Genomic profiling defines variable clonal relatedness between invasive breast cancer and primary ductal carcinoma in situ medRxiv 2021.03.22.21253209; doi: https://doi.org/10.1101/2021.03.22.21253209

[63] Wright JL, Rahbar H, Obeng-Gyasi S, et al. Overcoming barriers in ductal carcinoma in situ management: from overtreatment to optimal treatment. J Clin Oncol 2022;40(3):225–230.

For peer review only

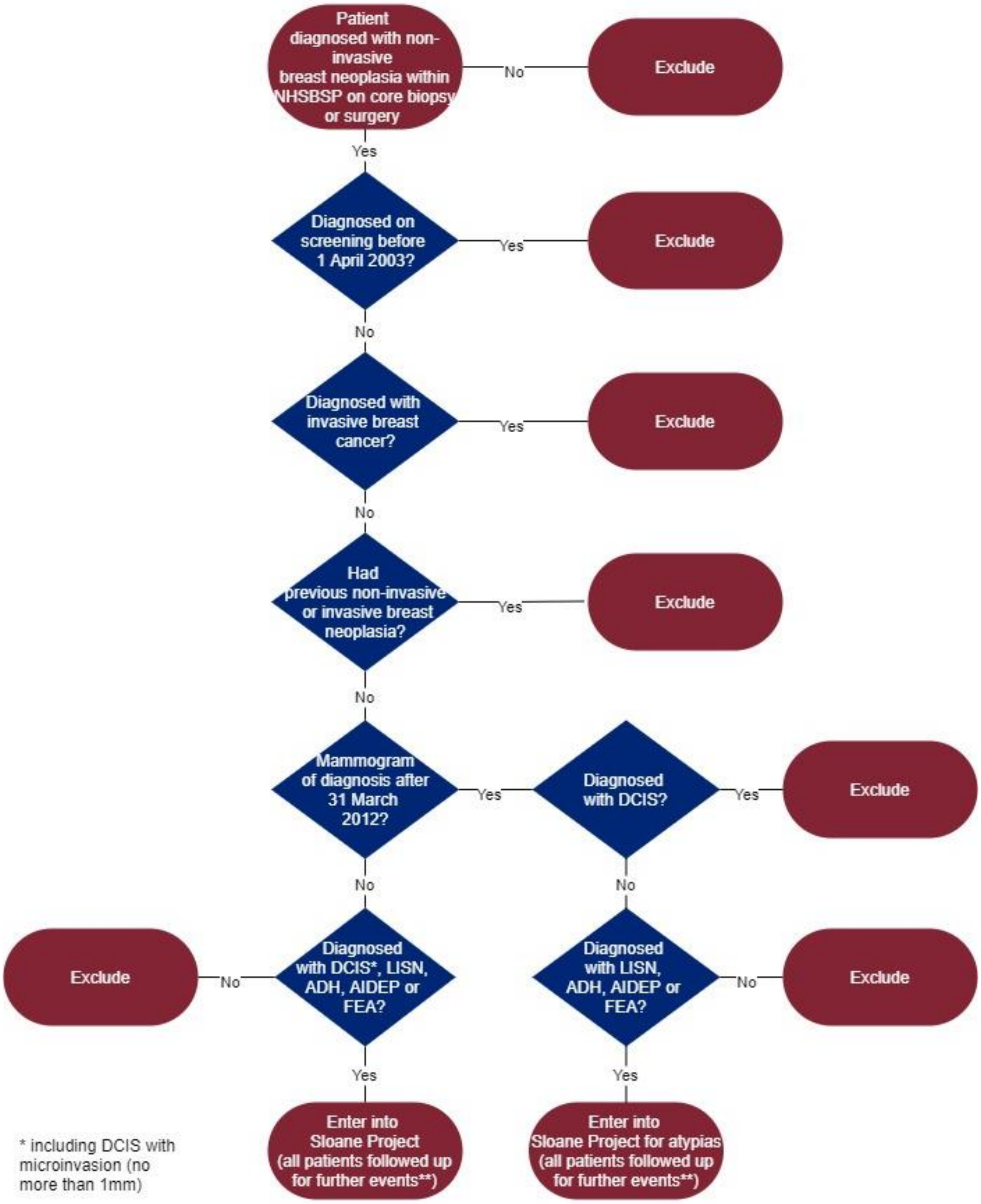


* including DCIS with microinvasion (no more than 1mm)

** further events include ipsilateral breast and nodal events, contralateral breast and nodal events and distant events, as well as deaths

| | |
|---|---|
| NHSBSP | National Health Service Breast Screening Programme |
| DCIS | ductal carcinoma in situ |
| LISN | lobular in situ neoplasia (including lobular carcinoma in situ and atypical lobular hyperplasia) |
| ADH | atypical ductal hyperplasia |
| AIDEP | atypical intraductal epithelial proliferation |
| FEA | flat epithelial atypia |

**Figure 1: Sloane eligibility flowchart**

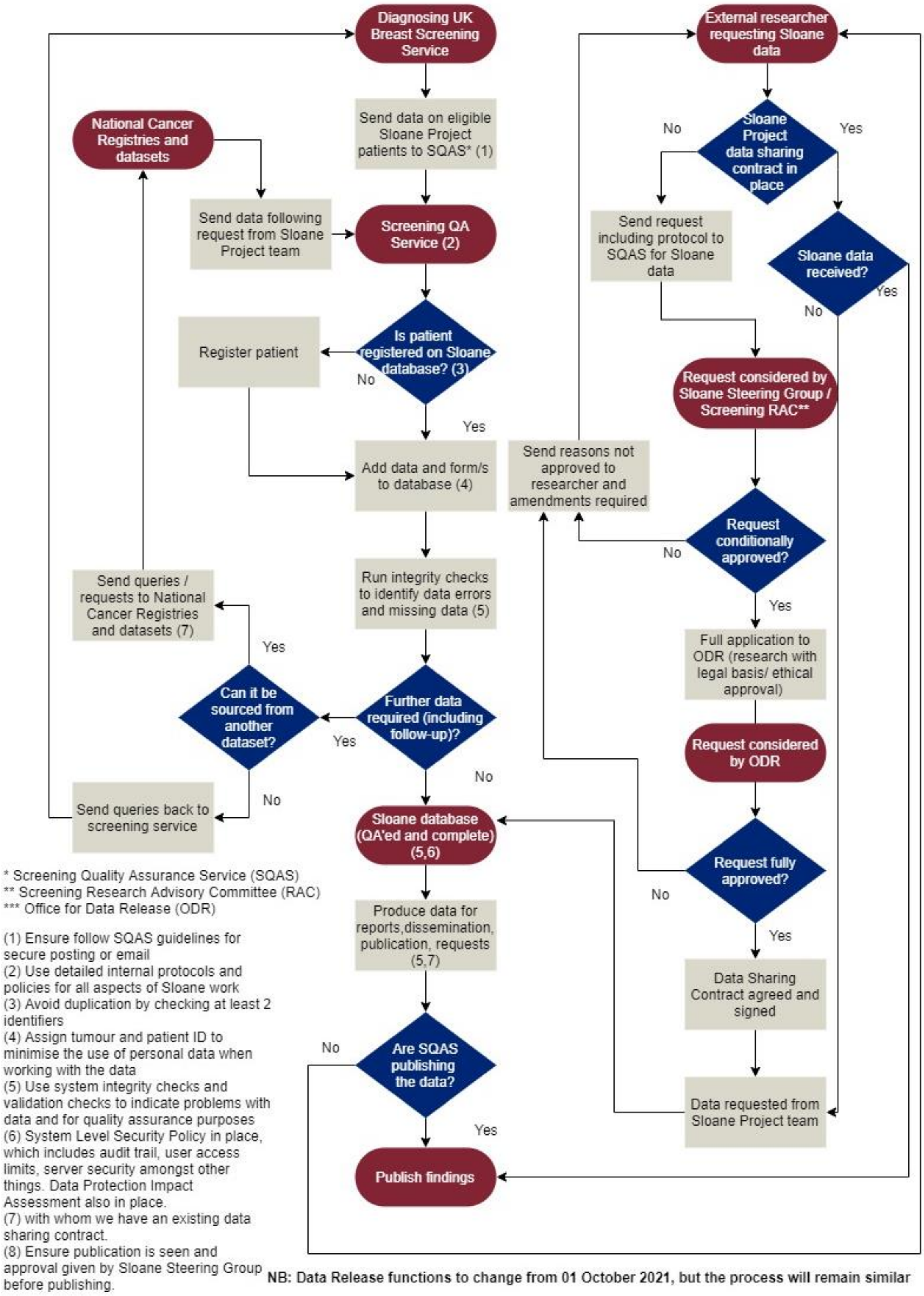


**Figure 2: Example data flow diagram and key for Sloane Project data**

# Sloane Project: Radiology Data Form

| Field | | Field | |
|---|---|---|---|
| **Surname** | | **Forename(s)** | |
| **Date of Birth** | | **Screening Unit** | |
| **Screening Number (Sx)** | | **Screening Round** | |
| **Date of Screening Mammogram** | | **NHS Number** | |

**Side:** **Left** ☐ **Right** ☐ ***For bilateral disease, please use separate form for each breast.***

**Site (Tick all that are involved)**

| | | | | | |
|---|---|---|---|---|---|
| Upper outer quadrant | ☐ | Upper inner quadrant | ☐ | | |
| Lower outer quadrant | ☐ | Lower inner quadrant | ☐ | Retroareolar | ☐ |

**Background Pattern (Tick ONE only)**

| | | | |
|---|---|---|---|
| Fatty | ☐ | Scattered fibroglandular density | ☐ |
| Hetrogeneously dense | ☐ | Extremely dense | ☐ |

**Predominant Radiological Feature (Tick ONE only)**

| | | | |
|---|---|---|---|
| Calcification | ☐ | Parenchymal Distortion | ☐ |
| Mass – Well Defined | ☐ | Spiculate Mass | ☐ |
| Mass – Ill Defined | ☐ | None of the above | ☐ |

**Does the lesion contain microcalcification?** **Yes** ☐ **No** ☐

**If "Yes", what is the most suspicious pattern? (Tick ONE only)**

| | | | | | |
|---|---|---|---|---|---|
| Cast/linear / Casting | ☐ | Granular/irregular / Crushed Stone | ☐ | Punctate / Powderish | ☐ |

**Sloane Project Number (For Official Use Only)** ☐

**QA Ref Number (For Official Use Only)** ☐

Version 2: Issued 01/08/2009

For peer review only

## Estimated Size of Lesion (in mm)

***Mammogram***

| | obl | cc |
|---|---|---|
| Distance from base of nipple to nearest part of the lesion | | |
| Maximum length of lesion | | |
| Maximum diameter of lesion at 90º to long axis of lesion | | |

## Data Required to Calculate Breast Volume (in mm)

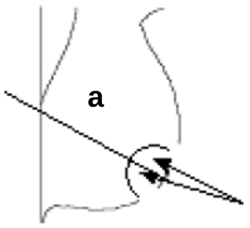


**a =**

Angles should be equal

**Chest wall to nipple (in the nipple plane)**

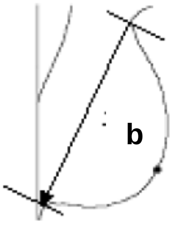


**b =**

**Infra mammary fold to supra mammary (axillary) fold**

***Magnetic Resonance Imaging (MRI)***

Was a pre-operative MRI performed? **Yes** ☐ **No** ☐

Estimated maximum dimension of lesion (mm) on MRI ☐

---

**Specimen X-Ray**

Was a specimen X-ray performed? **Yes** ☐ **No** ☐

**If "Yes":**

Was the specimen X-ray diagnostic or therapeutic? **Diagnostic** ☐ **Therapeutic** ☐

Is the lesion present in the specimen X-ray? **Yes** ☐ **No** ☐

Does the lesion appear radiologically to have been completely excised? **Yes** ☐ **No** ☐

Minimum Distance from Edge of Lesion to Edge of Specimen (in mm) ☐

---

**Date (DD/MM/YY)** ☐ **Radiologist (Print name)** ☐

For peer review only

# Sloane Project: Treatment Data Form

**Name** | **Screening No.**

**Date of Birth** | **Screening Unit**

**Hospital** | **Hospital No.**

**NHS No.**

**Side:** **Left** ☐ **Right** ☐ ***For bilateral disease, please use separate form for each breast.***

**Surgical Procedure(s)**
Total number of operations performed
(if more than 2 operations please use separate form for operation 3 onwards)

Was there a pre-operative diagnosis of DCIS? **Yes** ☐ **No** ☐

**Operation 1**
Date (dd/mm/yy)

Grade of surgeon performing operation

**Operation 2**
Date (dd/mm/yy)

Grade of surgeon performing operation

**Breast Procedures**
Please indicate procedures undertaken at Operation 1 & Operation 2.
(Tick one box only for each operation. If more than 2 operations please use separate form)

| | Op 1 | Op 2 |
|---|---|---|
| Diagnostic Biopsy | ☐ | ☐ |
| Wide Local Excision including excision of nipple | ☐ | ☐ |
| Wide Local Excision preserving nipple | ☐ | ☐ |
| Further excision to clear margins | ☐ | ☐ |

| | Op 1 | Op 2 |
|---|---|---|
| Simple mastectomy | ☐ | ☐ |
| Subcutaneous mastectomy | ☐ | ☐ |
| Skin sparing mastectomy | ☐ | ☐ |
| Therapeutic mammoplasty with nipple preservation | ☐ | ☐ |
| Therapeutic mammoplasty without nipple preservation | ☐ | ☐ |

**Immediate Breast Reconstruction**
(If more than 2 operations please use separate form)

| | Op 1 | Op 2 | ***Plus Implants (please tick)*** |
|---|---|---|---|
| Latissimus Dorsi flap | ☐ | ☐ | ☐ |
| D.I.E.P. flap | ☐ | ☐ | ☐ |
| T.R.A.M. flap | ☐ | ☐ | ☐ |

| | Op 1 | Op 2 |
|---|---|---|
| Wide Local Excision + mini latissimus dorsi flap (Rainsbury's procedure) | ☐ | ☐ |
| Implant only | ☐ | ☐ |
| Other autologous tissue technique | ☐ | ☐ |

**Sloane Project Number (For Official Use Only)**

**QA Ref Number (For Official Use Only)**

Version 2: Issued 01/08/2009

For peer review only - http://bmjopen.bmj.com/site/about/guidelines.xhtml

**Axillary Procedures**

| | Op 1 Yes | Op 1 No | Op 2 Yes | Op 2 No |
|---|---|---|---|---|
| Was a decision(s) made pre-operatively re axillary surgery at the MDM? | ☐ | ☐ | ☐ | ☐ |

Were axillary nodes taken? **Yes** ☐ **No** ☐

**If "Yes" which of the following procedures were carried out.**
(Tick all that apply. If more than 2 operations performed please use separate form.)

| | Op 1 | Op 2 |
|---|---|---|
| Sentinel node Procedure | ☐ | ☐ |
| Axillary node sample | ☐ | ☐ |
| Axillary node clearance | ☐ | ☐ |

**Further Surgical Details**
**For women treated with conservation surgery:**

| | Op 1 Yes | Op 1 No | Op 2 Yes | Op 2 No |
|---|---|---|---|---|
| Was the margin nearest to the nipple marked? | ☐ | ☐ | ☐ | ☐ |
| Was the specimen orientated? | ☐ | ☐ | ☐ | ☐ |

**For ALL operation types:**

| | Op 1 Yes | Op 1 No | Op 2 Yes | Op 2 No |
|---|---|---|---|---|
| Was a further excision performed at the same operation? | ☐ | ☐ | ☐ | ☐ |
| Was the immediate re-excision orientated? | ☐ | ☐ | ☐ | ☐ |
| Were cavity shavings (bed biopsies) taken? | ☐ | ☐ | ☐ | ☐ |
| Was a further excision carried out on the basis of the Specimen X-ray at the time? | ☐ | ☐ | ☐ | ☐ |

**Treatment Strategy – Following the Final Operation**

Was the status of the margins discussed at the MDM? **Yes** ☐ **No** ☐

Does the final margin status fulfil local protocol for adequacy of resection margins? **Yes** ☐ **No** ☐ **No Protocol** ☐

If "No" to above question, please state reasons why no further surgical treatment was undertaken if margins were inadequate. ☐

Did the post-op MDM decision on adjuvant therapy include (Tick all that apply):

Referral for Radiotherapy ☐ No further adjuvant therapy ☐ Other therapy ☐

Hormone therapy ☐ Date started Hormone Therapy ☐

Type of Hormone Therapy Tamoxifen ☐ Aromatase Inhibitor ☐ Other ☐

**Clinical Trials**
Patient entered Clinical Trial **Yes** ☐ **No** ☐ **Name of trial entered** ☐

**Date (DD/MM/YY)** ☐ **Surgeon (Print name)** ☐

For peer review only


Version 2: Issued 01/08/2009

# Sloane Project: Pathology Data Form

**Please complete ONE form for an EPISODE.**

***For bilateral disease, please use separate form for each breast.***

| | | | |
|---|---|---|---|
| **Surname** | | **Forename(s)** | |
| **Date of Birth** | | **Screening Unit** | |
| **Hospital** | | **Hospital No.** | |
| **Histology Report No.** | | **NHS No.** | |

**Side:** **Left** ☐ **Right** ☐

**Specimen Type (Tick ALL that apply, and weight, if known)**

| | Y/N | Disease present in core: DCIS | ADH | LCIS |
|---|---|---|---|---|
| Core biopsy (standard 14 gauge) | ☐ | ☐ | ☐ | ☐ |
| 8 or 11 gauge core (e.g. mammotomy) | ☐ | ☐ | ☐ | ☐ |

DCIS core grade: High ☐ Intermediate ☐ Low ☐

Calcification Present: None ☐ Benign ☐ Malignant ☐ Benign & Malignant ☐

| **Surgical Specimens:** | **Y/N** | **Weight (g)** | | **Y/N** | **Weight (g)** |
|---|---|---|---|---|---|
| Diagnostic Open Biopsy | ☐ | ☐ | Therapeutic Excision (WLE) | ☐ | ☐ |
| Bed Biopsies/ Cavity Shaves | ☐ | ☐ | Delayed re-excision (i.e. at a later operation) | ☐ | ☐ |
| Immediate re-excision (i.e. at time of WLE) | ☐ | ☐ | Completion Mastectomy | ☐ | ☐ |
| | | | Mastectomy | ☐ | ☐ |

| **For therapeutic excision / WLE:** | **Yes** | **No** | **Uninterpretable** |
|---|---|---|---|
| Nipple orientation marker present? | ☐ | ☐ | ☐ |
| Other orientation markers (sutures/clips)? | ☐ | ☐ | ☐ |

**Excision Histology – Lesion** (Tick all present): **DCIS** ☐ **ADH** ☐ **LISN** ☐

**For DCIS only**

Maximum (total) size of DCIS (mm) (summation from all specimens) ☐

Please tick if the total is size from one specimen only ☐

If summation from several specimens, please give size in each (mm) ☐ + ☐ + ☐

**Sloane Project Number (For Official Use Only)** ☐

**QA Ref Number (For Official Use Only)** ☐

Version 2: Issued 01/08/2009

For peer review only

**Highest Nuclear Grade (Please tick one only)** High ☐ Intermediate ☐ Low ☐

**Growth Pattern (Tick all that apply)**

Solid ☐ Cribriform ☐ Micropapillary ☐ Papillary ☐

Apocrine ☐ Flat/clinging ☐ Other type, please state……..…………………

| | **Yes** | **No** | | **Yes** | **No** | **N/A** |
|---|---|---|---|---|---|---|
| Comedo necrosis present? | ☐ | ☐ | Paget's disease? | ☐ | ☐ | ☐ |

| | **Yes** | **No** | | |
|---|---|---|---|---|
| Microinvasion (<1mm)? | ☐ | ☐ | No. foci microinvasion? | ☐ |

---

**Distance of DCIS to margins (mm)** **(Please complete all available and indicate if >10mm)**

| | Medial | Lateral | Superior | Inferior | Superficial | Deep | Nipple |
|---|---|---|---|---|---|---|---|
| **Therapeutic Excision** | ☐ mm | ☐ mm | ☐ mm | ☐ mm | ☐ mm | ☐ mm | ☐ mm |

***Please supply margin data above for a therapeutic excision, even if followed by mastectomy.***

| | | | | | | | |
|---|---|---|---|---|---|---|---|
| **If altered by further surgery, final margin distance** | ☐ mm | ☐ mm | ☐ mm | ☐ mm | ☐ mm | ☐ mm | ☐ mm |

**Mastectomy**
*(please tick if this applies)* ☐

Does another process extend to the margin? **ADH** ☐ **LISN** ☐
(Please tick all that apply)

---

**Immediate re-excision / Cavity Shaves / Bed Biopsies**

No. samples received ☐

| | **DCIS** | **ADH** | **LISN** |
|---|---|---|---|
| Did these samples contain (Tick all that apply) | ☐ | ☐ | ☐ |

---

| **Nodes** | **Axillary Nodes** | **Sentinel Node** | **Others** |
|---|---|---|---|
| Number examined overall | ☐ | ☐ | ☐ |
| Number positive overall | ☐ | ☐ | ☐ |

---

| **Receptor Status** | **Positive** | **Negative** | **Not Known** | **Cut off used** |
|---|---|---|---|---|
| ER Status | ☐ | ☐ | ☐ | ☐ |
| PGR Status | ☐ | ☐ | ☐ | ☐ |
| HER-2 Status | ☐ | ☐ | ☐ | ☐ |

---

**Date (DD/MM/YY)** ☐ **Pathologist (Print name)** ☐

For peer review only


Version 2: Issued 01/08/2009

# Sloane Project: Radiotherapy Data Form

**Surname** ☐ **Forename(s)** ☐

**Date of Birth** ☐ **Screening Unit** ☐

**Hospital** ☐ **Hospital No.** ☐

**NHS No.** ☐

**Side:** Left ☐ Right ☐ ***For bilateral disease, please use separate form for each breast.***

**Method of treatment**
**Radiotherapy - External Beam** **Yes** ☐ **No** ☐ ***If "No" why?*** ☐

***If "Yes" please complete the following:-***

Dose(Gy) ☐ Energy (MV) ☐ Number of fractions ☐

Treated daily? **Yes** ☐ **No** ☐ *If "**No**" please state frequency* ☐

Date treatment commenced (dd/mm/yy) ☐ Date treatment finished (dd/mm/yy) ☐

Axilla treated? **Yes** ☐ **No** ☐ Boost given? **Yes** ☐ **No** ☐

*If Boost given, please complete the following:-*

Electrons (Energy – MeV) ☐ Megavoltage (Energy – MV) ☐ Orthovoltage (Energy – kV) ☐ Dose (Gy) ☐ Number of fractions ☐

Treated daily? **Yes** ☐ **No** ☐

Date treatment commenced (dd/mm/yy) ☐ Date treatment finished (dd/mm/yy) ☐

**Brachytherapy – Interstitial**

**Yes** ☐ **No** ☐ *If "**Yes**" please complete the following:-* Dose(Gy) ☐

**Date (DD/MM/YY))** ☐ **Oncologist (Print name)** ☐

**Sloane Project Number (For Official Use Only)** ☐ **QA Ref Number (For Official Use Only)** ☐

For peer review only



Version 2: Issued 01/08/2009

# SLOANE PROJECT FOLLOW UP FORM
# (FOR RECURRENCES)

**The purpose of this detailed follow up form is to accumulate information on ipsilateral recurrences, contralateral breast disease and metastases. The form is split up into four sections:**

1) **Demographic details (these will already be completed if all information known)**
2) **Type of recurrence detected and route of presentation**

3) **Diagnosis, treatment and pathology of recurrence/contralateral disease**
   (a) **Ipsilateral recurrence**
   (b) **Contralateral breast disease**

4) **Diagnosis and treatment of distant metastases**

**Please complete all sections that apply to this patient.**

---

## SECTION 1 Demographic Details

**Screening Unit** ☐ **Date of Birth** ☐

**NHS Number** ☐ **Screening Number** ☐

**Hospital Number** ☐

---

## SECTION 2

### Diagnosis of recurrence / contralateral disease / metastases

**Type of Recurrence Detected and Date of Diagnosis** (please tick all that apply)

Local-regional ☐ Distant ☐ Contralateral disease ☐

Date ☐ Date ☐ Date ☐

**Route of Presentation** (tick all that apply)

Detected on FU mammogram ☐ Clinical Exam at routine FU ☐

Following GP referral to OPD clinic ☐ Other (please give details)

______________________________

---



For peer review only - http://bmjopen.bmj.com/site/about/guidelines.xhtml

## SECTION 3 (a) Local-regional recurrence

**The purpose of this section is to accumulate information on the IPSILATERAL RECURRENCE – when it occurred, where it occurred in the breast, how it presented and how it was treated.**

### Diagnosis of local-regional recurrence

| Site of local-regional recurrence(s) (Tick all that apply) | | Procedures used to confirm recurrence (Tick all that apply) | | | | | |
|---|---|---|---|---|---|---|---|
| | | Mammogram | FNA | Core Biopsy | Excision biopsy | MRI | Other (please specify) |
| Breast (if conserved) – at or adjacent to site of original primary | | | | | | | |
| Breast (if conserved) – second neoplasm some distance from site of original | | | | | | | |
| Nipple | | | | | | | |
| Mastectomy scar/flaps | | | | | | | |
| Ipsilateral axilla | | | | | | | |
| Ipsilateral supraclavicular fossa | | | | | | | |
| Reconstructed breast mound | | | | | | | |
| Other (Please specify) | | | | | | | |

For peer review only

### Treatment of local-regional recurrence

**Treatment of local-regional recurrence (Tick all that apply)**

Surgical procedures ☐ Radiotherapy ☐ Hormone Therapy ☐ Chemotherapy ☐

**Surgical procedures (tick all that apply)**

Further Wide Local Excision ☐ Mastectomy ☐ Axillary node surgery ☐ Other __________ (Please give details)

**Radiotherapy to recurrence (Please tick all sites that were treated)**

Breast ☐ Axilla ☐ Chest wall ☐ Supraclavicular fossa ☐ Interstitial ☐

**Hormone Therapy (please tick if given for recurrence)**

Tamoxifen ☐ Aromatase Inhibitor ☐ Other (Please state) __________

**Chemotherapy (please give details of the regime)**

CMF alone ☐ Herceptin ☐ Anthracycline containing regime (e.g. FEC, Epi-CMF) ☐

Taxane containing regime (e.g. Taxol, Taxotere) ☐ Other regime (Please give details) __________



For peer review only - http://bmjopen.bmj.com/site/about/guidelines.xhtml

## SECTION 3 (a) – continued
## Pathology of local-regional recurrence

**Type and Grade of recurrence (Please tick all that apply)**

Invasive ☐ Non-Invasive (DCIS) ☐ Non-invasive (LCIS/ALH) ☐

**Invasive grade** Grade 1 ☐ Grade 2 ☐ Grade 3 ☐

**DCIS Grade** Low ☐ Intermediate ☐ High ☐

**Size of local-regional recurrence**

DCIS (mm) ☐ Invasive size (mm) ☐ Whole tumour (DCIS + invasive) size (mm) ☐

**DCIS growth pattern(s) (Tick all that apply)**

Solid ☐ Cribriform ☐ Micropapillary ☐ Papillary ☐

Apocrine ☐ Flat ☐ Other (please specify) ☐

**Microinvasion** Present ☐ Not Present ☐

**Histological Type of Invasive Tumour**

No special type (ductal NST) ☐ Pure special type (90% purity, specify components present below) ☐ Mixed tumour type (50-90% special type component, specify components present below) ☐

**Component (s) present for pure special type and mixed tumour types:**

Tubular/cribriform ☐ Lobular ☐ Mucinous ☐ Medullary like ☐

Ductal / no special type ☐ Other (please specify) ☐

**Nodes**

Number examined overall ☐ Number positive ☐

**Vascular invasion** Present ☐ Possible ☐ Absent ☐ Not known ☐

**Receptor status**

| | Positive | Negative | Not known | Cut off for positivity used | Invasive | DCIS (indicate) |
|---|---|---|---|---|---|---|
| ER status | ☐ | ☐ | ☐ | ☐ | ☐ | ☐ |
| PgR status | ☐ | ☐ | ☐ | ☐ | ☐ | ☐ |
| HER-2 status | ☐ | ☐ | ☐ | ☐ | ☐ | ☐ |

Version 2: Issued 01/08/2009

For peer review only - http://bmjopen.bmj.com/site/about/guidelines.xhtml

## SECTION 3 (b) Contralateral disease

**We need to quantify the risk to the opposite breast, which is why we need to know whether the cancer is invasive or non-invasive and how it was treated.**

| Site of contralateral disease (Tick all that apply) | | Procedures used to confirm contralateral disease (Tick all that apply) | | | | | |
|---|---|---|---|---|---|---|---|
| | | Mammogram | FNA | Core Biopsy | Excision biopsy | MRI | Other (please specify) |
| Contralateral Breast | | | | | | | |
| Contralateral Nipple | | | | | | | |
| Contralateral axilla | | | | | | | |
| Other (Please specify) | | | | | | | |

### Treatment of contralateral disease

**Treatment of contralateral disease (Tick all that apply)**

Surgical procedures ☐ Radiotherapy ☐ Hormone Therapy ☐ Chemotherapy ☐

**Surgical procedures (tick all that apply)**

Wide Local Excision ☐ Mastectomy ☐ Axillary node surgery ☐ Other ____________ (Please give details)

**Radiotherapy for contralateral disease (Please tick all sites that were treated)**

Breast ☐ Axilla ☐ Chest wall ☐

Supraclavicular fossa ☐ Interstitial ☐

**Hormone Therapy (please tick if given after diagnosis of contralateral disease)**

Tamoxifen ☐ Aromatase Inhibitor ☐ Other (Please state) ____________

**Chemotherapy (please give details of the regime)**

CMF alone ☐ Herceptin ☐ Anthracycline containing regime (e.g. FEC, Epi-CMF) ☐

Taxane containing Regime (e.g. Taxol, Taxotere) ☐ Other regime (Please give details) ____________

**Other treatment given (please specify)** ____________

For peer review only



Version 2: Issued 01/08/2009

## SECTION 3 (b) – continued
## Pathology of contralateral disease

**Type and Grade of recurrence (Please tick all that apply)**

Invasive ☐ Non-Invasive (DCIS) ☐ Non-invasive (LCIS/ALH) ☐

**Invasive grade** Grade 1 ☐ Grade 2 ☐ Grade 3 ☐

**DCIS Grade** Low ☐ Intermediate ☐ High ☐

**Size of Contralateral recurrence**

DCIS (mm) ☐ Invasive size (mm) ☐ Whole tumour (DCIS + invasive) size (mm) ☐

**DCIS growth pattern(s) (Tick all that apply)**

Solid ☐ Cribriform ☐ Micropapillary ☐ Papillary ☐

Apocrine ☐ Flat ☐ Other (please specify) ☐

**Microinvasion** Present ☐ Not Present ☐

**Histological Type of Invasive Tumour**

No special type (ductal NST) ☐ Pure special type (90% purity, specify components present below) ☐ Mixed tumour type (50-90% special type component, specify components present below) ☐

**Component (s) present for pure special type and mixed tumour types:**

Tubular/cribriform ☐ Lobular ☐ Mucinous ☐ Medullary like ☐

Ductal / no special type ☐ Other (please specify) ☐

**Nodes**

Number examined overall ☐ Number positive ☐

**Vascular invasion** Present ☐ Possible ☐ Absent ☐

**Receptor status**

| | Positive | Negative | Not known | Cut off for positivity used | Invasive | DCIS (indicate) |
|---|---|---|---|---|---|---|
| ER status | ☐ | ☐ | ☐ | ☐ | ☐ | ☐ |
| PgR status | ☐ | ☐ | ☐ | ☐ | ☐ | ☐ |
| HER-2 status | ☐ | ☐ | ☐ | ☐ | ☐ | ☐ |

Version 2: Issued 01/08/2009

For peer review only - http://bmjopen.bmj.com/site/about/guidelines.xhtml

## SECTION 4 Distant metastases

**We would like information on whether or not the patient has confirmed distant metastases.**

**Site of distant metastases (Tick all that apply)**

Bone ☐ Lung ☐ Liver ☐ Brain ☐ Other (please specify) ____________________

**Treatment (Tick all that apply)**

**Surgical Procedures** ☐ If ticked, please indicate type of procedure ____________________

**Radiotherapy** ☐ If ticked, please give details of site ____________________

**Hormone Therapy** ☐ If ticked, please indicate type of hormone therapy ____________________

**Bisphosphonates** ☐

**Chemotherapy** ☐ If ticked, please give details of type of regime ____________________

**Other** ☐ If ticked, please give details ____________________

**Is there any evidence of any invasive focus to account for the metastases?**

Yes ☐ No ☐ N/K ☐ Please comment if you wish ____________________

**Thank you for your co-operation.**

For peer review only



For peer review only - http://bmjopen.bmj.com/site/about/guidelines.xhtml

The Sloane Project
Understanding non-invasive breast disease

# Sloane Project atypia audit form

**For Official Use Only**

**Sloane ID**

**Patient ID**

- Complete form for each Sloane Project patient
- Submit data on patients diagnosed with lobular neoplasia (LN), lobular carcinoma in situ (LCIS), pleomorphic lobular carcinoma in situ (PLCIS), atypical intraductal epithelial proliferation (AIDEP), atypical ductal hyperplasia (ADH), atypical lobular hyperplasia (ALH), flat epithelial atypia (FEA)
- Submit data from patients who screen-detected by the NHS Breast Screening Programme on or after 1 April 2012.
- Concurrent or previous Ductal Carcinoma In Situ (DCIS) or Invasive Breast Cancer are not eligible.
- Please return all forms securely using only nhs.net mail to **PHE.Sloaneproject@nhs.net** or to **Sloane Project team, Screening QA Service, NHS England & Improvement, 2nd Floor, 23 Stephenson Street, Birmingham B2 4BH**
- If you need help completing any part of this form, contact the team on PHE.Sloaneproject@nhs.net

| | | | |
|---|---|---|---|
| **Patient date of birth** | | **Patient NHS number** | |
| **Screening unit** | | **Screening number (SX)** | |
| **Date of screening mammogram** | | **Screening round** | |
| **Histology report number** | | **Hospital and hospital number** | |

**Side:** left ☐ right ☐ for bilateral disease, please use separate form for each breast

**Has patient had previous breast atypia? Y/N** ☐ **If yes, year of diagnosis** ☐

**Background pattern (Tick ONE only)**

Fatty ☐ Scattered fibroglandular density ☐ Hetrogeneously dense ☐ Extremely dense ☐

**Predominant radiological feature (Tick ONE only)**

Calcification ☐ Parenchymal distortion ☐ Mass: well-defined ☐ Spiculate mass ☐ Mass: ill-defined ☐ None of these ☐

**Does the lesion contain microcalcification?** Y/N ☐

**If yes, what is the most suspicious pattern? (Tick ONE only)**

Cast/linear/casting ☐ Granular/irregular/crushed stone ☐ Punctate/powderish ☐

**Maximum estimated size of lesion (in millimetres) on mammogram** ☐

| **Core biopsy** (tick all that apply) | Y/N | No. of cores | Gauge | AIDEP | LN | PLCIS | FEA | Benign |
|---|---|---|---|---|---|---|---|---|
| Standard core biopsy (14 gauge) | ☐ | ☐ | | ☐ | ☐ | ☐ | ☐ | |
| Diagnostic vacuum assisted biopsy (for example 8 or 11 gauge core) | ☐ | ☐ | ☐ | ☐ | ☐ | ☐ | ☐ | ☐ |

| | Y/N | No. of cores | Gauge | ADH | ALH | LCIS | PLCIS | FEA | Benign |
|---|---|---|---|---|---|---|---|---|---|
| Therapeutic vacuum assisted excision (for example 8 or 11 gauge core) | ☐ | ☐ | ☐ | ☐ | ☐ | ☐ | ☐ | ☐ | ☐ |

**Is calcification present in core?** Y/N ☐ If yes, indicate site (tick ONE only) Atypical lesion ☐ Benign lesion ☐ Atypia and benign ☐ Unknown site ☐



For peer review only - http://bmjopen.bmj.com/site/about/guidelines.xhtml

**Other benign lesion present?** Y/N ☐ If yes, specify which (tick ONE only)

Fibroadenoma ☐ Papilloma ☐ Radial scar ☐ Other ☐ If other, please specify: ☐

| **Surgical Specimens** | Y/N | DATE | | Y/N | DATE |
|---|---|---|---|---|---|
| Diagnostic open biopsy | ☐ | ☐ | Delayed re-excision (for example at a later operation) | ☐ | ☐ |
| Therapeutic excision (WLE) | ☐ | ☐ | Completion mastectomy | ☐ | ☐ |
| Bed biopsies/ cavity shaves | ☐ | ☐ | Mastectomy | ☐ | ☐ |
| Immediate re-excision (for example at time of WLE) | ☐ | ☐ | No surgical specimen | ☐ | |

**Disease present in surgical specimen (tick all that apply)**

| ADH | ALH | LCIS | PLCIS | FEA | No additional residual disease | Maximum (total) size of lesion |
|---|---|---|---|---|---|---|
| ☐ | ☐ | ☐ | ☐ | ☐ | ☐ | ☐ |

**Does any disease extend to the margin of the surgical specimen or re-excision specimen?(tick all that apply)**

ADH ☐ ALH ☐ LCIS ☐ PLCIS ☐ FEA ☐

**Is calcification present in surgical specimen?** Y/N ☐ If yes, indicate site (tick ONE only) Atypical lesion ☐ Benign lesion ☐ Atypia and benign ☐ Unknown site ☐

**Is comedo necrosis present?** Y/N ☐

**Other benign lesion present?** Y/N ☐ If yes, specify which (tick ONE only)

Fibroadenoma ☐ Papilloma ☐ Radial scar ☐ Other ☐ If other, please specify: ☐

| **Nodes** | **Axillary nodes** | **Sentinel nodes** | **Others** |
|---|---|---|---|
| Number examined overall | ☐ | ☐ | ☐ |
| Number positive overall | ☐ | ☐ | ☐ |

**At which operation were nodes taken?** (e.g. 1st, 2nd) ☐

**Treatment strategy – following the final operation**
Did the post-op MDM decision on adjuvant therapy include (tick all that apply):

Referral for radiotherapy ☐ Date started radiotherapy ☐

No adjuvant therapy ☐ Other therapy ☐ Endocrine therapy ☐

Date started endocrine therapy ☐ Endocrine therapy type (tick all that apply) Tamoxifen ☐ Aromatase inhibitor ☐ Other ☐

**Date (DD/MM/YY)** ☐ **Print name and email address** ☐


For peer review only - http://bmjopen.bmj.com/site/about/guidelines.xhtml

# SLOANE PROJECT ~

## GUIDANCE FOR COMPLETION OF RADIOLOGY DATA FORM

### Demographic Details

The demographic details include patient identification, date of screening mammogram and whether this is prevalent or incident screening.

### Side

A separate form is required for each side in the cases of bilateral disease. For multi-focal disease in a single site, only one form should be completed.

### Site

For multi-focal disease, it is important that there is concordance of the site which is reported on the pathology form with that which is measured on the radiology form. As these cases are being identified at the postoperative MDM, correlation should take place at this time.

### Background Pattern

During the pilot phase of the radiology data collection a number of classifications were used to describe the background parenchymal pattern. Recognising that this is a highly subjective classification, the pilot centres agreed that the birads classification was the most reproducible.

### Predominant Radiological Feature

For ease of analysis, please choose the predominant radiological feature on the mammogram and classify as appropriate.

### Calcification

With regard to the classification of calcification, two systems of classification have been included ~ please choose one system only and examples of the three groups are enclosed.

### Estimated Size of Lesion on Mammograms

All measurements have to be done both on the oblique view and the CC views. Three reproducible measurements have been chosen:

a. Distance from base of nipple to nearest part of the lesion i.e. border of mass/first dot of calcium, closest area of parenchymal distortion (see accompanying diagram)
b. Maximum length of lesion ~ see diagram
c. Maximum diameter of lesion at $90^0$ to long axis of lesion ~ see diagram

Please note that as cases of distortion are few and far between and because of difficulties in relation to measuring these cases, it has been agreed that measurements of distortions are no longer required. This includes measuring distance from nipple.

For peer review only

In cases where there are two distinct areas separated by apparently normal breast tissue, then the measurement should be from one end of one site to the further most end of the other site, along with all the apparently normal breast tissue in between. The reason for this is that it reflects how we clinically manage such cases (i.e. if DCIS is found in two sites with apparently normal breast tissue in between the patient will generally be subjected to mastectomy).

**Data required to Calculate Breast Volume**

The accompanying formula (1/3 pi $r^2h$) has been validated and is thought to be the closest approximation to breast volume that can be carried out from 2 view mammography. Please be aware that on the original form the labels for the measurements were “h” and “2r”. The labels have now been amended to “a” and “b” respectively (though the same two measurements are still required) as it was felt that the previous labels were causing some confusion.

Please note that even though the size of the lesion and distance from the nipple are not to be measured in cases of distortion, the data to calculate breast volume is still required.

**Specimen X-Ray**

Please identify by tick whether the specimen is as a result of a diagnostic or therapeutic biopsy.

With the increase in the use of mammotomy to diagnose these cases pre-operatively, there is the possibility that in some cases all the calcifications will have been removed and that a clip will be left in situ. For the purposes of completion of this section, it is possible to say that the entire lesion has been removed, but it is inappropriate to make measurements as to proximity to the margin etc.

**Excision**

With regard to completeness of excision, arbitrarily 2mm (unmagnified) of “normal” breast tissue has to be present between the margins which are closest to the mammographic abnormality.

For peer review only

# SLOANE PROJECT
# Guidelines for Pathological Assessment of DCIS
# Pathology Protocol

## Introduction

It is presumed that the vast majority of cases of DCIS entered into the DCIS trial will be identified as screen-detected microcalcification and in general the existing NHSBSP Pathology Reporting Guidelines (NHSBSP Publications. Sheffield. 1995), or updated guidelines, should be adhered to. Reporting pathologists should take part in the National Breast Screening Pathology EQA scheme. Unless pre-operative diagnosis of DCIS has been successful, these may therefore be a diagnostic needle localisation biopsy, prior to a therapeutic procedure. Both the surgical and histopathological technique for handling of these specimens differs significantly. Some general guidelines for specimen handling, both in the operating theatre and in the laboratory can be described. For example, frozen section examination is inappropriate in cases of screen-detected microcalcification. Whatever technique is used, the methodology should enable production of the minimum dataset information.

## Surgical Handling

- It is anticipated that lesions will be resected according to the defined protocol. If their surgical resection differs from the protocol, for example dissection does not extend to the deep fascia or skin, this should be clearly indicated on the request form.
- The surgeon should orientate all specimens. Each unit should establish a code of orientation using either different lengths of suture or metal staples/clips or ink, which are anatomically relevant and assist in accurate evaluation of the specimen. The nipple extension / direction of the nipple should be separately marked.
- If more than one piece of tissue is removed, it should be made clear how the samples are orientated with respect to each other in order to simplify assessment of size of the lesion and distance to margins.
- After surgical excision the specimen should be X-rayed. The X-ray should be reported by the breast radiologist. However the X-rays must be available to the pathologist so that they can determine a) whether the microcalcification has been excised and b) its site and extent within the sample.
- The specimen should be sent immediately to the pathology laboratory – ideally in the fresh state. If this is not possible it should be immediately placed in an appropriate quantity of fixative.

For peer review only

### General Laboratory Handling

- Once received in the laboratory the entire surface of the specimen should be inked so that the margins of excision can be easily determined. This can be performed by prior removal of surface lipid by dipping the specimen in alcohol and drying and then applying an appropriate pigment such as India Ink, Alcian blue, dyed gelatine or a multiple ink technique.
- Good fixation is vital to preserve the morphological detail. This is particularly relevant for the diagnosis of these sometimes difficult intraductal epithelial proliferations. They must be placed in sufficient formalin or other appropriate fixative either before, or preferably after, receipt by the laboratory.

### Diagnostic Biopsies

- The specimen should be weighed and measured and then serially sliced at intervals of approximately 3mm.
- Cases where block selection is required (i.e. not embedded in their entirety) will benefit from specimen slice examination, particularly those where microcalcification is the principle feature by which the DCIS was detected. It enables blocks to be taken from the areas corresponding to the mammographic abnormality, as well as any other suspicious areas identified.
- The sites of sampling can be marked on the specimen X-ray or X-ray of slices, by using a white wax (chinograph) pencil or other marker.
- The sampling technique and the number of blocks taken are clearly dependent on the size of the specimen and the size of the abnormality. If the specimen is small, it is often best to block and examine all of the tissue. Samples approximately 30 mm or less in maximum dimension should be completely sliced, embedded and examined histologically.
- For larger specimens sampling should be adequate to determine accurately the size of the DCIS and exclude, within the boundaries of practicality, associated invasion. Sampling should include the extremes of the mammographic abnormality and adjacent tissue, in order to avoid underestimation of size of DCIS. This is particularly important as it is recognised that mammographic size may be an underestimate of true tumour size.

For peer review only

**Diagnostic Biopsies (continued)**

- The margins of diagnostic specimens should also be identified. The nearest margin to the mammographic abnormality should be included in sections. It should be noted, however, that the aim of these procedures is not complete excision and the histopathological assessment of margin status is not the primary requisite of the examination.
- Accuracy of size measurement of DCIS is vital and measurements using the Vernier scale of the stage micrometer or an eyepiece graticule are the ideal ways to ascertain this measurement. If the DCIS extends over several blocks the measurement may be partly deduced by summating the thickness of the blocks (if they are parallel samples) or from measurements deduced by mapping the involvement on the specimen.
- Large blocks and sections may be extremely helpful, especially for determining the size of DCIS, and are particularly helpful when the DCIS area is large. Adequate fixation may, however, on occasions be more difficult to achieve and large sections are also more difficult to cut and to store and the longer processing necessary may introduce delays. Their use depends on local preference.
- If diagnostic samples are sent as more than one portion, it can be extremely difficult to measure the absolute extent of DCIS present. In these cases it is appropriate to take a pragmatic approach and measure the maximum distance in any piece of tissue and to add the dimensions to give an approximate total size. If, however, the orientation of the specimens can be determined, the size of DCIS can be ascertained more reliably.

**Therapeutic Excisions**

- The specimen should be weighed and measured in three dimensions.
- The technique for sampling the abnormality will vary somewhat according to type of sample, specimen size and also with pathologist/laboratory preference. Several options are available. Whichever is utilised, as an absolute minimum, the information for the minimum dataset, including accurate measurement of size and detailed examination of the margin status and distance to margins must be provided. Three suggestions for handling these samples are described below. It should be noted that these are schematic diagrams and that frequently the specimen is more irregular in outline than demonstrated.

The pathology data record should indicate which method has been used. If a local examination policy is used which differs from these methods this must be indicated and the protocol provided to the co-ordination centre.

For peer review only

**METHOD 1 – Serial slicing perpendicular to the medial – lateral plane. Figure 1.**

**Figure 1**

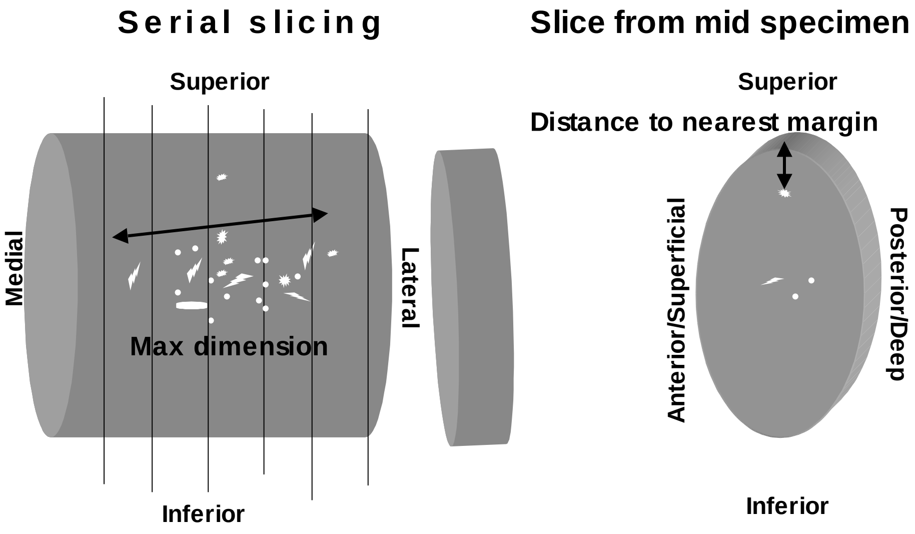


- The majority of these samples taken for mammographic calcification will require serial slicing at intervals of approximately 3mm.
- These specimens may benefit from specimen slice examination, but this may not be absolutely essential for all samples e.g. mass lesions. Where microcalcification is the principle feature by which the DCIS was detected, serially slicing and re-X-raying of the specimen slices will enable blocks to be taken most accurately from the areas corresponding to the mammographic abnormality, as well as any other suspicious areas identified. The sites of sampling can be marked on the specimen for radiological-pathological discussion in difficult cases.
- The number of blocks taken will depend on the size of the specimen and of the size of the abnormality. If the specimen is small, it is often best to block and examine all of the tissue. Samples 30mm or less in maximum dimension should be completely sliced, embedded and examined histologically.
- For larger specimens adequate sampling should be undertaken, to determine accurately the size of the DCIS and to exclude coexisting invasion. Sampling should include the extremes of the mammographic abnormality and adjacent tissue, in order to avoid underestimation of size of DCIS. This is particularly important as it is recognised that mammographic size may be an underestimate of true tumour size.

- If therapeutic samples are sent in more than one portion, it can be extremely difficult to measure the absolute largest extent of DCIS present. In these cases it is appropriate to take a pragmatic approach and measure the maximum distance in any piece of tissue and to add the dimensions to give an approximate total size. If, however, the orientation of the specimens can be determined, the size of DCIS can be ascertained more reliably.
- The margins of therapeutic excision specimens should also be extensively sampled. The nearest margin to the mammographic abnormality must be blocked, as an absolute minimum, in order to facilitate measurement of this distance. Accuracy is vital and measurement using the Vernier scale of the stage micrometer or an eyepiece graticule is the ideal way to ascertain this measurement. In larger lesions dot marking and mapping the lesion extent on the slide coverslip may be helpful.
- If the DCIS extends over several blocks the measurement of distance to margins may be partly deduced by summating the thickness of the parallel slices and from measurements deduced by mapping the involvement on the specimen.
- Large blocks and sections may be extremely helpful, especially for determining the size and the margin status of DCIS, particularly when the DCIS area is large. Adequate fixation may, however, on occasions be more difficult to achieve and large sections are also more difficult to cut and to store and the longer processing necessary may introduce delays. Their use depends on local preference.
- The use of different colour inks/markers on an individual section allows microscopic identification of specific margins.

For peer review only

**METHOD 2 - Serial slicing perpendicular to the superficial – deep plane. Figure 2.**

**Figure 2**

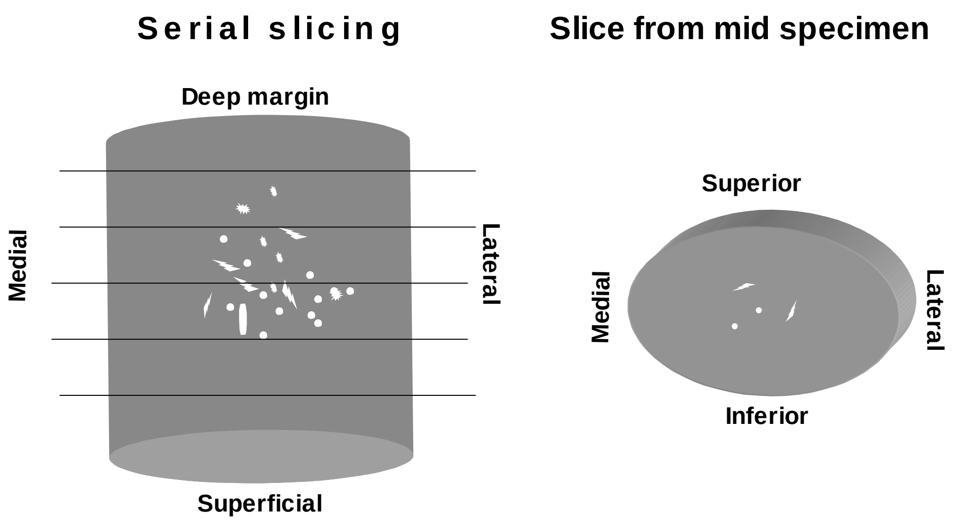


This is a variation of method 1, which is particularly suitable for smaller specimens, making use of large block techniques. The entire specimen can be examined as a small number of serial large sections and the technique is similar to the method used to examine radical prostatectomy specimens in many centres.

***METHOD 3 - Radial block and shave margin examination. Figure 3.***

**Figure 3**

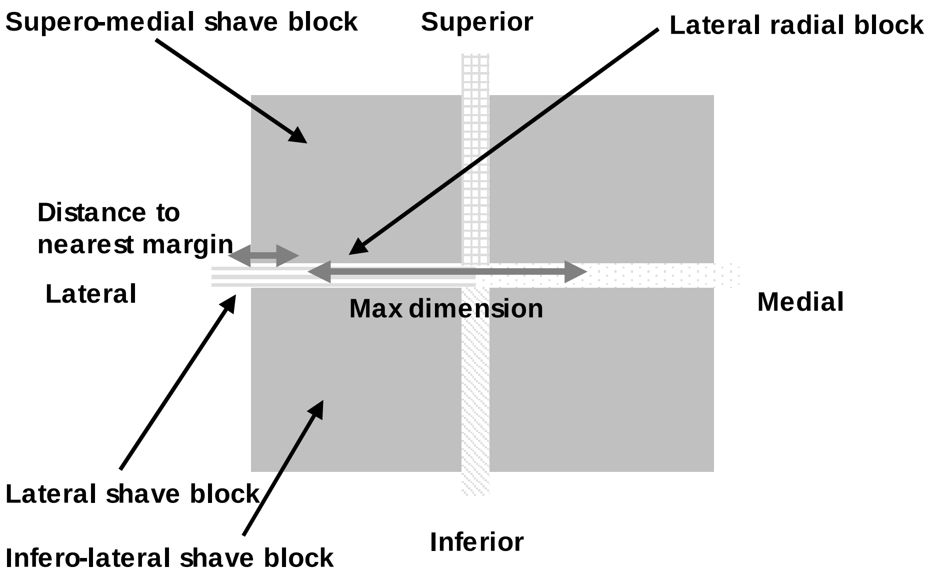


On occasions, particularly if a macroscopic abnormality is identified, it may be appropriate to sample the specimen with multiple blocks of the lesion, taken at right angles, as described below.

**Margins**

- Sections taken for measurement of distance to margins will include a slice through the lesion to the radial edges of the specimen and allow measurement of the distance using the stage micrometer.
- Blocks of the "*radial*" margins, lateral, medial, superior and inferior to the lesion should be sampled before taking "*shaves*" of the remaining circumferential aspect.
- If appropriate (i.e. nearest on the specimen) one or more of the following: supero-lateral, supero-medial, infero-medial, infero-lateral may be taken.
- It may be possible to take radial margin and the lesion in one section for smaller resections. For larger specimens, blocks may be of sufficient size to require blocking in 2 (or more) cassettes.
- The circumferential edge of the sample should be shaved, unless the surgeon provides cavity shaves. This may produce, for example: superior shave, supero-lateral shave, lateral shave, infero-lateral shave, inferior shave, infero-medial shave, medial shave and supero-medial shaved edges depending on the size of the specimen.

For peer review only - http://bmjopen.bmj.com/site/about/guidelines.xhtml

**Margins (continued)**

- It should be noted that shaved edges of the margins of the specimen of examination of “cavity shaves/bed biopsies” confirm completeness of excision but do not allow measurement of distance.
- **Cavity shave / biopsy specimens.** Centres may wish to use separately submitted surgical cavity shaving / biopsies as an adjunct to pathological examination of the margins of the main specimen. These specimens should be clearly marked and examined separately.

**Sampling the lesion**

- Blocks of the mammographic abnormality/lesion should be taken at right angles and allow as accurate as possible a determination of maximum extent of the DCIS.
- Additional blocks of the lesion should be sampled. In the majority of cases of DCIS treated by local surgical excision (i.e. not large lesions) it is appropriate to block the majority of the lesion/ area of calcification on specimen.
- In particular, the maximum extent of the calcification and the adjacent tissue should be taken to be certain that non-calcified DCIS does not extend beyond the radiological abnormality.
- If therapeutic samples are sent in more than one fragment, it can be extremely difficult to measure the absolute largest extent of DCIS present. In these cases it is appropriate to take a pragmatic approach and measure the maximum distance in any piece of tissue and to add the dimensions to give an approximate total size. If, however, the orientation of the specimens can be determined, the size of DCIS can be ascertained more reliably.

**Re-excision Specimens**

- If the radiological abnormality extends close to a margin on the specimen radiograph, the surgeon may undertake an immediate re-excision of that particular margin.
- A separate re-excision specimen may therefore be taken (a) at the time of initial surgery or (b) subsequent to the discovery of incomplete excision in a therapeutic marker or (c) following diagnostic localisation biopsy.
- The aim is to remove all of the previous biopsy site and its margins or one or more specific margins, which are known (or suspected to be) involved in the disease process. Whenever re-excision has been performed, the surgeon should orientate the re-excision specimen. It is therefore possible to measure the distance of any additional DCIS present to the new margin of excision or to calculate the distance of the DCIS to the new margin of excision if no DCIS is present.

For peer review only

## Re-excision Specimens (continued)

- If re-excision specimens are taken (or if diagnostic samples are sent in more than one fragment), it can be extremely difficult to measure the absolute largest extent of DCIS present. In these cases it is appropriate to take a pragmatic approach and measure the maximum distance in any piece of tissue and to add the dimensions to give an approximate total size. If, however, the orientation of the specimens can be determined, the size of DCIS can be ascertained more reliably.

## Histological Examination

2-4µm thick haematoxylin and eosin stained sections should be examined.

***Measurement of lesion size***

The size of DCIS and distance to the nearest margin of excision should be assessed accurately using a recognised method. Such methods include use of the microscope stage Vernier scale, a calibrated eyepiece graticule or lesion mapping technique involving dot marking of the lesion boundaries on the slide coverslip. This latter method is particularly suitable for lesions examined as large blocks and sections. If the DCIS lesion extends over several blocks the measurement of distance to margins may be partly deduced by summating the thickness of the parallel slices and from measurements deduced by mapping the involvement on the specimen. Knowledge of the size of the original mammographic lesion should be available and serves as a guide to assist both accurate measurement of lesion size and the assessment of excision status in the resection specimen.

***Grade of DCIS***

Grade should be ascertained according to the NHSBSP Guidelines.

A high power lens (40x) should be used to compare size of tumour cells with normal epithelial nuclear size and red blood cell size. Other features such as mitotic count, presence of prominent nucleoli and polarisation of nuclei may be helpful in assigning grade. In particular a high mitotic count is very rare in DCIS not of high histological grade.

For peer review only

***Grade of DCIS (continued)***

**High Nuclear Grade DCIS**

Cells have pleomorphic, irregularly spaced, and usually large nuclei exhibiting marked variation in size, irregular nuclear contours, coarse chromatin, and prominent nucleoli. Nuclei are often 3 or more times the size of erythrocytes. Mitoses are usually frequent and abnormal forms may be seen. If mitoses are prominent there is a high likelihood that a case is of high grade. High grade DCIS may exhibit several growth patterns. It is often solid with comedo type central necrosis, which frequently contains deposits of amorphous calcification. Sometimes a solid proliferation of malignant cells fills the duct without necrosis but this is relatively uncommon and may be confined to nipple/lactiferous ducts in cases presenting with Paget's disease of the nipple. High nuclear grade DCIS may also exhibit micropapillary and cribriform patterns frequently associated with central comedo type necrosis. Unlike low nuclear grade DCIS there is rarely any polarisation of cells covering the micropapillae or lining the intercellular spaces.

**Intermediate Nuclear Grade DCIS**

These types cannot be assigned readily to the high or low nuclear grade categories. The nuclei show moderate pleomorphism, less than that seen in the high grade disease, but lack the monotony of the small cell type. The nuclear to cytoplasmic ratio is often high and one or two nucleoli may be identified. The growth pattern may be solid, cribriform or micropapillary and the cells usually exhibit some degree of polarisation covering papillary processes or lining intercellular lumina.

**Low Nuclear Grade DCIS**

Low grade DCIS is composed of monomorphic evenly spaced cells with rounded, centrally placed nuclei and inconspicuous nucleoli. The nuclei are usually, but not invariably, small. Mitoses are few and there is rarely individual cell necrosis. These cells are generally arranged in micropapillary and cribriform patterns. Both patterns are frequently present within the same lesion although the cribriform pattern is more common and tends to predominate. There is usually polarisation of cells covering the micropapillae or lining the intercellular lumina. Less frequently low grade DCIS has a solid pattern.

When terminal duct lobular units are involved the process can be very difficult to distinguish from lobular carcinoma in situ (LCIS) and if both intraductal and intralobular patterns are seen it is appropriate to classify the process as DCIS **and** LCIS. Features in favour of DCIS are the slightly larger cell size, readily visible cell membranes, cytoplasmic basophilia, and variation in cellular arrangement and size, greater cellular cohesion and lack of intracytoplasmic lumina.

For peer review only

***Grade of DCIS (continued)***

**Mixed Types of DCIS**

A small proportion of cases of DCIS exhibit features of differing nuclear grade. Such variation in cell type is unusual but, if present, the case should be classified by the highest nuclear grade present.

***Architecture of DCIS***

The growth pattern should be recorded. All architectural patterns present should be noted including solid, cribriform, micropapillary, flat (clinging), and papillary. Comedo DCIS is considered to represent solid DCIS (usually of high nuclear grade) with central necrosis and is not therefore included as a specific growth pattern (see below – completing the Sloane Project Pathology Form).

**Rarer Subtypes of DCIS**

Other rare, but morphologically distinct, subtypes of DCIS are recognised. There is, however, no firm evidence to support the distinction of special DCIS types from commoner DCIS forms with the exception of encysted papillary carcinoma in situ and apocrine DCIS. The practical problem of inter-observer disagreement in distinction of some special DCIS subtypes, particularly apocrine and micropapillary DCIS, has led to some suggesting a working classification of DCIS with five subtypes; high, intermediate and low grade, with additionally apocrine and micropapillary DCIS as separate categories. Simultaneous use of the grading system described above and subtyping according to architecture is suggested.

***Apocrine DCIS***

The tumour cells show abundant granular cytoplasm, moderate to severe cytological atypia and central necrosis. Apical snouting (cytoplasmic protrusions) are not always seen. The cells may sometimes be highly atypical and no necrosis may be evident. The suggested diagnosis of apocrine DCIS should be made with caution particularly in the absence of comedo type necrosis. It may be extremely difficult to distinguish atypical apocrine hyperplasia from low grade apocrine DCIS. Benign apocrine change is, of course, frequent in breast biopsy material and is recognised to show nuclear atypia, which should not be interpreted as DCIS. Atypical apocrine adenosis may also mimic apocrine DCIS or even invasive apocrine carcinoma. Identification of periductal inflammation and fibrosis may be helpful and is rarely seen in atypical apocrine hyperplasia or apocrine proliferations other than DCIS.

For peer review only

### *Encysted Papillary Carcinoma in Situ*

This is a rare but distinctive form of DCIS, which is more common in older women. It carries an excellent prognosis if confined within the capsule without surrounding DCIS or foci of invasion. The presence of associated DCIS in the surrounding tissue is recognised to be of significance regarding local recurrence and should be recorded. Encysted papillary carcinoma in situ is usually circumscribed and accompanied by a hyalinised fibrous wall giving an encysted appearance. Adjacent to the fibrous capsule haemosiderin (or haematoidin) pigment is often seen. Encysted papillary carcinoma has a papillary structure with fibrovascular cores, which are usually fine but may be absent in at least part of the lesion. Other forms of DCIS, usually of micropapillary or cribriform type, may accompany it.

### *Clear Cell DCIS*

This is an intraductal proliferation of neoplastic cells with optically clear cytoplasm and distinct cell margins forming cribriform and solid structures. Central necrosis may be present. This may be mimicked by poor fixation in other forms of DCIS and care should be taken to achieve optimum fixation, as noted above.

### *Signet Ring DCIS*

This is a very rare variant characterised by the proliferation of signet ring cells in solid or papillary growth patterns. The cytoplasm stains positive with diastase-resistant-PAS or alcian blue.

### *Neuroendocrine DCIS*

The lesion has an organoid appearance with prominent argyrophilia, resembling a carcinoid tumour. The neoplastic cells may be arranged in a solid pattern or may be papillary, forming tubules, pseudorosettes, palisades, or ribbons. Where solid, the proliferation is nearly always punctuated by fine fibrovascular cores. An eosinophilic cytoplasmic granularity or organoid spindle morphology is all supportive of the neuroendocrine phenotype. Because of the lack of microcalcification these tumours tend to present symptomatically, most commonly in elderly patients with blood stained nipple discharge. Immunohistochemical stains for neuroendocrine markers (chromogranin, PGP9.5, synaptophysin, NSE) may be helpful in diagnosis of this sub-type of DCIS.

### *Cystic Hypersecretory DCIS and Mucocoele Like DCIS*

This type of DCIS is a variant of micropapillary DCIS. The cells produce mucinous secretions, which distend involved duct spaces giving a cystic appearance. Microcalcifications are often a very prominent feature.

For peer review only

## Rarer Subtypes of DCIS (continued)

### *Flat (clinging) DCIS*

This lesion is becoming increasingly recognised and presents particular problems of recognition. It forms part of the spectrum of columnar cell alterations which range from common benign blunt duct adenosis/columnar cell alteration through atypical forms to flat in situ carcinoma which is believed by many authorities to be a variant of low grade micropapillary DCIS.

### *Necrosis*

Necrosis can be identified by the presence of cell ghosts and is eosinophilic and granular in nature. Karyorrhectic debris is seen. The definition of necrosis does not include single apoptotic individual cells.

### Microinvasive Carcinoma

The NHSBSP guidelines definition of microinvasive carcinoma should be used. There is typically a dominant and often extensive DCIS lesion with one or more clearly separate foci of infiltration into non-specialised interlobular stromal tissue, none of which measures more than 1 mm in diameter. Tumours fulfilling these criteria are rare and if there is doubt about the presence of invasion the case should be classified as pure DCIS only. Microinvasion is very rare in DCIS other than high nuclear grade. Cases of pure high or intermediate nuclear grade DCIS and those with comedo type necrosis should be extensively sampled to exclude microinvasion or foci, which are more than 1 mm in size and represent established invasion. In particular, care should be taken to avoid overdiagnosis of cancerisation of lobules as microinvasive carcinoma.

The organoid appearance of cancerisation of lobules should be sought and deeper H&E sections from the paraffin block may be more helpful than immunohistochemical examination. However, stains that label myoepithelial cells (alpha-smooth muscle actin and myosin or cytokeratin 14,) or the basement membrane (laminin and Collagen IV) may assist in the diagnosis, as these will be absent on invasion fronts.

For peer review only

**Atypical Ductal Hyperplasia**

Atypical Ductal hyperplasia (ADH) is a rare lesion often co-existing with fibrocystic change, a sclerosing lesion, or a papilloma. The current definition rests on identification of the presence of some but not all of the features of DCIS. This is an area of diagnosis where even expert pathologists differ and acceptable diagnostic agreement is difficult to achieve. The difficulty is encountered in distinguishing ADH from low grade variants of DCIS. If pathologists adhere to the view that when cellular changes typical of DCIS occupy two separate duct spaces this is regarded as DCIS but if only one duct space is involved the disease is classified as ADH, then the majority of cases are more readily categorised; ADH is a small microfocal lesion. Two simple rules of thumb are helpful: a) not to entertain the diagnosis of ADH unless low grade DCIS has been seriously considered in the differential diagnosis and b) the larger the lesion the greater the likelihood that it is DCIS.

Tavassoli and Norris have argued that the overall geometric size of the DCIS lesion is more important than the number of duct spaces involved. Two mm in size was used as the cut-off point for the decision between ADH and DCIS in earlier published articles, a lesion less than 2mm in overall size indicating ADH. Page and co-workers have used a 3mm size cut-off in sub-classification of atypia within papillomas.

The AFIP criteria have been updated and the 2mm size criterion is now invoked *only* when assessing non-necrotic atypical intraductal proliferations with both architectural and cytological features similar to those of low grade DCIS. Proliferations with high grade cytology (with or without necrosis) qualify as DCIS, regardless of size or quantity of epithelial proliferation. Lesions that show comedo necrosis or high nuclear grade are not classified as ADH. Other pathologists would also classify a lesion as DCIS if the cellular changes typical of DCIS were present in only one duct space or if comedo type necrosis was present in the duct space in question even if the lesion was less than 2.0mm in size.

For peer review only

**In Situ Lobular Neoplasia**

The defining cell type in lobular neoplasia is round, cuboidal, or polygonal with clear or light cytoplasm. Nuclei are round to oval, cytologically bland, with an occasional small nucleolus. Mitotic figures and hyperchromatism are not often seen. There is an even distribution of cells and cellular monotony is the rule. Cytoplasmic clear vacuoles are often, although not invariable, present.

The diagnosis of in situ lobular neoplasia rests on finding the characteristic cells within *lobules*. Cytologically identical cells may focally involve the ducts in a 'pagetoid' manner. This proliferation of neoplastic cells above the basement membrane undermines the normal lining epithelial cells.

On occasions a regular, evenly-spaced monotonous population is seen within both ducts and lobules; in these circumstances it may be difficult to classify the lesion. If only scanty terminal ducts are involved and the proliferation is almost entirely lobular, the lesion is classified as in situ lobular neoplasia. However, distinguishing DCIS from lobular neoplasia may indeed be impossible if both an organoid lobular and ductal component are identified. If both ducts and lobules contain epithelial proliferation of this type, categorisation as both lobular neoplasia and DCIS is recommended at the present time to infer the precursor risk of DCIS and the bilateral cancer risk of lobular neoplasia.

**Receptor Measurement**

The methods used for receptor assay should follow national guidance. Laboratories performing such assays should participate in the relevant UK NEQAS scheme.

For peer review only

## COMPLETING THE SLOANE PROJECT PATHOLOGY FORM

One pathology data form should be completed for one episode of DCIS. It is presumed that this will be completed by the pathologist once complete excision of a lesion has been achieved.

**Demographic Details:** Please indicate surname, forename, date of birth, hospital where pathology was reported and hospital number, also screening number (if known).

**Therapeutic Excision Report Number:** It is recognised that some patients may have undergone multiple biopsies for diagnosis and therapeutic purposes. It is not necessary to provide all these data but please indicate the therapeutic excision report number for reference.

Side: Indicate left or right breast. For specimens from both sides, a separate form should be completed for each side.

**Specimen Type:** Choose one of the following terms:

**Core biopsy**

**11 gauge core biopsy (e.g. Mammotome)**

**Diagnostic open biopsy -** Includes lumpectomy, tylectomy, dochectomy etc, taken for diagnostic rather then therapeutic purposes.

**Therapeutic excision -** Includes lumpectomy/wide local excision, wedge excision, segmentectomy and partial mastectomy specimens with the aim of complete excision of the lesion.

**Bed biopsy/cavity shave -** A specimen shaved from one or more edges of the biopsy cavity of a therapeutic excision sent separately by the surgeon.

**Immediate re-excision specimen -** A re-excision specimen, of a specified margin, taken at the time of therapeutic procedure.

**Delayed re-excision specimen -** A re-excision specimen, usually of biopsy cavity and a specified margin, taken at a subsequent surgical procedure.

**Completion mastectomy -** Mastectomy performed for the purposes of obtaining complete excision as a subsequent surgical procedure.

**Mastectomy**

**Specimen weight:** Record the weight of any specimen if known. Weight is more reproducible than 3 dimensional measurements to determine volume, even taking into account the different densities of fat and fibrous tissue, which vary in proportion in breast specimens. Specimen weight is also used as the means of determining the likely cosmetic disadvantage to women undergoing benign biopsy in the NHS BSP.

For peer review only

**Nipple Orientation marker present?** Please indicate whether the surgeon has separately identified the nipple margin of the therapeutic excision. Surgical guidelines for the Sloane project recommend that this be undertaken.

**Other Orientation markers present?** Please indicate whether the surgeon has appropriately identified other margins of the specimen, e.g. with sutures of described length or clips, so that orientation is clear to the pathologist.

**Cut up/specimen information (for wide local excision specimens)** Please indicate how the therapeutic excision specimen has been sampled and which Sloane cut up protocol was used (see above). If another technique was used it would be helpful if the protocol was described and the protocol used was provided.

**Were specimen slices X-rayed?** Please indicate whether, after slicing, the specimen was re-X-rayed.

**Was a macroscopic abnormality present?** Some cases of DCIS may present as a mass and sampling may be targeted at a specific macroscopic abnormality. Please indicate if this was the case.

**Number of blocks taken to assess margins and number of blocks taken overall:** Please record the number of blocks taken specifically to assess whether the margins of excision were clear of DCIS and the overall number of blocks taken of the wide local excision. This will clearly vary according to the mode of sampling and the size of the specimen. In some cases it may not be possible to specifically ascribe a block as taken to assess the margin or the lesion; if these include the margin of excision they should be recorded in both sections.

**Histology:** Please indicate whether DCIS, ADH and lobular neoplasia are present. Tick all that are identified.

**The subsequent sections refer to DCIS ONLY**

**Maximum (total) size of lesion mm:** Please indicate the total size of the DCIS. This may be a summation from the total in several specimens e.g. diagnostic, therapeutic and re-excision samples, and may therefore necessarily be an approximation.

**Highest nuclear grade:** Rarely DCIS may demonstrate more than one nuclear morphology, please indicate the highest nuclear grade present.

For peer review only

**Growth pattern:** Please indicate **all** architectural patterns of DCIS present from solid, micropapillary, cribriform, papillary, apocrine, flat/clinging or other. In addition please record any special features such as neruoendocrine phenotype or clear cell morphology.

Comedo DCIS is considered to represent solid DCIS (usually of high nuclear grade) with central necrosis and is not therefore included as a specific growth pattern.

**Comedo Necrosis present:** Indicate whether there is central necrotic debris present. Individual cell death is not considered to represent necrosis and should not be recorded.

**Paget's disease present:** Indicate whether there is associated Paget's disease of the nipple in specimens which include nipple and skin. If nipple is not included in the sample please tick not applicable/not known.

**Microinvasion (<1mm) and number of foci of microinvasion?** Indicate whether there is no, definite or possible microinvasion, seen as one or more clearly separate foci of infiltration into non-specialised interlobular stromal tissue, none of which measures more than 1mm in diameter. Please also indicate the number of foci of definite microinvasion.

**Margins**

**Distance to margins in therapeutic excision:** Please record the distance of DCIS to margins in the therapeutic surgical procedure. Please complete all available; it is recognised that not all of the measurements will be available in all cases. If any/all of the margins are more than 10mm distant this is sufficient.

**If altered by further surgery:** The margins may have been altered by subsequent surgery/re-excision. Please also provide estimated final distance to specific margins if further surgery has altered the measurement from the therapeutic procedure.

**Does another process extend to the margin? Distance of ADH of lobular neoplasia to margin:** The clinical significance of ADH and lobular neoplasia at the margins of excision is unclear. Please indicate whether ADH and /or lobular neoplasia are present at the margin and the distance of this process to the nearest margin of excision.

For peer review only

**Immediate re-excision/cavity shaves/bed biopsies:** Please record the number of samples received from the surgeon and the total number of blocks taken from these specimens.
Please indicate whether DCIS, ADH, and/or lobular neoplasia were present in these additional specimens.

**Nodes:** Please record the number of nodes examined and the number positive from the axilla, as sentinel nodes or others, such as internal mammary nodes.

**Receptor Status:** Please record the oestrogen and/or progesterone receptor status and HER2 status, using the cut offs described on the data form. Alternatively indicate if not known/not performed.

**Date and pathologist:** Please record the data of completion of the form and print your name.

For peer review only

The Sloane Project

Understanding non-invasive breast disease

NHS

# Sloane Project Atypia Audit

# Radiology Protocol

For peer review only

Published July 2014

Version 1.0

www.sloaneproject.org.uk

Operated by Public Health England

For peer review only - http://bmjopen.bmj.com/site/about/guidelines.xhtml

The Sloane Project
Understanding non-invasive breast disease

NHS

# Guidelines for completion of Sloane Project Radiology data form

## Demographic Details

Please indicate date of birth, screening unit, screening number (SX number), screening round number (this is the number of times the patient has been screened as part of the NHS Breast Screening Programme), date of screening mammogram and NHS Number. The surname and forename boxes are for use within the Trust that is entering the patient, and should be crossed out before returning to the Sloane Project Manager.

## Side

Indicate left or right breast. A separate form is required for each side in the cases of bilateral disease. For multi-focal disease in a single side, only one form should be completed.

## Site

Please indicate all sites that are involved from the following: Upper Outer Quadrant, Upper Inner Quadrant, Lower Outer Quadrant, Lower Inner Quadrant and Retroareolar.

For multi-focal disease, it is important that there is concordance of the site which is reported on the pathology form with that which is measured on the radiology form. As these cases are being identified at the postoperative MDM, correlation should take place at this time.

## Background Pattern

During the pilot phase of the radiology data collection a number of classifications were used to describe the background parenchymal pattern. Recognising that this is a highly subjective classification, the pilot centres agreed that the birads classification was the most reproducible.

Please therefore indicate which one of the following patterns best describes the Background Pattern: Fatty, Scattered Fibroglandular Density, Heterogenously Dense and Extremely Dense.

## Predominant Radiological Feature

Please choose the predominant radiological feature on the mammogram and classify as appropriate. Please tick one box only of the following features: Calcification, Parenchymal Distortion, Mass-Well Defined, Spiculate Mass, Mass-Ill Defined or None of the above.

For peer review only


Operated by Public Health England

## Calcification

With regard to the classification of calcification, two systems of classification have been included ~ please choose one system only and examples of the three groups are enclosed in picture format at the end of this document.

To clarify, however, the classification is based only on the shape of the individual calcifications. i.e. the presence or absence of a prominent ductal distribution should not influence the classification.

1. **Casting/linear:** this should include any case where there is even a single linear or branching calcification even if the rest are granular or punctate in shape.
2. **Granular/crushed stone:** this should include cases which have irregularly shaped calcifications but no linear forms. Some or even most of the calcifications may be punctate.
3. **Powdery/punctate:** All calcifications are either round or oval in shape.

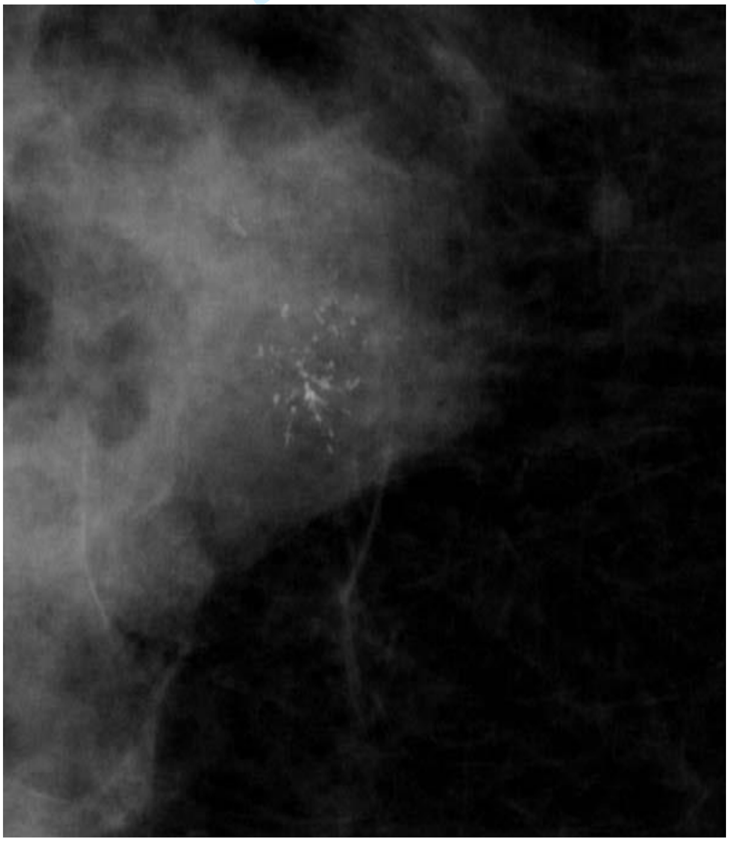

Figure 1. Localised casting type calcification

Operated by Public Health England

For peer

For peer review only - http://bmjopen.bmj.com/site/about/guidelines.xhtml

The Sloane Project
Understanding non-invasive breast disease

NHS

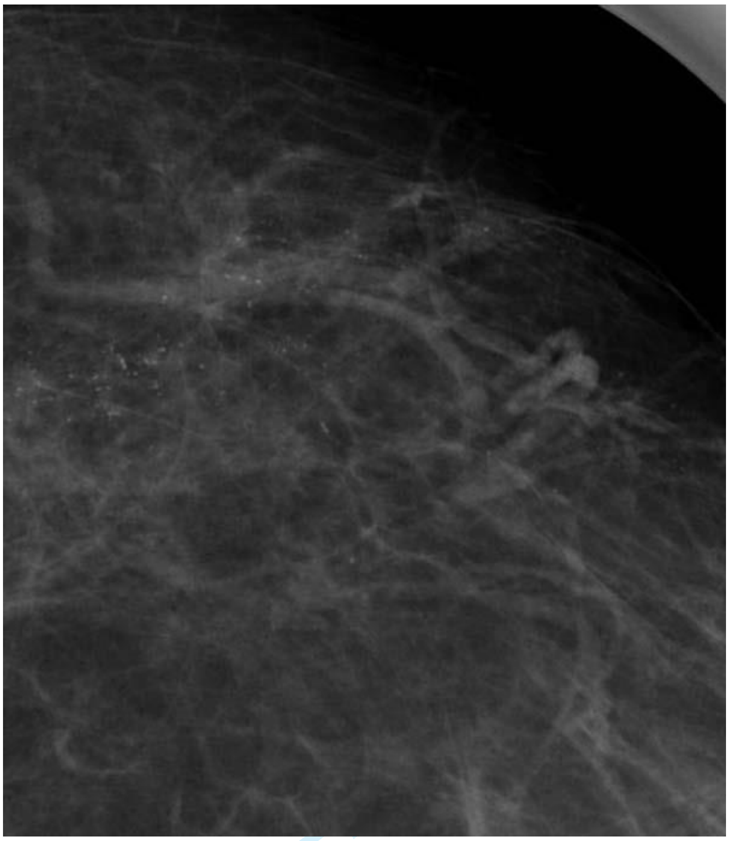

Figure 2. Extensive granular (Nottingham classification) or Crushed Stone (Tabar classification) calcification

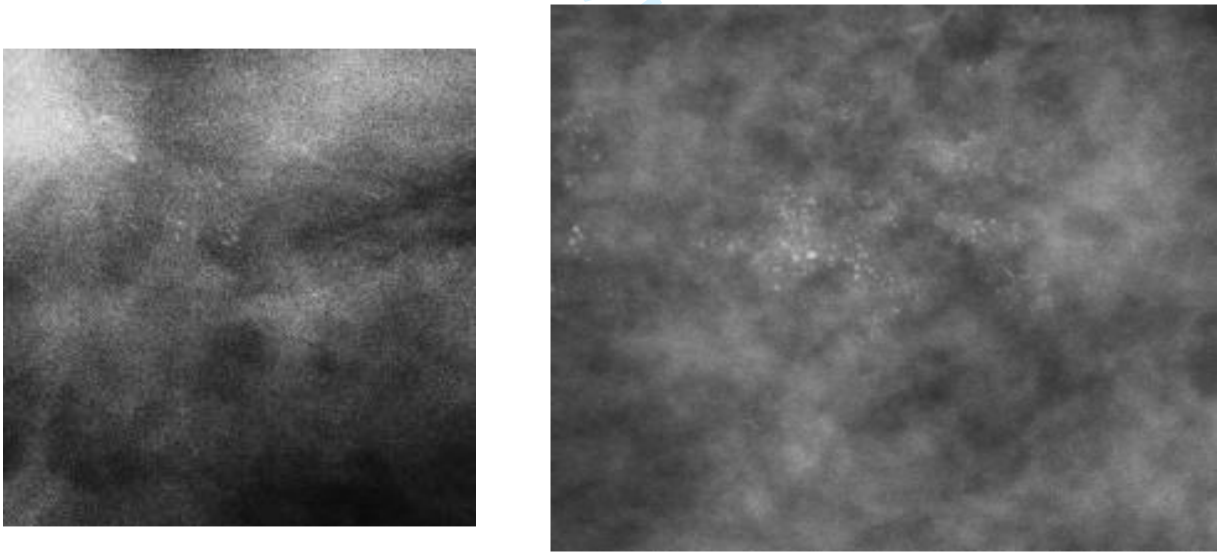

Figure 3. Punctate (Nottingham classification) or Powderish (Tabar classification) calcification


Operated by Public Health England

The Sloane Project
Understanding non-invasive breast disease

NHS

## Estimated Size of Lesion on Mammograms

All measurements have to be done both on the oblique view and the CC views. Three reproducible measurements have been chosen:

1. **Distance from base of nipple to nearest part of the lesion (mm)** i.e. border of mass/first dot of calcium, closest area of parenchymal distortion (see Figure 4 for example).
2. **Maximum length of lesion (mm)** (see Figure 5 for example).
3. **Maximum diameter of lesion at $90^0$ to long axis of lesion (mm)** (see Figure 6 for example)

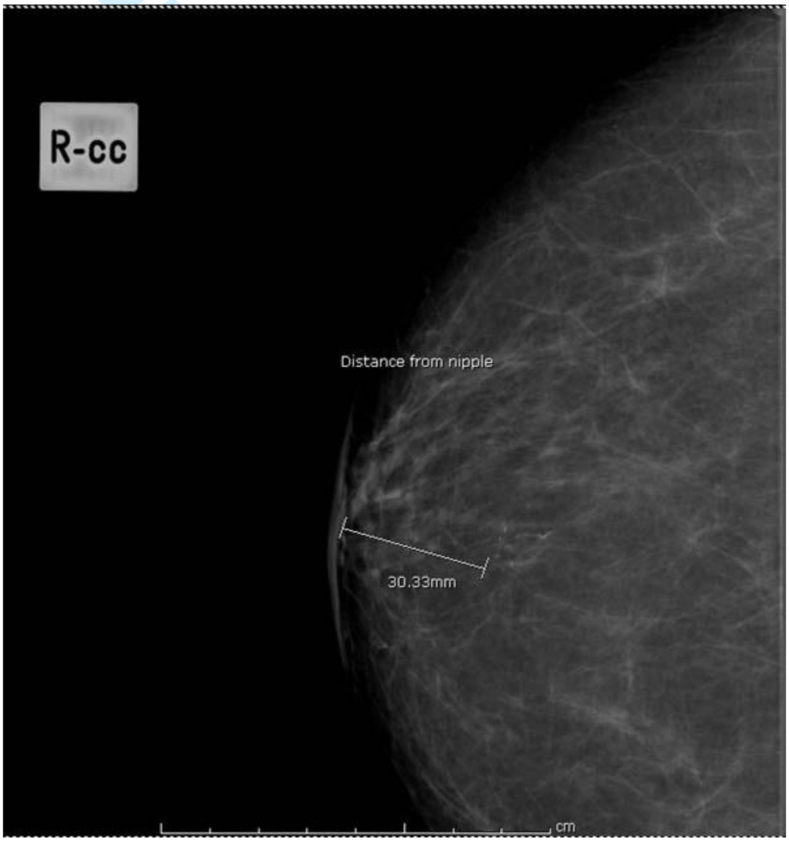


Figure 4. Measuring distance from nipple to lesion


Operated by Public Health England

The Sloane Project
Understanding non-invasive breast disease

NHS

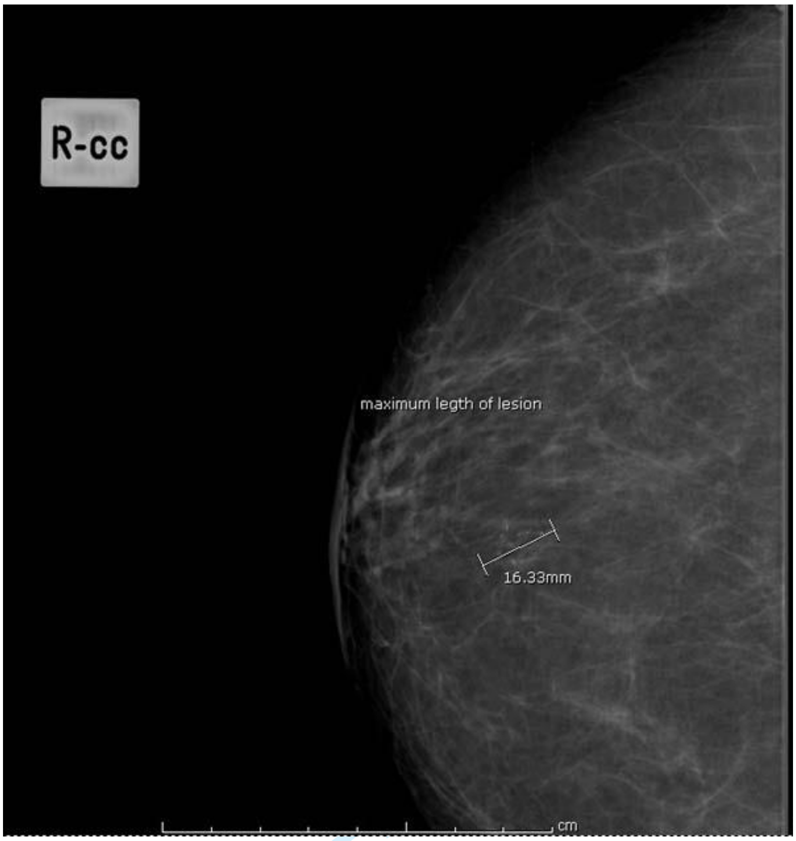


Figure 5. Measuring maximum length of lesion (mm)

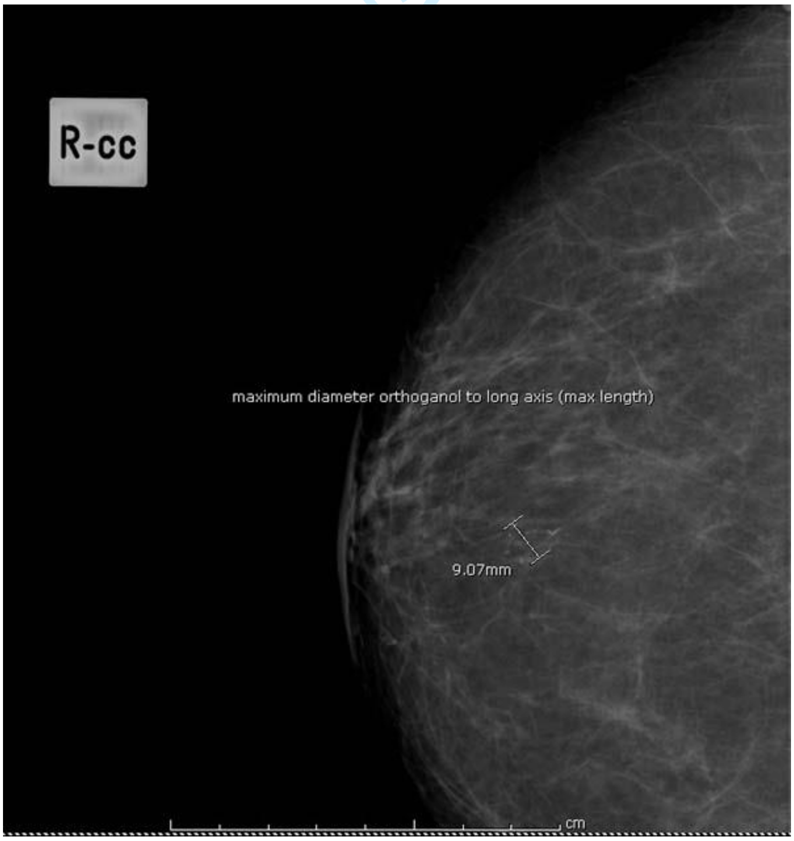


Figure 6. Measuring maximum diameter of lesion (mm)


Operated by Public Health England

Please note that as cases of distortion are few and far between and because of difficulties in relation to measuring these cases, it has been agreed that measurements of distortions are no longer required. This includes measuring distance from nipple.

In cases where there are two distinct areas separated by apparently normal breast tissue, then the measurement should be from one end of one site to the further most end of the other site, along with all the apparently normal breast tissue in between. The reason for this is that it reflects how we clinically manage such cases (i.e. if DCIS is found in two sites with apparently normal breast tissue in between the patient will generally be subjected to mastectomy) (See Figure 7).

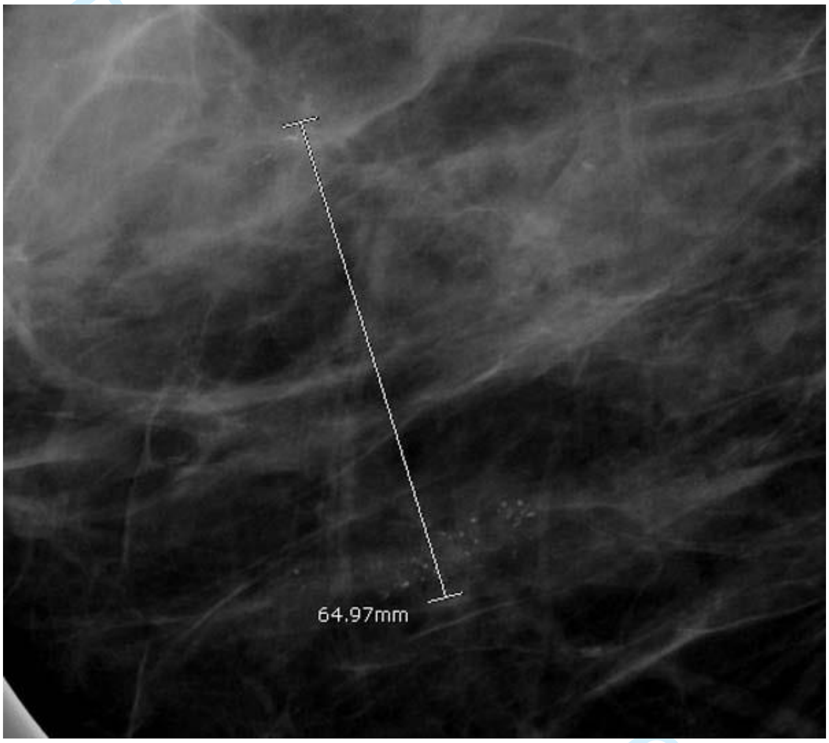


Figure 7. Measuring multi-focal disease

## Data required to Calculate Breast Volume

The accompanying formula (1/3 pi $r^2h$) has been validated and is thought to be the closest approximation to breast volume that can be carried out from 2 view mammography. The labels “a” and “b” correspond to the measurements “h” and “2r” that are required for the breast volume formula.

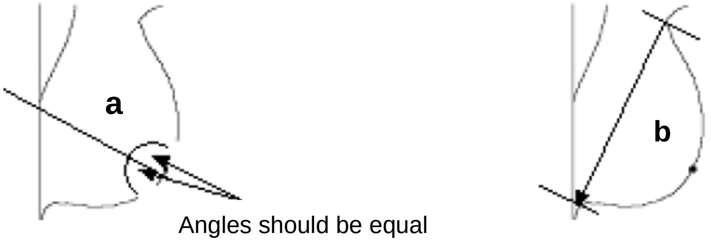


**Chest wall to nipple (in the nipple plane)** **Infra mammary fold to supra mammary (axillary) fold**

For clarification, the diagram of the MLO shows the perpendicular from the nipple running through the intersection of the anterior border of the pec major and the back of the image. When the mammographic image doesn't completely coincide with this diagram, please measure the perpendicular to the back of the image rather than the front of the pec.

Please note that even though the size of the lesion and distance from the nipple are not to be measured in cases of distortion, the data to calculate breast volume is still required.

## Magnetic Resonance Imaging (MRI)

Please indicate whether a pre-operative MRI was performed, and the estimated maximum dimension of the lesion on MRI (mm).

## Specimen X-Ray

Please indicate whether a Specimen X-Ray was carried out and identify by tick whether the specimen is as a result of a diagnostic or therapeutic biopsy. Please record whether the lesion is present in the Specimen X-Ray and if it appears to have been radiologically completely excised. With regard to completeness of excision, arbitrarily 2mm (unmagnified) of "normal" breast tissue has to be present between the margins which are closest to the mammographic abnormality.

With the increase in the use of mammotomy to diagnose these cases pre-operatively, there is the possibility that in some cases all the calcifications will have been removed and that a clip will be left in situ. For the purposes of completion of this section, it is possible to say that the entire lesion has been removed, but it is inappropriate to make measurements as to proximity to the margin etc.

Further radiological guidelines can be found on the Sloane Project website (www.sloaneproject.org.uk) Radiology Training Resource page or via the Sloane Project Manager.

For peer review only

The Sloane Project
Understanding non-invasive breast disease

NHS

# Sloane Project Atypia Audit Pathology Protocol

For peer review only

Published April 2019

Version 2.0

phe.sloaneproject@nhs.net

For peer review only - http://bmjopen.bmj.com/site/about/guidelines.xhtml

# Guidelines for Pathological Assessment of Atypical Intra Ductal Epithelial Proliferation (AIDEP), Atypical Ductal Hyperplasia (ADH), Lobular In Situ Neoplasia (LISN), Pleomorphic LCIS (PLCIS) and Flat Epithelial Atypia (FEA)

For peer review only

## Introduction

The Sloane Project Phase One Pathology Protocol (available on request), contains detailed information about surgical and laboratory handling of therapeutic surgical specimens, including sampling techniques and cut-up protocols. It also describes the assessment and measurement of margins and what is required from the histological examination. The Sloane Phase One protocol largely refers to ductal carcinoma *in situ* (DCIS), which was the predominant type of screen-detected lesion recorded in the first phase of the Sloane Project. This protocol refers to the ongoing collection of screen-detected atypia of the breast by the Sloane Project. The Sloane Project no longer collects data on patients with DCIS primaries.

## Specimen Handling

This Sloane Project Atypia Audit Pathology protocol gives some more detailed and updated guidelines for the pathological assessment of atypical ductal hyperplasia (ADH), lobular in situ neoplasia (LISN), pleomorphic LCIS (PLCIS) and flat epithelial atypia (FEA) which are the screen-detected lesions being recorded in the second phase of the Sloane Project (atypia audit). It is anticipated that these 'atypical' lesions are more likely to arise in *diagnostic* (rather than therapeutic) surgical specimens, as well as core biopsies and vacuum-assisted wider gauge samples; thus specimen handling for these may be different from DCIS lesions and pleomorphic LCIS. Such specimens may not be orientated by the surgeons who aim to remove the screen-detected abnormality, without surrounding disease-free margin (less than 20g tissue). For this reason such specimens may not requiring inking in different colours and distance to margins of these atypical lesions may not be applicable. Further guidance can be found in the updated NHS BSP publication 58.

The Sloane Project
Understanding non-invasive breast disease

NHS
Cancer Screening Programmes

## Core Biopsy and Diagnostic Vacuum Assisted Biopsy

Such specimens, typically for the diagnosis of screen-detected microcalcification, require adequate fixation in sufficient volume of formalin, routine processing and examination at three levels with haemotyxlin and eosin-stained sections, as per NHSBSP guidelines. Correlation of the histological and radiological extent, degree and size of the microcalcifications at multidisciplinary team meeting is essential. Additional levels may be advised in the assessment of some of these borderline atypical lesions.

## Therapeutic Vacuum-Assisted Biopsy

In some centres, after initial narrow (e.g. 14g) or wide gauge (e.g. 8 or 11g) core biopsy, the individual Unit protocol for 'B3' lesions may be that wider, vacuum-assisted *excision* biopsy be performed. As for *diagnostic* vacuum-assisted samples, these should be adequately fixed and all the cores examined with haematoxylin and eosin-stained sections. When the diagnosis is of flat epithelial atypia or atypical ductal hyperplasia, it is anticipated that additional levels will often be performed to exclude the diagnosis of low grade DCIS and/or associated invasive carcinoma.

## Diagnostic Localisation Excision Biopsies

These specimens are produced when a definitive pre-operative diagnosis of malignancy has not been made but where there are suspicious radiological or clinical findings, or when the pre-operative diagnosis is of an indeterminate (e.g. C3/B3 or C4/B4) lesion.

- The specimen should be inked, weighed, measured in three dimensions and then, usually, serially sliced at intervals of approximately 3-5mm.
- Specimens containing impalpable mammographic lesions, such as microcalcification or sometimes a tissue marker, will require specimen slice x-ray examination if a block selection process is undertaken (i.e. those that are not embedded in their entirety). This enables blocks to be taken from the areas corresponding to the mammographic abnormality as well as any other suspicious areas identified.
- The sites of sampling can be marked on the specimen x-ray, or the x-ray of specimen slices, by using a white wax (*Chinagraph*) pencil or other marker if hard copy film is used. Digital images can also be annotated to indicate sites of block selection.
- The sampling technique and the number of blocks taken are clearly dependent on the size of the specimen and the size of the abnormality.  If the specimen is small (e.g. less than 30mm), it is best to block and examine all of the tissue.



For peer review only - http://bmjopen.bmj.com/site/about/guidelines.xhtml

- Blocks should be taken ideally to enable assessment of the histological size of the lesion. Where the maximum macroscopic dimension of a tumour can be blocked directly, it is recommended that a single block across this aspect be taken.
- Where a lesion is larger than can be assessed in a single block, a large (mega) block to encompass the maximum dimension may be taken. When taking large blocks at least one other normal sized lesional block should be processed as well, to allow optimal processing and to avoid the excessive use of antibodies in any immunohistochemistry.
- If large blocks are not available, two or more blocks are recommended from the maximum macroscopic dimension, so that the total lesion size can be estimated by adding the portions together or measuring the maximum dimension on the two slides fitted together. A diagram may be made of how the adjacent blocks relate to each other to avoid misunderstanding of this relationship at the time of microscopy, with the risk of overestimating the lesion size. Occasionally, when the plane of maximum dimension of a non-spherical lesion is not known or is anticipated incorrectly, it may be necessary to stack together the estimated block thicknesses along that axis, to obtain a measurement. This will always be fairly inaccurate and the need to try to find the plane of maximum dimension of the tumour prior to slicing is important.
- For larger specimens, sampling should be adequate to determine accurately the size of the lesion. Sampling should include the extremes of the mammographic abnormality and adjacent tissue in order to avoid underestimation of size. This is particularly important with cases that prove to be DCIS (e.g. including cases with a pre-operative core biopsy containing an atypical ductal epithelial proliferation) as it is recognised that mammographic size may be an underestimate of true size.
- If specimens are sent as more than one piece of tissue, it can be impossible to measure the absolute extent of the lesion. In these cases, it is appropriate to take a pragmatic approach and to measure the maximum size in each piece of tissue and add the dimensions to give an estimated total size. If, however, the orientation of the specimens can be determined, the true size can be ascertained more reliably.
- If calcifications cannot be seen histologically, specimen block x-ray may be helpful to determine their location in the sampled tissue.

## Therapeutic Excisions

It is considered unlikely that (other than for pleomorphic LCIS) the atypical lesions to be recorded in the Sloane Atypia Audit will be seen alone in a therapeutic excision specimen. However, lesions that have a pre-operative diagnosis of malignancy (DCIS or pleomorphic LCIS) and are deemed to be suitable for breast conserving surgery with regard to clinical/radiological size may be excised as a therapeutic wide local excision.



For peer review only - http://bmjopen.bmj.com/site/about/guidelines.xhtml

- It is usual for the surgeon when performing a therapeutic operation to take all of the tissue from the subcutaneous aspect to the pectoral fascia as per Surgical Guidelines for the management of breast cancer. It is essential that the pathologist be informed if the usual surgical protocol has not been undertaken as this will affect the optimum specimen handling methodology e.g. central excisions. In such circumstances, when tissue remains at the deep (posterior) or superficial (anterior) aspects of the specimen, the distance to these margins is clinically relevant.
- The surgeon should orientate cancer resection specimens. Each unit should establish a code of orientation using either different lengths or numbers of sutures or metal staples/clips or ink. The code should be anatomically relevant and assist in accurate evaluation of the specimen and its margins. The nipple extension/direction of the nipple should ideally be separately marked by the surgeon and it is helpful if the request form indicates the site of the tumour within the breast so that the radial margin of the specimen nearest the nipple can be identified more easily.
- The specimen should be weighed and measured in three dimensions.
- The specimen excision margins should be inked and the specimen can be sliced either before fixation or (less preferably) after fixation.
- The technique for examining the specimen and sampling the abnormality will vary according to type of sample and the lesion nature (e.g. mass, calcification etc) and lesion size and also according to pathologist/laboratory preference; therefore a degree of flexibility is required. Several options are available. Whichever is utilised, as an absolute minimum, the information for the breast cancer minimum dataset, including accurate measurement of size and detailed examination of the margin status and distance to margins, must be provided.

## Wide Local Excisions for lesions presenting as mammographic calcification

Pleomorphic LCIS often also presents as radiologically-detected calcifications and it is presently recommended that such specimens are handled as per those with a pre-surgical diagnosis of DCIS

- DCIS and pleomorphic LCIS typically present as a mammographically detected abnormality, usually calcification, which may not be visible on macroscopic examination of the sliced tissue. Specimens from patients presenting with such lesions will therefore benefit from a combined radiological-pathological approach to diagnosis.
- It is usual for the surgeon when performing a therapeutic operation to take all of the tissue from the subcutaneous aspect to the pectoral fascia. It is essential that the pathologist is informed if the usual surgical protocol has not been undertaken as this will affect the optimum specimen handling methodology e.g. central excisions, or specimens where breast tissue remains at the



For peer review only - http://bmjopen.bmj.com/site/about/guidelines.xhtml

deep (posterior) and superficial (anterior) aspects of the excision, and the distance to these margins is thus clinically relevant.

- The surgeon should mark the nipple duct margin; DCIS tracks towards the nipple and, in this plane in particular, can be some distance from the obvious area of microcalcification. It is not known whether pleomorphic LCIS demonstrates a similar growth pattern. It is helpful if the request form indicates the site of the lesion within the breast so that the margin of the specimen nearest the nipple can be identified.
- The specimen should have been X-rayed intra-operatively prior to receipt in order to confirm the presence of the lesion within the specimen. The intraoperative radiographs – whether digital or hard copy film - should be available to the pathologist who should be aware of the size and location within the specimen of the radiological abnormality
- The specimen should be weighed and measured in three dimensions.
- The specimen excision margins should be inked and the specimen can be sliced either before or after fixation. The use of different colour inks/markers on an individual section can assist microscopic identification of specific margins. Inks which are radio-opaque should ideally be avoided if applied prior to slice X-ray.
- If the specimen is large, then incision before fixation is recommended. The specimen should be sliced at intervals of approximately 3-5mm
- Serial slicing enables specimen slice radiographic mapping of the specimen which provides a high level of confidence that the lesion has been accurately and adequately sampled; slicing and X-raying the specimen slices enables blocks to be taken most accurately from the areas corresponding to the mammographic abnormality as well as from any other suspicious areas identified. This is essential to avoid underestimation of lesion size and overestimation of the distance to specimen margins. Sites of sampling can be annotated on the specimen radiograph for radiological-pathological correlation.
- Macrophotography or schematic diagrams may also assist in recording macroscopic findings and the block map as well as identifying individual sampled margins.
- Sampling may be facilitated by the identification of any radiological marker (e.g. clip, collagen marker or coil). Tissue changes relating to previous core biopsy are an important landmark to indicate sampling of the site of the index lesion and should be recorded in the report, particularly if the whole abnormality was removed by the cores.
- The macroscopic and or radiographic lesion should be described and its size in 3 dimensions and distance to margins recorded.
- The number of blocks taken will depend on the size of the specimen and the size of the abnormality. If the specimen is small, or if slice radiology unavailable, it is best to block and



For peer review only - http://bmjopen.bmj.com/site/about/guidelines.xhtml

examine all of the tissue.  Samples 30mm or less in maximum dimension should be completely sliced, embedded and examined histologically. The site of all blocks taken should be recorded.

- For larger specimens sampling should include the extremes of the radiographic calcification and adjacent tissue in order to avoid underestimation of the size of the lesion.  This is particularly important for microcalcifications associated with DCIS, as it is recognised that mammographic size may be an underestimate of the true DCIS size. Comparable data is not available for pleomorphic LCIS but it is recommended that a similar approach for specimen handling to that for cases of DCIS is applied at present.
- Representative samples from the entire involved area should be taken. Sufficient blocks should be taken to identify associated invasive carcinoma if present.  Samples should include areas of breast tissue from proximal (towards the nipple) and distal to the calcification, as DCIS extends more frequently in this plane; again, there is no data to indicate whether the same is true for pleomorphic LCIS. Defining the minimum number of blocks that should be taken remains unclear, however it is recommended that one to two block per 10mm of the maximum dimension of the area of calcification be taken. Measurement can be made in this way from the most distal involved duct across the main area of calcification to the most proximal involved duct.
- Many units use large blocks to embed the entirety of segmental excisions although the proper processing of these can delay the reporting of the case and storage may also be problematic; units should therefore take a pragmatic approach.
- All surgically relevant margins of therapeutic excision specimens should be sampled. This will include all radial/circumferential margins (superior, inferior, medial, lateral and nipple margins), and the deep (posterior) and superficial (anterior) if dictated by local protocol or by the surgical procedure from an individual patient. Particular attention should be paid to the margin nearest the mammographic abnormality and the margin nearest the nipple.
- If therapeutic samples are sent in more than one portion, it can be extremely difficult to measure the absolute largest extent of the whole lesion present. In these cases, it is appropriate to measure the maximum distance in any piece of tissue and to add the dimensions to give an estimated total size.  If, however, the orientation of the specimens can be determined, the size can be ascertained more reliably.

## Cavity Shave/Biopsy Specimens

- The circumferential edge of a wide local excision specimen can be shaved and examined by the pathologist to allow more extensive examination of the relevant surgical resection margins. This is typically done after taking the radial tumour blocks, and particularly if a distance of more than 2mm to the surgical margins is required for completeness of excision in local protocol. This can



For peer review only

produce a series of additional blocks including: superior shave, supero-lateral shave, lateral shave, infero-lateral shave, inferior shave, infero-medial shave, medial shave and supero-medial shaved edge, depending on the size of the specimen. This peripheral shaving technique is not applicable to the anterior or posterior aspects; narrower margins are often acceptable at these aspects and widths of normal tissue of less than 2mm are regarded as complete excision.

- Alternatively, the surgeon may provide separate cavity shaves, which may be submitted to the laboratory as 'bed biopsies'. The site of each specimen should be clearly labelled and each specimen examined separately.
- It should be noted that shaved edged of the margins of the specimen or examination of 'cavity shaves/bed biopsies' assess adequacy of excision but do not allow measurement of distance between tumour and margins.
- Cavity shave specimens should be distinguished from more substantive margin re-excision specimens.
- Cavity shave specimens submitted by the surgeons are generally a sheet of fibroadipose tissue with marker suture or other marking on one surface. After weighing and measuring, paying particular regard to their thickness, their new external surface should be inked, taking great care not to ink the internal margin.
- Small cavity shaves should be blocked out in total. If of a larger size, such that this is impractical, they should be sliced and the densest or most suspicious areas blocked out.

## Re-excision Specimens

If a radiological abnormality extends close to a margin on an intraoperative specimen radiograph, the surgeon may undertake an immediate re-excision of that particular margin. Similarly, if there is a surgically palpable abnormality extending close to a margin intraoperatively during the operation, the surgeon may undertake an immediate re-excision of that particular aspect. A separate re-excision specimen may therefore be taken either: (1) at the time of initial surgery; (2) subsequent to the discovery of incomplete excision in a therapeutic excision for malignancy; (3) following diagnostic localisation biopsy when malignancy has been diagnosed. The aim of such a procedure is to remove either all of the previous biopsy site and its margins, or one or more specific margins known, or suspected, to be involved by the disease process. Whenever re-excision has been performed, the surgeon should orientate the re-excision specimen.

- Re-excision specimens should be weighed, and serially sliced at 3-5mm.
- Blocks taken should be recorded in such a way as to permit accurate assessment of the adequacy of excision and size of any malignant lesions identified.



For peer review only - http://bmjopen.bmj.com/site/about/guidelines.xhtml

- It is difficult to be prescriptive regarding the extent of block sampling as the nature and size of these specimens varies; the focus should be on the new excision margin rather than exhaustive detection of residual disease.
- If re-excision specimens have been taken which contain further tumour, it can be extremely difficult to determine the absolute size of lesion. A pragmatic approach is required, and the maximum distance in each piece of tissue can be measured and added to give an approximate total size of tumour. If, however, the orientation of the specimens can be determined, the size of tumour can be ascertained more reliably.
- The pathologist should measure the distance of any additional tumour present to the new margin of excision, or to approximate the distance of the original tumour to the new margin of excision if no tumour is present.

## Histological Examination

2-4µm thick haematoxylin and eosin stained sections should be examined for all specimen types.

## Columnar Cell Lesions

Columnar cell lesions (synonyms: blunt duct adenosis, columnar cell change, columnar cell hyperplasia, unfolded lobule, columnar alterations with prominent snouts and secretions (CAPSS)), in broad terms include the spectrum of changes ranging from bland columnar cell change through columnar cell hyperplasia to flat epithelial atypia. They have become increasingly identified clinically as a consequence of more rigorous investigation of radiological calcification and are most commonly seen in association with fibrocystic change. They represent a spectrum of lesions:

- Columnar cell change
- Columnar cell hyperplasia
- Flat epithelial atypia
- Flat high grade in situ carcinoma

### Columnar Cell Change and Columnar Cell Hyperplasia

These entities are benign and are NOT included in the Sloane Audit data collection. However, the histological features are included here for completeness and for diagnosis consideration of the differences between these and flat epithelial atypia.

Classical columnar cell change is comprised of lobular acini lined by epithelial cells that are tall and snouted in a manner similar to that observed in tubular carcinoma. Commonly this is associated



For peer review only - http://bmjopen.bmj.com/site/about/guidelines.xhtml

with luminal secretions and/or microcalcifications. If there is a piling up of several layers the term '*columnar cell hyperplasia'* is used, assuming that the stratification is real, as opposed to artefactual as a consequence of cross-cutting. There is morphological diversity within these groups, for example the hyperchromasia of the nuclei can vary, as well as nuclear shape and the 'tallness' of the cells; thus in some cases some lesions are more cuboidal than columnar. In columnar cell change, lobules are often dilated and are lined by epithelial cells with a columnar morphology. Other features include increased cytoplasm and apical snouts. The associated luminal secretions often undergo calcification. A single layer of columnar epithelial cells is the norm, although minor multilayering and tufting may be present. If a greater degree of multilayering of the epithelial cells is seen, the process is classified as columnar cell hyperplasia. At present, this is considered to be equivalent to usual epithelial hyperplasia and data is not collected in the Sloane Audit.

## Flat Epithelial Atypia

If superimposed mild cytological atypia is seen within the columnar cell change in the terminal duct-lobular unit, the lesion is classified as flat epithelial atypia. In this lesion the cells are morphologically similar to those of atypical ductal hyperplasia/low grade DCIS but are present, typically in a single layer lining round, mildly dilated acini. The nuclei are typically round or oval and evenly spaced. The cells may have clumped chromatin or vesicular nuclei or prominent multiple nucleoli. Mitoses are infrequent. If there is marked cytological atypia is seen the lesion is regarded as flat high grade in situ carcinoma and NOT as FEA.

## Differential diagnosis of FEA

True micropapillary structures and rigid epithelial bridges are not seen in columnar cell change or columnar cell hyperplasia. If such architectural atypia, usually in the form of bulbous micropapillary structures, is identified, the lesion should be assessed for degree within the membrane-bound spaces and overall extent, and classified as atypical ductal hyperplasia or low grade DCIS accordingly.

Apocrine metaplasia may occasionally mimic FEA, and vice versa; both may have apical snouts and both line the acini. In such circumstances, immunohistochemistry for oestrogen receptors may be helpful; apocrine lesions are typically ER negative whilst columnar cell lesions are uniformly strongly ER positive.

As well as ADH/low grade DCIS, other epithelial proliferations may merge, or be associated, with columnar cell hyperplasia, including atypical lobular hyperplasia, lobular carcinoma in situ (LCIS) and invasive carcinoma, often of low grade tubular or tubulo-lobular type.



For peer review only - http://bmjopen.bmj.com/site/about/guidelines.xhtml

## Atypical intraductal epithelial proliferation (AIDEP)

There is a range of intraductal epithelial atypia short of that required for a definite diagnosis of ductal carcinoma in situ. The definition of atypical ductal hyperplasia (ADH) is derived from surgical resection specimens and relies on a combination of architectural, cytological and size extent criteria; ADH is defined as an intraductal epithelial proliferation showing the features of low grade DCIS, but in less than two duct spaces or less than 2 mm in diameter. For this reason, accurate diagnosis of ADH is not possible on core biopsy.

In order to preserve the specific (bilateral) increased risk of subsequent breast cancer conferred by the diagnosis of ADH, the term AIDEP is therefore recommended, both for lesions akin to ADH in core biopsy as well as some lesser degrees of atypical epithelial proliferation that are of sufficient to be of concern histologically.

## Atypical Ductal Hyperplasia

Atypical ductal hyperplasia (ADH) is a rare lesion. Its current definition rests on identification of some but not all features of DCIS. Difficulties are encountered mainly in distinguishing ADH from the low grade variants of DCIS. The diagnosis of ADH is based on both a *qualitative* and *quantitative* assessment of the lesion.

The *qualitative* assessment is based on cytological features and architectural growth pattern. These include:

- A uniform monomorphic luminal epithelial cell population (ER, CK8, 18, 19 positive, CK5, 5/6, 14 negative
- Even spacing of the nuclei
- Secondary lumina, some of which are rigid whereas others are tapering
- Hyperchromatic nuclei
- Cribriform, micropapillary or solid growth pattern.

The *quantitative* assessment is based on assessment of lesion size:

- Areas of ADH are usually microfocal/small, not exceeding 2–3 mm in size/involve less than two complete membrane-bound spaces.

**Proliferations with high grade cytology qualify as DCIS, regardless of the size or quantity of epithelial proliferation.**

The diagnosis of ADH is made in those cases in which a diagnosis of **low grade** DCIS is seriously



For peer review only - http://bmjopen.bmj.com/site/about/guidelines.xhtml

considered but where the architectural, cytological and quantitative features do not amount to a confident diagnosis of DCIS. If a diagnosis of ADH is contemplated, extensive sampling and/or levels should be undertaken to search for more evidence to establish an unequivocal diagnosis of DCIS.

USEFUL RULES OF THUMB TO DISTINGUISH ADH FROM DCIS

- Restrict diagnosis of ADH to those cases in which DCIS is seriously considered but where the features are not sufficiently developed to make a confident diagnosis.
- DCIS usually extends to involve multiple duct spaces. If a lesion with features of ADH extends widely, the diagnosis of ADH should be questioned.

## Atypical Lobular Hyperplasia and Classical Lobular Carcinoma *In Situ* (*In Situ* Lobular Neoplasia)

Atypical lobular hyperplasia (ALH) and lobular carcinoma in situ (LCIS) have traditionally been separated as distinct entities based on cytological and quantitative features relating to the extent of lobular involvement. The justification for separating the entities is the differing risks of subsequent invasive cancer shown in long-term follow-up series, but molecular analysis suggests that biologically the two appear to be essentially similar and that these are neoplastic processes. In view of the subjective nature of separating ALH from LCIS and the similar molecular profiles, some experts have suggested that the two forms should be grouped together as 'lobular neoplasia' (in situ lobular neoplasia). However, for the purposes of recording risk of subsequent invasive carcinoma and for Cancer Registry recording, when the degree and extent can be assessed, for example in excision specimens, it is recommended that these are reported as separate entities. However, very mild forms of ALH can be found in association with fibrocystic change, involution and otherwise normal breast tissue.

As for ADH, assessment of extent and degree of acinar expansion by a lobular proliferation is required to distinguish atypical lobular hyperplasia (ALH) from lobular carcinoma in situ (LCIS). Even when LCIS has been diagnosed on core biopsy, the literature to date does not appear to indicate a different upgrade rate (risk of adjacent/associated invasive carcinoma or DCIS) and thus it is recommended that the term lobular neoplasia (encompassing both ALH and LCIS) is used in core biopsy specimens and a B3 category provided. The exceptions to this include pleomorphic



For peer review only - http://bmjopen.bmj.com/site/about/guidelines.xhtml

lobular carcinoma in situ (B5a) and mass-forming/florid LCICS (typically categorised as B4, suspicious).

In an excision specimen the pathologist should distinguish ALH from LCIS because of the reportedly different risk associated with subsequent carcinoma development.

In cytological terms, the two forms of the lesion are identical; in situ lobular neoplasia is characterised by proliferation within terminal duct lobular units of characteristic cells. The defining cell type is discohesive, round, cuboidal or polygonal with clear or light cytoplasm. Nuclei are small to moderate in size, round to oval and cytologically bland, with an occasional small inconspicuous nucleolus. The nucleus may be indented by an intracytoplasmic vacuole containing mucin. The cells have a high nuclear to cytoplasmic ratio. Mitotic figures and hyperchromatism are not often seen. There is an even distribution of cells and cellular monotony is the rule. Cytoplasmic clear vacuoles are often, although not invariably, present, sometimes having a central mucin blob. Pagetoid spread of cells may be present when the proliferation of neoplastic cells above the basement membrane undermines the normal lining epithelial cells.

The distension of lobular units may be variable from mild to gross, resulting in either patent lumina or complete obliteration. In atypical lobular hyperplasia there is minimal extension of less than half of the acini, whilst in lobular carcinoma in situ more than half of the acini within the TDLU are distended by an expansion of the typical cells (8 or more cells across each acinus).

Particularly in some more extensive lesions, distinction between in situ lobular neoplasia and DCIS may be difficult. For example, on occasions, a regular, evenly spaced monotonous population is seen within both ducts and lobules. E-cadherin membrane reactivity may be useful in such cases in distinguishing in situ lobular neoplasia from DCIS as the latter typically shows strong membrane positivity, whilst in the former expression is absent or weak. However, this marker can also be non-contributory or show varied levels within a case. Thus if both ducts and lobules contain epithelial proliferation of this type, particularly if E-cadherin is heterogeneous, categorisation as both LCIS and DCIS is recommended to imply the precursor risk of DCIS and the bilateral cancer risk of in situ lobular neoplasia. Such cases should also be recorded as DCIS **and** LCIS on the reporting form.

## Pleomorphic Lobular Carcinoma *In Situ*

Variants, particularly the pleomorphic subtype of lobular carcinoma in situ, are recognised. The discohesion and architecture of the classical form is present but the cells are larger and



For peer review only - http://bmjopen.bmj.com/site/about/guidelines.xhtml

pleomorphic (cytonuclear grade 3) with more abundant cytoplasm. Central necrosis and calcification are often seen. Mitoses may be seen, although uncommon in the classical form, and atypical forms may be noted. Pleomorphic LCIS is less frequently ER positive than classical forms and more often expresses HER2 oncoprotein. There is a paucity of information available on the clinical behaviour of pleomorphic LCIS, but it is widely regarded as a more aggressive form of the disease, which should be managed akin to DCIS rather than classical LCIS, based on its biological and molecular profile. As noted above, as for the 'classical' form, E-cadherin membrane negativity may be useful in confirming that the lesion represents a lobular lesion, rather than high grade DCIS.

## Extensive and Mass forming Classical LCIS with Necrosis

This rare variant of LCIS has classical cytology with central necrosis in distended acini. The degree of atypia is not sufficient for a diagnosis of pleomorphic LCIS. This variant is uncommon and the clinical behaviour is not well established.

In many centres both of the above entities are regarded as established forms of in situ carcinoma requiring therapeutic excision, equivalent to ductal carcinoma in situ (DCIS).

## Size of Lesion

If the size of the lesion has been measured by the histopathologists (for example using the Vernier scale) this should be completed on the Sloane Audit form. As ADH is, by definition, a small microfocal lesion, recording the size will allow some analysis of diagnostic criteria adherence. It is not anticipated that pathologists will routinely record the size of classical (as opposed to pleomorphic) LISN or of FEA. The size of pleomorphic LCIS should be completed as, at present, this is generally clinically managed akin to DCIS and the same dataset is collected.

## Margins

As, at present, pleomorphic LCIS is widely managed as per DCIS, the size of the lesion and the distance to margins should be recorded as for DCIS lesions.

If the pathologist has recorded that other atypical processes (FEA, ADH and classical LISN) extend to (or indeed has recorded the distance to) margins, this would be valuable information to collect and the Sloane Project Steering Committee would be



For peer review only - http://bmjopen.bmj.com/site/about/guidelines.xhtml

grateful to receive this data for subsequent follow-up analysis of clinical behaviour. It is not part of the routine NHSBSP minimum dataset, however, and we recognised that this is unlikely to be widely available/recorded.

## Receptor Measurement

The methods used for receptor assay should follow UK national guidance. Laboratories performing such assays should participate in the relevant UK NEQAS scheme.

## Lesions not included in the Sloane Atypia Audit

The above types of lesion are the only types of lesion that are being recorded in the Sloane Project Atypia Audit. We are no longer collecting data on DCIS, and have never recorded data on invasive disease. We are currently not collecting data on columnar cell change or columnar cell hyperplasia or usual epithelial hyperplasia.

For peer review only



For peer review only - http://bmjopen.bmj.com/site/about/guidelines.xhtml

### Validation checks

strErrorText
- Check # 01 entries have been found where the SLOANE ID is '0' in the operation details table.
- Check # 02 patient(s) do not have matching radiology details.
- Check # 03 radiology record(s) do not have matching patient records.
- Check # 04 radiology record(s) do not have a pathology record.
- Check # 05 pathology record(s) do not have a radiology record.
- Check # 06 radiology record(s) do not have a treatment strategy record.
- Check # 07 treatment strategy record(s) do not have a radiology record.
- Check # 08 appointment record(s) do not have a radiology record.
- Check # 09 appointment record(s) do not have an operation record.
- Check # 10 operation record(s) do not have an appointment record.
- Check # 11 treatment strategy record(s) do not have a radiotherapy record.
- Check # 12 radiotherapy record(s) do not have a treatment strategy record.
- Check # 13 appointment(s) are potentially duplicate records (Visit ID).
- Check # 14 operation(s) are potentially duplicate records (Visit ID).
- Check # 15 pathology record(s) are potentially duplicates.
- Check # 16 QARC ID(s) are potentially duplicates (Patient table).
- Check # 17 SLOANE ID(s) are potentially duplicates (Radiology table).
- Check # 18 radiotherapy ID(s) are potentially duplicates.
- Check # 19 treatment strategy ID(s) are potentially duplicates.
- Check # 20 patients have NHS numbers that do not conform to the NHS standard.
- Check # 21 patient(s) with more than one appointment (operation) recorded for the same day (this is unusual and should be checked for accuracy).
- Check # 22 patient(s) with more than two mammograms recorded (this is unusual and should be checked for accuracy).
- Check # 23 patient(s) with more than two mammograms recorded for the same side (this is unusual and should be checked for accuracy).
- Check # 24 patient(s) with the date of mammogram not recorded.
- Check # 25 patient(s) whose adjuvant therapy details are not recorded.
- Check # 26 patient(s) have clear margins but details are not recorded.
- Check # 27 case(s) of DCIS with no Highest Nuclear Grade recorded.

Check # 28 case(s) with multiple cut up protocols recorded.
Check # 29 case(s) with multiple specimen X-Ray types recorded.
Check # 30 case(s) where a lesion was excised without one being present.
Check # 31 case(s) without microcalcification that have a suspicious pattern recorded.
Check # 32 case(s) where a lesion was excised without a specimen X-Ray having been performed.
Check # 33 case(s) with no specimen X-Ray performed, but Diagnostic or Theraputic has been recorded.
Check # 34 case(s) where a lesion was present in the specimen X-Ray without one having been performed.
Check # 35 case(s) where a boost was given, but treated daily is unrecorded.
Check # 36 case(s) where an external beam was given, but axilla treated is unrecorded.
Check # 37 case(s) where an external beam was given, but boost given is unrecorded.
Check # 38 case(s) where both an external beam and brachytherapy were given.
Check # 39 case(s) where an external beam was given, but treated daily is unrecorded.
Check # 40 case(s) where treated daily, but boost given is unrecorded.

For peer review only

Check # 41 case(s) where treated daily, but external beam is unrecorded.
Check # 42 case(s) where axillary nodes not taken, but procedures have been recorded.
Check # 43 case(s) where axillary nodes are taken, but procedure is unrecorded.
Check # 44 case(s) where no further surgical treatment undertaken and no reason has been recorded.
Check # 45 case(s) where further surgical treatment undertaken and reason(s) for not have been recorded.
Check # 46 case(s) where no further excision was carried out, but was orientated.
Check # 47 case(s) where a further excision was carried out without a specimen X-Ray.
Check # 48 case(s) where the lesion was confirmed to the surgeon without a specimen X-Ray having been done.
Check # 49 case(s) where a lesion was present in the specimen X-Ray without one having been performed.
Check # 50 case(s) where the patient entered clinical trials, but a trial name has not been recorded.
Check # 51 case(s) where the patient refused clinical trials, but a trial name has been recorded.
Check # 52 case(s) where a grade hasn't been recorded against a surgeon.
Check # 53 case(s) where a surgeon's name hasn't been recorded against a grade.
Check # 54 case(s) where the recorded specimen sizes do not match the total size of the lesion.

- Check # 57 (AgeAtMammogram)
- Check # 58 (BiLateral_CheckDates)
- Check # 59 (BreastProcedure_10_or_14)
- Check # 61 (DCIS_TotalSpecimenSizes)
- Check # 62 (DOB_MammoDate_OpDates)
- Check # 63 (DuplicateQARCIDs)
- Check # 64 (DuplicateQARCIDs_NotBiLateral)
- Check # 65 (ExcisionalOperatorsIncorrect)
- Check # 66 (ExcisionalValuesIncorrect)
- Check # 67 (ImmedReconMoreThan2)
- Check # 68 (ImmedReconNoMx)
- Check # 69 (MoreThanOnePrimary)
- Check # 70 (MultipleImmediateReconstruction)
- Check # 71 (MultipleMx)
- Check # 72 (OperationsFinalOpNo)
- Check # 73 (OperationsMoreThan4)
- Check # 74 (Path_BiopsyNoDetail)
- Check # 75 (Path_CheckMicroinvasionPresent)
- Check # 76 (Path_CheckNoOfFociMicroinvasion)
- Check # 77 (Path_CompletionMxOnlyOneOp)
- Check # 78 (Path_CompletionMxWithMx)
- Check # 79 (Path_DCISMicroinvasionNull)
- Check # 80 (Path_DCISMicroinvasionUnknown)
- Check # 81 (Path_DCISNoComedoNecrosis)
- Check # 82 (Path_DCISSizeIncorrect)
- Check # 83 (Path_MarginClear)
- Check # 84 (Path_MxMarginCheck)
- Check # 85 (Path_MxNoOperation)
- Check # 86 (Path_NodesCheckBlanks)

For peer review only

- Check # 87 (Path_NodesCheckValues)
- Check # 88 (Path_NodesOverall)
- Check # 89 (Path_NodesPositive)
- Check # 90 (Path_UnknownHistology)
- Check # 91 (Pathology_DCISNoNuclearGrade)
- Check # 92 (Pathology_MultipleCutUpProtocols)
- Check # 93 (PreOpDiagnosis)
- Check # 94 (Radiology_MicrocalcificationNotPresentWithNottmDef)
- Check # 95 (Radiology_MicrocalcificationPresentNoNottmDef)
- Check # 96 (Radiology_MultipleSpecimenXRays)
- Check # 97 (Radiology_NoLesionPresentLesionExcised)
- Check # 98 (Radiology_NoMicrocalcificationWithNottmDefinition)
- Check # 99 (Radiology_NoSpecimenXRayLesionExcised)
- Check # 100 (Radiology_NoSpecimenXRayWithDiagOrTheraputic)
- Check # 101 (Radiology_NoSpecimenXRayWithLesionPresent)
- Check # 102 (RadiologyBackgroundPattern)
- Check # 103 (RadiologyCheckLesionValues)
- Check # 104 (RadiologyCheckLesionValuesErrorsDiameter)
- Check # 105 (RadiologyCheckLesionValuesErrorsDistance)
- Check # 106 (RadiologyCheckLesionValuesErrorsLength)
- Check # 107 (RadiologyCheckLesionValuesErrorsVolume_2r)
- Check # 108 (RadiologyCheckLesionValuesErrorsVolume_h)
- Check # 109 (RadiologyPredominantRadiologicalFeature)
- Check # 110 (Radiotherapy_BoostGivenYesTreatedDailyNull)
- Check # 111 (Radiotherapy_ExternalBeamYesAxillaTreatedNull)
- Check # 112 (Radiotherapy_ExternalBeamYesBoostGivenNull)
- Check # 113 (Radiotherapy_ExternalBeamYesBrachytherapyYes)
- Check # 114 (Radiotherapy_ExternalBeamYesTreatedDailyNull)
- Check # 115 (Radiotherapy_NoBoostTreatedDaily)
- Check # 116 (Radiotherapy_NoExternalBeamTreatedDaily)
- Check # 117 (RadiotherapyUnknown)
- Check # 118 (RadiotherapyYesFormNotReceived)
- Check # 119 (RadiotherapyYesWithMx)
- Check # 120 (ScreeningRound0NullOrMoreThan8)
- Check # 121 (ScreeningYearOfDiagnosis)
- Check # 122 (SLOANEIDCheckFirstAndLast)
- Check # 123 (Treat_AdjuvantTherapyYes)
- Check # 124 (Treat_AxProcedures)
- Check # 125 (Treatment_AxillaryNodesNotTakenWithProcedures)
- Check # 126 (Treatment_AxillaryNodesTakenWithNoProcedures)
- Check # 127 (Treatment_FurtherSurgeryNoWithNoReason)
- Check # 128 (Treatment_FurtherSurgeryYesWithReason)
- Check # 129 (Treatment_NoFurtherExcisionButOrientated)
- Check # 130 (Treatment_NoOperationDate)
- Check # 131 (Treatment_NoSpecimenXRayFutherExcisionCarriedOut)
- Check # 132 (Treatment_NoSpecimenXRayLesionConfirmedToSurgeon)

For peer review only

- Check #  133 (Treatment_NoSpecimenXRayLesionPresent)
- Check #  134 (Treatment_PatientNotEnteredTrialWithTrialName)
- Check #  135 (Treatment_PatientRefusedTrialAndEntered)
- Check #  136 (Treatment_SurgeonNoGrade)
- Check #  137 (Treatment_SurgicalGradeNoSurgeon)
- Check #  138 (TreatStrat_AdjuvantTherapyUnknown)
- Check #  139 (TreatStrat_RadiotherapyWithAdjuvantTherapy)
- Check #  140 (TreatStrat_RefereForRadioYesNoAdjuventYes)
- Check #  141 (TreatStrat_ReferForRadioYesMxYes)
- Check #  142 (TreatStrat_ReferForRadioYesNoRadioForm)
- Check #  143 (AgeCheck)
- Check #  144 (OpAttendanceDatesNoOperation)

For peer review only

| Type of further event | Definition |
|---|---|
| Further event | (any of): ipsilateral within breast recurrence (or new primary) after BCS; ipsilateral recurrence (includes post-mastectomy/chest wall recurrence); regional or distant recurrence; or contralateral re/occurrence. |
| Ipsilateral recurrence | First recurrence (DCIS or invasive) or new primary (DCIS or invasive) in the same breast, post mastectomy chest wall, or ipsilateral axillary nodes, occurring 6 months or later after surgery for the index lesion. |
| Ipsilateral breast recurrence | first within breast recurrence (DCIS or invasive) or new primary (DCIS or invasive) in the same breast or post mastectomy chest wall occurring 6 months or later after surgery for the index lesion. |
| Distant recurrence | recurrence outwith the breast or post-mastectomy chest wall, (i.e. distant metastatic disease or regional nodal recurrence (ipsilateral or contralateral) in the axilla or supraclavicular fossa) occurring 6 months or later after surgery for the index breast lesion. |
| Contralateral event | new primary (DCIS or invasive) in the contralateral breast or contralateral axillary nodes, occurring 6 months or later after surgery for the index lesion in the other breast. |

**Supplemental Table 1: Definitions of further events in the Sloane Project cohort**

For peer review only

| Cause of death | Definition |
|---|---|
| Breast cancer | Women were considered to have died from breast cancer if breast cancer appeared in the underlying cause of death column in the files available to, or in any section of part 1 (1a, 1b, or 1c) of the death certificate, or if metastases or carcinomatosis from breast cancer were recorded in part 1 of the death certificate, or if metastases or carcinomatosis were recorded in part 1 with breast cancer mentioned in part 2 and no other cancer identified. Women who died of breast cancer but who had no further events recorded were deemed to have had distant metastases on the date they died. |
| Other cancer | Women were considered to have an "other cancer" death if the underlying cause of death was cancer other than breast, or if one or more than one cancer at any site other than breast appeared in part 1 of the death certificate. Breast cancer may have appeared in part 2 or may not have appeared on the death certificate at all but it was not considered to be the underlying cause of death. |
| Cancer of unknown type | Women were considered to have died from an unknown type of cancer when they had metastatic cancer or carcinomatosis as the underlying cause of death, or if metastatic cancer or carcinomatosis was mentioned in part 1 of the death certificate but breast cancer or another type of cancer was not mentioned in any other section. Thus, cancer was known to be the cause of death, but the type of cancer was not known. |
| Non cancer (and non-circulatory) | Women were considered to have a non cancer (and non-circulatory) death if the underlying cause of death was not cancer and was not circulatory (circulatory was defined as any "I" ICD-10 code) or if the death was recorded as non cancer (and non-circulatory) in part 1 of the death certificate in the absence of cancer in part 1. If non cancer (and non-circulatory) cause of |

For peer review only

| | |
|---|---|
| | death was recorded in part 1 but cancer/or circulatory disease was noted in part 2, a check was done to see if it was likely the woman had died with, rather than because of, cancer/circulatory disease. |
| Non cancer (circulatory) deaths* | Women were considered to have a circulatory death if the underlying cause of death was a circulatory cause (defined as any "I" ICD-10 code) or a circulatory cause was recorded in part 1 of the death certificate in the absence of cancer in part 1. If cancer was identified in part 2, a check was done to ensure that it was clear that this could not considered to be the underlying cause. |
| Unknown cause of death | Unknown cause of death was recorded when the vital status and date of death had been reported but the cause of death had not been identified. |

**Supplemental Table 2: Definitions of cause of death in the Sloane Project**

*Non-circulatory deaths category included because of the potential for radiotherapy-induced circulatory events

For peer review only
